# Cardiovascular Disorders and Falls Among Older Adults: A Systematic Review and Meta-Analysis

**DOI:** 10.1093/gerona/glad221

**Published:** 2023-09-20

**Authors:** Robbie Bourke, Paul Doody, Sergio Pérez, David Moloney, Lewis A Lipsitz, Rose Anne Kenny

**Affiliations:** Department of Medical Gerontology, School of Medicine, Trinity College Dublin, Dublin, Ireland; Mercer’s Institute for Successful Ageing, St. James Hospital, Dublin, Ireland; Department of Medical Gerontology, School of Medicine, Trinity College Dublin, Dublin, Ireland; Nuffield Department of Primary Care Health Sciences, Medical Sciences Division, University of Oxford, Oxford, UK; Department of Medical Gerontology, School of Medicine, Trinity College Dublin, Dublin, Ireland; Mercer’s Institute for Successful Ageing, St. James Hospital, Dublin, Ireland; Department of Medical Gerontology, School of Medicine, Trinity College Dublin, Dublin, Ireland; Mercer’s Institute for Successful Ageing, St. James Hospital, Dublin, Ireland; Beth Israel Deaconess Medical Center, Harvard Medical School, Boston, Massachusetts, USA; Hinda and Arthur Marcus Institute for Aging Research, Hebrew Senior Life, Boston, Massachusetts, USA; Department of Medical Gerontology, School of Medicine, Trinity College Dublin, Dublin, Ireland; Mercer’s Institute for Successful Ageing, St. James Hospital, Dublin, Ireland; (Medical Sciences Section)

**Keywords:** Cardiovascular, Carotid sinus hypersensitivity, Hypertension, Orthostatic hypotension, Syncope

## Abstract

**Background:**

Falls are a common cause of injury, hospitalization, functional decline, and residential care admission among older adults. Cardiovascular disorders are recognized risk factors for falls. This systematic review assesses the association between cardiovascular disorders and falls in older adults.

**Methods:**

Systematic searches were conducted on MEDLINE and Embase, encompassing all literature published prior to December 31, 2022. Included studies addressed persons aged 50 years and older, and assessed the association between cardiovascular disorders and falls or the efficacy of cardiovascular-based interventions to reduce falls. Two reviewers independently extracted data and assessed study quality utilizing a modified Newcastle–Ottawa scale for observational studies, and the Cochrane Risk of Bias 2 tool for interventional studies. A systematic narrative analysis of all cardiovascular outcomes, and meta-analyses of unadjusted odds ratios (ORs) were performed.

**Results:**

One hundred and eighty-four studies were included: 181 observational and 3 interventional. Several cardiovascular disorders, including stroke, coronary artery disease, valvular heart disease, arterial stiffness, arrhythmia, orthostatic hypotension, and carotid sinus hypersensitivity, were consistently associated with falls. In meta-analysis of unadjusted ORs, the largest positive pooled associations with falls during a 12-month reporting interval were for stroke (OR: 1.90, 95% confidence interval [CI]: 1.70–2.11), peripheral arterial disease (OR: 1.82, 95% CI: 1.12–2.95), atrial fibrillation (OR: 1.52, 95% CI: 1.27–1.82), and orthostatic hypotension (OR: 1.39, 95% CI: 1.18–1.64).

**Conclusions:**

Several cardiovascular disorders are associated with falls. These results suggest the need to incorporate cardiovascular assessments for patients with falls. This review informed the cardiovascular recommendations in the new World Guidelines for falls in older adults.

Clinical Trials Registration Number: CRD42021272245

Falls are a growing global concern, and the World Health Organization (WHO) estimates that falls lead to 37 million hospitalizations each year (1). Falls incidence rises significantly with increased age and frailty (2). Moreover, falls are the most common cause of injury in older adults, frequently resulting in hospitalization, accelerated functional decline, admission to residential care (3), and increased mortality (4,5). Thirty-five percent of community-dwelling older adults fall at least once a year, rising to 50% among those in long-term care (6).

Over 3 million older people in the United States attend emergency departments (EDs) following a fall each year (7), and falls represent 10% of all ED presentations in those over the age of 65 (8,9). Falls can cause serious injuries, with 10%–20% of falls leading to fractures, dislocation, head injury, and death (6). Falls can also have profound psychological consequences, such as fear of falling, which is associated with poorer quality of life, social isolation, cognitive and physical decline, and negative mental health outcomes (10). As demographic aging rises, so too will the incidence and cost of falls, with direct implications for health care provision (11–15).

In 2006, the estimated medical cost of falls for people aged ≥65 in the United States was $20 billion (16). By 2015, this rose to $50 billion (17). The mean cost of an individual fall resulting in hospitalization has been estimated to be $14 000 (18), whereas the length of stay is on average 8 days longer if a patient has an in-hospital fall resulting in further costs (19). The overall burden of falls in both health care and community settings can be reduced by targeting known risk factors, including cardiovascular risk factors (20,21).

Several cardiovascular disorders are reported to be associated with falls in older adults. These include orthostatic hypotension (OH), hypertension, bradyarrhythmias (eg, sick sinus syndrome, and atrioventricular block), tachyarrhythmias (eg, atrial tachycardia including atrial fibrillation (AF) and ventricular tachycardia), carotid sinus hypersensitivity (CSH), and vasovagal syncope (VVS) (22,23).

Given the projected changing global demographics and the rising frequency of cardiovascular disease, the purpose of this review was to systematically explore the association between falls and common cardiovascular disorders in adults aged ≥50 years.

## Method

This systematic review and meta-analysis were designed and conducted in accordance with Preferred Reporting Items for Systematic Reviews and Meta-Analyses (PRISMA) standards (24,25). A comprehensive review protocol was developed, registered, and adhered to PROSPERO registration: CRD42021272245.

### Data Sources and Searches

Systematic searches were conducted on MEDLINE (Ovid) and EMBASE encompassing all available literature published prior to December 31, 2022, and supplemented with manual reference searches of all included articles (Supplementary Appendix 1).

### Study Selection

Eligible studies addressed persons aged 50 years and older, were published as primary research papers in peer-reviewed journals, measured falls as an outcome, included diagnosis or assessment of cardiovascular disorders and the association between cardiovascular disorders and falls, or provided a comparison of the prevalence of falls among individuals with and without specific cardiovascular disorders. Studies were ineligible if the sample comprised a specific disease or ­condition-defined population (eg, Parkinson’s disease, dementia); a full text was not available in the English language; the design was a case report or conference abstract. Interventional studies additionally included the efficacy of cardiovascular intervention on falls outcome but did not include studies examining falls in treated hypertension.

Title and abstract, and full-text screening were performed by 2 independent reviewers (R.B. and P.D.) using Covidence systematic review management software. During full-text screening, the reason for exclusion was recorded (Supplementary Appendix 2). Any conflicts were resolved by a third reviewer (S.P.). Studies of the same cohort were included only once, using the study with the most information about the cohort. If 2 or more studies utilized the same data set, only the first published study was included to prevent duplication.

The WHO definition for falls was used to operationally define falls within the review: “a fall is an event which results in a person coming to rest inadvertently on the ground or floor or other lower level” (26). Included prefixes to the word “fall” that appeared in the literature were also utilized including recurrent, accidental, nonaccidental, and injurious.

### Data Extraction and Quality Assessment

Data extraction was performed by 2 reviewers independently (P.D. and S.P.). Conflicts were resolved with a third reviewer (R.B.). Extracted data are available in Supplementary Tables 1–15.

Quality assessment (QA) was performed by 2 reviewers (S.P. and R.B.; Supplementary Appendix 3). Conflicts were resolved with a third reviewer (P.D.). Observational studies were assessed using a modified version of the Newcastle–Ottawa scale (27) and were classified according to the following scoring system: 0–3 = low quality, 4–6 = intermediate quality, 7–10 high quality. Interventional studies were assessed using the Revised Cochrane Risk of Bias tool for randomized trials (RoB2) (28), and were classified as “low risk of bias,” “some concerns,” or “high risk of bias.”

### Data Synthesis and Analysis

A systematic narrative analysis of all outcomes was performed with findings presented in both textual and tabular formats. Further, random-effects meta-analyses of studies with unadjusted odds ratios (ORs; reported or calculated) were performed using Review Manager (RevMan version 5.4, The Cochrane Collaboration, the Nordic Cochrane Centre, Copenhagen, Denmark). A 12-month reporting interval was chosen for main and stratified analyses due to its ubiquity in reported studies and the likelihood that any cardiovascular disorders causing falls would likely do so within a year’s time period of follow-up (Supplementary Figures 1–8). Stratified analyses by age (50–64, 65–79, and ≥80 years), setting (community, hospital, and residential care) and assessment method were performed for each disorder (Supplementary Figures 9–26). Secondary analyses were also performed for alternative reporting intervals where relevant, for example, 1 month, 6 months, and 24 months (Supplementary Figures 27–33). Meta-analyses of unadjusted ORs were favored over adjusted ORs due to the heterogeneous nature of the adjusted analyses (29,30). Nonmeta-analysis forest plots for adjusted ORs are available as Supplementary Figures 34–39.

## Results

**Figure 1.**
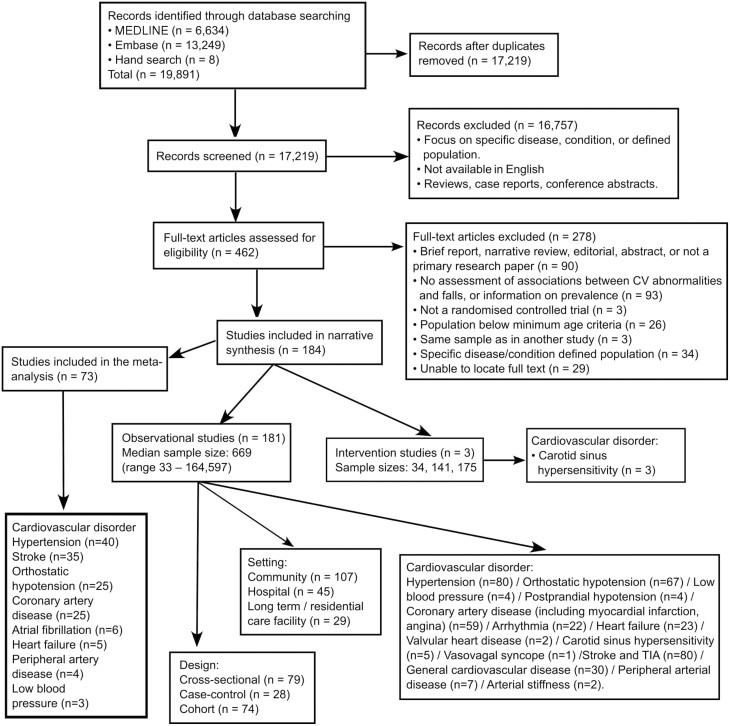
PRISMA flow diagram of systematic review and meta-analysis process and studies description.

Systematic searches yielded a combined total of 19 891 results of which 184 studies were included: 181 observational and 3 interventional (Figure 1). Seventy-three studies were included in meta-analyses based on the availability of unadjusted ORs.

Descriptive details of the 184 included studies are displayed in Supplementary Tables 1A and 1B.

Overall, there were consistent associations between cardiovascular disorders and falls. Certain subgroups of cardiovascular disorders were more consistently associated with falls than others such as stroke, coronary artery disease (CAD), valvular heart disease, arterial stiffness, arrhythmia, OH, and CSH. There was a wide variation in sampling frames, study designs, reporting interventions, assessment methods, and QA scores. CSH was the only disorder for which there were eligible interventional studies.

For the purposes of narrative presentation, cardiovascular disorders have been subdivided into 4 distinct categories (Supplementary Table 1C):

1. Blood-pressure-related disorders
2. Cardiac (structural and arrhythmia)
3. Reflex syncope
4. Other

### Blood-Pressure-Related Disorders

Overview: Conditions directly related to blood pressure, both hypertension and hypotension, demonstrate a somewhat inconsistent association with falls. The association between falls and OH is more consistent when contemporary methods of measurement for OH are applied.

#### Hypertension

Eighty *observational studies* investigated the association between hypertension and falls. Sixty studies did not report an association (31–90): QA 7.0 (range 4–10). Twenty reported a significant association: 17 positive (91–107) and 3 negative (108–110). Mean QA score was 6.9 (range 5–8) for positive and 6.3 (range 5–9) for negative studies (Supplementary Table 2).

Of note the 3 largest studies (*n* > 14 000) demonstrated an inconsistent association, with intermediate to high QA scores. The largest of these studies (*n* = 43 367) demonstrated a positive multivariate association with falls and had a high QA score. The next largest (*n* = 17 712) demonstrated no association and had a high QA score. Finally, the third largest (*n* = 14 881) demonstrated a univariate association with an intermediate QA score.

#### Orthostatic hypotension

Sixty-seven *observational studies* investigated OH and falls (Supplementary Table 3A). OH was assessed using ­beat-to-beat (BTB) measurement in 15 studies (46,55,68,111–122), an oscillometric sphygmomanometer in 22 (42,45,58,61,71,72,75,84,123–136), and an auscultatory sphygmomanometer in 11 studies (79,103,109,137–144). Thirteen studies utilized a sphygmomanometer but did not specify the type (36,43,60,122,145–153). The measurement instrument was unspecified in 8 studies (32,39,67,85,101,153–155). One study (122) measured OH with both BTB and a sphygmomanometer.

##### Results by measurement technique

Of 15 studies utilizing BTB, 12 reported a positive association, QA 7.4 (range 5–9) (68,111–117,119–122); 3 reported no association: QA 7.7 (range 7–8) (46,55,118).

Of 22 studies using an oscillometric sphygmomanometer, 3 studies reported a positive association: QA 10, 7, 7 (126,130,135). Nineteen studies reported no association: QA 7.1 (range 4–9) (42,45,58,61,71,72,75,84,123–125,127–129,131–134,136).

Of 11 studies using an auscultatory sphygmomanometer, 3 studies reported a positive association: QA 9, 6, 7 (138,140,142). The remaining 9 studies reported no ­association: QA 7.2 (range 5–9) (79,103,109,137,139,141–144). Aydin et al. (142) were counted in 2 of these analyses as the authors reported OH both in supine-to-tilted and ­supine-to-standing positions.

Of 13 studies that utilized sphygmomanometers, but did not specify which type, 6 reported a positive association: QA 6.8 (range 5–9) (36,145,147,150,151,153). Seven reported no association: QA 7.5 (range 4–9) (43,60,122,146,148,149,152).

Seven studies did not provide any details on the measurement instrument or assessment position. Five reported no association: QA 7.6 (range 7–10) (32,39,67,154,155). Two reported a significant positive association:QA 7, 8 (85,101).

##### Results by OH measurement instrument and assessment position

Supplementary Table 3B classifies studies by OH measurement instrument and assessment position. Overall, 80.0% (12/15) of studies using BTB showed a positive association between OH and falls, compared to 25.5% (12/47) of studies using any type of sphygmomanometer. In relation to the assessment position, 35.6% (16/45) of studies using supine to standing showed a positive association between OH and falls, compared to 75.0% (6/8) using supine to tilt (on a tilt bed) and 20% (1/5) using sitting to standing.

When OH was measured using the BTB method, the majority showed an association between OH and falls. However, no consistent association was noted when other OH measurement techniques were used. Of note, postural change from supine to standing demonstrated a more consistent association than sitting to standing.

#### Low blood pressure

Four *observational studies* investigated low blood pressure and falls. Three studies reported no significant association each with a QA score of 8 (70,156,157). One reported a positive association: QA 5 (158) (Supplementary Table 4). There was an inconsistent association between low blood pressure and falls in the limited number of studies available.

#### Postprandial hypotension

Four *observational studies* examined postprandial hypotension and falls. Two identified a positive association: QA 5, 8 (159,160). Two studies did not report an association: QA 6, 8 (161,162) (Supplementary Table 5).

There were a limited number of studies, and each had a small sample size. The literature remains inconclusive.

### Cardiac (Structural and Arrhythmia)

Cardiac disorders included CAD, arrhythmia, heart failure, and valvular heart disease. Two studies that evaluated valvular heart disease found a positive association with falls.

#### Coronary artery disease

Sixty *observational studies* investigated CAD and falls. Forty-two reported no association: QA 7.3 (range 4–10) (32–34,37,39,40,43,44,52,57,60,61,67,68,71–73,75–77,80,81,85,87–89,93,99,106–109,126,129,154,163–166). Eighteen reported a significant association: 17 positive, QA 6.8 (range 4–8) (49,58,62,63,83,94,104,167–175), 1 negative, QA 6 (176) (Supplementary Table 6).

A positive association was found in the 3 largest studies (with *n* > 100 000 participants and high QA scores), otherwise, no consistent association was noted.

#### Heart failure

Twenty-three *observational studies* examined heart failure and falls. Fifteen studies showed no association: QA 6.9 (range 4–10) (33,37,60,61,67,68,76,81,83,85,88,129,168,171,176). Eight identified a positive association: QA 6.8 (range 4–8) (40,47,49,51,59,100,169,177) (Supplementary Table 7).

The majority of studies showed no association with falls; of note, the 4 largest studies (*n* > 10 000 participants) showed inconsistent associations (2 positive, with high QA, and 2 negative, with intermediate to high QA).

#### Arrhythmia

Twenty-two *observational studies* investigated cardiac arrhythmias and falls: 13 regarding AF (37,48,60,61,67,68,76,85,174,178–181), 1 ventricular arrhythmia (182), 1 atrioventricular block (76), 1 sinus bradycardia (76), and 6 unclassified arrhythmias (33,40,81,88,183,184).

Of the 13 studies that examined AF, 8 reported no association, QA 7.6 (range 6–10) (37,60,61,67,68,76,85,181) and 5 reported a positive association, QA 6.4 (range 5–8) (48,174,178–180). No association was evident for ventricular arrhythmia, QA 8 (182), and atrioventricular block or sinus bradycardia, QA 10 (76).

Of 6 studies investigating arrhythmias, in general, 3 reported positive associations, QA 8, 4, 6 (33,40,183); and 3 reported no association, QA 7, 4, 6 (81,88,184) (Supplementary Table 8).

The majority of studies showed no association with falls; however, a positive association was found in the 2 largest studies (*n* > 25 000 participants, and intermediate to high quality).

#### Valvular heart disease

Two *observational studies* examined valvular heart disease. One study reported a positive association between mitral, tricuspid, and pulmonary valve regurgitation and falls, QA 9 (185). A second study reported a positive association between “heart murmurs” and falls, QA 8 (33) (Supplementary Table 9).

### Reflex Syncope

Reflex (or neurally mediated) syncope includes CSH and VVS. The pathophysiology for these conditions is similar, with both conditions characterized by hypotension and/or bradyarrhythmia. There were limited observational studies for both conditions. CSH showed an inconsistent association with falls, whereas VVS showed no association in a single study.

In the interventional studies for CSH, it was noted that implantation of a device, be it a permanent pacemaker (PPM) switched on or off, or an implantable loop recorder (ILR), and a corresponding decrease in falls rates.

#### Carotid sinus hypersensitivity

Five *observational studies* investigated CSH and falls. Three reported no association: QA 6, 7, 8 (162,186,187). Two reported a positive association: QA 6, 7 (111,188) (Supplementary Table 10).

Three *interventional studies* examined pacemaker intervention for falls reduction. In the first study, falls were reduced in paced patients compared to controls over a 12-month follow-up (189). In the second, cross-over interventional design (PPM on, PPM off), there was no difference in fall rates (190). A third study compared fall rates in patients with pacemakers against controls (ILR). The rate of falls was significantly reduced in both groups compared to the run-in period (191). The RoB2 QA for each of these studies concluded “some concerns” of bias (Supplementary Table 1B). However, the studies were underpowered and therefore deemed of low quality.

#### Vasovagal syncope

Only 1 study was included which reported no association with falls, QA 6 (186) (Supplementary Table 11).

### Other

This grouping of other vascular conditions includes stroke/transient ischemic attack (TIA), general cardiovascular disease, peripheral vascular disease, and arterial stiffness. Stroke was associated with falls in half the included studies, although each of the 2 studies that evaluated arterial stiffness demonstrated a positive association. “General cardiovascular disease” was a term used in 30 studies when specific conditions were not clarified, which demonstrated an inconsistent association with falls. Peripheral vascular disease was not associated with falls.

#### Stroke/transient ischemic attack

Eighty-two *observational studies* investigated stroke/TIA and falls. Forty-four reported a significant association: 42 positive, QA 7.0 (range 4–10) (32,37,43,54,57,59,62,64,66,72,73,76–78,82,87,89,93,101,104,147,154,155,158,163,167,170–172,176,183,192–202); 2 negative, QA 7, 7 (203,204). Thirty-seven studies reported no association: QA 7.2 (range 4–10) (31,33–35,38,39,41,51,52,56,60,61,65,67,69,71,80,81,83,85,88,90,95,106,107,112,126,129,164–166,173,205–209) (Supplementary Table 12).

Half of the studies demonstrated a positive association with falls. Of note, the 4 largest studies (*n* > 100 000 participants and QA scores intermediate to high) showed a positive association.

#### General cardiovascular disease

Thirty *observational studies* investigated general cardiovascular disease (unspecified) and falls. Seventeen studies reported no association: QA 7.2 (range 5–10) (35,38,53,54,64,82,86,89,109,112,125,126,156,209–212). Thirteen reported a positive association: QA 6.9 (range 4–8) (47,65,66,69,74,94,102,163,172,177,206,213,214) (Supplementary Table 13). The majority of studies reported no association with falls. However, due to limited and inconsistent operational definitions for the term “general ­cardiovascular disorders” throughout the literature, it is difficult to provide further elucidation upon these specific results.

#### Peripheral arterial disease

Seven *observational studies* examined peripheral arterial disease and falls. Six studies reported no association: QA 7.3 (range 6–8) (61,72,85,95,173,209), whereas 1 reported a negative association: QA 10 (154) (Supplementary Table 14).

#### Arterial stiffness

Two *observational studies* investigating arterial stiffness and falls reported a positive association: QA 9, 8 (72,75) (Supplementary Table 15).

Both are high-quality studies. The measurement techniques utilized were carotid–femoral pulse wave velocity and ­cardio-ankle vascular index, respectively.

### Meta-Analysis of Unadjusted ORs

Eight cardiovascular disorders were eligible for inclusion in a meta-analysis of unadjusted ORs for falls. Results are displayed in Table 1 and Supplementary Figures 1–8).

**Table 1.**
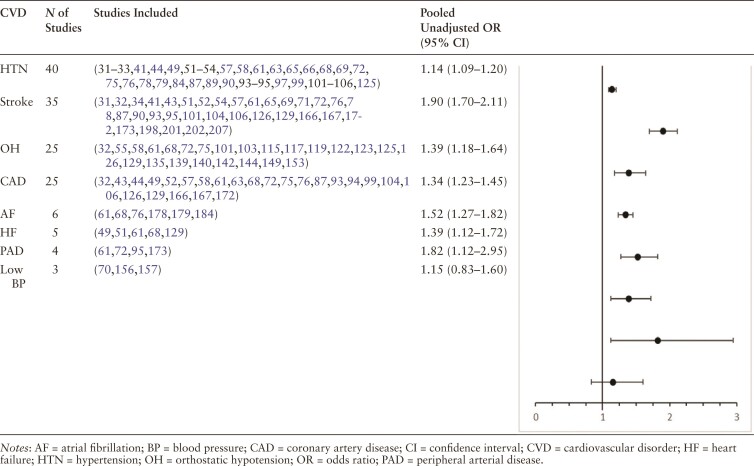
Meta-Analysis of Unadjusted OR for Falls Among Older Adults With Cardiovascular Disorders Within a 12-Month Period

### Stratified Analyses

Stratified analysis by the age category, study setting, assessment method, and time intervals were conducted when sufficient data were available. These produced no significant differences in the association between any cardiovascular disorder and falls (Supplementary Figures 9–39). There was a significant difference in stratified analysis by the ­assessment method for OH, contrasting assessment by sphygmomanometer (OR: 1.26, 95% confidence interval [CI]: 1.03–1.53) versus BTB (OR: 1.96, 95% CI: 1.40–2.73; *p≤*.02; Figure 2).

**Figure 2.**
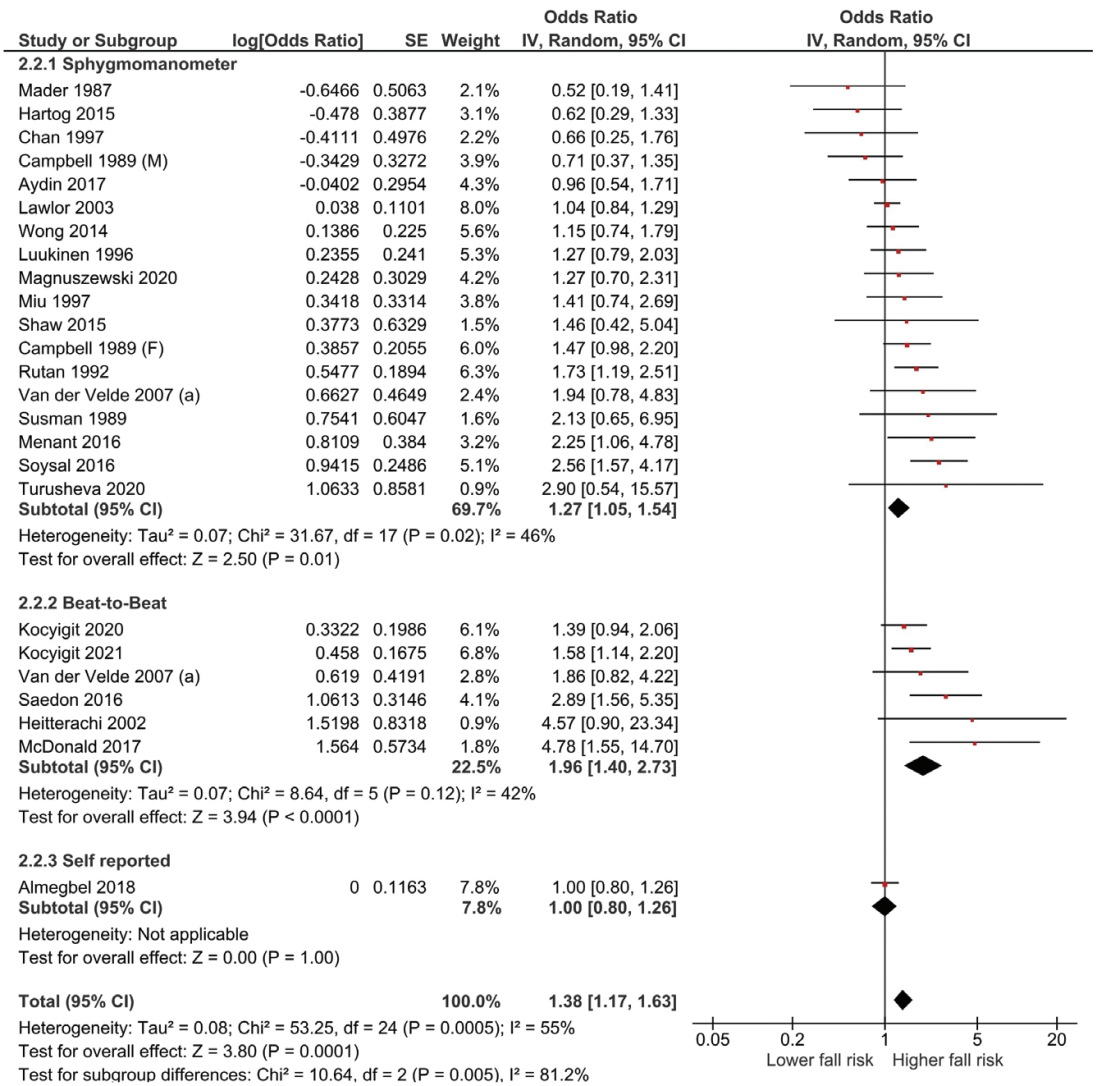
The association between orthostatic hypotension and falls among adults aged 50+ stratified by assessment method.

## Discussion

There are several key findings from this systematic review and meta-analysis with potential clinical implications: This review clearly demonstrates an association between falls and several cardiovascular disorders, including stroke, peripheral arterial disease, AF, OH, heart failure, CAD, and hypertension.

To the authors’ knowledge, there has been 1 previous systematic review examining the association between cardiovascular disorders and falls (22). This present review updates the literature in this area and builds on the prior review by increasing the scope of cardiovascular disorders examined, performing meta-analysis of unadjusted ORs, and including interventional trials, resulting in a more expansive review increasing from 86 studies to 184. This review was also used to inform the newly published Global Guidelines for Falls in Older Adults (215).

A recent consensus statement from the American Heart Association (216) emphasized that the association between cardiovascular risk factors and falls is poorly understood. The pathophysiological mechanisms that underpin the predisposition of older adults with cardiovascular disease to falls are complex. Mechanisms such as OH, tachyarrhythmia, and bradyarrhythmia may cause falls through frank syncope or alternatively, transient disruption of gait and balance through transient cerebral hypoperfusion without frank syncope. If syncope is unwitnessed, as is the case in many older people (217,218), and the person has amnesia or loss of consciousness (219), the clinical interpretation for syncope may be “a fall” (218,220). Gait and balance disorders (221) are common in fallers—present in over 60% of adults aged >80 years (222). The overlap between gait and balance problems and conditions that may lead to transient cerebral hypoperfusion (OH in particular) have been implicated in falls in older adults (223,224).

Other cardiovascular disorders such as hypertension, ischemic heart disease, and heart failure may share pathophysiological substrates, such as vascular damage to neural pathways governing gait and balance, thereby predisposing to falls (225). These disorders are often accompanied by medications that may increase the likelihood of falls (226). The possible associations between medications and falls, however, were beyond the scope of this review.

Hypertension is common with advanced age: 63% of adults aged >60 years in the United States are hypertensive (227). The association between hypertension and falls is not consistent in the observational literature. In the ­meta-analysis, there was an overall significant positive association between hypertension and falls, although only persisting within the stratified analysis for self-reported hypertension, community-dwelling adults, and adults under 80 years (Supplementary Figure 16). It is possible that the association between hypertension and falls was due to the hypotensive effects of hypertension itself or medications used to treat hypertension (228,229) or the results of hypertensive heart disease with left ventricular hypertrophy, reduced diastolic ventricular filling, and an associated decrease in cardiac output during preload reduction.

Regarding OH, 2 important findings have emerged from this review. First, the association between fallers and OH varied and was dependent on the method of measurement and the position of the patient during the assessment. Studies using a BTB measurement demonstrated a stronger association than traditional sphygmomanometer-based methods. In the meta-analysis of unadjusted ORs, there was also a significant difference in stratified analysis by assessment method (BTB: OR = 1.96 [95% CI: 1.4–2.73], Sphygmomanometer: OR = 1.27 [95% CI: 1.05–1.54]; *p* = .02). BTB blood pressure measurement allows clinicians to accurately assess blood pressure changes within the first minute of standing, therefore, capturing early transient changes. Recent studies have identified “OH 40” (the failure of blood pressure to return to baseline within 40 seconds of standing) to be associated with greater falls risk, including a higher risk of injurious falls (113,114). The definition of OH applied using rapid changes in systolic blood pressure during the first minute compared with changes during the first 3 minutes may have influenced this outcome. This new technology is more complex to use and more time consuming than traditional technology (sphygmomanometer) and is not presently widely available. BTB measurement is adept at measuring rapid and transient changes in blood pressure behavior and giving more granular information on different orthostatic hemodynamic patterns (230).

Meta-analysis demonstrates that studies using postural change from supine to standing show a significant association between OH and falls OR: 1.3 (95% CI: 1.06–1.6), but not sitting to standing OR: 1.36 (95% CI: 0.89–2.09), which suggest that initial supine measurements should be the preferred measurement choice (131,231).

The only accurate way to confirm a cardiovascular risk factor is to show that interventions that remove that risk reduce the incidence of falls. In this context, one would think that pacemakers could eliminate the bradycardia associated with CSH and reflex syncope, but 3 intervention studies (189–191) demonstrated that the presence of an implanted device whether a pacemaker in an active or inactive state, or an ILR, effectively reduced the rate of falls, even if they didn’t eliminate bradycardic episodes. Therefore, there may be additional neuropsychological contributions to these types of falls, consistent with previous literature on reflex syncope (232,233).

Given the association between cardiovascular disorders and falls, we concur with both the European Society of Cardiology Syncope Guidelines and the Global Guidelines for Falls in Older Adults (215,234) that the initial falls assessment should include a review of cardiovascular history, cardiac auscultation, surface electrocardiogram, and lying and standing blood pressure measurement. Additionally, BTB measurement should be employed, where possible.

This systematic review has several strengths and important findings. We have applied rigorous eligibility criteria, included interventional trials, and collated results to offer a comprehensive narrative synthesis generating novel findings (notably for OH and CSH). We have also applied a random-effect ­meta-analysis of unadjusted ORs to conditions where appropriate comparable data were available. This has allowed for a quantitative component to be included in this review. These findings have potentially useful clinical implications for falls risk, suggest directions for future research, and provide a systematic evidence base for the recent Global Guidelines for Falls in Older Adults (215).

The majority of the studies included in the review are observational and as such it is not possible to draw definitive causal inferences from these associations. Most of the studies assessed falls retrospectively by self-report and clinical notes. We recommend well-designed prospective studies that account for the complexity of vulnerable cohorts (ie, ­co-occurrence of cardiovascular risk factors such as hypertension, OH, and CSH) and heterogeneity of older fallers (co-occurrence of noncardiovascular falls risk factors) in order to provide more definitive clinical guidance.

We did not include cardiovascular medications as this topic has already been dealt with comprehensively (228,235,236). In brief, whereas loop diuretics, as a treatment for heart failure, are associated with falls, other cardiovascular medications demonstrate an inconsistent association (237). A recent meta-analysis of clinical trials showed that intensive lowering of blood pressure over the long term with antihypertensive medications was not associated with an increased risk of OH (229), although short-term effects or association with falls was not examined.

Other cardiovascular disorders such as hypertrophic cardiomyopathy, micturition syncope, and defecation syncope have been associated with falls in case reports and experimental literature but were not captured by the search strategy (83,238,239). Likewise, the combination of factors such as OH, medications, and postprandial hypotension can lead to falls in clinical practice in a given individual but this was not captured in our search.

A further challenge encountered in the review process was the inconsistency in operational definitions and nomenclature of key phrases used in studies, making it difficult for clear clinical inferences. For example, the term “cardiovascular disease” was used in 30 studies without defining, or providing distinctions to, exactly what cardiovascular disorders were being referred to and analyzed (74,172,206). CAD is a similarly imprecise term used in 19 studies, the majority of which did not show an association with falls. It is difficult to make any clinical recommendations in these instances given the lack of specificity of these terms. Similarly, there was a lack of clinical time stamping in conditions such as AF or congestive heart failure (40,47,48). Our interpretation of the methodologies is that these refer to chronic as opposed to acute conditions.

Also, the definition of “falls” was inconsistent, with few studies making a distinction between types of falls (ie, accidental and nonaccidental falls, or explained and unexplained falls). We included all of these subcategories in our analysis. These definitions also do not include loss of consciousness. Given the overlap between unwitnessed falls and syncope (240,241), clearer definitions around the loss of consciousness and witnessed events will greatly enhance the literature.

## Conclusion

There is a positive association between most common cardiovascular disorders and falls in adults aged over 50 years. These findings provide physicians with potential targets for assessment and intervention for falls risk in clinical practice. They also highlight the need to further deepen our understanding of this complex association between cardiovascular disorders and falls in older adults with well-constructed interventional studies.

## Supplementary Material

glad221_suppl_Supplementary_Material
