# Supplementary material for: Cardiovascular Disorders and Falls Among Older Adults: A Systematic Review and Meta-Analysis
Source: J Gerontol A Biol Sci Med Sci. 2023 Sep 20;79(2):glad221. doi: 10.1093/gerona/glad221 (PMC10809055; doi:10.1093/gerona/glad221)
Supplement: glad221_suppl_Supplementary_Material [file glad221_suppl_supplementary_material.pdf]

Online only supplementary material:

eTable 1A. Selected characteristics of all 181 included observational studies

eTable 1B. Selected characteristics of all three included interventional studies

eTable 1C. Cardiovascular disorders and falls

eTable 2. Hypertension and falls

eTable 3A. Orthostatic hypotension and falls

eTable 3B. OH and falls by measurement instrument and assessment position

eTable 4. Low blood pressure and falls

eTable 5. Postprandial hypotension and falls

eTable 6. Coronary artery disease and falls

eTable 7. Heart failure and falls

eTable 8. Arrhythmia and falls

eTable 9. Valvular heart disease and falls

eTable 10. Carotid sinus hypersensitivity and falls

eTable 11. Vasovagal syncope and falls

eTable 12. Stroke/Transient ischemic attack and falls

eTable 13. General cardiovascular disease and falls

eTable 14. Peripheral arterial disease and falls

eTable 15. Arterial stiffness and falls

eFigure 1. Association between hypertension and falls (unadjusted OR)

eFigure 2. Association between stroke and falls (unadjusted OR)

eFigure 3. Association between orthostatic hypotension and falls (unadjusted OR)

eFigure 4. Association between coronary artery disease and falls (unadjusted OR)

eFigure 5. Association between atrial fibrillation and falls (unadjusted OR)

eFigure 6. Association between heart failure and falls (unadjusted OR)

eFigure 7. Association between peripheral artery disease and falls (unadjusted OR)

eFigure 8. Association between low blood pressure and falls (unadjusted OR)

eFigure 9. Association between hypertension and falls (unadjusted OR) stratified by age

eFigure 10. Association between stroke and falls (unadjusted OR) stratified by age

eFigure 11. Association between orthostatic hypotension and falls (unadjusted OR) stratified by age

eFigure 12. Association between coronary artery disease and falls (unadjusted OR) stratified by age

eFigure 13. Association between heart failure and falls (unadjusted OR) stratified by age

eFigure 14. Association between peripheral artery disease and falls (unadjusted OR) stratified by age

eFigure 15. Association between low blood pressure and falls (unadjusted OR) stratified by age

eFigure 16. Association between hypertension and falls (unadjusted OR) stratified by setting

eFigure 17. Association between stroke and falls (unadjusted OR) stratified by setting

eFigure 18. Association between orthostatic hypotension and falls (unadjusted OR) stratified by setting

eFigure 19. Association between coronary artery disease and falls (unadjusted OR) stratified by setting

eFigure 20. Association between heart failure and falls (unadjusted OR) stratified by setting

eFigure 21. Association between hypertension and falls (unadjusted OR) stratified by assessment method

eFigure 22. Association between stroke and falls (unadjusted OR) stratified by assessment method

eFigure 23. Association between coronary artery disease and falls (unadjusted OR) stratified by assessment method

eFigure 24. Association between heart failure and falls (unadjusted OR) stratified by assessment method

eFigure 25. Association between peripheral artery disease and falls (unadjusted OR) stratified by assessment method

eFigure 26. Association between low blood pressure and falls (unadjusted OR) stratified by assessment method

eFigure 27. Association between hypertension and falls (unadjusted OR) stratified by reporting interval

eFigure 28. Association between stroke and falls (unadjusted OR) stratified by reporting interval

eFigure 29. Association between orthostatic hypotension and falls (unadjusted OR) stratified by reporting interval

eFigure 30. Association between coronary artery disease and falls (unadjusted OR) stratified by reporting interval

eFigure 31. Association between heart failure and falls (unadjusted OR) stratified by reporting interval

eFigure 32. Association between peripheral artery disease and falls (unadjusted OR) stratified by reporting interval

eFigure 33. Association between low blood pressure and falls (unadjusted OR) stratified by reporting interval

eFigure 34. Association between hypertension and falls (adjusted OR)

eFigure 35. Association between stroke and falls (adjusted OR)

eFigure 36. Association between orthostatic hypotension and falls (adjusted OR)

eFigure 37. Association between coronary artery disease and falls (adjusted OR)

eFigure 38. Association between arrhythmia and falls (adjusted OR)

eFigure 39. Association between heart failure and falls (adjusted OR)

Appendix 1. Systematic search strategy

Appendix 2. Full text screening table of excluded studies

Appendix 3. Quality assessments

eTable 1A. Selected characteristics of all 181 included observational studies

First author	Year	Design	Setting	Data gathering	Falls outcome	Falls assessment method	Falls reporting interval	N	Age, Mean (SD)/ Median (IQR range)*	% female	Exposure(s)	NOS Score
Abbs (171)	2020	Cohort	Community (homeless aged 50+)	Prospective	Any fall	Retrospective (self reported)	Six months prospectively and retrospectively	350	Median = 58 (IQR = 54-61)	22.9%	Myocardial infarction; congestive heart failure; stroke	7
Aburub (210)	2021	Cross sectional	Community	Cross sectional	Any fall	Retrospective (self reported)	Previous 12 months	860	>65	50.5%	Cardiovascular disease	7
Akande-Sholabi (31)	2020	Cross sectional	Hospital outpatients department	Cross sectional	Any fall	Retrospective (self reported)	Previous 12 months	400	72.4 (SD = 7.3)	63.7%	Hypertension; stroke	6
Alamgir (211)	2015	Cross sectional	Community (home)	Cross sectional	Any fall	Retrospective	Past 3 months	5,996	65+	Not reported	Cardiovascular disease	5
Almegbel (32)	2018	Cross sectional	Community	Cross sectional	Any fall	Retrospective (self reported)	Previous 12 months	1,182	68.8 (SD = 9.0)	53.9%	Coronary artery disease; stroke / transient ischemic attack; hypotension; hypertension	7
Anpalahan (186)	2012	Case-control	Acute hospital	Retrospective	Unexplained falls and accidental falls	In ER or admitted for falls	Falls leading to hospital admission	21 / 17	80 (SD = 6) / 77 (SD = 5)	55.0%	Carotid sinus hypersensitivity; Vasovagal syncope	6
Aronow (159)	1997	Cohort	Long-term care	Prospective	Any fall	Incident reports	29 (SD = 10) months	499	80 (SD = 9)	Not reported	Postprandial hypotension	5
Arseven (154)	2008	Cohort	Community (Primary care)	Prospective	Non-syncopal falls	Prospective (self reported)	9.6 (SD = 2.9) months	168	70.0 (SD = 9.0)	46.0%	Peripheral arterial disease; cardiac disease; stroke; orthostatic hypotension	10
Assantachai (91)	2003	Cross sectional	Community (home)	Cross sectional	Any fall	Retrospective	Past 6 months	1,043	Men 69.0 (SD = 6), women 68.0 (SD = 7)	64.0%	Hypertension	6
Aydin (142)	2017	Cross sectional	Outpatient clinic	Cross sectional	Reoccurring falls	Retrospective (self reported or reported by patients relatives)	Previous 12 months	290	> 65	59.5%	Orthostatic hypotension	7
Banu (80)	2018	Case-control	Community	Cross sectional	Injurious falls	Retrospective (electronic medical records)	3 years	210	78.1 (SD = 8.3)	60.4%	Hypertension; stroke or transient ischaemic attack; Ischaemic heart disease	8

First author	Year	Design	Setting	Data gathering	Falls outcome	Falls assessment method	Falls reporting interval	N	Age, Mean (SD)/ Median (IQR range)*	% female	Exposure(s)	NOS Score
Bergland (92)	2003	Cohort	Community (home)	Prospective	Any fall	Prospective	3-monthly for 12 months	307	81.0 (Range 75–93)	100.0%	Hypertension	8
Bhangu (33)	2017	Cohort	Community	Prospective	All falls, non-accidental falls, syncope	Retrospective (self reported)	Previous 24 months	8,172	63.7 (SD = 9.7)	55.6%	Hypertension; angina; myocardial infarction; heart failure; stroke; transient ischemic attack; heart murmur; arrhythmia	8
Brassington (102)	2000	Cross sectional	Community (home)	Cross sectional	Any fall	Retrospective	12 months	1,526	64–99	64.0%	Hypertension; cardiovascular disease	6
Bumin (145)	2002	Cross sectional	Long-term care	Cross sectional	Any fall	Retrospective	Any previous fall	33	fallers 73.0 (SD = 2), non-fallers 68.0 (SD = 2)	Not reported	Orthostatic hypotension	5
Callisaya (34)	2014	Cohort	Community	Prospective	Multiple falls	Prospective (self reported)	12 months	665	74.5 (SD = 6.7)	58.7%	Hypertension; myocardial infarction; stroke	10
Campbell (146)	1981	Cross sectional	Community (home and residential facility), Acute hospital	Cross sectional	Any fall	Retrospective	Past 12 months	559	65+	Not reported	Orthostatic hypotension	4
Campbell (123)	1989	Cohort	Community (home and residential facility)	Prospective	Any fall	Prospective	Monthly for 12 months	761	70+	68.0%	Orthostatic hypotension	9
Chan (103)	1997	Cross sectional	Community (home)	Cross sectional	Any fall	Retrospective	Past 12 months	401	69 (Range 60–90)	48.0%	Orthostatic hypotension; hypertension	6
Chang (36)	2010	Cross sectional	Community (home)	Cross sectional	Injurious falls	Retrospective	Past 12 months	1,361	72.0 (SD = 5.0)	40.0%	Orthostatic hypotension; hypertension	7
Chang (35)	2011	Case-control	Hospital	Retrospective	Any fall	Retrospective (reported on patient safety reporting system)	Any time during hospitalisation	330	72.2 (SD = 6.6)	39.4%	Hypertension; heart disease; stroke	7
Chang (104)	2015	Cross sectional	Community	Cross sectional	Any fall	Retrospective (self reported)	Previous 12 months	14,881	65+	Not reported	Hypertension; heart disease; stroke	6

First author	Year	Design	Setting	Data gathering	Falls outcome	Falls assessment method	Falls reporting interval	N	Age, Mean (SD)/ Median (IQR range)*	% female	Exposure(s)	NOS Score
Chen (38)	2008	Cross sectional	Residential care	Cross sectional	Any fall	Retrospective (self reported)	Previous 6 months	585	80.9 (SD = 5.4)	0.0%	Hypertension; cardiovascular disease; stroke	8
Chen (37)	2010	Case-control	Hospital	Retrospective	Any fall	Retrospective (reported on incident information management system)	Any time during hospitalisation	408	80.0 (SD = 10.1)	47.5%	Ischaemic heart disease; hypertension; congestive heart failure; atrial fibrillation; stroke	7
Choi (93)	2014	Cross sectional	Community	Cross sectional	Any fall	Retrospective (self reported)	Previous 12 months	43,367	Fallers: 72.8 (SD = 5.8); Non-fallers: 73.2 (SD = 5.8)	54.8%	Hypertension; stroke; coronary artery disease	7
Chu (39)	2007	Cohort	Community	Prospective	Any fall	Prospective (self reported)	12 months	1,517	73.0 (SD = 6.0)	49.0%	Stroke; orthostatic hypotension; hypertension; coronary heart disease	7
Claffey (112)	2022	Cross sectional	Community	Cross sectional	Any fall	Retrospective (self reported)	2 years	934	75.5	51.0%	Orthostatic hypotension; heart disease; stroke	8
Coutaz (143)	2012	Cohort	Hospital	Prospective	Any fall	Retrospective (self reported)	Previous 6 months	340	80.7 (8.2)	68.5%	Orthostatic hypotension	7
Coutinho (158)	2008	Case-control	Hospital	Retrospective	Injurious falls	Retrospective	Directly prior to hospitalisation	500	Fallers: 75.5 (SD = 8.2); non-fallers: 75.3 (SD = 7.7)	22.0%	Low blood pressure; stroke	5
Dahodwala (205)	2017	Cohort	Community (Primary Care)	Prospective	Any fall	Prospective (self reported)	6 months	1,100	68.2 (SD = 8.8)	56.6%	Stroke	7
Damian (40)	2013	Cross sectional	Community (residential facility)	Cross sectional	Any fall	Incident reports	1 month	733	83	76.0%	Hypertension; cardiac arrhythmia; coronary artery disease; heart failure	4
Davies (111)	2001	Case-control	Emergency department	Cross sectional	Unexplained falls	Retrospective	Falls leading to hospital admission	26 / 54	79.0 (SD = 7.0), 78.0 (SD = 7.0)	80% / 80%	Orthostatic hypotension; Carotid sinus hypersensitivity	6
Davison (184)	2005	Case-control	Emergency department	Cross sectional	Reoccurring falls	Retrospective	24 hours (during ECG recording)	128 / 100	77.0 (SD = 6.0), 75.0 (SD = 6.0)	67%, 59%	Cardiac arrhythmia	7

First author	Year	Design	Setting	Data gathering	Falls outcome	Falls assessment method	Falls reporting interval	N	Age, Mean (SD)/ Median (IQR range)*	% female	Exposure(s)	NOS Score
Del Brutto (41)	2019	Cross sectional	Community	Cross sectional	Any fall	Retrospective (self reported)	Previous 12 months	327	70.4 (SD = 7.9)	57.0%	Hypertension; stroke	8
Dokuzlar (42)	2020	Cross sectional	Hospital outpatient department	Cross sectional	Any fall	Retrospective (self reported)	Previous 12 months	334	75.0 (SD = 7.3)	0.0%	Hypertension; orthostatic hypotension	7
Dolinis (201)	1997	Cross sectional	Community	Cross sectional	Any fall	Retrospective (self reported)	Previous 12 months	1,947	70+	47.0%	Stroke / transient ischemic attack	8
Donoghue (113)	2021	Cohort	Community	Prospective	Reoccurring falls; injurious falls; unexplained falls; and syncope	Prospective (self reported)	5 years	1,500	71.1 (SD = 5.2)	51.0%	Orthostatic hypotension; sustained orthostatic hypotension; Orthostatic hypotension (40)	8
dos Reis (192)	2015	Cohort	Residential care	Prospective	Any fall	Prospective (report book completed by care team)	Six months	271	79.1 (SD = 9.7), 60-111	57.5%	Stroke	8
Downton (43)	1991	Cross sectional	Community (home)	Cross sectional	Any fall	Retrospective	Past 12 months	203	83.0 (SD = 5.0), range 75–97	70.0%	Orthostatic hypotension; hypertension; coronary artery disease; stroke	6
Ek (206)	2019	Cohort	Community	Prospective	Injurious falls	Prospective (national patient database - clinical examination)	4 years, and 48-120 months.	3,112	Females: 75.2 (SD = 11); Males: 71.5 (SD =9.9)	63.7%	Stroke; heart disease (Arrhythmia, bradycardia and conduction disease, atrial fibrillation, ischemic heart disease, heart failure)	8
Ensrud (137)	1992	Cross sectional	Community (home)	Cross sectional	Any fall	Retrospective	12 months	9,704	72 (Range 65–99)	100.0%	Orthostatic hypotension	7
Finucane (114)	2017	Cohort	Community	Prospective	Falls, unexplained falls, injurious falls, syncope, multiple falls	Prospective (self reported)	24 months	4,127	61.5 (SD = 8.2); 50+	54.2%	Orthostatic hypotension; initial orthostatic hypotension; impaired orthostatic BP stabilization	8

First author	Year	Design	Setting	Data gathering	Falls outcome	Falls assessment method	Falls reporting interval	N	Age, Mean (SD)/ Median (IQR range)*	% female	Exposure(s)	NOS Score
Frankenthal (172)	2021	Cohort	Community	Cross sectional	Any fall	Retrospective (self reported)	Previous 12 months	3,159	75.1 (SD = 6.2)	57.3%	Myocardial infarction; arrhythmia/chronic heart failure; stroke	7
Frels (155)	2002	Case-control	Hospital	Prospective	Any fall	Prospective (incident book)	During hospitalisation (mean length of stay not reported)	362	Fallers: 73.3 (13); Controls: 73.7 (13)	Fallers: 52%; Controls: 48%	Stroke; orthostatic hypotension	7
Freud (124)	2015	Cross sectional	Outpatient hospital unit	Cross sectional	Any fall	Retrospective (self reported)	Previous 12 months	571	83.7 (SD = 6.1)	64.1%	Orthostatic hypotension	8
Friedman (193)	2002	Cohort	Community	Prospective	Any fall	Prospective (self reported)	Previous 12 months, and 12 months prospective	2,212	Median: 72.6 (Range: 65.9-86.3)	58.6%	Stroke	8
Gamage (94)	2019	Cross sectional	Community	Cross sectional	Any fall, reoccurring falls	Retrospective (self reported)	Previous 12 months	300	73.0 (SD = 6.7)	58.3%	Hypertension; general cardiovascular disease; coronary artery disease	8
Gangavati (138)	2011	Cohort	Community (home)	Prospective	Reoccurring falls	Prospective	Monthly, range 183–365 days	722	78.0 (SD = 5)	64.0%	Orthostatic hypotension	9
Gebre (213)	2021	Cohort	Community	Prospective	Injurious falls resulting in hospitalisation	Prospective (self reported)	15 years, 11 months	1,053	75.0 (SD = 2.6)	100.0%	Abdominal aortic calcification	8
Gebre (163)	2022	Cohort	Community	Prospective	Injurious falls	Prospective (self reported)	10.8 years (SD = 4.2 years)	1,431	75.2 (SD = 2.7)	100.0%	Cardiovascular disease; ischaemic heart disease; cerebrovascular disease	7
Geng (202)	2017	Cross sectional	Community	Cross sectional	Any fall	Retrospective (self reported)	Previous 12 months	6,277	74.4 (Range: 65-90)	100.0%	Stroke	4
George (165)	2014	Cross sectional	Community	Cross sectional	Any fall	Retrospective (self reported)	Previous three months	1,653	65+	57.2%	Coronary heart disease; stroke	6
Goh (44)	2017	Cohort	Community	Prospective	Any fall	Retrospective (self reported)	Previous 12 months	1,218	55+	56.6%	Myocardial infarction; angina; hypertension	8
Graafmans (147)	1996	Cohort	Community (home and residential facility)	Prospective	Any fall, and reoccurring falls	Prospective	Weekly, returned 2-monthly for 28 weeks	354	70+	84.0%	Orthostatic hypotension; stroke	9

First author	Year	Design	Setting	Data gathering	Falls outcome	Falls assessment method	Falls reporting interval	N	Age, Mean (SD)/ Median (IQR range)*	% female	Exposure(s)	NOS Score
Granek (81)	1987	Case-control	Residential care	Prospective	Any fall	Prospective (incident reports)	10 months prospectively	384	Median: Fallers: 83; Controls: 81	Not reported	Arrhythmias; heart failure; cardiovascular disease; hypertension; cerebrovascular incidents	4
Grundstrom (194)	2012	Cross sectional	Community	Cross sectional	Any fall	Retrospective (self reported)	Previous 3 months	12,684	85+	71.2%	Stroke	7
Ha (95)	2021	Cross sectional	Hospital (outpatients)	Cross sectional	Any fall (with no external cause e.g., violence)	Retrospective (self reported)	Previous 12 months	539	69.4 (SD = 7.5)	49.7%	Hypertension; peripheral vascular disease; stroke;	8
Ham (45)	2017	Cohort	Community	Prospective	Injurious falls	Prospective (self reported)	Median follow up of 137 months	7,662	55+	60.4%	Hypertension, hypotension	9
Hanlon (208)	2002	Cohort	Community	Prospective	Any fall, reoccurring falls	Retrospective (self reported)	Previous 12 months	2,996	72.3 (5.8)	64.0%	Stroke	7
Hartog (125)	2015	Cohort	Residential care (nursing home)	Prospective	Any fall	Retrospective	Previous 12 months	128	80.7 (SD = 7.9)	71.9%	Hypotension; hypertension; cardiovascular disease	7
Hartog (46)	2017	Cohort	Community	Cross sectional	Any fall	Retrospective (self reported)	Previous 12 months	1,407	78 (SD = 7.0), 65+	63.0%	Orthostatic hypotension	8
Heckenbach (47)	2014	Cross sectional	Community (home)	Cross sectional	Any fall	Retrospective (Medical records, ICD-10 codes)	Within one month or at least one month from the prescription of fall risk increasing drugs	5,124	73	65.0%	Hypertension; cardiovascular disease; heart failure	6
Heitterachi (115)	2002	Cohort	Community (home)	Prospective	Any fall	Prospective	Monthly for 12 months	70	77.0 (SD = 6.0)	80.0%	Orthostatic hypotension	9
Herndon (108)	1997	Case-control	Community (home)	Cross sectional	Injurious falls	In ER or admitted for falls	Past 7 days	467 / 691	65+	Not reported	Hypertension; coronary artery disease	5
Himes (195)	2012	Cohort	Community	Retrospective	Any fall	Retrospective (self reported)	Previous 24 months	10,755	65+	Not reported	Stroke	7

First author	Year	Design	Setting	Data gathering	Falls outcome	Falls assessment method	Falls reporting interval	N	Age, Mean (SD)/ Median (IQR range)*	% female	Exposure(s)	NOS Score
Ho (196)	1996	Cross sectional	Community	Cross sectional	Any fall	Retrospective (self reported)	Previous 12 months	2,032	70+	50.9%	Stroke / transient ischemic attack	8
Homer (183)	2021	Cohort	Community	Prospective	Insurance claims related to falls	Prospectively (insurance claims)	24 months prospectively	120,881	75 (Interquartile range: 12)	60.0%	Cerebrovascular Disease; Cardiac Dysrhythmias	6
Hosseini (212)	2020	Cross sectional	Community	Cross sectional	Any fall	Retrospective (self reported)	Previous 12 months	1,616	69.4 (SD = 7.4), 60+	45.3%	Heart disease	8
Hung (48)	2013	Cross sectional	Acute hospital	Cross sectional	Any fall	Retrospective	Past 3 years	401	82.0 (SD = 0.2)	24.0%	Cardiac arrhythmia; hypertension	5
Hung (173)	2017	Cross sectional	Residential care (veterans homes)	Cross sectional	Single fall, reoccurring falls	Retrosepctive (self reported)	Previous 12 months	871	85.5 (SD = 5.2)	0.0%	Coronary artery disease; stroke	6
Hussain (177)	2022	Cohort	Community	Prospective	Any fall	Retrospective (self reported)	Mean follow-up: 49 months	16,703	75.3 (SD = 4.4)	55.0%	Cardiovascular disease; heart failure	8
Jacob (82)	2022	Cohort	Community	Prospective	Any fall	Retrospective (self reported)	2 years	6,900	63.1 (SD = 8.9)	51.6%	Heart disease; hypertension; stroke	7
Jansen (a) (49)	2015	Cross sectional	Community (home)	Cross sectional	Any fall, and reoccurring falls	Retrospective	Past 12 months	8,173	64.0 (SD = 10.0	54.0%	Heart failure; hypertension, coronary artery disease	6
Jansen (b) (178)	2015	Cross sectional	Community (home)	Cross sectional	Any fall	Retrospective	Past 12 months	4,886	62.0 (SD = 8.0)	54.0%	Cardiac arrhythmia	6
Jia (197)	2019	Cohort	Community	Prospective	Any fall	Retrospective (self reported)	Previous 12 months	164,597	75.1	57.8%	Stroke	6
Jitapunkul (96)	1998	Cross sectional	Community (home)	Cross sectional	Any fall	Retrospective	Past 6 months	4,480	69.0 (SD = 8.0)	60.0%	Hypertension	6
Jodaitis (116)	2015	Case-control	Hospital	Cross sectional	Reoccurring falls	Retrospective (self reported)	Previous 6 months	285	85.0 (SD = 5.0)	54.0%	Orthostatic hypotension	5
Jonsson (83)	1990	Case-control	Residential care	Prospective	Reoccurring falls	Retrospective (medical records or incident reports)	Previous 6 months	58	Fallers: 87 (6); Controls: 85 (5)	Fallers: 71%; Controls: 55%	Ischemic heart disease; hypertension; congestive heart failure; cerebrovascular disease	7
Jorgensen (176)	2015	Case-control	Hospital	Retrospective	Injurious falls	Retrospective (incident reports)	During hospitalisation	28,524	65+	64.2%	Stroke; heart failure; ischemic heart disease	6

First author	Year	Design	Setting	Data gathering	Falls outcome	Falls assessment method	Falls reporting interval	N	Age, Mean (SD)/ Median (IQR range)*	% female	Exposure(s)	NOS Score
Juraschek (50)	2019	Cohort	Community	Prospective	Falls related to hospitalisation or claim	Retrospective (Database surveillance)	Median follow-up of 54 months	3,973	75.7 (SD = 5.0)	62.0%	Hypertension	8
Juraschek (131)	2022	Cohort	Community	Prospective	Any fall	Prospective (self reported)	1 month	534	76.0 (SD = 5.0)	42.0%	Orthostatic hypotension	9
Just (51)	2021	Cohort	Community	Prospective	Any fall, multiple falls	Prospective and retrospective (self reported)	Previous 12 months and 12 months prospectively	1,377	74.1	42.9%	Hypertension; heart failure; stroke	8
Kallin (166)	2004	Cohort	Residential care	Prospective	Any fall	Prospective (post fall evaluations)	12 months	199	82.4 (SD = 6.8)	70.4%	Heart disease; stroke	6
Kamali (84)	2022	Cross sectional	Community	Cross sectional (and prospective)	Any fall	Retrospective, prospective (self reported)	12 months, and 6 months follow up	750	67.7 (SD = 6.7) (no HTN) - 68.0 (SD = 6.7) (HTN no meds) - 67.7 (SD = 6.4) HTN with meds	50.0%	Hypertension; orthostatic hypotension	7
Kang (52)	2018	Cohort	Community	Prospective	Any fall	Retrospective (self reported)	Previous 12 months	619	67.4	57.7%	Hypertension; heart disease; stroke	7
Kao (53)	2012	Cross sectional	Community (home)	Cross sectional	Reoccurring and injurious falls	Retrospective	Past 12 months	360	76 (Range 64–91)	61.0%	Hypertension; cardiovascular disease	6
Kario (109)	2001	Cohort	Community (home)	Prospective	Any fall	Prospective and retrospective	Monthly for 12 months	266	75.5 (SD = 5.0)	54.0%	Orthostatic hypotension; hypertension; myocardial infarction; heart disease	9
Kelly (54)	2003	Case-control	Hospital (emergency departments)	Retrospective	Injurious falls	Retrospective (self reported)	Previous 12 months	2,278	Fallers: 78.5 (SD = 7.7); non-fallers: 74.5 (SD = 6.7)	Fallers: 69.0%; non-fallers: 57.0%	Hypertension; cardiovascular disease; stroke	6
Klein (110)	2013	Cohort	Community (home)	Cross sectional	Any fall	Retrospective	Past 3 months	3,544	70 (Range = 60–97)	56.0%	Hypertension	5
Koca (174)	2020	Case-control	Hospital outpatient department	Prospective	Any fall	Retrospective (Self reported history of falls)	Previous 12 months	123	73.7 (SD = 5.9)	61.8%	Atrial fibrillation; coronary artery disease	8

First author	Year	Design	Setting	Data gathering	Falls outcome	Falls assessment method	Falls reporting interval	N	Age, Mean (SD)/ Median (IQR range)*	% female	Exposure(s)	NOS Score
Kocyigit (55)	2020	Case-control	Hospital	Prospective	Any fall	Retrospective (self reported)	Previous 12 months	741	75 (SD = 8.0)	65.0%	Orthostatic hypotension; hypertension	7
Kocyigit (117)	2021	Cohort	Hospital (outpatient geriatric department)	Prospective	Any fall	Retrospective and prospective (self reported)	Previous 12 months, and six months prospectively	692	75.0 (SD = 7.7)	64.8%	Orthostatic hypotension	7
Kojima (56)	2011	Cross sectional	Outpatient clinic	Cross sectional	Any fall	Retrospective (self reported)	Previous 12 months	262	76.2 (SD = 6.8)	67.9%	Hypertension; stroke	7
Ku (57)	2013	Cross sectional	Residential care	Cross sectional	Any fall	Prospective (resident records)	Previous 12 months	940	85.5 (SD = 5.7); range = 65-110	0.0%	Hypertension; coronary artery disease; stroke	9
Kumar (188)	2003	Case-control	Falls & syncope clinic	Retrospective	Unexplained falls	Referred for falls	Referred to hospital after a fall	265 / 44	79.5 (Range = 60–92) / 71.3 (Range = 63–86)	76%, 36%	Carotid sinus hypersensitivity	7
Lam (203)	2019	Cohort	Hospital	Prospective	Any fall, injurious falls	Prospective (self reported)	Six months prospectively	267	78.3 (SD = 7.6)	56.9%	Stroke	7
Lawlor (58)	2003	Cross sectional	Community (home)	Cross sectional	Any fall	Retrospective	Past 12 months	4,050	71	100.0%	Orthostatic hypotension; coronary artery disease; hypertension	4
Le Couteur (161)	2003	Cross sectional	Community (residential facility)	Cross sectional	Any fall	Incident reports	Past 12 months	179	83.0 (SD = 7.0)	80.0%	Postprandial hypotension	6
Lee (214)	2006	Cross sectional	Community (home)	Cross sectional	Any fall, reoccurring falls	Retrospective	Past 12 months	4,000	73.0 (SD = 5.0)	50.0%	Cardiovascular disease	5
Lee (175)	2009	Cross sectional	Community (home)	Cross sectional	Reoccurring falls	Retrospective	Past 12 months	11,113	55% 65–75 years, 45%76+	58.0%	Coronary artery disease	4
Lee (59)	2020	Cohort	Community	Retrospective	Any fall	Retrosepctive (Self reported history of falls)	Previous 24 months	17,712	70.4 (SD = 6.6)	56.9%	Heart failure; hypertension; stroke/transient ischemic attack	8
Lee (90)	2021	Cohort	Community	Prospective	Any fall	Prospectively (self reported)	12 months prospectively	232	70.5 (SD = 9.2)	74.0%	Hypertension; stroke	7
Liao (105)	2012	Cross sectional	Community (home)	Cross sectional	Any fall	Retrospective	Past 12 months	1,165	75.0 (SD = 7.0)	54.0%	Hypertension	5

First author	Year	Design	Setting	Data gathering	Falls outcome	Falls assessment method	Falls reporting interval	N	Age, Mean (SD)/ Median (IQR range)*	% female	Exposure(s)	NOS Score
Lipsitz (60)	1991	Case-control	Residential care	Prospective	Any fall	Prospective (computer records and review of incidence reports)	6 months	126	87.0 (SD = 6.0)	69.3%	Ischemic heart disease; hypertension; congestive heart failure; stroke / transient ischemic attack; atrial fibrillation or flutter; orthostatic hypotension	8
Liu (148)	1995	Cohort	Community (residential facility)	Prospective	Any fall	Prospective	Weekly for 12 months	100	83.0 (SD = 6.0)	83.0%	Orthostatic hypotension	9
Lord (204)	2003	Cohort	Residential care	Prospective	Any fall, injurious falls	Prospective (incident reports and medical records)	Mean: 15.3 (SD = 7.5) months; range 6-29 months	1,000	85.0 (SD = 7.4); Range = 65-103	7440.0%	Stroke	7
Lukaszyk (198)	2018	Cross sectional	Community	Cross sectional	Any fall	Retrospective (self reported)	Previous 12 months	336	67.0 (SD = 6.3)	59.0%	Stroke	7
Luukinen (149)	1996	Cohort	Community (home)	Prospective	Reoccurring falls	Prospective	3-montly during 12 months	1,016 / 650	76.0 (SD = 5)	63.0%	Orthostatic hypotension	9
Mader (139)	1987	Cross sectional	Outpatient clinic, community clinic	Cross sectional	Any fall	Retrospective	Past 12 months	300	70 (Range 56–93)	77.0%	Orthostatic hypotension	5
Magnuszewski (61)	2020	Cross sectional	Hospital (geriatric ward)	Cross sectional	Any fall	Retrospective (self reported)	Previous 12 months	358	Median: 82 (IQR = 76-86)	77.9%	Hypertension; Ischemic heart disease; myocardial infarction; atrial fibrillation; heart failure; peripheral arterial disease; stroke / transient ischemic attack; orthostatic hypotension	8

First author	Year	Design	Setting	Data gathering	Falls outcome	Falls assessment method	Falls reporting interval	N	Age, Mean (SD)/ Median (IQR range)*	% female	Exposure(s)	NOS Score
Magnuszewski (85)	2022	Cross sectional	Hospital (geriatric ward)	Prospective	Any fall	Prospective (incident reports)	8 months	416	Median: 82 (IQR= 77 – 86)	77.4%	Hypertension; Orthostatic hypotension; Ischemic heart disease; chronic cardiac failure; atrial fibrillation; peripheral arterial disease; stroke	
Margolis (97)	2019	Cohort	Community	Prospective	Any fall	Prospective (self reported)	12 months	5,971	78.8 (SD = 6.7), 50+	100.0%	Hypertension	8
Maurer (118)	2004	Cohort	Long-term care	Prospective	Any fall	Incident reports	Weekly during 270-day FU (range 8–657)	111	88.0 (SD = 7)	82.0%	Orthostatic hypotension	8
Maurer (98)	2005	Cohort	Long-term care	Prospective	Any fall	Incident reports	12 months	139	88.0 (SD = 7)	85.0%	Hypertension	8
McDonald (119)	2017	Cohort	Community	Prospective	Any fall	Prospective (self reported)	12 months	297	65+	51.0%	Orthostatic hypotension	9
Menant (126)	2016	Cohort	Community	Prospective	Unexplained falls	Prospective	12 months	523	Non fallers: 79.6 (SD = 4.4); Unexplained fallers: 79.6 (SD = 4.7); Balance related fallers: 80 (SD = 4.4)	52.2%	Hypotension; heart disease; myocardial infarction; stroke	10
Mitchell (63)	2013	Cross sectional	Community (home)	Cross sectional	Any fall	Retrospective	12 months	5,681	65+	55.0%	Coronary artery disease, hypertension	6
Mitchell (62)	2015	Cross sectional	Community	Cross sectional	Any fall	Retrospective (self reported)	Previous 12 months	5,681	65+	55.6%	Heart disease; hypertension; stroke	8
Miu (144)	1997	Cross sectional	Community	Cross sectional	Any fall	Retrospective (self reported)	Previous 12 months	400	73.1 (SD = 6.0)	58.8%	Orthostatic hypotension	7
Mol (152)	2022	Cross sectional	Hospital (geriatric ward)	Retrospective	Any fall	Retrospective (self reported)	12 months	635	81.1 (SD = 6.9)	58.9%	Orthostatic hypotension	7
Moloney (230)	2021	Cohort	Community	Cross sectional	Any fall	Retrospective (self reported)	Previous 12 months	4,899	61.0 (SD = 8.8)	55.2%	Orthostatic hypotension	8
Murphy (187)	1986	Cohort	Long-term care	Prospective	Any fall	Incident reports	2 years, 9 months	100	80, Range = 63–97 (with no CSH)/ 83, Range = 61–97 (with CSH)	86.0%	Carotid sinus hypersensitivity	7

First author	Year	Design	Setting	Data gathering	Falls outcome	Falls assessment method	Falls reporting interval	N	Age, Mean (SD)/ Median (IQR range)*	% female	Exposure(s)	NOS Score
O'Neal (179)	2015	Cross sectional	Community	Cross sectional	Reoccurring falls	Retrospective (self reported)	Previous 12 months	24,117	65.0 (SD = 9.3)	55.0%	Atrial fibrillation	8
Ooi (150)	2000	Cohort	Long-term care	Prospective	Any fall, reoccurring falls	Incident reports	Incident reports during 18 months	844	60+	80.0%	Orthostatic hypotension	8
Ooi (64)	2021	Cohort	Community	Prospective	Any fall	Retrospective and prospective (self reported)	Previous 12 months, and 18 months prospectively	1,763	≥ 65	50.8%	Hypertension; stroke; cardiovascular disease	7
Oren (100)	2022	Case-control	Hospital (geriatric ward)	Retrospective	Any fall	Incident reports	6 years	699	Fallers: 86.9 (SD = 4.4) Controls: 87.0 (SD = 4.9)	58.4%	Hypertension; congestive heart failure;	7
Paganini-Hill (65)	2017	Cohort	Residential care	Retrospective	Any fall	Retrospective (self reported)	Previous 12 months	1,536	94.0 (SD = 4.1)	78.0%	Hypertension; heart disease; transient ischemic attack or Stroke	8
Paliwal (167)	2017	Cross sectional	Residential care	Cross sectional	Any fall	Retrospective (self reported)	Previous 12 months	159,336	65+	56.2%	Myocardial infarction; angina; stroke	8
Pasma (127)	2014	Cross sectional	Outpatient clinic	Cross sectional	Any fall	Retrospective	Past 12 months	197	82	60.0%	Orthostatic hypotension	6
Press (132)	2016	Cross sectional	Community	Cross sectional	Any fall	Retrospective	any fall	571	83.7 (SD=6.1)	64.1%	Orthostatic hypotension	7
Prudham (66)	1981	Cross sectional	Community	Cross sectional	Any fall	Retrospective (self reported)	Previous 12 months	2,793	65+	58.9%	Heart disease; hypertension; stroke	4
Puisieux (160)	2000	Case-control	Acute hospital	Cross sectional	Any fall	In ER or admitted for falls	Falls leading to hospital admission	45 / 36	80.9 (SD = 8.5) / 78.5 (SD = 7.2)	73%, 68%	Postprandial hypotension	8
Rafiq (168)	2014	Cohort	Community (home)	Cross sectional	Any fall	GP visit for fall	2 years, 6 months baseline, 30 months FU	135,433	75 (SD = 8), Range = 65–104	56.0%	Coronary artery disease; heart failure	7
Rivan (86)	2021	Cohort	Community	Prospective	Any fall, injurious falls	retrospective	5 years	400	67.7 (SD = 5.3)	54.9%	Hypertension; Heart disease	7
Rivera-Chavez (67)	2021	Cross sectional	Hospital	Cross sectional	Any fall	Retrospective (self reported)	Unspecified	669	81.0 (SD = 7.5)	66.0%	Stroke; myocardial infarction; atrial fibrillation; heart failure; postural hypotension; systemic arterial hypertension	7

First author	Year	Design	Setting	Data gathering	Falls outcome	Falls assessment method	Falls reporting interval	N	Age, Mean (SD)/ Median (IQR range)*	% female	Exposure(s)	NOS Score
Roca (133)	2022	Cross sectional	Outpatient clinic	Cross sectional	Any fall	Retrospective	Prevous 6 months	530	82.9 (SD = 5.1)	66.0%	Orthostatic hypotension;	6
Romero-Ortuno (121)	2011	Cross sectional	Community (home)	Cross sectional	Any fall	Retrospective	Past 6 months	598	72	72.0%	Orthostatic hypotension	6
Rosado (182)	1989	Case-control	Long-term care, Community (residential facility)	Cross sectional	Any fall	Incident reports	Past 7 days	51 / 27	86	Not reported	Cardiac arrhythmia	8
Rosendahl (207)	2003	Cohort	Residential care	Prospective	Any fall	Prospective (incident reports)	12 months	78	81.0 (SD = 6.0)	72.0%	Stroke	5
Rutan (140)	1992	Cross sectional	Community (home)	Cross sectional	Any fall	Retrospective	12 months	4,931	OH+: 73.6, OH-: 72.6	56.0%	Orthostatic hypotension	6
Saedon (68)	2016	Case-control	Hospital	Cross sectional	Multiple falls or injurious falls	Retrospective (self reported)	Previous 12 months	262	Fallers: 75.0 (SD = 7.0); Non-fallers: 72.0 (SD = 6.0), 65+	70.2%	Hypertension; myocardial infarction; heart failure; atrial fibrillation; orthostatic hypotension	6
Sagawa (156)	2018	Cohort	Community	Prospective	Injurious falls	Prospective	12 months	1,819	76.6 (SD = 2.9)	53.0%	Cardiovascular disease; low blood pressure	8
Salgado (199)	1994	Case-control	Hospital	Cross sectional	Any fall	Retrospective (incident reports)	Falls during hospital stay	88	Fallers: 80.7 (SD = 8.2); non-fallers: 80.0 (SD = 6.8)	47.7%	Stroke	7
Salgado (200)	2004	Cohort	Hospital	Prospective	Any fall	Prospective	During hospitalisation	88	85.9 (SD = 4.2)	59.1%	Stroke	6
Sanders (180)	2012	Case-control	Emergency department	Retrospective	Unexplained falls and accidental falls	Retrospective	Falls leading to hospital admission	211 / 231	82 (SD = 9) / 79 (SD = 7)	62.0%	Cardiac arrhythmia	5
Sasidharan (134)	2022	Cross sectional	Community	Cross sectional	Any fall	Retrospective (self reported)	12 months	240	68.9 (SD = 7.1)	59.2%	Orthostatic hypotension	7
Schell (128)	2021	Cohort	Hospital	Retrospective	Any fall	Retrospective (self reported)	During hospital stay (no time period reported)	7,745	65+	61.6%	Orthostatic hypotension	7

First author	Year	Design	Setting	Data gathering	Falls outcome	Falls assessment method	Falls reporting interval	N	Age, Mean (SD)/ Median (IQR range)*	% female	Exposure(s)	NOS Score
Schoon (162)	2013	Case-control	Falls & syncope clinic	Cross sectional	Any fall and syncope	Referred for falls	Referred to hospital after a fall	105 / 25	79.0 (SD = 7.0) / 74.0 (SD = 4.0)	67%, 20%	Head turning induced hypotension; postprandial hypotension	8
Sharma (69)	2017	Cohort	Community	Retrospective	Any fall	Retrospective (self reported)	Previous 12 months	561	67.5 (SD = 6.4)	50.1%	Stroke; hypertension; cardiovascular disease	7
Shaw (129)	2015	Cross sectional	Residential care	Cross sectional	Any fall	Retrospective (self reported)	Previous 12 months	59	Fallers: 83.0 (SD = 6.8); Non-fallers: 83.4 (SD = 9.1)	54.3%	Heart disease; heart failure; stroke; hypotension	5
Sibley (99)	2014	Cross sectional	Community (home)	Cross sectional	Any fall	Retrospective	12 months	16,357	65+	55.0%	Hypertension; coronary artery disease	6
Song (70)	2021	Cohort	Community	Cross sectional	Any fall, injurious falls	Retrospective (self reported)	Previous 12 months	6,595	91.0 (SD = 7.5)	56.1%	Hypertension; low blood pressure	8
Sorock (209)	1983	case-control	Hospital rehab hospital	retrospective	Any fall	Retrospective medical records	length of admission	512	case 74.5 control 73.8	cases 52.6%- control 67.3%	stroke, heart disease, peripheral vascular disease	6
Soysal (135)	2016	Cross sectional	community	prospective	Reoccurring falls	Retrospective (self reported)	Previous 12 months	407	Without OH 74.1 (SD = 8.6) - age with OH 78.7 (SD=7.8)	62.9%	Orthostatic hypotension	7
Stenhagen (169)	2013	Cohort	Community (home)	Prospective	Any fall	Retrospective	Past 6 months, at 3 and 6 years	1,763	60–93	54.0%	Heart failure; coronary artery disease	7
Subramanian (71)	2020	Cross sectional	Outpatient	Cross sectional	Any fall	Retrospective	Not reported	160	74.5 (SD = 8.9)	26.3%	Hypertension; coronary artery disease; stroke / transient ischemic attack; orthostatic hypotension	5
Susman (153)	1989	Cross sectional	Community	prospective	Any fall	Retrospective (self reported)	Previous 12 months	100	73	62.0%	Orthostatic hypotension	7

First author	Year	Design	Setting	Data gathering	Falls outcome	Falls assessment method	Falls reporting interval	N	Age, Mean (SD)/ Median (IQR range)*	% female	Exposure(s)	NOS Score
Svensson (87)	1992	Cross sectional	community	retrospective	Any fall	Retrospective (self reported)	fall in the last 12 months	732	84-85	66.7%	Hypertension, coronary artery disease angina, myocardial infarction, transient ischaemic attack	7
Swanenburg (157)	2010	Cohort	Community	Prospective	Multiple falls	Retrospective and prospective	Previous 12 months, and 12 months prospectively	270	73.7 (SD = 7)	83.0%	Low blood pressure	8
Teoh (106)	2021	Cohort	Community	Cross sectional	Any fall	Retrospective (self reported)	Previous 12 months	1,415	68.6 (SD = 7.3)	57.2%	Hypertension; myocardial infarction; stroke	8
Thapa (88)	2022	Cross sectional	Residential care	Cross sectional	Any fall	Electronic health records (Medical records)	approximately 14 years	1,749	60+	66.0%	Hypertension; chronic heart failure; myocardial infarction; arrhythmia; stroke	6
Tinetti (151)	1986	Cohort	Long-term care	Prospective	Reoccurring falls	Incident reports	3 months	79	81.0 (SD = 7.0), 78.0 (SD = 7.0)	78%, 62%	Orthostatic hypotension	5
Tsai (89)	2021	Cross sectional	community	Retrospective	Any fall	Retrospective (self reported)	Fall in last month	6,153	≥ 65	53.2%	Myocardial infarction, heart disease, hypertension, stroke	6
Turusheva (72)	2020	Cross sectional	Community	Cross sectional	Any falls, injurious falls	Retrospective (self reported)	Previous 12 months	163	70.9 (SD = 6.5)	40.0%	Arterial stiffness; hypertension; hypotension; myocardial infarction; stroke; peripheral arterial disease	8
Valderrama-Hinds (73)	2018	Cohort	Community	Prospective	Any fall	Retrospective (self reported)	Previous 24 months	6,247	69.6	51.8%	Hypertension; myocardial infarction; stroke	6
van der Velde (a) (122)	2007	Cross sectional	Outpatient clinic	Cross sectional	Any fall	Retrospective	12 months	217	Fallers 79.0 (SD = 6), non fallers 75.0 (SD =6)	66.0%	Orthostatic hypotension	6
van der Velde (b) (185)	2007	Cohort	Outpatient clinic	Prospective	Any fall	Prospective	Monthly during 3 month FU	215	77.4 (SD = 6.0)	65.0%	Heart valve abnormalities	9

First author	Year	Design	Setting	Data gathering	Falls outcome	Falls assessment method	Falls reporting interval	N	Age, Mean (SD)/ Median (IQR range)*	% female	Exposure(s)	NOS Score
Vieira (74)	2018	Cross sectional	Community	Cross sectional	Any fall	Retrospective (self reported)	Previous 12 months	1,448	60+	63.0%	Hypertension; heart problems	8
von Heideken Wågert (164)	2009	Cohort	community housing (49%) and institutional housing 51%(NH, dementia communities residential care)	Cross sectional	Any fall	Prospective - diaries, phone calls, charts	6 months	220	90.3 (SD = 4.8)	76.0%	Coronary artery disease, Stroke	10
Wallace (181)	2017	Cohort	Community	Prospective	Any fall	Retrospective (self reported)	Mean follow-up of 106 months	4,462	65+	59.4%	Atrial fibrillation	8
Welmer (141)	2020	Cohort	Community	Prospective	Injurious falls	Retrospective (self reported)	3 years, and 10 years	1,624	67.7 (SD = 7.4)	57.8%	Orthostatic hypotension	8
Wong (75)	2014	Cohort	Community (home)	Prospective	Any fall	Prospective	Monthly for 12 months	481	80.0 (SD = 4.0)	51.0%	Arterial stiffness, orthostatic hypotension; hypertension; coronary artery disease	9
Xu (76)	2015	Cohort	Military communities	Prospective	Any fall	Prospective	12 months	447	82.2 (SD = 4.7)	7.7%	Stroke; coronary disease; sinus bradycardia; atrial fibrillation; atrioventricular block; chronic heart failure; hypertension	10
Yasumura (77)	1994	Cross sectional	Community	Cross sectional	Any fall	Retrospective (self reported)	Previous 12 months	807	Men: 71.7; Women: 72.4	54.6%	Hypertension; heart disease; stroke	7
Yi (170)	2021	Cross sectional	Community	Cross sectional	Any fall	Retrospective (self reported)	Previous 12 months	113,805	63.8 (SD = 9.3)	53.5%	Stroke; myocardial infarction	8
Yoo (78)	2016	Cross sectional	Community	Cross sectional	Any fall	Retrospective	Previous 12 months	534	65+	83.5%	Hypertension; stroke	6
Yu (101)	2009	Cross sectional	Community	Retrospective	Any fall	Retrospective self (report and medical records)	12 months	1,512	70.6 (SD= 6.5)	59.1%	Stroke, orthostatic hypotension, hypertension	7

First author	Year	Design	Setting	Data gathering	Falls outcome	Falls assessment method	Falls reporting interval	N	Age, Mean (SD)/ Median (IQR range)*	% female	Exposure(s)	NOS Score
Zhao (107)	2019	Cross sectional	Community	Cross sectional	Any fall	Retrospective (self reported)	Previous month	1,356	≥ 65	68.4%	Hypertension; heart disease; stroke	7
Zhao (130)	2020	Case-control	Hospital	Retrospective	Injurious falls	Retrospective (self reported)	Previous 12 months	345	≥ 65	45.5%	Orthostatic hypotension	7
Zhu (136)	2016	Cross sectional	community	Prospective	Any fall	Retrospective (self reported)	Unspecified	364	74.6 (64.1–98.0)	50.5%	Orthostatic hypotension	7
Zia (79)	2015	Case-control	Outpatient	Cross sectional	Reoccurring or injurious falls	Retrospective (self reported)	Previous 12 months	358	Fallers: 75.2 (SD = 7.1); Non-fallers: 72.2 (SD = 5.5)	67.6%	Hypotension; hypertension	9

* Information on age varied across articles (e.g., sometimes only mean is provided, or lower age limit, or age is specified separately for sex or study groups)

eTable 1B. Selected characteristics of all three included interventional studies

First author, Year	Design	Falls outcome	Falls reporting interval	N	Age, years (Mean (SD), range)	% female	Condition	Intervention	Control	Efficacy	QA assessment (overall risk of bias)
Kenny 2001 (189)	Interventional (randomised, controlled trial)	Non-accidental falls; any syncopal event	12 months	175	Intervention: 72 (SD = 10); Control: 74 (SD = 10)	Intervention: 57%; Control: 61%	Non-accidental fallers with cardioinhibitory carotid sinus hypersensitivity	Dual chamber pacemaker implantation	Standard care (no pacing)	Paced patients were significantly less likely to fall OR= 0.4 (0.2-0.8), p=<0.05, than controls. There was no significant difference between the proportion of paced patients who reported syncope (11%) and the proportion of controls (22%), p>0.05	Some concerns
Parry 2009 (190)	Interventional (randomised, double-blind, placebo, controlled crossover trial)	Unexplained falls	Six months intervention, six months control	34	76.8 (SD = 9)	79%	reoccurring unexplained fallers with carotid sinus hypersensitivity	Double chamber pacemaker implantation (Turned on)	Double chamber pacemaker implantation (Turned off)	The pacing intervention had no significant effect on the number of falls: mean of 4.0 (9.5) falls while pacemaker turned on, 3.5 (7.2) falls while pacemaker turned off, p=>0.05. Relative risk of falling while pacemaker turned off was RR= 0.82 (0.6-1.1), p=>0.05.	Some concerns
Ryan 2010 (191)	Interventional (multicentre, randomised, controlled trial)	Any fall	24 month mean follow up	141	Intervention: 78.0 (SD = 7.3); Control: 77.5 (SD = 8.3)	Intervention: 67%; Control: 56%	Cardioinhibitory carotid sinus hypersensitivity	Dual-chamber pacemaker (k 700 or k 400 system) implantation	Implantable loop recorder	The overall relative risk of falling after device implantation compared with before was RR= 0.2 (0.1-0.3) (p=<0.05). No significant reduction in falls was seen between paced and loop recorder groups RR= 0.78 (0.4- 1.5).	Some concerns

eTable 1C. Cardiovascular disorders and falls

Cardiovascular disorder (*n* of studies)	Study design	Setting (n)	Sample size - Median (Range)	Associations with falls (observational studies)*			Main findings
				n of positive associations (%)<br><br>QA (range)	n of negative associations (if any) (%)<br><br>QA (range)	n of studies with no association (%)<br><br>QA (range)	
				**Blood pressure**			
Hypertension (n=80)	41 Cross sectional; 12 Case-control; 27 Cohort	Community (55); Hospital (16); Long term/residential care facility (9); Clinical sites (2)	733 (57 - 43,367)	17 positive (11 m, 6 u) (21.3%)<br><br>QA: 6.9 (5-8)	3 (3.6%)<br><br>QA: 6.3 (5-9)	60 (75%)<br><br>QA: 7.0 (4-10)	The majority of studies (75%) show no significant association between hypertension and falls. Of note is that the 3 largest studies demonstrate inconsistent associations. The largest of these studies (n=43,367 participants) demonstrated a significant positive multivariate association with falls and had a high-quality assessment score. The second largest (n=17,712) demonstrated no significant association and also had a high-quality assessment (QA) score. Finally, the third largest (n=14,881) demonstrated a significant univariate association with an intermediate QA score. There is a need for interventional studies in frail older adults.
Orthostatic hypotension (OH)** (n=67)	33 Cross sectional; 8 Case-control, 26 Cohort. (15 studies utilised BTB measurement)	Community (33); Hospital (27; Long term/residential care facility (7)	407 (26 - 9,704)	26 (16 m, 10 u) (38.8%)***<br>QA: 7.3 (5-10)	-	43 (64.2%)***<br><br>QA: 7.3 (4-10)	Beat-to-beat (BTB) measurement of OH demonstrated a positive association with falls in 12/15 studies. When sphygmomanometer (auscultatory, oscillometric or unspecified) was used, the majority of studies showed no association between OH and falls (34/46 studies) Baseline measurement position also appeared an important factor with 35.6% of studies commencing in the supine position demonstrating a positive relationship compared to 20% of those studies commencing in the seated position. Refer to eTable 3B for further details.
Low blood pressure (n=4)	1 Case-control, 3 Cohort	Community (3), Hospital (1)	1,160 (270 - 6,595)	1 (m)<br><br>QA: 5	-<br><br>-	3<br><br>QA: 8,8,8	There were a limited number of studies available. 3/4 demonstrated no relationship between low blood pressure and falls.
Postprandial hypotension (n=4)	1 Cross sectional; 2 Case-control; 1 Cohort	Hospital (2), Long term/residential care facility (2)	155 (45 - 499)	2 (1 m, 1 u)<br>QA: 5, 8	-	2<br>QA: 6, 8	There were a limited number of studies available with small sample sizes. The literature remains inconclusive. Further research is required in this area

Continued							
**Cardiovascular disorder (*n* of studies)**	**Study design**	**Setting (n)**	**Sample size - Median (Range)**	**n of positive associations (%)**	**n of negative associations (if any) (%)**	**n of studies with no association (%)**	**Main findings**
				**Cardiac**			
Coronary artery disease (n=60)	29 Cross sectional; 9 Case-control; 21 Cohort	Community (40); Hospital (9); Long term/residential care facility (10)	839 (58 - 159,336)	18 (8 m, 10 u) (30.0%)<br>QA: 6.8 (4-8)	1 (u) (1.7%)<br>QA: 6	41 (68.3%)<br>QA: 7.3 (4-10)	Whereas majority of studies showed no association with falls, a positive association was found in the 3 largest studies (with over 100,000 participants each and high QA scores).
Heart failure (n=23)	8 Cross sectional; 7 Case-control; 8 Cohort	Community (11); Hospital (7); Long term/residential care facility (5)	699 (58 - 135,433)	8 (6 m, 2 u) (34.8%)<br>QA: 6.8 (4-8)	-	15 (65.2%)<br>QA: 6.9 (4-10)	The majority of studies showed no association with falls; of note, the largest 4 studies (n=>10,000 participants) showed inconsistent associations (2 positive, with high QA, and 2 negative, with intermediate to high QA). The studies relate to a chronic history of heart failure as opposed to an acute decompensation event.
Arrhythmia (n=22)	8 Cross sectional; 8 Case-control; 6 Cohort	Community (9); Hospital (9); Long term/residential care facility (4)	432 (51 - 120,881)	8 (5 m, 3 u) (36.4%)<br>QA: 6.3 (4-8)	-	14 (63.6%)<br>QA: 7.6 (4-10)	The majority of studies showed no association with falls; however, a positive association was found in the 2 largest studies (with over n=25,000 participants, and QA scores intermediate to high respectively). Studies in this category refer to the presence of chronic arrhythmia or a history of arrhythmia as opposed to an acute episode correlating with a fall.
Valvular heart disease (n=2)	2 Cohort	Community (1), Hospital (1)	Sample sizes: 215 and 8,172	2 (m)<br>QA: 8, 9	-	-	Both high quality studies demonstrated a positive association with falls. The studies refer to (1) mitral, tricuspid, and pulmonary valve regurgitation, and (2) unspecified murmurs.
				**Reflex**			
Carotid sinus hypersensitivity (n=8)	4 Case-control; 1 Cohort; 3 Interventional	Hospital (7), Long term/residential care facility (1)	103 (34 - 309)	2 (u)<br>QA: 6, 7	-	3<br>QA: 7.0 (6-8)	The association between falls and CSH is inconsistent in the observational literature. 2/5 demonstrate a positive association, 3/5 no association. The interventional data demonstrates that implantation of a device (ILR or PPM-switched on or off) may decrease falls rates. Though these studies possessed small sample sizes and were likely underpowered. Larger randomized control trials are required.
Vasovagal syncope (n=1)	1 Case-control	Hospital (1)	Sample size: 38	-<br>-	-<br>-	1<br>QA: 6	No association was found between vasovagal syncope and falls in a single study.

Continued							
**Cardiovascular disorder (*n* of studies)**	**Study design**	**Setting (n)**	**Sample size - Median (Range)**	**n of positive associations (%)**	**n of negative associations (if any) (%)**	**n of studies with no association (%)**	**Main findings**
				**Other**			
Stroke/transient ischemic attack (n=82)	35 Cross sectional; 11 Case-control; 34 Cohort	Community (49); Hospital (17); Long term/residential care facility (14)	903 (58 - 164,597)	42 (28 m, 14u) (51.2%) QA: 7.0 (4-10)	2 (1 m, 1 u) (2.5%) QA: 7, 7	38 (46.3%) QA: 7.2 (4-10)	51.2% of the studies demonstrated a positive association with falls. Of note, the 4 largest studies (over 100,000 participants and QA scores intermediate to high) showed a positive association. These studies refer to history of stroke as opposed to acute events.
General cardiovascular disease (n=30)	14 Cross sectional; 2 Case-control; 14 Cohort	Community (24); Hospital (3); Long term/residential care facility (3)	1,487 (128 - 16,703)	13 (7 m, 6 u) (43.3%) QA: 6.9 (4-8)	-	17 (56.6%) QA: 7.2 (5-10)	This category has captured non-specific terms used in the literature such as "cardiovascular disease", "heart disease" and "heart problems". Whilst the majority of studies demonstrated no association with falls, it is difficult to make any substantial clinical inferences due to the lack of specificity in the nomenclature utilised. This an issue within the literature which requires rectification in the form of the implementation of higher standards.
Peripheral Arterial Disease (n=7)	6 Cross sectional, 1 Cohort	Community (2); Hospital (4); Long term/residential care facility (1)	416 (163 - 871)	-	1 (m) QA 10	6 QA 7.3 (6-8)	The majority of studies (6/7) demonstrated no association with falls. 1/7 studies demonstrated a negative association.
Arterial stiffness (n=2)	1 Cross sectional, 1 Cohort	Community (2)	Sample sizes: 163 and 531	2 (1 m, 1 u) QA: 8, 9	-	-	Both high quality studies demonstrated a positive association with falls. The measurement techniques utilised were Carotid–femoral Pulse wave velocity and Cardio-ankle vascular index.

Note. m: multivariate. u: univariate. QA: mean score for Newcastle Ottawa Scale quality assessment.
* Percentage reported when n of studies on CVD condition is greater than 10.
** Two studies measured OH twice with different methods.
*** Percentages surpass 100% as some studies reported both positive and negative associations, depending on measurement instrument and OH type.

eTable 2. Hypertension and falls

First author, Year	Falls outcome	Falls assessment method	HTN assessment method	Main findings and prevalence of HTN	OR/RR/HR	Conclusion	Association	NOS
Akande-Sholabi 2020	Any fall	Retrospective (Self reported)	Self reported	45.3% of participants had fallen in the previous 12 months. 63% of fallers had HTN, compared to 65% of non-fallers	Unadjusted OR= 0.9 (0.6, 1.3)	No significant difference reported between falls in the previous 12 months among those with and without a self reported history of HTN	No significant difference	6
Almegbel 2018	Any fall	Retrospective (self reported)	Self reported	Approximately 48% of non-fallers had HTN; 54% of fallers	Unadjusted OR= 1.3 [1.0, 1.6)	No significant difference in the prevalence of HTN among fallers and non-fallers, p=0.24	No significant difference	7
Assantachai 2003	Any fall	Retrospective	Self reported, medical diagnosis of HTN	42% (n=87) of fallers, 25% (n=223) of non-fallers	OR= 1.6 (1.1–2.3)	HTN was associated with falls	Significant multivariate association	6
Banu 2018	Injurious falls	Retrospective (medical records)	Medical records	37 (77.1%) of fallers had HTN. 125 (77.2%) of non-fallers had HTN. P=0.88	Unadjusted OR= 1.1 (0.5–2.4) p>0.05, Adjusted OR: 0.3 (0.1–1.8)	No significant difference between HTN and falls	No significant difference	8
Bergland 2003	Any fall	Prospective	Self reported, medical diagnosis of HTN		OR= 1.8, p<0.02	HTN was associated with future falls	Significant multivariate association	8
Bhangu 2017	All falls, non-accidental falls, syncope	Retrospective (self reported)	Self reported	19.4% of participants had a fall in the previous 12 months; 5.1% had a non-accidental fall; and 4.4% had a syncopal event.	Univariate analyses: All falls: OR= 1.1 (1.0-1.3),p=>.05); non-accidental falls: OR=1.5 (1.2-1.8), p=<0.05); syncope: OR= 1.5 (1.2-1.8), p=<0.05); Adjusted analyses: All falls: OR=1.0 (0.8-1.1),p=>.05); non-accidental falls: OR= 1.1 (0.8-1.5), p=>0.05); syncope: OR= 1.3 (1.0-1.7), p=>0.05	Significant univariate associations between self reported HTN and non accidental falls, and syncope. No significant association between all falls and self reported HTN, or in adjusted models	No significant multivariate association	8
Brassington 2000	Any fall	Retrospective	Self reported, medical diagnosis of HTN	54% of fallers, 44% of non-fallers	Unadjusted OR= 1.5 (1.1–1.9)	HTN was univariably associated with falls	Significant univariate association	6
Callisaya 2014	Multiple falls	Prospective (self reported)	Medical history	47.2% of non fallers or single fallers had HTN; 56.1% of multiple fallers.		No significant difference in the prevalence of HTN among fallers and non-fallers, p>0.05	No significant difference	10
Chan 1997	Any fall	Retrospective	Not given	52.2% (n=37) of fallers and 37.9% (n=126) of non-fallers.	OR= 1.8 (1.1–3.0)	HTN was univariably associated with falls	Significant univariate association	6

First author, Year	Falls outcome	Falls assessment method	HTN assessment method	Main findings and prevalence of HTN	OR/RR/HR	Conclusion	Association	NOS
Chang 2010	Injurious falls	Retrospective	Self reported, medical diagnosis of HTN	49% of fallers, 43% of non-fallers		HTN was not associated with falls	No significant difference	7
Chang 2011	Any fall	Retrospective (reported on patient safety reporting system)	Hospital records	43.0% of fallers had HTN; 51.5% of non-fallers	Unadjusted OR= 0.7 (0.4–1.1), p>0.05	No significant association between HTN and falls	No significant univariate association	7
Chang 2015	Any fall	Retrospective (self reported)	Self reported	18.2% of men with HTN fell; 16.6% of men without HTN. 23.8% of women with HTN fell, 20.9% of women without. HTN	Men: univariate OR= 1.1 (0.1, 1.4) Women: univariate OR=1.2 (1.0, 1.5)	HTN was significantly associated with falls among women, but not among men	Significant univariate association	6
Chen 2008	Any fall	Retrospective (self reported)	Medical records	56.3% of fallers had a history of HTN; 50.5%, of non-fallers p>0.05	Unadjusted OR= 1.3 (0.7, 2.3)	No significant difference in the prevalence of HTN among fallers and non-fallers	No significant difference	8
Chen 2010	Any fall	Retrospective (reported on incident information management system)	Medical records	55.7% of reoccurring fallers had HTN; 64.3% of single fallers; 47.8% of non-fallers, p=0.03	Unadjusted OR= 1.4 (0.7, 2.7)	Significant difference in the prevalence of HTN among reoccurring, single, and non-fallers	No significant difference	7
Choi 2014	Any fall	Retrospective (self reported)	Operational defined by the prescription of medication to treat HTN	48.9% of fallers had HTN; 47.2% of non-fallers, p=0.004.	Unadjusted OR= 1.07 (1.0, 1.1) / Adjusted OR= 0.8 (0.8-0.9,) p<0.001	Significant difference in the prevalence of HTN among fallers and non-fallers. Significant association between falls and HTN	Significant multivariate association	7
Chu 2007	Any fall	Prospective (self reported)	Self reported		Univariate analyses showed a significant association between HTN and falls	Univariate analyses showed a significant association between HTN and falls. However, this was found to be non-significant in multivariate analyses.	No significant multivariate association	7
Damian 2013	Any fall	Retrospective (incident report)	Medical records	45% of cohort, not given for fallers	RR= 1.0 (0.6–1.8)	HTN was not associated with a fall in the past month	No significant multivariate association	4
Del Brutto 2019	Any fall	Retrospective (self reported)	Self reported	45% of fallers had a history of HTN; 40% of non-fallers, p>0.05	Unadjusted OR= 1.6 (0.8, 1.9) / Multivariate OR= 1.1 (0.7–1.8), p>0.05	No association between HTN and falls	No significant multivariate association	8

First author, Year	Falls outcome	Falls assessment method	HTN assessment method	Main findings and prevalence of HTN	OR/RR/HR	Conclusion	Association	NOS
Dokuzlar 2020	Any fall	Retrospective (self reported)	Self reported	54.5% of participants had self reported HTN. 25.4% of participants had a self reported fall in the past 12 months	OR= 1.4, p>0.05	No significant association between self reported HTN, and self reported falls in the past 12 months	No significant multivariate association	7
Downton 1991	Any fall	Retrospective	Sitting blood pressure	Mean sBP was not different between groups		Mean sBP was not associated with falls	No significant difference	6
Gamage 2019	Any fall	Retrospective (self reported)	Self reported diagnosis by a physician	Prevalence of falls was 34.3%; reoccurring falls 9.7%. 54.4% of fallers had HTN; 34.5% of non-fallers had HTN (p=0.001). 72.4% of reoccurring fallers had HTN; 38% of non-reoccurring fallers (single and non-fallers) had HTN (p=<0.001).	Falls (Crude OR= 2.2 (1.3-3.6), p=0.001; reoccurring falls (Crude OR=2.2 (1.3-3.6), p=0.001; Adjusted OR=3.7 (1.0-13.0), p=0.04)	Significant associations reported	Significant multivariate association	8
Goh 2017	Any fall	Retrospective (self reported)	Self reported	51.5% of non-fallers had a previous history of HTN; 53.9% of fallers, p>0.05.	Unadjusted OR= 1.1 (0.8, 1.4)	No significant differences in previous history of self reported HTN in those with and without a self reported fall in the previous 12 months	No significant difference	8
Granek 1987	Any fall	Prospective (incident report)	Medical records	36% of fallers, and 34% of non-fallers had HTN	Unadjusted 1.1 (0.8, 1.5) / Adjusted OR= 1.1, p>0.05	There was no significant association between HTN and falls	No significant univariate association	4
Ha 2021	Any fall (with no external cause e.g., violence)	Retrospective (self reported)	Medical records	65.6% of fallers had HTN; 52.6% of non-fallers (p=0.009)	Adjusted: OR=1.9 (1.2-3.1), p=<0.05	There was a significant difference in the prevalence of HTN between fallers and non-fallers. There was a significant multivariate association between falls and HTN	Significant multivariate association	8
Ham 2017	Injurious falls	Retrospective (self reported)	sBP > 140 mmHg and/or dBP > 90 mmHg	23% of participants experienced a fall. 58.2% of fallers had HTN; 56.9% of non-fallers	Unadjusted OR= 1.0 (0.8, 1.1) (B-Proof study) / 1.1 (1.0, 1.2)	No significant differences reported	No significant difference	9
Hartog 2015	Any fall	Retrospective	Medical records	82% of participants had HTN	Univariate analyses: OR=0.6 (0.2-1.4), p=>0.05	No significant association between HTN and self reported falls within the last 12 months	No significant difference	7
Heckenbach 2014	Any fall	Retrospective (Medical records, ICD-10 codes)	Medical records (GP, ICD code)	From 1,940 participants with HTN, 9.6% were fallers, and 90.4% were non-fallers. Unadjusted OR= 1.3 (1.0-1.6)	OR: 1.1 (0.9-1.4)	HTN was not associated with falls	No significant multivariate association	6

First author, Year	Falls outcome	Falls assessment method	HTN assessment method	Main findings and prevalence of HTN	OR/RR/HR	Conclusion	Association	NOS
Herndon 1997	In ER or admitted for falls	Retrospective	Self reported, medical diagnosis of HTN	7% of respondents had HTN, adjusted	Unadjusted OR= 0.7 (0.5, 1.0) / Adjusted OR= 0.7 (0.5–0.9)	HTN is associated with a decreased risk of injurious falls	Significant (negative) multivariate association	5
Hung 2013	Any fall	Retrospective	Average sBP calculated from sBP (2–4x/day) before discharge (for 3 days).	sBP>140 mmHg was 27% in non-fallers and 23% in fallers. Medical history of HTN 76% in fallers and 79% in non-fallers.	Unadjusted OR= 0.85 (0.5, 1.4)	HTN was not associated with falls in the past year	No significant difference	5
Jacob 2022	Any falls	Retrospective (self reported)	Self reported		OR= 1.1 (1.0-1.3) p>0.05	There was no significant association between HTN and falls	No significant multivariate association	7
Jansen 2015 (a)	Any fall	Retrospective	Self reported, medical diagnosis of HTN	38% of fallers, 37% of non-fallers.	HTN & any falls OR 0.9 (0.8–1.0), HTN & reoccurring falls 1.0 (0.8–1.2)	HTN was not associated with falls	No significant multivariate association	6
Jitapunkul 1998	Any fall in past 6 months	Retrospective	Self reported	28% of fallers, 25% of non-fallers.	Multivariate association between HTN and falls, association not reported	HTN was a risk factor for falls in males	Significant multivariate association	6
Jonsson 1990	Reoccurring falls	Retrospective (medical records or incident reports)	Medical records	50% of fallers, and 35% of non-fallers had HTN (p=>0.05)	Unadjusted OR= 1.9 (0.6, 5.7)	There was no significant difference in the prevalence of HTN between fallers and non-fallers	No significant difference	7
Juraschek 2019	Falls related to hospitalisation or claim	Retrospective (Database surveillance)	HTN was defined as self-report of antihypertensive medication use16 or a systolic BP ≥140 mm Hg or diastolic BP ≥90 mm Hg	11.5% had a fall. 70.8% of non-fallers had HTN; 74% of fallers had HTN.	Unadjusted OR= 1.2 (0.9, 1.5)	No significant differences reported	No significant difference	8
Just 2021	Any fall	Prospective and retrospective (self reported)	Self reported	53.2% of non-fallers had HTN; 52.7% of fallers, p>0.05; 54.7% of multiple fallers, p>0.05	Unadjusted OR= 1.0 (0.8, 1.2)	There was no significant difference in the prevalence of HTN between fallers and non-fallers	No significant difference	8

First author, Year	Falls outcome	Falls assessment method	HTN assessment method	Main findings and prevalence of HTN	OR/RR/HR	Conclusion	Association	NOS
Kamali 2022	Any falls	Retrospective, prospective (self reported)	Omron M3 Intelligence sphygmomanometer in supine position on two occasions in a standard way. High blood pressure in this study was defined as mean sBP≥ 140 mmHg or dBP≥ 90 mmHg.	Falls in last 12 months: no HTN 36 (14.4%) - HTN without meds 41 (16.4%) - HTN with meds 49 (19.6%) p>0.05	Unadjusted OR= 1.17 (0.7, 1.9) / HTN without meds OR=1.1 (0.3-1.8), p>0.05 - HTN with meds OR=1.1 (0.6-1.9), p>0.05	No significant association between HTN and falls	No significant multivariate association	7
Kang 2018	Any fall	Retrospective (self reported)	Medical history	41.5% of fallers had HTN; 46.6% of non-fallers	Univariate OR= 1.2 (0.8-1.8), p>0.05	No significant association between HTN and falls	No significant univariate association	7
Kao 2012	Reoccurring and injurious fall	Retrospective	Self reported	52% of fallers, 52% of non fallers OR= 0.8 (0.5–1.3)	OR= 0.8 (0.5–1.3)	HTN was not associated with falls	No significant multivariate association	6
Kario 2001	Any fall	Prospective and retrospective	BP measured supine, then two standing BP measurements, immediately after standing and 2 min afterwards. Those taking antihypertensive medications were considered treated hypertensives and those with supine BP >=140 mm Hg systolic and/or >=90 mm Hg diastolic in an untreated condition were considered untreated hypertensives.	Falls less common in treated (17%) and untreated (20%) hypertensive subjects compared with normotensives (34%).	Objectively measured sBP (10 mmHg increase) & falls: RR= 0.8 (0.7–0.9)	HTN was associated with a decreased risk of falls	Significant (negative) multivariate association	9
Kelly 2003	Injurious falls, previous 12 months	Retrospective (self reported)	Medical records	31% of fallers had HTN; 31% of controls	Univariate OR= 1.0 (0.9-1.1); Multivariate OR= 0.9 (0.8-1.0)	No significant association between HTN and falls	No significant multivariate association	6

First author, Year	Falls outcome	Falls assessment method	HTN assessment method	Main findings and prevalence of HTN	OR/RR/HR	Conclusion	Association	NOS
Klein 2013	Any fall	Retrospective	sBP and dBP measured in sitting position with mercury sphygmomanometer. sBP/dBP HTN >140/90	24.8% of female fallers had sBP HTN 14.1% of male fallers had sBP HTN 12.7% of females had dBP HTN 9% of males had dBP HTN	dBP HTN women & falls OR= 0.6 (0.4–0.9). dBP HTN men & falls OR=0.9 (0.5–1.5). sBP HTN women & falls OR= 0.7 (0.5–1.0). sBP HTN in men & falls OR= 0.7 (0.4–1.2)	HTN was associated with a decreased risk of falls in women, but not in men.	Significant (negative) multivariate association	5
Kocyigit 2020	Any fall	Retrospective (Self reported history of falls)	Hut test	27.1% of the sample had hypotension.	There was not a significant difference with regard to falls in those with and without HTN, p>0.05	No significant difference with regard to falls in those with and without HTN	No significant difference	7
Kojima 2011	Any fall	Retrospective (self reported)	Medical records	64.1% of participants had HTN	Multivariate OR= 1.9 (0.6–5.8)	No significant association between HTN and falls	No significant multivariate association	7
Ku 2013	Any fall	Prospective (resident records)	Self reported	59.1% of fallers had HTN; 44.5% of non-fallers, p=0.09	Univariate OR= 1.4 (1.0-1.9) p=0.09	No significant difference in the prevalence of HTN between fallers and non-fallers; no significant univariate association between HTN and falls	No significant univariate association	9
Lawlor 2003	Any fall	Retrospective	Oscillometer, 2x seated, sBP >160/95mmHg or receiving treatment for blood pressure	51.6% of fallers and 50.6% of non-fallers, p>0.05		HTN was not associated with falls	No significant difference	4
Lee 2020	Any fall	Retrospective (self reported)	Self reported	56.2% of participants reported s history of HTN. 27.3% of participants had a fall in the previous year.	Unadjusted: 0R= 1.4 (1.4-1.5), p=<0.05); Adjusted: OR= 1.0 (1.0-1.1), p=>0.05	Significant association between falls and HTN in unadjusted analyses, non significant in adjusted analyses	No significant multivariate association	8
Lee 2021	Any fall	Prospectively (self reported)	Self reported	50% of fallers, and 41.1% of non-fallers had HTN, p>0.05	Unadjusted OR= 1.4 (0.8, 2.6)	There was no significant difference in the prevalence of HTN among fallers and non-fallers	No significant difference	7
Liao 2012	Any fall	Retrospective	Sphyg., sBP/dBP >130/85mmHg or use of antihypertensive medication	60% fallers, 50% non-fallers, p=0.009	Unadjusted OR=1.5 (1.1, 2.0)	HTN was more prevalent in fallers than non-fallers	Significant difference	5
Lipsitz 1991	Any fall	Prospective (computer records and reports)	Clinical evaluation	41% of fallers had HTN; 39% of non-fallers, p>0.05	Unadjusted OR=1.1 (0.5, 2.2)	No significant difference in the prevalence of HTN between fallers and non-fallers	No significant difference	8

First author, Year	Falls outcome	Falls assessment method	HTN assessment method	Main findings and prevalence of HTN	OR/RR/HR	Conclusion	Association	NOS
Magnuszewski 2020	Any fall	Retrospective (self reported)	Self reported	81.5% of fallers had HTN; 79.6% of non-fallers, p>0.05	Unadjusted OR=1.1 (0.7, 1.9)	No significant difference in the prevalence of HTN between fallers and non-fallers	No significant difference	8
Magnuszewski 2022	8 months	Prospective (incident reports)	Medical records	Fallers 85.7%, non-fallers 78.4% p>0.05	Unadjusted OR=1.6 (0.4, 7.5)	No significant difference in the prevalence of HTN between fallers and non-fallers	No significant difference	8
Margolis 2019	Any fall	Prospective (self reported)	BP < 140/90 mm Hg	70% had HTN. 21.3% of those with no HTN reported a single fall, 20.8% recurring falls; treated controlled HTN 23.6% had a single fall, 20.4% reoccurring falls; treated uncontrolled HTN 21.2% had a single fall, 22.0% reoccurring falls; untreated uncontrolled HTN 24.6% a single fall, 16.9% reoccurring falls	Unadjusted OR= 0.9 (0.7, 1.2) / Unadjusted: Treated controlled HTN IRR=0.8 (0.7-.9), p=<0.001 treated uncontrolled HTN IRR=0.9 (0.8-1.1), p>0.05, Untreated uncontrolled HTN IRR=1.2 (0.9-1.5), p>0.05; Adjusted: Treated controlled HTN IRR=0.8 (0.7-.9), p=<0.001, treated uncontrolled HTN IRR=0.7 (0.6-0.9), p=<0.001, Untreated uncontrolled HTN IRR=1.2 (0.9-1.5), p>0.05	Unadjusted: Significant association between treated controlled HTN and no HTN. No significant difference between treated uncontrolled, or untreated uncontrolled HTN. Adjusted: Significant association between treated controlled HTN and treat uncontrolled HTN and no HTN. No significant difference between in treated uncontrolled HTN and no HTN with regard to falls	Significant multivariate association	8
Maurer 2005	Any fall	Prospective (incident reports)	Medical records and elf reported, continuous; sBP/dBP >140/90 or use of anti-hypertensives	55% of cohort	Unadjusted HR= 2.0 (1.1–3.7)	Patients with HTN are more likely to suffer future falls	Significant multivariate association	8
Mitchell 2013	Any fall in past 12 months	Retrospective	Self reported, medical diagnosis of HTN	54% of fallers, 51% of non-fallers.	Unadjusted OR= 1.1 (1.0–1.3)	HTN is not associated with falls	No significant univariate association	6
Mitchell 2015	Any fall	Retrospective (self reported)	Self reported		Unadjusted; RR=1.0 (1.0-1.1), p=0.2	No significant association reported	No significant univariate association	8
Ooi 2021	Any fall	Retrospective and prospective (self reported)	Self reported	51.8% of fallers had HTN; 49.1% of non-fallers, p>0.05; 54.1% of reoccurring fallers, p>0.05	Unadjusted OR= 1.1 (0.8, 1.5)	There were no significant differences in the prevalence of HTN between non fallers and occasional fallers, and reoccurring fallers	No significant difference	7

First author, Year	Falls outcome	Falls assessment method	HTN assessment method	Main findings and prevalence of HTN	OR/RR/HR	Conclusion	Association	NOS
Oren 2022	Any falls	Retrospective (medical records)	Medical records	218 (84.2%) of fallers had HTN, 165 (37.6%) of controls had HTN	Unadjusted OR= 8.8 (6.0–13.0) p <0.001 - Adjusted OR= 10.3 (5.8–18.5) p<0.001	There was a significant association between HTN and falls	Significant multivariate association	7
Paganini-Hill 2017	Any fall	Retrospective (self reported)	Medical history	50% of non-fallers had HTN, 53% of fallers, p>0.05	Unadjusted OR= 1.1 (0.9, 1.4)	No significant difference in prevalence of HTN among fallers and non-fallers	No significant difference	8
Prudham 1981	Any fall	Retrospective (self reported)	Self-reported	23.4% of fallers had HTN; 21.8% of non-fallers, p>0.05		No significant difference in the prevalence of HTN between fallers and non fallers	No significant difference	4
Rivan 2021	Any falls, injurious falls	Retrospective	Self reported	Fallers. 68 (50%) of fallers had HTN, 123 (47.1%) of non fallers. P=0.598 - injurious falls 46 (47.4%) injurious fall had HTN, 172 (48.6%) of non injured with HTN p>0.05	Unadjusted Or= 1.15 (0.76, 1.73)	No significant association between HTN and falls or injurious falls	No significant difference	7
Rivera-Chavez 2021	Any fall	Retrospective (self reported)	Direct interrogation and clinical history, as part of comprehensive geriatric assessment	72%% of fallers had arterial HTN; 73% of non-fallers, p>0.05	Univariate: 0R=1 (0.7-1.3), p>0.05	There was no significant association between falls and arterial HTN, or significant difference in the prevalence of arterial HTN among fallers and non-fallers	No significant univariate association	7
Saedon 2016	Multiple falls or injurious falls	Retrospective (self reported)	Objective digital plethysmography	54.2% of fallers had HTN, 48.2% of fallers, p>0.05	Unadjusted OR= 1.27 [0.78, 2.07]	No significant difference in the prevalence of HTN between fallers and non-fallers	No significant difference	6
Sharma 2017	Any fall	Retrospective (self reported)	Medical history (self reported)	49.8% of non-fallers had HTN, compared to 53.6% of fallers, p>0.05	Unadjusted OR= 1.30 [0.79, 2.15]	No significant difference in HTN among fallers and non-fallers	No significant difference	7
Sibley 2014	Any fall	Retrospective	Self reported, medical diagnosis of HTN	21% of those with HTN fell, compared to 18% of people without HTN	A cluster 'HTN' was associated with falls, OR 1.2	HTN is associated with falls	Significant multivariate association	6
Song 2021	Any fall	Retrospective (self reported)	A mercury sphyg was used to measure BP while participants were in a seated position in their home. Two measurements were taken and the average of the two values was used for analyses. High sBP ≥ 140 mmHg		Any fall: Multivariate: OR=1.2 (1-1.4), p>0.05; Injurious falls: Multivariate: OR=1 (0.8-1.4), p>0.05	No significant association between HTN and falls, or injurious falls	No significant multivariate association	8

First author, Year	Falls outcome	Falls assessment method	HTN assessment method	Main findings and prevalence of HTN	OR/RR/HR	Conclusion	Association	NOS
Subramanian 2020	Any fall	Retrospective	Self reported	52.6% of fallers had HTN; 54.1% of non-fallers, p>0.05	Unadjusted OR= 0.94 [0.45, 1.96]	No significant difference in the prevalence of HTN between fallers and non-fallers	No significant difference	5
Svensson 1992	Any falls, previous 12 month	Retrospective (self reported) interview	Clinical evaluation	96 (32.0%) of fallers had HTN v 148 (33.0%) non fallers - not significant.	Unadjusted OR= 0.95 [0.70, 1.31]	No significant difference in HTN among fallers and non-fallers	No significant difference	7
Teoh 2021	Any fall, previous 12 months	Retrospective (self reported)	HTN was defined as the presence of an elevated mean systolic (≥130 mmHg) and/or diastolic (≥85 mmHg) blood pressure or use of blood pressure-lowering therapy	58.8% of non fallers had HTN; 65.2% of fallers had HTN, p>0.05	Unadjusted OR= 1.31 [1.01, 1.70]	There was a significant difference in the prevalence of HTN between fallers and non-fallers	Significant difference	8
Thapa 2022	Any falls	Medical records	Medical records	96 (62.7%) of fallers had HTN, 1,121 (70.2%) of non-fallers had HTN, p>0.05	Unadjusted OR= 0.71 [0.51, 1.01]	No significant difference in the prevalence of HTN between fallers and non-fallers	No significant difference	6
Tsai 2021	Any fall	Retrospective (self reported) interview	Medical records	187 (9.4%) of people with HTN fell, 1,813 (90.7%) of people with HTN did not fall. Unadjusted OR= 1.2 (1.0-1.5), p=0.03	Adjusted OR= 1.0 (0.8-1.2), p>0.05	No significant association between HTN and falls	No significant multivariate association	6
Turusheva 2020	Any falls, injurious falls	Retrospective (self reported)	Medical records	5.6% of patients with a fall had HTN; 23.4% without a fall had HTN, p=>0.05	Unadjusted OR= 0.19 [0.02, 1.50]	No significant difference in HTN between fallers and non-fallers	No significant difference	8
Valderrama-Hinds 2018	Any fall, previous 24 months	Retrospective (self reported)	Self reported	44.8% of fallers had HTN; 38.4% of non-fallers, p=0.007	Unadjusted OR= 1.0 (0.9–1.1)	Significant difference in the prevalence of HTN between fallers and non-fallers; no significant association between falls and HTN	No significant univariate association	6
Vieira 2018	Any falls, previous 12 months	Retrospective (self reported)	Self reported		Gross PR=1 (0.9-1.2), p=0.6, Adjusted PR=0.85 (0.7-1). P=0.1	No significant difference in falls in those with and without HTN	No significant difference	8
Wong 2014	Any fall	Prospective	Self reported	55% of fallers and 62% of non-fallers.	Unadjusted OR= 0.76 [0.53, 1.10] / HTN & falls unadjusted RR= 0.9 (0.7–1.0)	HTN is not associated with falls	No significant univariate association	9

First author, Year	Falls outcome	Falls assessment method	HTN assessment method	Main findings and prevalence of HTN	OR/RR/HR	Conclusion	Association	NOS
Xu 2015	Any fall	Prospective	Self reported medical history	62.2% of fallers had HTN; 61.6% of non-fallers, p>0.05	Unadjusted OR= 1.02 [0.66, 1.59] / Multivariate RR= 0.94 (0.6-1.4), p>0.05	No significant difference in the prevalence of HTN between fallers and non-fallers; no significant multivariate association between falls and HTN	No significant multivariate association	10
Yasumura 1994	Any fall	Retrospective (self reported)	Self reported		Multivariate OR= 1.4 (0.7-2.8)	No significant association between HTN and falls	No significant multivariate association	7
Yoo 2016	Any fall	Retrospective	Self reported	City: 70.5% of fallers had HTN, 66.7% of non-fallers, p>0.05; Rural: 73.1% of fallers had HTN, 71.9% of non-fallers, p>0.05		No significant difference in the prevalence of HTN between fallers and non-fallers	No significant difference	6
Yu 2009	Any fall	Retrospective	Not specified	146 (21.2%) of fallers had HTN; 126 (15.3%) of fallers had no HTN	OR=1.8 (1.1-1.8) p=0.022	Significant association between HTN and falls	Significant multivariate association	7
Zhao 2019	Any fall	Retrospective (self reported)	Self reported	82.6% of participants had HTN. 85.7% of fallers had HTN; 82% of non-fallers had HTN, p>0.05	Unadjusted OR= 1.26 [0.88, 1.80] / Adjusted OR=1.6 (1.0-2.5), p=0.04	No significant difference in HTN between fallers and non-fallers; significant association in HTN between those with and without falls	Significant univariate association	7
Zia 2015	Reoccurring or injurious falls	Retrospective (self reported)	Self reported	56.9% of fallers had HTN, 49.4% of non-fallers, p>0.05	Unadjusted OR= 1.36 [0.89, 2.06]	No significant difference in HTN among fallers and non-fallers	No significant difference	9

Note. SD: standard deviation. IQR: interquartile range. OR: odds ratio. HR: hazard ratio. RR: relative risk. HTN: Hypertension. NOS: Newcastle Ottawa Scale

eTable 3A. Orthostatic hypotension and falls

First author, Year	Falls outcome	Falls assessment method	Assessment method of OH	Main findings and prevalence of OH	OR/RR/HR	Conclusion	Association	NOS
Almegbel 2018	Any fall	Retrospective (self reported)	Self reported	Approximately 50% of fallers and non-fallers had OH		No significant difference in OH among fallers and non-fallers (p=0.33)	No significant difference	7
Arseven 2008	Non-syncopal falls	Prospective (self reported)	Patient report, a primary care physician questionnaire, and medical record review, including laboratory results and medications	5.4% of fallers had OH; 8.5% of non-fallers, p=0.5	Unadjusted OR= 0.62 [0.13, 2.95]	No significant difference in the prevalence of OH among fallers and non-fallers.	No significant difference	10
Aydin 2017	Reoccurring falls	Retrospective (self reported or reported by patients relatives)	Objectively measured. OH diagnosis was defined as a drop of at least 20 mmHg in sBP and/or 10 mmHg in dBP upon the change in position in both AST and HUT.	Assessed by Head Up Tilt (HUT), 19% of the sample had OH, and 36.4% of those with OH had reoccurring falls compared to 18.5% of those without (p>0.01); assessed by Active Stand (AST) 37% of the sample had OH, and 21.7% of those with OH had reoccurring falls, compared to 225 without (p>0.05)	Unadjusted OR= 0.96 [0.54, 1.71]	There was a significant difference in the prevalence of reoccurring falls between those with and without OH when assessed by HUT, but not when assessed by AST	Significant difference with HUT; no significant difference with AST	7
Bumin 2002	Any fall	Retrospective	Sphygmomanometer, sitting and standing at 3 min, 20 sBP	44% of fallers, 18% of non fallers	Unadjusted OR= 3.63 [0.74, 17.81]	OH was univariately associated with falls	Significant difference	5
Campbell 1981	Any fall	Retrospective	Supine and standing at 1 and 3 min, sphyg., 20 sBP	13% (74/559) of total sample, considered attributable cause of a fall in 3%.		OH considered an attributable cause of a fall in 3%.	No significant difference	4
Campbell 1989	Any fall	Prospective	(oscillometric), Lying and standing at 1 and 3 min. sphyg, 20 sBP	40% in female fallers and 31% in female non-fallers, 22% in male fallers and 29% in male non-fallers.	Unadjusted OR= 1.47 [0.98, 2.20] (Women) - 0.71 [0.37, 1.35] (Men) / Postural OH & falls RR= 1.5 (1.0–2.3) in women	OH was not significantly associated with future falls.	No significant difference	9
Chan 1997	Any fall	Retrospective	(auscultatory), Standing at 3 min., sphyg, 20 sBP	7.2% (n=5) in fallers and 10.5% (n=35) in non-fallers.	Adjusted OR=0.7 (0.3–1.8)	OH was not associated with falls.	No significant association	6

First author, Year	Falls outcome	Falls assessment method	Assessment method of OH	Main findings and prevalence of OH	OR/RR/HR	Conclusion	Association	NOS
Chang 2010	Any injurious fall	Retrospective	Sphygmomanometer, Supine and standing, immediately,20/10	36% in fallers, 24% in non-fallers. Prevalence of OH in injurious fallers higher than in non-injurious fallers.	OH & falls unadjusted OR = 1.30 [0.96, 1.76] / OH & injurious falls vs non-injurious falls OR=2.3 (1.1–5.12) OH & remarkable injury vs. no injury: OR=4.0 (1.6–10.0).	OH and any falls were not associated. OH was associated with injurious falls compared to non-injurious falls	Significant multivariate association	7
Chu 2007	Any fall	Prospective (self reported)	Self reported		Univariate analyses showed a significant association between OH and falls	Univariate analyses showed a significant association between OH and falls. However, this was found to be non-significant in multivariate analyses	No significant multivariate association	7
Claffey 2022	Any fall	Retrospective (self reported)	Active stand test using a Finometer calibrated against BP measured by a brachial sphygmomanometer and servo-calibration was switched off during the recording.		Asymptomatic OH OR=2.0 (1.1-3.7) p=0.02, Symptomatic OH OR=0.9 (0.3-2.2) p>0.05	Participants with asymptomatic OH had a significantly higher risk of unexplained falls during follow-up	Significant multivariate association	8
Coutaz 2012	Any fall	Retrospective (self reported)	Self reported	46.5% of those with OH had a fall in the previous 6 months; 40.3% of those without OH (p=0.225)	Unadjusted OR= 1.29 [0.83, 1.99]	There was no significant difference in the prevalence of falls between those with and without OH	No significant difference	7
Davies 2001	Cases: non-accidental falls. Controls: accidental falls or other	Retrospective	Active stand for 2 min, continuous, 20 sBP	31% cases, 19% controls	Unadjusted OR= 1.96 [0.66, 5.75]	Prevalence of OH was higher in accidental fallers than controls.	Significant difference	6
Dokuzlar 2020	Any fall	Retrospective (self reported)	Active stand assessment (auscultatory sphygmomanometer BP measurement)	36.9% of participants had self reported OH. 25.4% of participants had a self reported fall in the past 12 months	OR= 0.9, p>.05	No significant association between self reported OH, and self reported falls in the past 12 months	No significant multivariate association	7

First author, Year	Falls outcome	Falls assessment method	Assessment method of OH	Main findings and prevalence of OH	OR/RR/HR	Conclusion	Association	NOS
Donoghue 2021	Reoccurring falls; injurious falls; unexplained falls; and syncope, 60 months	Prospective (self reported)	BTB was measured during an AS procedure using digital photoplethysmography. sBP, dBP and HR were recorded during the 10 minutes rest period and for 2 minutes after standing. Supine HTN was defined as sBP of ≥ 140 mm Hg or dBP of ≥ 90 mm Hg using the mean blood pressure data during the first 30 seconds of the minute before standing. OH was defined as a drop of ≥ 20 mmHg in sBP and/or ≥ 10 mm Hg in dBP within 3 minutes of standing up. Impaired stabilisation (OH40) was defined as a drop in sBP of ≥ 20 mm Hg or dBP of ≥10 mm Hg at 40 seconds. Sustained OH was defined as a drop in sBP of ≥ 20 mm Hg or dBP of ≥ 10 mm Hg sustained at all timepoints between 60 and 120 seconds after standing		Reoccurring falls: Multivariate: OH(40) RR=1.3 (1-1.7), p=<0.05; Sustained OH RR=1.3 (0.9-1.8), P=>0.05; Injurious falls: Multivariate: OH(40) RR=1.4 (1.3-1.8), p=<0.01; Sustained OH RR=1.6 (1.2-2.1), p=<0.01; Unexplained falls: Multivariate: OH(40) RR=1.6 (1.1-2.1), p=<0.01; Sustained OH RR=1.6 (1.1-2.5), p=<0.05; Unexplained falls: Multivariate: OH(40) RR=1 (0.7-1.5), p=>0.05; Sustained OH RR=1 (0.7-1.7), p=>0.05	There was a significant multivariate association between reoccurring falls and OH40, but not sustained OH; injurious falls and OH(40), and sustained OH; unexplained falls and OH(40) and sustained OH; unexplained falls and OH(40) and sustained OH; and none between syncope and OH(40), and sustained OH	Significant multivariate association	8
Downton 1991	Any fall	Retrospective	Sphygmomanometer, Sitting and standing at 1 and 2 min., 20 sBP	31% of subjects; equal between fallers and non-fallers.		OH was not associated with falls	No significant difference	6
Ensrud 1992	Any fall	Retrospective	(auscultatory), Supine and standing at 1 minute, sphyg, 20 sBP		Falls and OH: OR=1.0 (0.9–1.2)	OH was not associated with a history of falls	No significant difference	7

First author, Year	Falls outcome	Falls assessment method	Assessment method of OH	Main findings and prevalence of OH	OR/RR/HR	Conclusion	Association	NOS
Finucane 2017	Falls, unexplained falls, injurious falls, syncope; multiple falls;	Prospective (self reported)	OH, IOH, OH (40) assessed by active stand assessment utilising a calibrated beat to beat volume clamp method. OH defined as sustained failure of sBP 158 or dBP to stabilise to within 20mmHg sBP or 10mmHgdBP of supine levels throughout the 159 active stand; IOH defined as an initial drop in sBP≥40mmHg and/or drop indBP≥20mmHg occurring 154 within 15 seconds of standing (with or without symptoms): OH(40) was defined by failure to return to within sBP≥20mmHg and/or dBP 157 ≥10mmHg of supine levels at 40s after standing	15.8% of participants with OH had a fall, compared to 13.4% without; 11.3% had multiple falls, compared to 8% without, 7.7% had unexplained falls, compared to 4% without*; 15.4% had injurious falls, compared to 8.6% without*, 8.1% with syncope, 4.6% without*; 18.2% of participants with OH(40) had a fall, compared to 12.8% without*, 10.2% of participants with OH(40), 7.9% without*, 6.4% had unexplained falls, 3.9% without*, 13.1% had injurious falls, 8.3% without*, 6.2% had syncope, 4.5% without; 12.8% of participants with IOH had a fall; compared to 13.9% without; 8.2 of participants with IOH had multiple falls, 8.3% without, 3.6% had unexplained falls, 4.6% without, 8.2% had injurious falls, 9.3% without, 3.5% had syncope, 5.4% without* (Significant differences marked with an *)	Unadjusted OR= 1.36 [1.00, 1.85] / All-cause falls: IOH RR=1.1 (0.9-1.3), p>0.05; OH(40) RR=1.2 (1-1.6), p>0.05), RR=1.4 (1.0-2.0), p=0.04; Unexplained falls IOH RR=0.9 (0.7-1.3), p>0.05, OH(40) RR=1.5 (1.0-2.3), p=0.04; OH RR=1.8 (1.1-3.1), p>0.05; Injurious falls: IOH RR=0.9 (0.8-1.2), p>0.05, OH(40) RR=1.3 (1-1.7), p>0.05, OH=1.6 (1.1-2.2), p=0.01; Syncope IOH RR=0.8 (0.5-1.1), p>0.05, OH(40) RR=0.9 (0.6=1.4), p>0.05; OH RR=1.2 90.7-2.1), p=0.5	Significantly increased risk of all falls in those with OH; unexplained falls in those with OH(40) and OH. Also a significantly increased risk of injurious falls in those with OH. All other measures non-significant	Significant multivariate association	8
Frels 2002	Any fall	Prospective (incident book)	Medical records	4% of fallers, and 1% of non-fallers had a postural OH (p=>0.05)	Unadjusted OR= 3.60 [0.74, 17.57]	There was no significant difference in the prevalence of previous stroke between fallers and non-fallers	No significant difference	7

First author, Year	Falls outcome	Falls assessment method	Assessment method of OH	Main findings and prevalence of OH	OR/RR/HR	Conclusion	Association	NOS
Freud 2015	Any fall	Retrospective (self reported)	Assessed using an oscillometric electronic instrument. First standing blood pressure measurement taken within 1 minute of standing and was repeated at the third minute of standing, drop in sBP of ≥20 mm Hg, a drop in dBP of ≥ 10 mm Hg	No differences in the number of falls between those with OH, and without OH. 32.1% of participants had OH		No differences in the number of falls between those with OH, and without OH, in the previous 12 months	No significant difference	8
Gangavati 2011	Reoccurring falls	Prospective	(auscultatory), Supine and standing at 1 and 3 min., sphyg, 20/10	Falls similar in those with and without OH. : 39% of participants with uncontrolled HTN and OH had reoccurring falls, vs. 17% in those without OH.	Unadjusted OR= 0.63 [0.40, 0.98] / reoccurring falls & OH at 1 min in uncontrolled HTN: HR= 2.5 (1.3–5.0).	OH was associated with future reoccurring falls in those with uncontrolled HTN.	Significant multivariate association	9
Graafmans 1996	Any fall, reoccurring falls	Prospective	Sphygmomanometer, Supine and standing at 1 minute, 20/10		OH & falls unadjusted OR= 1.34 [0.76, 2.36]: adjusted OR=1.4 (0.8–4.8) p>0.05. OH & reoccurring falls: adjusted OR=2.0 (1.0–4.2)	OH was associated with future reoccurring falls but not with any falls	Significant multivariate association	9
Ham 2017	Injurious falls	Prospective (self reported)	OH was measured using an automatic oscillometric blood pressure recorder. A decrease of ≥20 mmHg in systolic and/or a decrease of ≥10 mmHg in dBP.	23% of participants experienced a fall. 13.8% of fallers had OH; 13.5% of non-fallers	Unadjusted OR=1.03 [0.87, 1.22]	No significant differences reported	No significant difference	9
Hartog 2015	Any fall	Retrospective	Blood pressure measurement using oscillometric sphygmomanometer (Omron M6). Blood pressure was measured twice in supine proton, and twice after postural change after 1 and 3 minute.	36.7% had OH	Univariate: OR=0.6 (3-1.4), p=>0.05	OH was not significantly associated with falls	No significant multivariate association	7

First author, Year	Falls outcome	Falls assessment method	Assessment method of OH	Main findings and prevalence of OH	OR/RR/HR	Conclusion	Association	NOS
Hartog 2017	Any fall	Retrospective (self reported)	Blood pressure was measured following a standardized protocol, using an oscillometric device (Omron M6). 1996 OH definition: a drop in sBP of >20 mmHg or dBP of >10 mmHg after postural change; 2011 OH definition: a drop in sBP of >30 mmHg or dBP of >10 mmHg after postural change. Both measured three minutes after postural change	38% of participants who had OH according to 1996 definition, experienced a fall	1996 definition: OR=1.0 (0.8-1.3); 2011 definition: OR=1.1 (0.8-1.4)	No significant association between self reported falls in the previous year, and OH, as operationally defined by either definition	No significant association	8
Heitterachi 2002	Any fall	Prospective	HUT at 60 degrees, continuous, 20 sBP	OH at 3 min.: 22% of fallers, 6% of non-fallers.	Unadjusted OR= 4.57 [0.90, 23.34] / OH at 3 min. & falls: RR 1.7 (1.1–2.6).	OH at 3 min. after HUT was associated with future falls.	Significant multivariate association	9
Jodaitis 2015	Reoccurring falls	Retrospective (self reported)	HUT test by tilt table. A decrease of at least 20 mmHg in sBP or of at least 10 mmHg in dBP within 3 minutes after standing.	41% had OH; 49% had reoccurring falls. 62% of those with OH, had reoccurring falls; 40% of those without OH, p=<0.001). Further, falls was the reason for admission for 49% of those with OH; compared to 29% of those without, p=<0.001	Univariate OH= 2.7 (1.7–4.5)	Significant differences were observed between those with and without OH, with regard to reoccurring falls, and falls being the reason for hospital admission	Significant difference	5
Juraschek 2022	Any falls	Prospective (self reported)	OH was defined as a drop in systolic or diastolic BP of at least 20 or 10 mmHg, measured at pre-randomization, 3-, 12-, and 24-month visits with each of 2 protocols: seated-to-standing and supine-to standing.	76.2% of participants with OH (seated) experienced falls in the previous year, 62.4% of participants with no OH (seated) did not experience falls in the previous year.66.1% of participants with OH (supine) experienced falls in the previous year, 62.1% of participants with no OH (supine) did not experience falls in the previous year.	OH and falls (seated)unadjusted OR=1.93 [0.70, 5.35] HR 0.7 (0.3, 1.6) p= 0.4 OH and falls (supine)unadjusted OR= 1.19 [0.76, 1.85] HR= 1.6 (1.0, 2.5) p=0.08	No significant association between OH and falls	No significant multivariate association	9

First author, Year	Falls outcome	Falls assessment method	Assessment method of OH	Main findings and prevalence of OH	OR/RR/HR	Conclusion	Association	NOS
Kamali 2022	Any falls	Retrospective, prospective, (self reported)	Self reported		OR=1.1 (0.7-1.7), p>0.05	No significant association between OH and falls	No significant multivariate association	7
Kario 2001	Any fall	Prospective, retrospective	(auscultatory), Supine, immediately after standing and at 2 min. Sphygmomanometer, 20/10	OH not different between fallers and non-fallers		OH was not associated with future falls	No significant difference	9
Kocyigit 2020	Any fall	Retrospective (Self reported history of falls)	HUT test by tilt table, beat to beat assessment (Gemesan1 Tilt Table G-71, Turkey). 20 or 10-mmHg drop in systolic and/or dBP from supine to standing position.	17.4% of the sample had OH. 43.4% of those with OH had a fall, compared to 35.4% of those without, p=0.03	Unadjusted OR= 1.39 [0.94, 2.06] / OR= 1.36 (0.9-2.1), p>0.05	No significant association observed between OH and falls. Significant difference was observed with regard to falls in those with and without OH	No significant multivariate association	7
Kocyigit 2021	Any fall	Retrospective and prospective (self reported)	The HUT table test was applied for the diagnosis of OH. The HUT table test was performed using a tilt table. Monitoring over the course of the HUT test was performed. The measurements within the 3rd min of standing were recorded, and patients were questioned as to whether they had postural symptoms, such as dizziness and nausea. OH was diagnosed if the blood pressure dropped by 20+ mmHg systolically and 10+ mmHg diastolically during the transition from the supine to the standing position during the HUT test	32.6% of those without OH had falls; 43.4% with, p=0.006	Unadjusted OR= 1.58 [1.14, 2.20]	Significant difference in the prevalence of falls among those with, and without, OH	Significant difference	7

First author, Year	Falls outcome	Falls assessment method	Assessment method of OH	Main findings and prevalence of OH	OR/RR/HR	Conclusion	Association	NOS
Lawlor 2003	Any fall	Retrospective	(oscillometric), Mean of two standing measurements with sphyg, 20/10	17.6% of fallers and 17.1% of non-fallers	Unadjusted OR= 1.04 [0.84, 1.29]	OH was not associated with falls in the past year	No significant difference	4
Lipsitz 1991	Any fall	Prospective (computer records and review of incidence reports)	Clinical evaluation	6% of fallers had atrial fibrillation or a flutter; 2% of non-fallers, p>0.05	Unadjusted OR= 3.33 [0.36, 30.70]	No significant difference in the prevalence of OH between fallers and non-fallers	No significant difference	8
Liu 1995	Any fall	Prospective	Immediately on standing and after 5 min., sphyg, 20/10	Prevalence OH 3–15%, no difference between fallers and non-fallers		OH is not associated with future falls	No significant difference	9
Luukinen 1996	Reoccurring falls	Prospective	Sitting and standing at 1 minute, sphyg, 20 sBP	35% in fallers, 29% in non-fallers	Unadjusted OR= 1.27 [0.79, 2.03] / RR 1.3 (0.8–1.9)	OH was not associated with future falls	No significant multivariate association	9
Mader 1987	Any fall	Retrospective	(auscultatory), Supine and standing at 1 minute sphyg, 20 sBP.	7% of fallers, 12% of non-fallers	Unadjusted OR= 0.52 [0.19, 1.41]	OH was not associated with falls in the past year	No significant difference	5
Magnuszewski 2020	Any fall	Retrospective (self reported)	Self reported	16.6% of fallers had OH; 15.0% of non-fallers, p>0.05	Unadjusted OR= 1.27 [0.70, 2.31]	No significant difference in the prevalence of OH between fallers and non-fallers	No significant difference	8
Magnuszewski 2022	Any fall	Prospective (incident reports)	Medical records	Fallers 42.9%, non-fallers 15.0% p=0.01	Unadjusted OR= 5.16 [1.72, 15.48] / OR= 21.5 (2.3–206.2), p<0.01	Significant multivariate association between OH and falls.	Significant multivariate association	8
Maurer 2004	Any fall	Prospective (incident reports)	Beat to beat, Sitting and standing for 5 min., continuous, 20/10		OH at 1-minute & falls HR 0.98 (0.5–2.0), OH at 3 min. & falls HR 1.3 (0.7–2.5)	OH was not associated with future falls	No significant multivariate association	8

First author, Year	Falls outcome	Falls assessment method	Assessment method of OH	Main findings and prevalence of OH	OR/RR/HR	Conclusion	Association	NOS
McDonald 2017	Any fall	Prospective (self reported)	Continuous beat-to-beat BP was recorded from the hand using digital photoplethysmography. OH was defined as a 20-mmHg drop in systolic BP and/or a 10-mmHg drop in diastolic BP within 3 min of standing unless either (i) the participant has supine HTN in which case a fall in systolic BP of at least 30 mmHg is required for a diagnosis of OH or (ii) the BP nadir occurred within the first 15 s of standing in which case a 40-mmHg systolic BP and/or 20- mmHg diastolic BP was required for a diagnosis of OH	OH defined according to the 2011 criteria affected 40% of fallers and 12% of non-fallers, p= 0.004	Unadjusted OR= 4.78 [1.55, 14.70] / OH defined by 2011 criteria remained an independent predictor of falls OR= 10.3 (1.70–61.4) p=0.011	OH was significantly associated with falls, and significantly different in falls compared to non-fallers	Significant multivariate association	9
Menant 2016	Unexplained falls	Prospective	Brachial BP was measured using an oscillometric sphygmomanometer. OH (OH) was defined as a reduction of 20 mmHg or more in sBP or 10 mmHg or more in dBP within 3 minutes of tilting	22.4% of non-fallers had OH, 39.4% of unexplained fallers, p=<0.05, and 20.5% of balance related fallers.	Multivariate OR=2.3 (1.1-4.9), p=<0.05	There was a significant difference in OH between non-fallers and unexplained fallers, and a significant association between OH and falls	Significant multivariate association	10
Miu 1997	Any fall	Retrospective	Ausc - Mercury sphyg. OH = 20mmhg drop in systolic on standing	53/400 = fallers. 15 (16.5%) of fallers had OH compared to 38 (12.3%) non fallers, p>0.05	Unadjusted OR= 1.41 [0.74, 2.69]	No significant association between OH and falls	No significant difference	7
Mol 2022	Any falls	Retrospective (self reported)	BP measurements were performed in all patients in the supine position (baseline) and after 1 and 3 min standing up using a sphygmomanometer.		OH, sBP recovery: OR=1.0 (1.0–1.0) p=0.45; dBP recovery: OR=1.0 (0.9–1.0) p=0.17 / no OH, sBP recovery: OR=1.0 (1.0–1.0) p=0.47; dBP recovery: OR=1.0 (1.0–1.0) p=0.45	No significant associations between OH and falls	No significant multivariate association	7

First author, Year	Falls outcome	Falls assessment method	Assessment method of OH	Main findings and prevalence of OH	OR/RR/HR	Conclusion	Association	NOS
Moloney 2021	Any fall	Retrospective (self reported)	Active stand test with a Finometer MIDI device, utilising BP response data up to 120 sec post-stand, at 10-sec intervals. Active stand patterns were generated based on three sequential binary sBP features: drop ≥40 mmHg within 10 sec post-stand ("immediate deficit"), failure to return to within 20 mmHg of supine level at 40 sec after standing ("stabilisation deficit") and drop ≥20 mmHg between >40 and 120 sec post-stand ("late deficit"). These three features were grouped together to defined orthostatic intolerance	28.3% of those with orthostatic intolerance experienced a fall; 19.0% of those without experienced a fall, p=0.025	Multivariate : OR=1.5 (1.2-2.1), p=0.004	There is a significant difference in the prevalence of falls among those with an without orthostatic intolerance. Further, there is a significant multivariate association between falls and orthostatic intolerance	Significant multivariate association	8
Ooi 2000	Any fall	Prospective (incident reports)	Supine and standing at 1 & 3 min., 8 measurements sphyg. 20/10	50% in fallers and non-fallers.	Unadjusted OR= 2.72 [1.92, 3.84] / OH & reoccurring falls in previous fallers RR 2.1 (1.4 - 3.1). Risk of subsequent falls was greatest in previous fallers with OH at two or more measurements, RR 2.6 (1.7 - 4.6)	OH was associated with reoccurring falls in those who had previous falls	Significant multivariate association	8
Pasma 2014	Any fall	Retrospective	(oscillometric), Supine and standing at 1 & 3 min. with sphyg & continuous, 20/10	Intermittent OH not different between fallers and non-fallers. Patients with a larger drop in BP during 15–60 seconds after standing more likely to have fallen in the past 12 months.	Continuous: OH overall (0–180 s) & falls, OR=2.5 (0.8–8.1). sBP decrease 15–60s: OR=2.0 (1.1–1.5), dBP decrease 15–60s (OR=2.1 (1.2–3.6).	Continuous OH was not associated with a history of falls. Greater dBP and sBP drop at 15–60 seconds were associated with a falls. Intermittent OH was not associated with falls.	No significant multivariate association	6

First author, Year	Falls outcome	Falls assessment method	Assessment method of OH	Main findings and prevalence of OH	OR/RR/HR	Conclusion	Association	NOS
Press 2016	Any fall	Retrospective medical record	OH assessed using oscillometric measurement- supine for 10 mins then stand and measure at 1 and 3 mins. OH if greater than 2ommhg sys or 10mmhg diastolic at 1 or 3 mins	fallers 82.6% in OH group. 82.9% in non OH group p>0.05	Unadjusted OR= 0.94 [0.62, 1.44]	No significant association between OH and falls	No significant difference	7
Rivera-Chavez 2021	Any fall,	Retrospective (self reported)	Direct interrogation and clinical history, as part of comprehensive geriatric assessment	16% of fallers had postural OH syncope; 14% of non-fallers, p>0.05	Univariate: 0R=1.1 (0.7-1.7), p>0.05	There was no significant association between falls and postural OH syncope, or significant difference in the prevalence postural OH syncope among faller and non-fallers	No significant univariate association	7
Roca 2022	Any falls	Retrospective (self reported)	Automatic blood pressure monitor with an adapted cuff in a supine position after at least 5 min of rest and after 1 and 3 minutes of standing. OH was defined as a decrease of at least 20 mmHg in sBP and/or 10 mmHg in dBP after 1 or 3 min while standing.	No OH and falls = 39.0%. OH and falls = 48.0%. OHT and falls = 36.7%, p>0.05	Unadjusted OR= 1.45 [0.93, 2.27]	No significant difference between OH and falls	No significant difference	6
Romero-Ortuno 2011	Any fall	Retrospective	Active stand for 3 min, continuous. COH: >20 sBP or 10 dBP drop. IOH: 40 sBP / 20 dBP drop < 15 seconds	Falls in those with IOH (24.7%) vs no-IOH (10.4%), p<0.001. No difference in falls between those with consensus OH	Unadjusted OR= 2.84 [1.56, 5.17]	IOH was univariately associated with a history of falls in the past 6 months	Significant univariate association	6
Rutan 1992	Any fall	Retrospective	(auscultatory), Supine and standing at 3 min., sphyg. 20/10	OH in frequent fallers: 27%, OH in non-fallers: 17%	Unadjusted OR= 1.73 [1.19, 2.51] / OR=1.5 (1.0 - 2.2)	OH was associated with a history of frequent falls in the past year	Significant multivariate association	6
Saedon 2016	Multiple falls or injurious falls	Retrospective (self reported)	Blood pressure was assessed using non-invasive BTB blood pressure using digital plethysmography	79% of fallers had OH, 57% of non-fallers	Adjusted OR= 3.6 (1.7-6.3), p=<0.001	Significant association between OH and falls	Significant multivariate association	6

First author, Year	Falls outcome	Falls assessment method	Assessment method of OH	Main findings and prevalence of OH	OR/RR/HR	Conclusion	Association	NOS
Sasidharan 2022	Any falls	Retrospective (self reported)	The Bed Side Orthostatic Test, Supine to Stand Method, was used for measuring OH. Blood pressure readings were taken after at least 5 min of rest while lying down, with appropriately sized cuffs positioned at heart levels and both arms supported using a semiautomatic validated digital sphygmomanometer.		OR=1.3 (0.5, 3.0), p>0.05	No significant association between OH and falls	No significant multivariate association	7
Schell 2021	Any fall	Retrospective (self reported)	Orthostatic vital measurement using the Welch Allyn oscillometric blood pressure monitor and OVS procedure in Lippincott Procedures	42.8% of participants had HTN.	OR=1.3 (0.8-2.2), p>0.05	No significant association between OH and falls	No significant multivariate association	7
Shaw 2015	Any falls	Retrospective (self reported)	Passive seated orthostatic stress test (PSOST). BTB arterial blood pressure was continuously recorded noninvasively using finger plethysmography applied to the middle finger of the right hand.	7.4% of fallers, had initial systolic OH, 20% of non-fallers; 11.1 had initial diastolic OH,, 15% of non-fallers; 37% had consensus systolic OH; 31.6% of non-fallers; 33.3% had consensus diastolic OH, 36.8% of non-fallers; 40.7% had delayed systolic OH, 26.3% of non-fallers; 48.2% had delayed diastolic OH, 31.6% of non-fallers	Unadjusted OR= 1.46 [0.42, 5.04]	No significant difference in falls among those with and without OH	No significant difference	5
Soysal 2016	Reoccurring falls	Retrospective	Head up tilt with BP measured at 3 minutes using sphyg	24.6 - no OH 45.6 OH P<0.001	Unadjusted OR= 2.56 [1.57, 4.17]	Significant difference between OH and falls	Significant difference	7
Subramanian 2020	Any fall	Retrospective	Self reported	10.5% of fallers had OH; 13.9% of non-fallers, p>0.05	Unadjusted OR= 0.73 [0.23, 2.31]	No significant difference in the prevalence of OH between fallers and non-fallers	No significant difference	5

First author, Year	Falls outcome	Falls assessment method	Assessment method of OH	Main findings and prevalence of OH	OR/RR/HR	Conclusion	Association	NOS
Susman 1989	Any fall	Retrospective	BP measured with mercury manometer and stethoscope. Patient supine for 5 minutes then BP taken supine and at 1 and 2 minutes of standing. OH at least a 20 mmHg systolic or 10 diastolic mmHg diastolic drop in blood pressure between the average baseline blood pressure and readings at either 1 or 2 minutes after standing	10.1% of those without OH were fallers. 19.4% with OH were fallers. (not significant) However when combined systolic and diastolic OH were considered 8/88 without OH were fallers v 5/12 with OH were fallers, p<0.01	Unadjusted OR= 2.13 [0.65, 6.95]	Significant difference between OH and falls	Significant difference when using sys and dias together	7
Tinetti 1986	Reoccurring falls	Prospective (incident reports)	Sphygmomanometer, Supine and standing at 1 & 3 min., 20 sBP.	12% (3/25) of reoccurring fallers, 0% (0/54) of single/non fallers	Unadjusted OR= 16.96 [0.84, 341.81]	OH was more prevalent in reoccurring fallers than single/non-fallers	Significant difference	5
Turusheva 2020	Any falls, injurious falls	Retrospective (self reported)	Assessed using a oscillometric sphygmomanometer. OH was diagnosed with a decrease in sBP ≥ 20 mm and/or a decrease in dBP ≥ 10 mm within 3 minutes after moving to a vertical position (standing position) from the supine position	11.1% of fallers had OH; 4.2% of non-fallers, p=>0.05	Unadjusted OR= 2.90 [0.54, 15.57]	No significant difference in OH between fallers and non-fallers	No significant difference	8
Van der Velde 2007(a)	Any fall	Retrospective	Passive (HUT) at 70°, continuous. Supine and standing at 1,2 & 3 min. with sphyg. 20/10	Sphyg OH 27% of fallers (n=33), 17% (n=12) of non-fallers. Continuous OH: 72% (n=89) of non-fallers vs 50% (n=34) of non-fallers.	Sphyg OH & falls OR=1.9 (0.8–4.4). Continuous 1-s average & falls OR=2.3 (1.1–4.7). Continuous 5 sec average & falls OR=2.5 (1.4–4.7). Unadjusted for confounders.	Continuous measured OH was associated with falls in the past year, sphyg measured OH was not.	No significant difference, significant univariate association	9

First author, Year	Falls outcome	Falls assessment method	Assessment method of OH	Main findings and prevalence of OH	OR/RR/HR	Conclusion	Association	NOS
Welmer 2020	Injurious falls	Retrospective (self reported)	OH was assessed by an auscultatory sphygmomanometer. OH was defined as a decrease in sBP of at least 20 mmHg or dBP of at least 10 mmHg, after assuming a standing position	19.7% had OH	HR=0.7 (0.4-1.4) ,p=>0.05	No significant association between OH and falls	No significant multivariate association	8
Wong 2014	Any fall	Prospective	(oscillometric), Passive (HUT), supine and at 70 deg, immediately and at 1,2,3,4,5 min, sphyg, 20/10	23% of fallers, 21% of non-fallers	Unadjusted OR= 1.15 [0.74, 1.79] / OH & falls: univariate RR= 1.1 (0.9–1.4)	OH was not associated with future falls	No significant univariate association	9
Yu 2009	Any fall	Retrospective	Not specified	21 (7.7%) of fallers had OH; 54 (4.4%) of fallers did not have OH	Unadjusted OR= 1.84 [1.09, 3.10] / OR= 1.9 (1.1-3.3) p=0.018	Significant association between OH and falls	Significant multivariate association	7
Zhao 2020	Injurious falls	Retrospective (self reported)	Not specified	27% of those with injurious falls had OH, compared to 11.3% of those without injurious falls, p=0.003	Unadjusted OR= 2.90 [1.44, 5.85] / OR=5.6 (2.1-15), p=0.001	Significant difference with regard to OH in those with injurious falls and those without	Significant multivariate association	7
Zhu 2016	Any falls	Retrospective	BP measured oscillometric. Supine for 5 minutes followed by measurement at 1 minute and 3 minutes of standing: a reduction of sBP ≥ 20 mmHg or dBP ≥ 10 mmHg = OH	10 (16.1%) of fallers had OH; 30 (9.9%) of non-fallers had OH. PR 1.6 (0.8–3.1)	Unadjusted OR= 1.74 [0.80, 3.78] / Falls with hospitalization PR= 1.31 (0.2-8.2)	No significant association between OH and falls	No significant association	7
Zia 2015	Reoccurring or injurious falls	Retrospective (self reported)	All BP measurements were obtained with an automated sphygmomanometer BP machine. A reduction in BP of 20/10 mmHg within 3 min of standing.		Multivariate: OR=1.5 (0.9-2.5), p>0.05	No significant difference between OH and falls	No significant multivariate association	9

Note. OR= odd ratio. RR: risk ratio. HR: hazard ratio. OH: orthostatic hypotension. IOH: initial orthostatic hypotension. OH40: orthostatic hypotension at 40 seconds. sBP: systolic blood pressure. dBP: diastolic blood pressure. BP: blood pressure. HTN: hypertension. NOS: Newcastle Ottawa Scale.

eTable 3B. OH and falls by measurement instrument and assessment position

Oh measurement instrument									
Beat to beat			Oscillometric sphygmomanometer			Auscultatory sphygmomanometer		Unspecified type of sphygmomanometer	
Supine To Standing	Supine To Tilted	Sitting To Standing	Supine To Standing	Supine To Tilted	Sitting To Standing	Supine To Standing	Supine To Tilted	Supine To Standing	Sitting To Standing
**Donoghue<br>**Finucane<br>**McDonald<br>**Moloney<br>**Saedon<br>**Claffey<br>*Davies<br>*Kocyigit<br>*Romero-Ortuno<br>Hartog	**Heitterachi<br>*Jodaitis<br>*Van der Velde<br>Kocyigit	Maurer	Campbell<br>Dokuzlar<br>Freud<br>Hartog<br>Magnuszewski<br>Pasma<br>Shaw<br>Subramanian<br>Turusheva<br>Juraschek<br>Kamali<br>Press<br>Roca<br>Sasidharan<br>Zhu	**Menant<br>Wong<br>*Soysal	Lawlor	**Gangavati<br>**Rutan<br>Ensrud<br>Kario<br>Mader<br>Welmer<br>Zia<br>Coutaz<br>Miu<br>Aydin	*Aydin	**Chang<br>**Graafmans<br>**Ooi<br>*Susman<br>*Tinetti<br>Campbell<br>Lipsitz<br>Liu<br>Van der Velde<br>Mol	*Bumin<br>Downton<br>Luukinen
			TOTAL: 15	TOTAL: 3	TOTAL: 1	TOTAL: 10	TOTAL: 1		
			Assessment position not specified<br>**Zhao<br>Schell<br>Ham			Assessment position not specified<br>Chan			
TOTAL: 10	TOTAL: 4	TOTAL: 1	TOTAL: 3			TOTAL: 1		TOTAL: 10	TOTAL: 3

Note. * statistically significant positive univariate association. ** statistically significant positive multivariate association. Almegbel, Areseven, Chu, Frels, (**)Magnuszewski, (**)Yu, and Rivera Chavez not included on table as OH measurement instrument and assessment position was not specified (no significant associations).

eTable 4. Low blood pressure and falls

First author, Year	Falls outcome	Falls assessment method	Assessment method of LBP	Main findings and prevalence of LBP	OR/RR/HR	Conclusion	Association	NOS
Coutinho 2008	Injurious falls	Retrospective	Retrospective, self reported	6% of fallers had LBP; 1.2% of non-fallers	Univariate OR= 5 (1.5-17.3), p=0.01	Significant multivariate association between falls and LBP	Significant univariate association	5
**Sagawa, 2018**	Injurious falls	Prospective	BP was measured by centrally trained and certified staff after 5 min of quiet rest while sitting. The average of two seated measurements was used. BP levels for sitting systolic BP diastolic BP (dBP) of ≤ 60, mmHg	dBP ≤ 60 mmHg with fall injury 125 (21.9%) and without fall injury 204 (16.3%) p<0.001	Univariate OR= 1.44 [1.12, 1.85] / dBP ≤ 60 mmHg was not significantly associated with increased risk of injurious falls HR= 1.2; (1.0–1.5).	dBP ≤ 60 mmHg increased risk of fall injury, though were attenuated by total medication use and comorbidity.	No significant multivariate association	8
**Song 2021**	Any fall	Retrospective (self reported)	Retrospective (self reported) A mercury sphygmomanometer was used to measure BP while participants were in a seated position in their home. Two measurements were taken and the average of the two values was used for analysis. Low diastolic pressure < 60 mmHg		Univariate OR= 1.06 [0.77, 1.47] / Any fall: Multivariate: OR=1.1 (0.8-1.6), p>0.05; Injurious falls: Multivariate: OR=1.1 (0.6-1.9), p>0.05	No significant association between LBP and falls, or injurious falls	No significant multivariate association	8
**Swanenburg 2010**	Multiple falls, previous 12 months, and 12 months prospectively	Retrospective and prospective	Retrospective and prospective, self report	13% of multiple fallers had LBP; 17% of non-fallers, p>0.05	Univariate OR= 0.71 [0.34, 1.49]	No significant difference in the prevalence of LBP between fallers and non-fallers	No significant difference	8

Note. OR= odd ratio. sBP: systolic blood pressure. dBP: diastolic blood pressure. LBP: low blood pressure. NOS: Newcastle Ottawa Scale.

eTable 5. Postprandial hypotension and falls

First author, Year	Falls outcome	Falls assessment method	Assessment method of PPH	Main findings and prevalence of PPH	OR/RR/HR	Conclusion	Association	NOS
Aronow 1997	Any fall	Prospective (incident reports)	Baseline BP before lunch and at 15, 30, 45, 60, 75 and 120 minutes after lunch. Participant in sitting position for at least 2 minutes before measuring.	mean maximal decrease in fallers 20 [±5]mmHg, in non-fallers 12 [±4]mmHg.	RR= 1.2 (1.2 - 1.2)	PPH is associated with future falls	Significant multivariate association	5
Le Couteur 2003	Any fall	Retrospective (incident reports)	Postprandial BP measurements at 60 min after the meal in both supine and upright position	38% of subjects had PPH.	PPH & falls OR= 1.0 (0.6–1.9), & reoccurring falls OR= 0.9 (0.4–1.9). sBP <=115 mm Hg after a meal & falls OR= 3.7 (1.3–11.1)	PPH was not associated with falls or reoccurring falls, but sBP postprandial drop below 115 mmHg was	No significant multivariate association	6
Puisieux 2000	Anny fall	Retrospective	24 hour. Recordings every 15 minutes during the day, every 30 minutes during the night.	PPH 27% in the syncope group, 18% in the fall group, 9% in the control group, p<0.05		PPH is common in patients admitted for falls and syncope	Significant difference	8
Schoon 2013	Any fall and syncope	Retrospective	10 minutes of rest, standardised fluid meal consumed within 10 mins (292 calories). Heart rate and BP continuously measured until 75 minutes after the meal.	53% of cases, 64% of controls, p>0.05		No significant difference in prevalence of PPH among fallers and controls.	No significant difference	8

Note. OR= odd ratio. RR: risk ratio. HR: hazard ratio. sBP: systolic blood pressure. dBP: diastolic blood pressure. BP: blood pressure. PPH: postprandial hypotension NOS: Newcastle Ottawa Scale.

eTable 6. Coronary artery disease and falls

First author, Year	Falls outcome	Falls assessment method	Assessment method of CAD	Main findings and prevalence of CAD	OR/RR/HR	Conclusion	Association	NOS
Abbs 2020	Any fall	Retrospective (self reported)	Self reported	12.7% of fallers had a history of MI; 7.3% of non-fallers, p=0.1	Unadjusted OR = 2.15 (1.3–3.56)	Significant association between MI and falls	Significant univariate association	7
Almegbel 2018	Any fall	Retrospective (self reported)	Self reported	Approximately 50% of non fallers had CAD; 52% of non-fallers	unadjusted OR= 1.08 (0.86, 1.35)	No significant difference in the prevalence of CAD among fallers and non-fallers , p>0.05	No significant difference	7
Arseven 2008	Non-syncopal falls	Prospective (self reported)	One or more of the following: history of myocardial infarction, heart failure, significant coronary atherosclerosis on cardiac catheterization, or angina.	35.1% of fallers had cardiac disease; 35.9% of non-fallers (p>0.05)		No significant difference in the prevalence of CAD among fallers and non-fallers, p>0.05	No significant difference	10
Banu 2018	Injurious falls	Retrospective (medical records)	Medical records	12 (25.0%) of fallers had history of ischaemic heart disease, 40 (24.7%) of non-fallers had history of ischaemic heart disease, p>0.05	unadjusted OR=1.3 (0.6–2.8) p>0.05 I found different OR 1.02 (.48-2.14)	No significant difference between prevalence of ischaemic heart disease and falls	No significant difference	8
Bhangu 2017	All falls, non-accidental falls, syncope	Retrospective (self reported)	Self reported	19.4% of participants had a fall in the previous 12 months; 5.1% had a non-accidental fall; and 4.4% had a syncopal event.	Adjusted OR .84(.64-1.11) for MI Unadjusted OR .98(.75-1.27) Falls and MI Univariate analyses: All falls: OR=1 (0.8-1.3),p=>0.05); non-accidental falls: 1.2 (0.8-1.9), p=>0.05); syncope: OR= 1.8 (1.2-2.7), p=<0.05); Adjusted analyses*: All falls: OR=1.3 (0.8-2.1),p=>.05); non-accidental falls: 0.8 (0.5-1.3), p=>0.05); syncope: OR= 1.4 (0.7-3.1), p=>0.05	Significant univariate associations between self reported history of myocardial infarction and syncope. No significant association between all falls, non-accidental falls, and myocardial infarction in univariate analyses, or all falls, non-accidental falls, syncope and self reported history of myocardial infarction in adjusted models	No significant multivariate association	8

First author, Year	Falls outcome	Falls assessment method	Assessment method of CAD	Main findings and prevalence of CAD	OR/RR/HR	Conclusion	Association	NOS
Bhangu 2017	All falls, non-accidental falls, syncope	Retrospective (self reported)	Self reported	19.4% of participants had a fall in the previous 12 months; 5.1% had a non-accidental fall; and 4.4% had a syncopal event.	Univariate analyses: All falls: OR=1.4 (1.1-1.8),p=<0.05); non-accidental falls: 2.0 (1.4-2.8), p=<0.05); syncope: OR= 2.2 (1.6-3.1), p=<0.05); Adjusted analyses: All falls: OR=1.1 (0.9-1.4),p=>.05); non-accidental falls: 1.2 (0.8-1.7), p=>0.05); syncope: OR= 1.6 (1.1-2.3), p<0.05	Significant univariate associations between self reported history of angina and all falls, non accidental falls, and syncope. Significant association between self reported history of angina and syncope in adjusted model. No significant association between all falls, non-accidental falls and self reported history of angina in adjusted models	No significant multivariate association	8
Callisaya 2014	Multiple falls, 12 months prospectively	Prospective (self reported)	Medical history	11.5% of non fallers or single fallers had a history of MI; 10.6% of multiple fallers.		No significant difference in the prevalence of MI among fallers, and non-fallers, p>0.05	No significant difference	10
Chang 2015	Any fall	Retrospective (self reported)	Self reported	20.1% of men with heart disease fell; 16.3% of men without heart disease. 30.0% of women with heart disease fell; 20.7% of women without.	Men: univariate OR= 1.2 (0.1, 1.4) Women: univariate OR=1.7 (1.4, 2.0)	Heart disease was significantly associated with falls among women, but not among men	Significant univariate association	6
Chen 2010	Any fall, any time during hospitalisation	Retrospective (reported on incident information management system)	Medical records	24.3% of reoccurring fallers had CAD; 29.7% of single fallers; 23.2% of non-fallers, p>0.05	Falls + CAD unadjusted OR 1.33 (.72-2.44)	No significant difference in the prevalence of CAD among reoccurring, single and non-fallers	No significant difference	7
Choi 2014	Any fall, previous 12 months	Retrospective (self reported)	Self reported	Non-fallers: 2,427 (7.1%), fallers 787 (8.7%) p=0.001	multivariate OR 1.1 (.99-1.22) univariate OR 1.24(1.14-1.35)	Significant difference in the prevalence of CAD among fallers and non-fallers. No significant association	No significant multivariate association	7

First author, Year	Falls outcome	Falls assessment method	Assessment method of CAD	Main findings and prevalence of CAD	OR/RR/HR	Conclusion	Association	NOS
Chu 2007	Any fall	Prospective (self reported)	Self reported		Univariate analyses showed a significant association between CAD and reoccurring falls	Univariate analyses showed a significant association between CAD and reoccurring falls. However, this was found to be non-significant in multivariate analyses	No significant multivariate association	7
Damian 2013	Any fall in the past month	Retrospective (incident reports)	Medical records, interview with physician	17% in cohort	IHD & falls RR= 0.6 (0.3 – 1.2) CAD + non severe fall adjusted OR=1.19 (0.51-2.79)CAD + severe fall adjusted OR= 0.79 (0.23-2.74)	IHD was not associated with falls	No significant multivariate association	4
Downton 1991	Any fall	Retrospective	Self reported	37.2% of fallers had IHD 31.6% of non-fallers.	unadjusted OR=1.28 [0.71, 2.30]	IHD was not associated with falls	No significant difference	6
Frankenthal 2021	Any fall	Retrospective (self reported)	Self reported	16.4% of fallers had previous MI; 13.4% of non-fallers, p=0.046	unadjusted OR=1.26 (1.01-1.58) for MI and Falls	There was a significant difference in the prevalence of MI among fallers and non-fallers	Significant difference	7
Gamage 2019	Any fall, reoccurring falls, previous 12 months	Retrospective (self reported)	Self reported previous diagnosis by physician	Prevalence of falls was 34.3%; reoccurring falls 9.6%	Falls (Crude OR=3.1 (1.5-6.5), p=0.002); reoccurring falls (Crude OR=2.8 (1.5-6.5), p=0.002)no adjust OR	Significant univariate association between falls and coronary heart disease	Significant univariate association	8
Gebre 2022	Injurious falls	Prospective (self reported)	Medical records		unadjusted HR= 1.4 (1.0–1.8), adjusted HR= 1.2 (0.9–1.6)	No significant association between ischaemic heart disease and falls	No significant association	8
George 2014	Any fall, previous three months	Retrospective (self reported)	Self reported	18% of participants had a fall in the previous three months. 25% of participants CHD had a fall over this period, compared to 16.5% of those without (p=0.016)	unadjusted OR= 1.70 [1.10, 2.62]Adjusted OR = 1.3 (0.9-2.0). P>0.05	Those with CHD were significantly more likely to have fallen, compared to those without; no significant association between coronary heart disease and falls	No significant multivariate association	6

First author, Year	Falls outcome	Falls assessment method	Assessment method of CAD	Main findings and prevalence of CAD	OR/RR/HR	Conclusion	Association	NOS
Goh 2017	Any fall	Retrospective (self reported)	Self reported	21% of participants experienced a fall in the previous year; 5.7% reoccurring falls. 4.3% of non-fallers had a previous history of Angina; 5.1% of fallers, p>0.05		No significant differences in previous history of self reported angina among this with and without a self reported fall in the previous 12 months	No significant difference	8
Goh 2017	Any fall	Retrospective (self reported)	Self reported	21% of participants experienced a fall in the previous year; 5.7% reoccurring falls. 6.8% of non-fallers had a previous history of MI; 6.6% of fallers, p>0.05	unadjusted OR for MI and Falls .98 (.56-1.7)	No significant differences in previous history of self reported MI among those with and without a self reported fall in the previous 12 months	No significant difference	8
Granek 1987	Any fall	Prospective (incident report)	Medical records	54% of fallers, and 48% of non-fallers had arteriosclerotic cardiovascular disease	OR= 1.2,(.8-1.87) p>0.05	There was no significant association between general cardiovascular disease and falls	No significant univariate association	4
Herndon 1997	In ER or admitted for falls	Retrospective	Self reported	14% of cases, 12% of controls	unadjusted OR= 1.2 (0.8–1.7)	MI was not associated with falls	No significant multivariate association	5
Hung 2017	Single fall, reoccurring falls	Retrospective (self reported)	Self reported	15.6% of non-fallers had coronary artery disease, 22.9% of single fallers, 23.1% of reoccurring fallers, p=0.02	unadjusted OR 1.6(1.1-2.3) No adjusted	Significant difference in coronary artery disease between non fallers and single fallers, and reoccurring fallers	Significant difference	6
Jansen 2015 (a)	Any fall	Retrospective	Self reported	Angina 7.1% of fallers, 5.1% of non-fallers. MI 4.5% of fallers , 4.6% of non-fallers.	Angina & falls OR= 1.1 (0.9–1.4), & reoccurring falls OR= 1.4 (1.0 -1.9). MI & falls OR= 0.8 (0.6-1.1), & reoccurring falls OR= 1.2 (0.8–1.7) MI and Falls -Unadjusted OR= .98 (75-1.2) Angina and falls OR 1.41(1.13-1.76)	MI is not associated with falls, angina is associated with reoccurring falls	Significant multivariate association	6

First author, Year	Falls outcome	Falls assessment method	Assessment method of CAD	Main findings and prevalence of CAD	OR/RR/HR	Conclusion	Association	NOS
Jonsson 1990	Reoccurring falls	Retrospective (medical records or incident reports)	Medical records	53% of fallers, and 20% of non-fallers had coronary artery (ischemic heart) disease (p=0.02)	unadjusted OR 4.4 (1.25-15	There was a significant difference in the prevalence of coronary artery disease between fallers and non-fallers	Significant difference	7
Jorgensen 2015	Injurious falls	Retrospective (incident reports)	Medical records	15.6% of fallers had a history of ischemic heart disease; 19.3% of non-fallers, p=<0.001	Univariate OR= 0.71 (0.59-0.85)	Significant difference in the prevalence of CAD among fallers and non-fallers; significant negative univariate association between CAD and falls	Significant difference; Significant negative univariate association	6
Kallin 2004	Any fall	Prospective (post fall evaluations)	Medical records	61.9% of fallers had heart disease; 55.3% of non-fallers, p>0.05	unadjusted OR 1.3 (.76-2.3) Falls and Heart disease	No significant difference in the prevalence of heart disease between fallers and non-fallers	No significant difference	6
Kang 2018	Any fall	Retrospective (self reported)	Medical history	27.6% of fallers had CAD; 22.2% of non-fallers	Univariate OR= 1.32 (0.82-2.1), p>0.05	No significant association between CAD and falls	No significant univariate association	7
Kario 2001	Any fall	Prospective, retrospective	Self reported	7.5% of the sample had a previous myocardial infarction	RR= 0.65 (0.2-1.9)	There was no significant association between previous myocardial infarction and falls	No significant univariate association	9
Koca 2020	Any fall	Retrospective (self reported)	Self reported	CHD was significantly associated with falls, p=0.02		CHD was significantly associated with falls	Significant difference	8
Ku 2013	Any fall, previous 24 months	Prospective (resident records)	Self reported	27.2% of fallers had CAD; 20.1% of non-fallers, p=0.06	Univariate OR= 1.49 (1.01-2.19), p=0.045; Multivariate OR= 1.28 (0.86-1.93), p>0.05	Significant difference in the prevalence of CAD between fallers and non-fallers; significant univariate association between CAD and falls; no significant multivariate association between CAD and falls	No significant multivariate association	9
Lawlor 2003	Any fall	Retrospective	Self reported and medical records	23% of fallers, 14% of non-fallers	Falls and CAD unadjusted OR= 1.82 (1.47-2.2)adjusted OR= 1.5(1.16-1.95)	CAD was associated with falls	Significant multivariate association	4

First author, Year	Falls outcome	Falls assessment method	Assessment method of CAD	Main findings and prevalence of CAD	OR/RR/HR	Conclusion	Association	NOS
Lee 2009	Reoccurring falls	Retrospective	Self reported	23% of patients who had a fall had CAD compared to 16% of the overall population		CAD was more prevalent in fallers compared to non fallers	Significant difference	4
Lipsitz 1991	Any fall	Prospective (computer records and review of incidence reports)	Clinical evaluation	44% of fallers had ischemic heart disease; 36% of non-fallers, p>0.05	Falls and IHD OR= 1.42(.96-2.9)	No significant difference in the prevalence of ischemic heart disease between fallers and non-fallers	No significant difference	8
Magnuszewski 2020	Any fall	Retrospective (self reported)	Self reported	55.4% of fallers had Ischemic heart disease; 53.7% of non-fallers, p>0.05	IHD and Falls unadjusted OR= 1.07(.7-1.6)	No significant difference in the prevalence of CAD between fallers and non-fallers	No significant difference	8
Magnuszewski 2020	Any fall	Retrospective (self reported)	Self reported	10.8% of fallers had history of MI; 9.5% of non-fallers, p>0.05	MI and falls Unadjusted OR= 1.16(.58-2.3)	No significant difference in the prevalence of MI between fallers and non-fallers	No significant difference	8
Magnuszewski 2022	8 months	Prospective (incident reports)	Medical records	Fallers with ischemic heart disease 71.4%, non fallers with ischemic heart disease 53.0% p=0.13	Falls and IHD unadjusted O=R 2.21 (.68-7.19)	No significant difference in the prevalence of ischemic heart disease between fallers and non-fallers	No significant difference	8
Menant 2016	Unexplained falls	Prospective	Self reported	10.5% of non-fallers had previous myocardial infarction; 5.7% of unexplained fallers	unadjusted OR prior MI and falls OR= .52(.12-2.25)	No significant difference in previous myocardial infarction between fallers and non-fallers; no significant association between myocardial infarction and falls	No significant multivariate association	10
Mitchell 2013	Any fall	Retrospective	Self reported	Heart disease/angina 30% of fallers, 24% of non fallers, poor circulation in legs/peripheral vascular disease 28.1% of fallers, 17.4% of non-fallers.	Heart disease and Falls unadjusted OR 1.33 (1.12-1.57)	Circulatory disease was associated with falls	Significant multivariate association	6

First author, Year	Falls outcome	Falls assessment method	Assessment method of CAD	Main findings and prevalence of CAD	OR/RR/HR	Conclusion	Association	NOS
Mitchell 2015	Any fall	Retrospective (self reported)	Self reported		RR=1.1 (1-1.1), p=0.001	Significant association between coronary heart disease and falls	Significant multivariate association	8
Paliwal 2017	Any fall	Retrospective (self reported)	Self reported	15.2% of fallers had angina, 10.6% of non-fallers	Crude OR = 1.49 (1.4-1.6), p=<0.05; Adjusted OR = 1.2 (1.1-1.3), p=<0.05	No significant differences reported, significant association between falls and angina	Significant multivariate association	8
Paliwal 2017	Any fall	Retrospective (self reported)	Medical records	14.4% of fallers had myocardial infarction; 10.1% of non-fallers	Crude OR = 1.48 (1.4-1.6), p=<0.05	No significant differences reported with regard to myocardial infarction in those with and without falls. Significant association between falls and myocardial infarction	Significant univariate association	8
Rafiq 2014	Any fall	Retrospective	Medical records	IHD 15%, CAD 5%, MI 4%. IHD & falls	IHD and falls unadjusted OR= 1.16 (1.11–1.21) adjusted OR 1.2(1.1-1.2)	IHD was independently associated with falls; CHF, CAD and MI were not	Significant multivariate association	7
Rivera-Chavez 2021	Any fall	Retrospective (self reported)	Direct interrogation and clinical history, as part of direct geriatric assessment	16% of fallers had a history of myocardial infarction; 15% of non-fallers, p>0.05	Univariate: 0R=1.05 (0.68-1.58), p>0.05	There was no significant association between falls and history of previous MI, or significant difference in the prevalence of previous MI among fallers and non-fallers	No significant univariate association	7
Saedon 2016	Multiple falls or injurious falls	Retrospective (self reported)	Self reported	10.3% of fallers had a history of myocardial infarction; 6.3% of non-fallers, p>0.05	MI and Falls unadjusted OR 1.7(0.68-4.3)	No significant difference in history of myocardial infarction between fallers and non-fallers	No significant difference	6
Shaw 2015	Any falls	Retrospective (self reported)	Medical records	19.2% of fallers had atherosclerotic heart disease, 0% of non-fillers, p=0.058	Falls and atherosclerotic heart disease unadjusted OR 10.4(.54-201.9)	No significant difference in heart disease between fallers and non-fallers	No significant difference	5
Sibley 2014	Any fall	Retrospective	Self reported	24% of those with heart disease fell, compared to 19% of those without heart disease	unadjusted OR 1.42 [1.30, 1.55] Adjusted OR= 1.3, p>0.05	Cluster 'heart disease' was not significantly associated with falls	No significant multivariate association	6
Stenhagen 2013	Any fall	Retrospective	Medical records (ICD codes)	Heart disease (including angina, MI and arrhythmia) in 23% of fallers, 77% of non fallers.	Heart disease and falls unadjusted OR 1.7(1.28-2.24) adjusted OR 1.36 (1-1.83)	Heart disease was associated with future falls	Significant multivariate association	7

First author, Year	Falls outcome	Falls assessment method	Assessment method of CAD	Main findings and prevalence of CAD	OR/RR/HR	Conclusion	Association	NOS
Subramanian 2020	Any fall, reporting interval not reported	Retrospective	Self reported	21% of fallers had CAD; 13.1% of non-fallers, p>0.05	CAD and falls unadjusted OR= 1.76 (.68-4.5)	No significant difference in the prevalence of CAD between fallers and non-fallers	No significant difference	5
Svensson 1992	Any falls	Retrospective (self reported) interview	Self reported	72 (24.0%) of fallers had CAD 91 (21.0%) v non fallers - not significant. 36 (12.0%) of fallers had angina v 11% non fallers - not significant. 47 (6.0%) of fallers had a prior MI v 8% non fallers- not significant	MI and falls Unadjusted OR = .742(.41-1.34) Coronary insufficiency and falls OR= 1.17(0.82-1.66) Angina and falls OR =1.1 (0.69-1.7)	No significant difference in heart diseases (CAD/ MI/angina) between fallers and non-fallers. No significant association between heart disease and falls	No significant difference	7
Teoh 2021	Any fall	Retrospective (self reported)	Self reported	7.5% of non fallers had a previous MI; 6.8% of fallers, p>0.05	unadjusted OR= 0.71 [0.29, 1.73]	There was no significant difference in the prevalence of previous MI among fallers and non-fallers	No significant difference	8
Thapa 2022	Any falls	Medical records	Medical records	3 (2.0%) of fallers had history of myocardial infarction, 49 (3.1%) of non-fallers did, p>0.05	MI and falls unadjusted OR= 0.63(0.19-2.05)	No significant difference in the prevalence of myocardial infarction between fallers an non-fallers	No significant difference	6
Tsai 2021	Any fall	Retrospective (self reported) interview	Self reported	520 (9.9% of people with heart attack fell, 4,716 (90.1%) of people with heart attach did not fall. Unadjusted OR= 1.5 (1.2-1.9) p<0.001	Mi and falls Unadjusted OR= 1.52(1.23-1.87) adjusted OR= 1.1 (0.9-1.4) p>0.05	No significant association between heart attacks and falls	No significant association	6
Turusheva 2020	Any falls, injurious falls	Retrospective (self reported)	Self reported	16.7% of fallers had myocardial infarction; 15.9% of non fallers, p=>0.05	MI and falls unadjusted OR = 1.06 (0.28-3.96)	No significant difference in history of myocardial infarction between fallers and non-fallers	No significant difference	8

First author, Year	Falls outcome	Falls assessment method	Assessment method of CAD	Main findings and prevalence of CAD	OR/RR/HR	Conclusion	Association	NOS
Valderrama-Hinds 2018	Any fall, previous 24 months	Retrospective (self reported)	Self reported	3.4% of fallers had MI; 3% of non-fallers, p>0.05	MI and falls unadjusted OR =1.25(0.97-1.61) multivariate OR= 0.99 (0.8.-1.2)	No significant difference in the prevalence of MI between fallers and non-fallers; no significant association between falls and MI	No significant univariate association	6
von Heideken Wågert 2009	Any fall	Prospective (falls diaries, phone calls, medical records)	Medical records and self report	Cardiac insufficiency was present in 26% of fallers and 24% of non fallers, p>0.05	cardiac insufficiency and falls Unadjusted OR= 1.13(0.61-2.09)	No significant difference between cardiac insufficiency and falls	No significant difference	10
Wong 2014	Any fall	Prospective	Self reported	MI in 10% of fallers, 9% of non fallers. MI & falls unadjusted	Mi and falls unadjusted OR = 1.07 (0.57-2.02) RR= 1.0 (0.7–1.5)	MI was not associated with future falls	No significant multivariate association	9
Xu 2015	Any fall	Prospective	Self reported	79.3% of fallers had CAD; 66.7% of non-fallers, p<0.05	CAD and falls unadjusted OR= 1.91(1.14-3.1)Multivariate RR= 1.6 (1-2.5), p>0.05	Significant difference in the prevalence of CAD between fallers and non-fallers; no significant multivariate association between falls and previous CAD	No significant multivariate association	10
Yasumura 1994	Any fall	Retrospective (self reported)	Self reported		Men: Multivariate OR= 1.4 (1.0-2.0); Women: Multivariate OR= 1.3 (1.0-1.7)	No significant association between CAD and falls	No significant multivariate association	7
Yi 2021	Any fall	Retrospective (self reported)	Self reported		Men: Multivariate OR= 1.2 (0.97–1.4), p<0.1; Women: Multivariate OR= 1.4 (1.2–1.7), p<0.001	Significant multivariate association between MI and falls among women, not among men	Significant multivariate association (women)	8
Zhao 2019	Any falls	Retrospective (self reported)	Self reported	32.4% of participants had heart disease. 30.7% of fallers had a history of heart disease; 33% of non-fallers, p>0.05	adjusted OR=0.65 (0.43-0.98). P=0.05	No significant difference in heart disease between fallers and non-fallers. No significant association between heart disease and falls	No significant multivariate association	7

Note. OR= odd ratio. RR: risk ratio. HR: hazard ratio. CAD: coronary artery disease. CHD: chronic heart disease. CHF: chronic heart failure. MI: myocardial infarction. IHD: ischemic heart disease. NOS: Newcastle Ottawa Scale.

eTable 7. Heart failure and falls

First author, Year	Falls outcome	Falls assessment method	Assessment method of heart failure	Main findings and prevalence of heart failure	OR/RR/HR	Conclusion	Association	NOS
Abbs 2020	Any fall	Retrospective (self reported)	Self reported	6.8% of fallers had a history of congestive heart failure; 7.3% of non-fallers, p=0.85	Unadjusted OR = 1.7 (1.0–2.9)	No significant association between falls and congestive heart failure	No significant multivariate association	7
Bhangu 2017	All falls, non-accidental falls, syncope	Retrospective (self reported)	Self reported	19.4% of participants had a fall in the previous 12 months; 5.1% had a non-accidental fall; and 4.4% had a syncopal event.	Univariate analyses: All falls: OR=1.6 (1.0-2.6),p=<0.05); non-accidental falls: 2.5 (1.3-4.8), p=<0.05); syncope: OR= 2.2 (1.0-4.5), p=<0.05); Adjusted analyses: All falls: OR=1.3 (0.8-2.1),p=>0.05); non-accidental falls: 1.4 (0.7-2.8), p=>0.05); syncope: OR= 1.4 (0.7-3.1), p=>0.05	Significant univariate associations between self reported history of heart failure and all falls, non accidental falls, and syncope. No significant association between all falls, non-accidental falls, syncope and self reported history of heart failure in adjusted models	No significant multivariate association	8
Chen 2010	Any fall, any time during hospitalisation	Retrospective (reported on incident information management system)	Medical records	11.4% of reoccurring fallers had hypertension; 13.8% of single fallers; 17.4% non-fallers, p>0.05	unadjusted OR=0.73 [0.36, 1.46]	No significant difference in the prevalence of heart failure among reoccurring, single, and non-fallers	No significant difference	7
Damian 2013	Any fall in the past month	Retrospective (incident report)	Medical chart, interview with physician	20% in cohort	RR= 2.2 (1.2- 4.0)	HF was associated with a fall in the past month	Significant multivariate association	4
Granek 1987	Any fall	Prospective (incident reports)	Medical records	34% of fallers, and 26% of non-fallers had heart failure	unadjusted OR=1.44 [0.92, 2.26], p=>0.05	There was no significant association between heart failure and falls	No significant univariate association	4
Heckenbach 2014	Any fall	Retrospective (Medical records, ICD-10 codes)	Medical records (GP, ICD code)	19% of fallers, 9% of non-fallers.	adjusted OR= 1.7 (1.3–2.3) unadjusted OR=2.41 [1.85, 3.13]	HF was associated with previous falls	Significant multivariate association	6
Hussain 2022	Any fall	Any fall	Medical records	1.7% of fallers, and 0.5% of non-fallers had previously been hospitalised for heart failure, p=<0.05	unadjusted OR= 3.46 [2.21, 5.42]	There was a significant difference in the prevalence hospitalisation for heart disease between fallers and non-fallers	Significant difference	8

First author, Year	Falls outcome	Falls assessment method	Assessment method of heart failure	Main findings and prevalence of heart failure	OR/RR/HR	Conclusion	Association	NOS
Jansen 2015 (a)	Any fall	Retrospective (Medical records - ICD 10 codes)	Self reported doctor-diagnosed	1.6% of fallers, 0.9% of non-fallers	unadjusted OR=1.69 [1.06, 2.69]HF & falls Adjusted OR= 1.38 (0.85-2.23). HF & reoccurring falls OR= 1.5 (1.0–2.1)	HF was associated with falls and reoccurring falls	Significant multivariate association	6
Jonsson 1990	Reoccurring falls	Retrospective (medical records or incident reports)	Medical records	40% of fallers, and 25% of non-fallers had heart failure disease (p=>0.05)	unadjusted OR=1.96 [0.59, 6.52]	There was no significant difference in the prevalence of heart failure between fallers and non-fallers	No significant difference	7
Jorgensen 2015	Injurious falls	Retrospective (incident reports)	Medical records	19.4% of fallers had a history of heart failure; 21.6% of non-fallers, p=0.001	unadjusted OR = 0.88 [0.81, 0.95]	Significant difference in the prevalence of heart failure among fallers and non-fallers; no significant association between heart failure and falls	No significant univariate association	6
Just 2021	Any fall	Prospective and retrospective (self reported)	Self reported	13.7% of non-fallers had heart failure; 16.9% of fallers, p>0.05; 23% of multiple fallers (p=0.002)	unadjusted OR=1.28 [0.94, 1.75]	There was no significant difference in the prevalence of heart failure between fallers and non-fallers. There was a significant difference in the prevalence of heart failure between non-fallers and multiple fallers	Significant difference	8
Lee 2020	Any fall	Retrospective (self reported history of falls)	Self reported	Not reported	Adjusted; OR= 1.1 (1.0-1.3), p=<0.05 unadjusted OR = 1.46 [1.31, 1.63]	Significant association between falls and heart failure in unadjusted and adjusted analyses	Significant multivariate association	8
Lipsitz 1991	Any fall	Prospective (computer records and review of incidence reports)	Clinical evaluation	37% of fallers had heart failure; 34% of non-fallers, p>0.05	Unadjusted OR= 1.15 [0.55, 2.40]	No significant difference in the prevalence of heart failure between fallers and non-fallers	No significant difference	8
Magnuszewski 2020	Any fall	Retrospective (self reported)	Self reported	42% fallers had history of MI; 34.3% of non-fallers, p=0.14	unadjusted OR = 1.39 [0.90, 2.13] Multivariate OR= 1.7 (0.9-3.1), p>0.05	No significant difference in the prevalence of heart failure between fallers and non-fallers	No significant multivariate association	8
Magnuszewski 2022	8 months	Prospective (incident reports)	Medical records	100.0% fallers had history of chronic heart failure, 90.6% of non-fallers did, p>0.05	Unadjusted OR = 3.06 [0.18, 52.35]	No significant difference between prevalence of chronic heart failure between fallers and non-fallers	No significant difference	8

First author, Year	Falls outcome	Falls assessment method	Assessment method of heart failure	Main findings and prevalence of heart failure	OR/RR/HR	Conclusion	Association	NOS
Oren 2022	Any falls	Retrospective (medical records)	Medical records	190 (73.9%) of fallers had congestive heart failure, 69 (15.7%) of controls had congestive heart failure	Unadjusted OR= 15.2 (10.4–22.2) p<0.001 - Adjusted OR= 18.9 (10.7–33.3) P<0.001	Congestive heart failure was associated with falls	Significant multivariate association	7
Rafiq 2014	Any fall	Retrospective	Medical chart, GP charts,	4% in whole cohort	unadjusted OR=3.06 [0.18, 52.35]	HF was not associated with falls	No significant difference	7
Rivera-Chavez 2021	Any fall	Retrospective (self reported)	Direct interrogation and clinical history, as part of direct geriatric assessment	16% of fallers had atrial fibrillation; 17% of non-fallers, p>0.05	unadjusted OR=1.0 (0.7-1.5), p>0.05	There was no significant association between falls and heart failure, or significant difference in the prevalence of heart failure among fallers and non-fallers	No significant univariate association	7
Saedon 2016	Multiple falls or injurious falls	Retrospective (self reported)	Self reported	3.2% of fallers have a history of heart failure; 3.5% of non-fallers, p>0.05	unadjusted OR=0.90 [0.24, 3.43]	No significant difference in history of heart failure among fallers and non-fallers	No significant difference	6
Shaw 2015	Any falls	Retrospective (self reported)	Medical records	19.2% of fallers had heart failure 10% of non-fallers, p>0.05	unadjusted OR=2.14 [0.37, 12.41]	No significant difference with regard previous history of heart failure among fallers and non-fallers	No significant difference	5
Stenhagen 2013	Any falls	Retrospective	Medical chart, ICD-10, examination by a physician	11% of fallers and 4% of non-fallers.	unadjusted OR= 2.81 [1.78, 4.42]adjusted OR= 1.9 (1.2–3.0)	HF was associated with future falls	Significant multivariate association	7
Thapa 2022	Any falls	Medical records	Medical records	30 (19.6%) of fallers had history of chronic heart failure, 264 (16.5%) of fallers did, p>0.05	unadjusted OR= 1.23 [0.81, 1.87]	No significant difference between prevalence of CHF between fallers and non-fallers	No significant difference	6
Xu 2015	Any fall	Prospective	Self reported	6.3% of fallers had atrioventricular block; 6.8% of non-fallers, p>0.05	unadjusted OR = 0.92 [0.38, 2.20]	No significant difference in the prevalence of heart failure between fallers and non-fallers; no significant multivariate association between falls and heart failure	No significant multivariate association	10

Note. OR= odd ratio. RR: risk ratio. HR: hazard ratio. CHF: chronic heart failure. HF: heart failure. NOS: Newcastle Ottawa Scale.

eTable 8. Arrhythmia and falls

First author, Year	Falls outcome	Falls assessment method	Assessment method of arrhythmia	Main findings and prevalence of CA	OR/RR/HR	Conclusion	Association	NOS
Bhangu 2017	All falls, non-accidental falls, syncope	Retrospective (self reported)	Self reported	19.4% of participants had a fall in the previous 12 months; 5.1% had a non-accidental fall; and 4.4% had a syncopal event.		Significant univariate associations between self reported history of arrhythmia and all falls, non-accidental falls, and syncope. Also significant association between all falls, syncope and arrhythmia in adjusted model. No significant association between non-accidental and self reported history of arrhythmia in adjusted model.	Significant multivariate association	8
Chen 2010	Any fall, any time during hospitalisation	Retrospective (reported on incident information management system)	Medical records	20.0% of reoccurring fallers had hypertension; 14.1% of single fallers; 20.3% non-fallers, p=0.3	AF and Falls unadjusted OR= 0.71 [0.37, 1.37]	No significant difference in the prevalence of atrial fibrillation among reoccurring, single, and non-fallers	No significant difference	7
Damian 2013	Any fall in the past month	Retrospective (incident reports)	Medical chart, interview with physician	Arrhythmias in 22.3% of fallers	Arrhythmias RR=3.4 (1.8–6.3)	Medical history of arrhythmia was associated with a fall in the past month	Significant multivariate association	4
Davison 2005	reoccurring falls in the past 12 months	Retrospective	24-hour ambulatory ECG recorder.	One or more ECG abnormalities were identified in 49% of fallers and 41% of controls. No causative arrhythmias were identified.	Unadjusted OR = 1.82 [0.54, 6.08]Any ECG abnormality & falls: RR=1.2 (0.9–1.6).	No causative arrhythmias identified in reoccurring fallers compared to controls without a history of falls.	No significant association	7
Granek 1987	Any fall	Prospective (incident report)	Medical records	18% of fallers, and 14% of non-fallers had an arrhythmia	Unadjusted OR =1.44 [0.82, 2.53]	There was no significant association between arrhythmia and falls	No significant univariate association	4
Homer 2021	Insurance claims related to falls, 24 months prospectively	Insurance claims	Medical records	10.3% of the sample had at least one fall within the follow up period	Unadjusted OR= 2.1 (2.0-2.2)	There was a significant univariate association between cardiac dysrhythmias and falls	Significant univariate association	6
Hung 2013	Any fall	Retrospective	12-lead ECG, Telemetry, Medical chart history	AF 20% of fallers, 11% of non-fallers, p 0.029	unadjusted OR= 2.10 [2.00, 2.20]	AF was independently associated with history of falls.	Significant multivariate association	5
Jansen 2015 (b)	Any fall	Retrospective	ECG	AF 3.6% in fallers, 2.1% in non-fallers	unadjusted OR= 1.72 [1.15, 2.58] AF & any fall. adjusted x. Age 65–74: OR= 2.0 (1.0–4.1)	AF is associated with any fall in the past year in those aged 65–74, but not in the overall age group	Significant multivariate association	6

First author, Year	Falls outcome	Falls assessment method	Assessment method of arrhythmia	Main findings and prevalence of CA	OR/RR/HR	Conclusion	Association	NOS
Koca 2020	Any fall	Retrospective (self reported)	Electrocardiogram	52% had atrial fibrillation. Falls within the last year were significantly higher in the AF cohort, p=0.016		Self reported falls in the previous 12 months were significantly higher those with atrial fibrillation compared to those without	Significant difference	8
Lipsitz 1991	Any fall	Prospective (computer records and review of incidence reports)	Clinical evaluation	20% of fallers had atrial fibrillation or a flutter; 19% of non-fallers, p>0.05	unadjusted OR= 0.95 [0.38, 2.40]	No significant difference in the prevalence of atrial fibrillation or flutter between fallers and non-fallers	No significant difference	8
Magnuszewski 2020	Any fall	Retrospective (self reported)	Self reported	22.9% of fallers had atrial fibrillation; 22.9% of non-fallers, p>0.05	unadjusted OR= 1.00 [0.61, 1.65]	No significant difference in the prevalence of atrial fibrillation between fallers and non-fallers	No significant difference	8
Magnuszewski 2022	8 months	Prospective (incident reports)	Medical records	21.4% of fallers had atrial fibrillation, 23.6% of non-fallers did p>0.05	unadjusted OR= 0.88 [0.24, 3.22]	No significant difference in the prevalence of atrial fibrillation between fallers and non-fallers.	No significant difference	8
O'Neal 2015	Multiple falls	Retrospective (self reported)	Self reported	8.3% of participants had atrial fibrillation, 6.7% of participants had falls	unadjusted OR = 1.66 [1.43, 1.94]adjusted OR=1.2 (1-1.4), p=0.02	Significant association between atrial fibrillation and falls	Significant univariate association	8
Rivera-Chavez 2021	Any fall, unspecified	Retrospective (self reported)	Direct interrogation and clinical history, as part of direct geriatric assessment	8% of fallers had atrial fibrillation; 5% of non-fallers (p=0.24)	Univariate: 0R=1.4 (0.8-2.7), p=0.24	There was no significant association between falls and atrial fibrillation, or significant difference in the prevalence of atrial fibrillation among fallers and non-fallers	No significant univariate association	7
Rosado 1989	Any fall	Retrospective (incident reports)	Holter monitoring	82% ventricular arrhythmias in both groups, 100% supraventricular arrhythmias in both groups.	ventricular arrhythmia and falls unadjusted OR= 1.06 [0.32, 3.55]	Cardiac arrhythmia was not more prevalent in those who had falls	No significant difference	8
Saedon 2016	Multiple falls or injurious falls	Retrospective (self reported)	Self reported	1.9% of fallers had a self reported history of atrial fibrillation; 1.8% of non-fallers, p>0.05	unadjusted OR= 1.09 [0.18, 6.61]	No significant difference in history of atrial fibrillation among fallers and non-fallers	No significant difference	6

First author, Year	Falls outcome	Falls assessment method	Assessment method of arrhythmia	Main findings and prevalence of CA	OR/RR/HR	Conclusion	Association	NOS
Sanders 2012	Unexplained falls and accidental falls	Retrospective	12-lead ECG, medical history (chart review)	26% of non- accidental fallers had a medical history of AF, compared to 15% of those with accidental falls	History of AF & non accidental falls Log OR= 1.2 [1.0–2.7] compared to non-accidental falls. Objectified AF not associated with falls	AF is associated with non-accidental (unexplained) falls compared to accidental falls	Significant multivariate association	5
Tapha 2022	Any falls	Medical records	Medical records	37 (24.2%) of fallers had arrhythmia, 432 (27.1%) of non-fallers did, p>0.05	unadjusted OR= 0.86 [0.58, 1.26]	There was no significant difference between arrhythmias and falls	No significant difference	6
Wallace 2017	Any fall	Retrospective (self reported)	Medical records		Adjusted HR=1 (0.9-1.1), p=>0.05	No significant association between atrial fibrillation and falls	No significant multivariate association	8
Xu 2015	Any fall	Prospective	Self reported medical history	16.2% of fallers had sinus brachycardia; 10.7% of non-fallers, p>0.05	unadjusted OR= 1.06 [0.59, 1.90] Multivariate RR= 1.04 (0.6-1.7), p>0.05	No significant difference in the prevalence of sinus brachycardia between fallers and non-fallers; no significant multivariate association between falls and sinus bracycardia	No significant multivariate association	10
Xu 2015	Any fall	Prospective	Self reported medical history	10.8% of fallers had atrial fibrillation; 10.7% of non-fallers, p>0.05	unadjusted OR= 1.01 [0.51, 2.02] Multivariate RR= 1.02 (0.6-1.9), p>0.05	No significant difference in the prevalence of atrial fibrillation between fallers and non-fallers; no significant multivariate association between falls and atrial fibrillation	No significant multivariate association	10
Xu 2015	Any fall	Prospective	Self reported medical history	18.9% of fallers had atrioventricular block; 17.3% of non-fallers, p>0.05	unadjusted OR=1.12 [0.64, 1.94]Multivariate RR= 1.01 (0.6-1.6), p>0.05	No significant difference in the prevalence of atrioventricular block between fallers and non-fallers; no significant multivariate association between falls and atrioventricular block	No significant multivariate association	10

Note. OR= odd ratio. RR: risk ratio. HR: hazard ratio. AF: atrial fibrillation. NOS: Newcastle Ottawa Scale.

eTable 9. Valvular heart disease and falls

First author, Year	Falls outcome	Falls assessment method	Assessment method and definition of disorder	Main findings and prevalence of disorder	OR/RR/HR	Conclusion	Association	NOS
Bhangu 2017	All falls, non-accidental falls, syncope	Retrospective (self reported)	Self reported. Heart murmur	19.4% of participants had a fall in the previous 12 months; 5.1% had a non-accidental fall; and 4.4% had a syncopal event.	Univariate analyses: All falls: OR=1.6 (1.3-2.0), p=<0.05 ; non-accidental falls: OR= 1.5 (1.0-2.2), p=>0.05; syncope: OR= 2.0 (1.4-2.9), p=<0.05; Adjusted analyses*: All falls: OR=1.4 (1.1-1.7), p=<0.05; non-accidental falls: OR=1.1 (0.7-1.7), p=>0.05; syncope: OR=1.7 (1.2-2.5), p=<0.05	Significant univariate associations between self reported history of heart murmur and all falls and syncope. Also significant association between all falls, and syncope and heart murmur in adjusted model. No significant association between non-accidental falls and self report history of heart murmur in univariate or adjusted model.	Significant multivariate association (except non-accidental falls)	8
Van der Velde 2007(b)	Any fall	Retrospective	Echocardiography. Aortic valve stenosis, aortic valve regurgitation, mitral valve regurgitation, tricuspid valve regurgitation, pulmonary valve regurgitation, pulmonary hypertension, LV hypertrophy (septum >12mm), LVEF <40%	AVS: 7% fallers, 10% non-fallers. AVR: 29% fallers, 24% non-fallers. MVR: 43% fallers, 29% non-fallers. TVR: fallers 67%, non-fallers 37%*. PVR fallers 47%, non- fallers 29%*. *Significant p<0.05	PVR and Falls: Unadjusted OR =2.07 [1.10, 3.86]. TR and Falls: Unadjusted OR =3.21 [1.70, 6.09]. MVR and Falls: Unadjusted OR =1.84 [0.98, 3.46]. AR and Falls :Unadjusted OR =1.23 [0.62, 2.44 .] AVS and falls: Unadjusted OR =0.69 [0.22, 2.15] AVS: HR=0.6 (0.2–1.8), AVR: HR= 1.6 (0.9–2.9), MVR: HR= 1.7 (1.0–2.9)*, TVR: HR= 2.4 (1.3–4.4), *PVR: HR= 1.8 (1.0–3.0)*. *Significant p<0.05	Mitral, tricuspid and pulmonary valve regurgitation were associated with falls	Significant multivariate association	9

Note. OR= odd ratio. RR: risk ratio. HR: hazard ratio. AVS: aortic valve stenosis. AVR: aortic valve regurgitation. MVR: mitral valve regurgitation. TVR: tricuspid valve regurgitation. PVR: pulmonary valve regurgitation. NOS: Newcastle Ottawa Scale.

eTable 10. Carotid sinus hypersensitivity and falls

First author, Year	Falls outcome	Falls assessment method	Assessment method and definition of disorder	Main findings and prevalence of CSH	OR/RR/HR	Conclusion	Association	NOS
Anpalahan 2012	Referred for unexplained and accidental falls	Retrospective	CSM L+R, supine & upright (70°). vasodepressor drop of 50mmHG sBP and/or >3 second asystole on ECG	19% of unexplained fallers had CSS (2 CI, 2 VD), 0% of accidental fallers.	CSH and VVS combined OR= 5.3 (0.6–10.4), p=0.05	Neurally mediated syncope (CSS or VVS) was not associated with unexplained falls when compared to accidental falls	No significant multivariate association	6
Davies 2001	In ED for unexplained (non-accidental) or accidental falls	Retrospective	CSM L+R, supine & upright (70°). vasodepressor drop of 50mmHG sBP and/or >3 second asystole on ECG	CI CSS: 46% (12/26) cases, 13% (7/54) of controls, p< .05. VD CSS: 69% (18/26) cases, 22% (16/54) controls, p<0.05.	unadjusted OR= 3.07 [0.98, 9.59]	CSS was more prevalent in non-accidental fallers than accidental fallers and other controls	Significant difference	6
Kumar 2003	Referred for falls	Retrospective	CSM L+R, supine & upright (70°). vasodepressor drop of 50mmHG sBP and/or >3 second asystole on ECG	Prevalence of CSS in fallers was 17% and 0% in asymptomatic controls, p<0.05		CSS was more prevalent in fallers compared to asymptomatic controls	Significant difference	7
Murphy 1986	Any fall	Prospective (incident reports)	CSM L+R, supine & upright (70°). Vasodepressor drop of 50mmHG sBP and/or >3 second asystole on ECG	Prevalence of CI CSH was 11% in fallers and 21% in non fallers, difference n/s. VD CSS not measured.	CSH and Falls unadjusted OR= 0.46 [0.14, 1.47]	CI CSH was not associated with future falls	No significant difference	7
Schoon 2013	Any fall and syncope	Retrospective	10 min. active stand, continuous BP. Three head movements (rotation right, left and hyperextension). sBP calculated as mean of three beats with lowest sBP during the HTT. HTT defined as a drop in sBP of ≥20 mmHg. mean sBP drop 36 mm Hg	39% of cases had HTIH, compared to 44% of controls, p>0.05		HTIH is not different between those presenting with falls and syncope compared to healthy controls	No significant difference	8

Note. OR= odd ratio. RR: risk ratio. HR: hazard ratio. CI: cardioinhibitory. VD: vasodepressor. CSM: carotid sinus massage. CSH: carotid sinus hypersensitivity. CSS: carotid sinus syndrome. HTIH: head turning-induced hypotension. HTT: head turning test. NOS: Newcastle Ottawa Scale.

eTable 11. Vasovagal syncope and falls

First author, Year	Falls outcome	Falls assessment method	Assessment method of vasovagal syncope	Main findings and prevalence of VVS	Conclusion	Association	NOS
Anpalahan 2012	Unexplained and accidental falls	Retrospective	TTT 40mins with continuous monitoring	5% of unexplained fallers had VVS, vs 0% of accidental fallers	VVS was more common in unexplained fallers compared with accidental fallers	No significant difference	6

Note. VVS: vasovagal syncope. TTT: tilt table testing. NOS: Newcastle Ottawa Scale.

eTable 12. Stroke/Transient ischemic attack and falls

First author, Year	Falls outcome	Falls assessment method	Assessment method of stroke	Main findings and prevalence of stroke	OR/RR/HR	Conclusion	Association	NOS
Abbs 2020	Any fall	Retrospective (self reported)	Self reported	21.4% of fallers had a history of stroke; 6.0% of non-fallers, p<0.01)	Unadjusted OR = 3.4 (2.1–5.5); Adjusted OR = 2.2 (1.4-3.3)	Significant difference in stroke among fallers and non-fallers; significant positive association between falls and stroke in both univariate and multivariate analyses	Significant multivariate association	7
Akande-Sholabi 2020	Any fall	Retrospective (self reported)	Self reported	45.3% of participants fell. 3.9% of fallers reported a previous stroke; compared to 2.7% of non-fallers.	Unadjusted OR= 1.43 [0.47, 4.33]	No significant difference reported between falls in the past 12 months and previous history of stroke	No significant difference	6
Almegbel 2018	Any fall	Retrospective (self reported)	Self reported	Approximately 75% of fallers had a stroke / transient ischemic attack; 50% of non-fallers	Unadjusted OR = 2.75 [1.18, 6.41] / OR= 2.8 (1.2- 6.4)	Significant univariate association between CVA and falls	Significant univariate association	7
Arseven 2008	Non-syncopal falls	Prospective (self reported)	Patient report, a primary care physician questionnaire, and medical record review, including laboratory results and medications	24.3% of fallers had history of stroke; 9.9% of non-fallers, p=0.022	Unadjusted OR= 2.92 [1.13, 7.50]	Significant difference in the prevalence of history of stroke among fallers and non-fallers, p=0.022	Significant difference	10
Banu 2018	Injurious falls	Retrospective (medical records)	Medical records	12 (25.0%) of fallers had history of stroke, 27 (16.7%) of non-fallers had history of stroke, P>0.05	Unadjusted OR=2.0 (0.9–4.5), p>0.05	No difference in the prevalence of stroke between fallers and non fallers	No significant difference	8
Bhangu 2017	All falls, non-accidental falls, syncope	Retrospective (self reported)	Self reported	19.4% of participants had a fall in the previous 12 months; 5.1% had a non-accidental fall; and 4.4% had a syncopal event.	Univariate analyses: All falls: OR=1.9 (1.3-2.7),p=<0.05); non-accidental falls: 3.5 (2.2-5.7), p=<0.05); syncope: OR= 3.9 (2.4-6.4), p=<0.05); Adjusted analyses: All falls: OR=1.2 (0.8-1.8),p=>0.05); non-accidental falls: 1.7 (1.0-2.9), p=>0.05); syncope: OR= 2.9 (1.7-4.9), p=<0.05	Significant univariate associations between self reported history of stroke and all falls, non-accidental falls, and syncope. Also significant association between syncope and stroke in adjusted model. No significant association between all falls, non-accidental and self reported history of stroke in adjusted model.	No significant multivariate association	8

First author, Year	Falls outcome	Falls assessment method	Assessment method of stroke	Main findings and prevalence of stroke	OR/RR/HR	Conclusion	Association	NOS
Bhangu 2017	All falls, non-accidental falls, syncope	Retrospective (self reported)	Self reported	19.4% of participants had a fall in the previous 12 months; 5.1% had a non-accidental fall; and 4.4% had a syncopal event.	Univariate analyses: All falls: OR=1.8 (1.3-2.5),p=<0.05); non-accidental falls: 2.7 (1.7-4.3), p=<0.05); syncope: OR= 2.4 (1.4-3.9), p=<0.05); Adjusted analyses: All falls: OR=1.4 (1.0-1.9),p=>0.05); non-accidental falls: 1.5 (1.0-2.6), p=>0.05); syncope: OR= 1.8 (1.1-3.1), p=<0.05	Significant univariate associations between self reported history of transient ischemic attack and all falls, non-accidental falls, and syncope. Also significant association between syncope and TIA in adjusted model. No significant association between all falls, non-accidental and self reported history of TIA in adjusted model.	No significant multivariate association	8
Callisaya 2014	Multiple falls, 12 months prospectively	Prospective (self reported with falls diaries)	Medical history	5.0% of non fallers or single fallers had a history of stroke; 6.1% of multiple fallers.	Unadjusted OR= 1.25 [0.53, 2.93]	No significant difference in the prevalence of stroke among fallers, and non-fallers, p>0.05	No significant difference	10
Chang 2011	Any fall, any time during hospitalisation	Retrospective (reported on patient safety reporting system)	Hospital records	24.2% of fallers had history of stroke; 25.5% of non-fallers	Unadjusted OR= 0.9 (0.6-1.6), p>0.05	No significant association between stroke and falls	No significant univariate association	7
Chang 2015	Any fall	Retrospective (self reported)	Self-reported	33.0% of men with heart disease fell; 16.6% of men without heart disease. 40.3% of women with heart disease fell; 21.6% of women without.	Men: univariate OR= 1.9 (1.3-2.7) Women: univariate OR= 1.53 (1.0-2.3)	Stroke was significantly associated with falls	Significant univariate association	6
Chen 2008	Any fall	Retrospective (self reported)	Medical records	6.4% of fallers had a history of stroke; 9.3% of non-fallers, p=0.5	Unadjusted OR= 0.65 [0.19, 2.17]	No significant difference in the prevalence stroke among fallers and non-fallers	No significant difference	8
Chen 2010	Any fall, any time during hospitalisation	Retrospective (reported on incident information management system)	Medical records	57.1% of reoccurring fallers had stroke; 40.7% of single fallers; 13.0% non-fallers, p=<0.001	Unadjusted OR= 5.23 [2.51, 10.88] / OR= 2.3 (1.3 - 4.3)	Significant difference in the prevalence of stroke among reoccurring, single, and non-fallers, and significant association between all falls and stroke	Significant multivariate association	7
Choi 2014	Any fall, previous 12 months	Retrospective (self reported)	Self reported	8.0% of fallers had stroke; 5.2% of non-fallers, p<0.001	Unadjusted OR= 1.59 [1.46, 1.74] / Multivariate OR= 1.4 (1.3-1.6), p=<0.001	Significant difference in the prevalence of stroke among fallers and non-fallers. Significant association between falls and stroke	Significant multivariate association	7

First author, Year	Falls outcome	Falls assessment method	Assessment method of stroke	Main findings and prevalence of stroke	OR/RR/HR	Conclusion	Association	NOS
Chu 2007	Any fall	Prospective (self reported)	Self reported		Univariate analyses showed a significant association between history of stroke and falls, and reoccurring falls	Univariate analyses showed a significant association between history of stroke and falls, and reoccurring falls. However, these were found to be non-significant in multivariate analyses.	No significant multivariate association	7
Claffey 2022	Any fall	Retrospective (self reported)	Self reported		OR=1.7 (0.7–4.3) p>0.05	No significant association between stroke and falls	No significant multivariate association	8
Coutinho 2008	Injurious falls, directly prior to hospitalisation	Retrospective	Self reported	11.2% of fallers had a history of stroke; 3.2% of non-fallers	Unadjusted OR = 3.82 [1.70, 8.55] / Multivariate OR= 4.3 (1.8-10.5), p<0.01	Significant multivariate association between falls and history of stroke	Significant multivariate association	5
Dahodwala 2017	Any fall	Prospective (self reported)	Medical records		Univariate OR= 1.1 (0.8-1.6), p>0.05; Multivariate OR= 1.1 (0.8-1.6), p>0.05	No significant association between falls and history of stroke	No significant multivariate association	7
Del Brutto 2019	Any fall	Retrospective (self reported)	Self reported	9% of fallers had a history of stroke; 5% of non-fallers, p>0.05	Unadjusted OR= 1.86 [0.77, 4.48] / Multivariate OR= 2.2 (0.9-5.6) p>0.05	No association between stroke and falls	No significant multivariate association	8
Dolinis 1997	Any fall	Retrospective (self reported)	Self reported	16% of fallers had history of stroke or TIA 11% of non-fallers	Unadjusted OR= 1.54 [1.16, 2.05] / Multivariate OR= 1.5 (1.1-2.0)	Significant association between history of stroke or TIA and falls	Significant multivariate association	8
Downton 1991	Any fall	Retrospective	Self reported	16.3% of fallers had history of stroke 5.1% of non-fallers.	Stroke and Falls unadjusted OR=3.60 [1.32, 9.79]	Significant association between stroke and falls	Significant univariate association	6
dos Reis 2015	All fall	Prospective (report book completed by care team)	Self reported	34.2% of participants had a self reported fall in the previous 12 months. 27.4% of participants had a self reported history of stroke. 36.2% of fallers had a self reported history of stroke, compared to 23.8% of non-fallers	Unadjusted OR=1.8 (1.0-3.3), p=0.045	Significant association between self reported history of stroke, and self reported history of falls in the previous 12 months	Significant univariate association	8
Ek 2019	Injurious fall	Derived from national patient database records (clinical examination)	Derived from national patient database records	11.6% of females, and 6.2% of males had an injurious fall within a 48 month period. Within a 48-120 month period, 21.1% of females, and 13.2% of males experienced an injurious fall. 7% of females, and 6.9% of males, had a self reported history of stroke	48 month follow up: Females - OR=1.2 (0.8-1.9), P>0.05; Males - OR - 0.9 (0.4-2.1), P>0.05. 48-120 month follow up: Females - OR=1.4 (1-2.1), p>0.05; Males - OR=0.8 (0.4-1.6), p>0.5	No significant association between self reported falls and self reported history of stroke	No significant multivariate association	8

First author, Year	Falls outcome	Falls assessment method	Assessment method of stroke	Main findings and prevalence of stroke	OR/RR/HR	Conclusion	Association	NOS
Frankenthal 2021	Any fall	Retrospective (self reported)	Self reported	13% of fallers had previous history of stroke; 7.2% of non-fallers, P=<0.001	Unadjusted OR= 1.93 [1.48, 2.51]	There was a significant difference in the prevalence of previous stroke among fallers and non-fallers	Significant difference	7
Frels 2002	Any fall	Prospective (incident book)	Medical records	35% of fallers, and 24% of non-fallers had a stroke (p=<0.05)	Unadjusted OR= 1.66 [1.05, 2.63]	There was a significant difference in the prevalence of previous stroke between fallers and non-fallers	Significant difference	7
Friedman 2002	Any fall	Prospective (self reported)	Self reported	8.1% of participants had a history of stroke	OR= 1.6 (1.2–2.3)	Significant multivariate association between falls and stroke	Significant multivariate association	8
Gebre 2022	Injurious falls	Prospective (self reported)	Medical records		Unadjusted OR= 1.31 [0.91, 1.87] / unadjusted HR: 1.8 (1.2–2.6), adjusted HR: 1.5 (1.0–2.3)	Cerebrovascular disease was associated with a 51% greater relative hazard	Significant multivariate association	8
Geng 2017	Any fall	Retrospective (self reported)	Self reported		Univariate OR= 1.5, CI 1.1–2.1)	Significant univariate association between falls and stroke	Significant univariate association	4
George 2014	Any fall, previous three months	Retrospective (self reported)	Self reported	18% of participants had a fall in the previous three months. 23.4% of participants with a previous stroke had a fall over this period, compared to 17.5% of those who had not had a previous stroke (p>0.05)	Stroke and Falls unadjusted OR=1.45 [1.07, 1.96]	Those with a previous self reported history of stroke were not significantly more likely to have fallen, compared to those without a previous history of stroke.	No significant difference	6
Granek 1987	Any fall	Prospective (incident report)	Medical records	20% of fallers, and 21% of non-fallers had a cerebrovascular accident	OR= 0.9, p>0.05	There was no significant association between cerebrovascular accidents and falls	No significant univariate association	4
Graafmans 1996	Any fall	Prospective	Self reported		Stroke and Falls unadjusted OR=1.95 [0.99, 3.84]	Significant association between stroke and falls	Significant univariate association	9
Grundstrom 2012	Any fall	Retrospective (self reported)	Self reported		Multivariate OR= 1.3 (1.1-1.6)	Significant multivariate association between falls and history of stroke	Significant multivariate association	7
Ha 2021	Any fall (with no external cause e.g., violence)	Retrospective (self reported)	Self reported	7.8% of fallers had a history of stroke; 2.2% of non-fallers, p=0.003	Adjusted OR=2.5 (0.9-7.2), p=>0.05	There was a significant difference in the prevalence of previous stroke among fallers and non-fallers. There was no significant multivariate association between falls and previous history of stroke	No significant multivariate association	8

First author, Year	Falls outcome	Falls assessment method	Assessment method of stroke	Main findings and prevalence of stroke	OR/RR/HR	Conclusion	Association	NOS
Hanlon 2002	Any fall, reoccurring falls	Retrospective (self reported)	Self reported	History of stroke was not significantly associated with falls, p=>0.05	Not reported	There was no association between previous history of stroke and falls	No significant difference	7
Himes 2012	Any fall	Retrospective (self reported)	Self reported		Multivariate OR= 1.7 (1.5-2), p=<0.001	Significant multivariate association between history of stroke and falls	Significant multivariate association	7
Ho 1996	Any fall	Retrospective (self reported)	Self reported	6.5% of participants reported having a previous stroke or TIA	Multivariate OR=: 1.9 (1.2-2.8) p<0.01	Significant association between previous stroke / TIA and falls	Significant multivariate association	8
Homer 2021	Insurance claims related to falls, 24 months prospectively	Insurance claims	Medical records	10.3% of the sample had at least one fall within the follow up period	Unadjusted OR= 2.1 (2.1-2.2)	There was a significant univariate association between cerebrovascular disease and falls	Significant univariate association	6
Hung 2017	Single fall, reoccurring falls, previous 12 months	Retrospective (Self reported history of falls)	Self reported	7.2% of non-fallers had previous history o stroke, 10.7% of single fallers, 8.8% of reoccurring fallers, p>0.05	Unadjusted OR= 1.41 [0.83, 2.40]	No significant difference in stroke between non-fallers, single fallers, and reoccurring fallers	No significant difference	6
Jacob 2022	Any falls	Retrospective (self reported)	Self reported		OR= 2.5 (1.5-4.1), p<0.05	There was a significant multivariate association between stroke and falls	Significant multivariate association	7
Jia 2019	Any fall	Retrospective (self reported)	Self reported		Multivariate OR= 1.2 (1.2, 1.3)	Significant multivariate association between reported history of stroke and falls	Significant multivariate association	6
Jonsson 1990	Reoccurring falls	Retrospective (medical records or incident reports)	Medical records	18% of fallers, and 35% of non-fallers had cerebrovascular disease (p=>0.05)	Unadjusted OR= 0.42 [0.12, 1.44]	There was no significant difference in the prevalence of cerebrovascular disease between fallers and non-fallers	No significant difference	7
Jorgensen 2015	Injurious falls	Retrospective (incident reports)	Medical records	26.7% of fallers had a history of stroke; 20.3%, p=0.001	Unadjusted OR= 1.43 [1.33, 1.54] / OR= 1.6 (1.4-1.9)	Significant difference in the prevalence of stroke between fallers and non-fallers. Significant univariate association between stroke and falls	Significant multivariate association	6
Just 2021	Any fall	Prospective and retrospective (self reported)	Medical history	5.5% of non-fallers had a stroke; 5% of fallers, p>0.05; 6.2% of multiple fallers, p>0.05	Unadjusted OR= 0.90 [0.54, 1.50]	No significant difference between fallers and non fallers with regard to previous history of stroke	No significant difference	8
Kallin 2004	Any fall	Prospective (post fall evaluations)	Medical records	38.9% of fallers had history of stroke; 27.0% of non-fallers, p=0.1	Unadjusted OR= 1.68 [0.91, 3.10]	No significant difference in the prevalence of stroke between fallers and non-fallers	No significant difference	6

First author, Year	Falls outcome	Falls assessment method	Assessment method of stroke	Main findings and prevalence of stroke	OR/RR/HR	Conclusion	Association	NOS
Kang 2018	Any fall, previous 12 months	Retrospective (self reported)	Medical history	11.4% of fallers had CAD; 6.5% of non-fallers	Univariate OR= 1.5 (0.9-3.8), p=0.7	No significant association between stroke and falls	No significant univariate association	7
Kelly 2003	Injurious falls	Retrospective (self reported)	Medical records	6% of fallers had cerebrovascular disease; 3% of non-fallers, p<0.05	Univariate OR= 2.4 (1.9-2.9); Multivariate OR= 1.5 (1.2-1.9)	Significant difference in the prevalence of stroke between fallers and non-fallers; significant univariate association between stroke and falls; significant multivariate association between stroke and falls	Significant multivariate association	6
Kojima 2011	Any fall	Retrospective (self reported)	Medical records	6.5% of participants had a history of stroke	Multivariate OR= 1.4 (0.4–5.5)	No significant association between history of stroke and falls	No significant multivariate association	7
Ku 2013	Any fall	Prospective (resident records)	Self reported	11.1% of fallers had history of stroke; 5.1% of non-fallers, p=0.007	Univariate OR= 2.3 (1.3-4.1), p=0.005; Multivariate OR= 2.2 (1.2-4.0), p=0.013	Significant difference in the prevalence of previous stroke between fallers and non-fallers; significant univariate association between previous stroke and falls; significant multivariate association between previous stroke and falls	Significant multivariate association	9
Lam 2019	Any fall, Injurious falls	Retrospective (self reported)	Self reported		IRR= 0.4 (0.2, 0.9), p=0.02	Significant negative univariate association between falls and stroke	Significant (negative) multivariate association	7
Lee 2020	Any fall	Retrospective (self reported)	Self reported	Not reported	Unadjusted: 0R: 2.1 (2.0-2.3), p=<0.05); Adjusted; OR= 1.3 (1.2-1.4), p=<0.05	Signification association between falls and stroke/TIA in unadjusted and adjusted analyses	Significant multivariate association	8
Lee 2021	Any fall	Prospectively (self reported)	Self reported	7.8% of fallers, and 10.1% of non-fallers had stroke, p>0.05	Unadjusted OR= 0.75 [0.27, 2.13]	There was no significant difference in the prevalence of stroke among fallers and non-fallers	No significant difference	7
Lipsitz 1991	Any fall, 6 moths prospectively	Prospective (computer records and review reports)	Clinical evaluation	20% of fallers had a history of stroke / TIA; 29% of non-fallers, p>0.05	Unadjusted OR= 0.63 [0.27, 1.42]	No significant difference in the prevalence of stroke /TIA between fallers and non-fallers	No significant difference	8
Lord 2003	Any fall, Injurious falls	Prospective (medical records)	Medical reports and other		Unadjusted OR= 1.15 [0.77, 1.73] / Able to stand unaided: IRR= 1.2 (0.9–1.6), p>0.05; Unable to stand unaided: IRR=0.6 (0.3-0.9), P<0.05	Significant negative univariate association between stroke and falls in those unable to stand	Significant (negative) univariate association	7

First author, Year	Falls outcome	Falls assessment method	Assessment method of stroke	Main findings and prevalence of stroke	OR/RR/HR	Conclusion	Association	NOS
Lukaszyk 2018	Any fall	Retrospective (self reported)	Self reported	37% of fallers had a history of stroke; 18% of non-fallers	Unadjusted OR= 2.60 [1.49, 4.52] / RR= 1.9 (1.3–2.9), p<0.05	Significant multivariate association between stroke and falls	Significant multivariate association	7
Magnuszewski 2020	Any fall	Retrospective (self reported)	Self reported	14.7% of fallers had a history of stroke / TIA; 9% of non-fallers, p>0.05	Unadjusted OR= 1.75 [0.91, 3.36] / OR= 1.8 (0.7-4.7), p>0.05	No significant difference in the prevalence of stroke / TIA between fallers and non-fallers	No significant multivariate association	8
Magnuszewski 2022	Any fall	Prospective (incident reports)	Medical records	21.4% of fallers had history of stroke/TIA, 13.2% of non-fallers did p>0.05	Unadjusted OR= 1.80 [0.49, 6.65]	No significant difference in the prevalence of stroke/TIA between fallers and non-fallers	No significant difference	8
Menant 2016	Unexplained falls	Prospective	Self report	3.4% of non-fallers had previous history of stroke, 2.9% of unexplained fallers, p=>0.05	Unadjusted OR= 0.83 [0.10, 6.66] / OR = 0.8 (0.1-6.6), p=>0.05	No significant difference in previous history of stroke between non-fallers and fallers; no significant association between stroke and falls	No significant multivariate association	10
Mitchell 2015	Any fall	Retrospective (self reported)	Self reported		RR=1.1 (1-1.2), p=0.003	Significant association between self reported history of stroke, and self reported history of falls in the previous 12 months	Significant multivariate association	8
Ooi 2021	Any fall	Retrospective and prospective (self reported)	Self reported	1.5% of non-fallers had previous stroke; 1.3% of occasional fallers, p>0.05; 4.7% of reoccurring fallers, p=0.026	Reoccurring falls: Univariate: OR=3.2 (1.1-9.6), p=0.036	There was a significant difference in the prevalence of previous stroke among fallers and non-fallers. There was a significant univariate association between falls and previous history of stroke	Significant univariate association	7
Paganini-Hill 2017	Any fall	Retrospective (self reported)	Medical records	23% of non-fallers had a TIA or stroke; 35% of fallers	Unadjusted OR= 1.71 [1.36, 2.15]	No significant difference in prevalence of stroke/TIA among fallers and non-fallers	No significant difference	8
Paliwal 2017	Any fall	Retrospective (self reported)	Medical records	10.8% of fallers had previous history of stroke, 6.% of non-fallers	Crude OR = 2 (1.8-2.1), p=<0.05; Adjusted OR =1.6 (1.5-1.8), p=<0.5	No significant difference reported with regard to stroke between fallers and non-fallers; significant association between falls and stroke	Significant multivariate association	8
Prudham 1981	Any fall	Retrospective (self reported)	Self reported	10.4% of fallers had stroke; 5.6% of non-fallers, p<0.001	Unadjusted OR= 1.97 [1.42, 2.72]	Significant difference in the prevalence of stroke between fallers and non fallers	Significant difference	4

First author, Year	Falls outcome	Falls assessment method	Assessment method of stroke	Main findings and prevalence of stroke	OR/RR/HR	Conclusion	Association	NOS
Rivera-Chavez 2021	Any fall	Retrospective (self reported)	Direct interrogation and clinical history, as part of direct geriatric assessment	16% of fallers had a previous stroke; 19% of non-fallers, p>0.05	Unadjusted OR= 0.77 [0.52, 1.16] / Univariate: 0R=0.8 (0.5-1.2), p>0.05	There was no significant association between falls and previous stroke, or significant difference in the prevalence of previous stroke among fallers and non-fallers	No significant univariate association	7
Rosendahl 2003	Any fall	Prospective	Medical records	35% of fallers had a history of stroke; 27% of non-fallers, p>0.05	Unadjusted OR= 1.51 [0.55, 4.11]	No significant difference in the prevalence of stroke between fallers and non-fallers	No significant difference	5
Salgado 1994	Any fall	Retrospective (incident reports)	Clinical examination		Unadjusted OR= 6.92 [2.10, 22.77] / Multivariate OR=4.5 (1.2-21.7)	Significant association between history of stroke and falls	Significant multivariate association	7
Salgado 2004	Any fall	Prospective	Clinical examination	13.3% of fallers had a history of stroke; 1.4% of non-fallers	Unadjusted OR= 11.08 [0.94, 131.24] / RR=4.4 (1.7–11.2)	Significant association between falls and stroke	Significant multivariate association	6
Sharma 2017	Any falls, previous 12 months	Retrospective (self reported)	Medical records	8.2% of non-fallers had a history of stroke, 16.7% of fallers, p=0.15	Unadjusted OR= 1.83 [0.84, 3.98]	No significant difference in the prevalence of history of stroke between fallers and non-fallers	No significant difference	7
Shaw 2015	Any falls	Retrospective (self reported)	Medical records	30.8% of fallers had a stroke; 10% of non-fillers, p>0.05	Unadjusted OR= 4.00 [0.74, 21.50]	No significant difference between fallers and non fallers with regard to previous history of stroke	No significant difference	5
Sorock 1983	Any fall	Retrospective (medical records)	Medical records	21.6% of fallers had stroke. 25.1% Of non fallers had a stroke	Unadjusted OR= 0.83 [0.53, 1.28]	No difference between stroke and falls	No significant difference	6
Subramanian 2020	Any fall	Retrospective	Self reported	5.3% of fallers had a history of stroke / TIA; 5.7% of non-fallers, p>0.05	Unadjusted OR= 0.91 [0.18, 4.59]	No significant difference in the prevalence of stroke / TIA between fallers and non-fallers	No significant difference	5
Svensson 1992	Any falls	Retrospective (self reported) interview	Self reported	5% of fallers had TIA v 2% non fallers - significant, p<0.05	Unadjusted OR= 2.47 [1.07, 5.73]	Significant association between TIA and Falls	Significant difference	7
Teoh 2021	Any fall	Retrospective (self reported)	Self reported	1.2% of non-fallers had a previous history of stroke; 2.2% of fallers, p>0.05	Unadjusted OR= 0.71 [0.29, 1.73]	No significant difference in the prevalence of stroke between fallers and non-fallers	No significant difference	8
Thapa 2022	Any falls	Medical records	Medical records	17 (11.1%) of fallers had history of stroke, 255 (16.0%) of non-fallers did, p>0.05	Unadjusted OR= 0.66 [0.39, 1.11]	No significant difference between history of stroke and falls	No significant difference	6
Tsai 2021	Fall in last month	Retrospective (self reported) interview	Medical records	523 (9.6%) of people with stroke fell, 4,923 (90.4%) of people with stroke did not fall. Unadjusted OR 2.10 (1.70-2.59) p<0.001	OR= 1.5 (1.2-1.9) P<0.001	Significant association between stroke and Falls	Significant multivariate association	6
Turusheva 2020	Any falls, injurious falls	Retrospective (self reported)	Medical records	44.4% of fallers had stroke; 11% non-fallers, p<0.05	Unadjusted OR= 6.45 [2.22, 18.71]	No significant difference in stroke between fallers and non-fallers	Significant difference	8

First author, Year	Falls outcome	Falls assessment method	Assessment method of stroke	Main findings and prevalence of stroke	OR/RR/HR	Conclusion	Association	NOS
Valderrama-Hinds 2018	Any fall, previous 24 months	Retrospective (self reported)	Self reported	3% of fallers had history of stroke; 2.3% non-fallers, p>0.05	Unadjusted OR= 1.53 [1.15, 2.05] / OR= 1.4 (1.0-1.9)	No significant difference in the prevalence of previous stroke between fallers and non-fallers; significant association between falls and previous stroke	Significant univariate association	6
von Heideken Wågert 2009	Any fall	Prospective (falls diaries, phone calls, medical records)	Medical records and self report	23% of fallers had history of stroke. 22% of non fallers had history of stroke, p>0.05	Unadjusted OR= 1.65 [0.85, 3.18]	No significant difference between stroke and falls	No significant difference	10
Xu 2015	Any fall	Prospective	Self reported medical history	27% of fallers had a previous stroke; 4.2% of non-fallers, p<0.001	Unadjusted OR= 8.52 [4.32, 16.81] / Multivariate RR= 2.4 (1.5–3.9), p<0.001	Significant difference in the prevalence of stroke between fallers and non-fallers; significant multivariate association between falls and previous stroke	Significant multivariate association	10
Yasumura 1994	Any fall	Retrospective (self reported)	Self reported		Multivariate OR= 2.9 (1.0-8.0) (for men)	Significant association between previous stroke and falls	Significant multivariate association	7
Yi 2021	Any fall	Retrospective (self reported)	Self reported		Men: Multivariate OR= 2.3 (2.0–2.6), p<0.001; Women: Multivariate OR= 1.8 (1.6–2.1), p<0.001	Significant multivariate association between stroke and falls	Significant multivariate association	8
Yoo 2016	Any fall	Retrospective	Self reported	City: 10.5% of fallers had history of stroke, 3.1% of non-fallers, p=0.03; Rural: 9.7% of fallers had a history of stroke, 10.7% of non-fallers, p>0.05	Unadjusted OR= 3.59 [1.09, 11.82]	Significant difference in the prevalence of stroke between fallers and non-fallers in the city, not in rural settings	Significant difference	6
Yu 2009	Any fall	Retrospective	Not specified	38 (28.8%) of fallers had prior stroke; 234 (17.0%) of fallers did not have stroke	Unadjusted OR= 1.98 [1.32, 2.96] / OR= 1.9 (1.2-2.8), p=0.003	Significant association between stroke and Falls	Significant multivariate association	7
Zhao 2019	Any falls	Retrospective (self reported)	Self reported	6.6% of fallers had a history of stroke, 4.8% of non-fallers, p>0.05	Unadjusted OR= 1.41 [0.82, 2.43]	No significant difference in stroke between fallers and non-fallers	No significant difference	7

Note. OR= odd ratio. RR: risk ratio. HR: hazard ratio. TIA: transient ischemic attack. NOS: Newcastle Ottawa Scale.

eTable 13. General cardiovascular disease and falls

First author, Year	Falls outcome	Falls assessment method	Assessment method of general cardiovascular disease	Main findings and prevalence of CVD	OR/RR/HR	Conclusion	Association	NOS
Aburub 2021	Any fall	Retrospective (self reported)	Self reported	53.3% of fallers had CVD; 48.1% of non-fallers, p=0.17		There was no significant difference in the prevalence of cardiovascular disease between fallers and non-fallers	No significant difference	7
Alamgir 2015	Any fall	Retrospective	Self reported	Not given	CVD & falls RR= 1.1 (0.6–1.8)	CVD was not associated with falls	No significant multivariate association	5
Brassington 2000	Any fall	Retrospective	Self reported	30% of fallers, 22% of non-fallers.	CVD & falls Unadjusted OR= 1.5 (1.1–2.0)	CVD is univariately associated with falls	Significant univariate association	6
Chang 2011	Any fall, any time during hospitalisation	Retrospective (reported on patient safety reporting system)	Hospital records	23.0% of fallers had heart disease; 27.3% of non-fallers	Heart disease & falls Unadjusted OR= 0.8 (0.5-1.3), p>0.05	No significant association between heart disease and falls	No significant univariate association	7
Chen 2008	Any fall	Retrospective (self reported)	Medical records	12.5% of fallers had a history of cardiovascular disease; 5.2% of non-fallers, p=0.04	CVD & falls Unadjusted OR= 2.60 [1.02, 6.62]	Significant difference in the prevalence of cardiovascular disease among fallers and non-fallers. No significant association cardiovascular disease and falls	No significant univariate association	8
Claffey 2022	Any falls	Retrospective (self reported)	Self reported		Adjusted OR=1.2 (0.8–1.8) p>0.05	No significant association between heart disease and falls	No significant multivariate association	8
Ek 2019	Injurious fall	Derived from national patient database records (clinical examination)	Derived from national patient database records	11.6% of females, and 6.2% of males had an injurious fall within a 48 month period. Within a 48-120 month period, 21.1% of females, and 13.2% of males experienced an injurious fall. 22.4% of females, and 27.9% of males, had self reported history of heart disease***	48 month follow up: Females - HR=1.6 (1.2-2.1), p=0.002; Males - HR - 1.6 (0.9-2.5), p>0.05. 48-120 month follow up: Females - HR=1.5 (1.2-1.9), p=0.002; Males - HR=2.1 (1.5-2.9), p=<0.001. Multivariate: HR=2.2 (1.5-3.2), p=<0.05	Significant association between self reported falls and self reported history of heart disease in females at 48 months, and 48-120 months follow up. No significant associations among males at 48 month follow-up, however significant association found during 48-120 month follow up; Multivariate: significant association between cardiovascular disease and falls	Significant multivariate association	8

First author, Year	**Falls outcome**	**Falls assessment method**	**Assessment method of general cardiovascular disease**	**Main findings and prevalence of CVD**	**OR/RR/HR**	**Conclusion**	**Association**	**NOS**
Frankenthal 2021	Any fall, previous 12 months	Retrospective (self reported)	Self reported	32.9% of fallers had cardiac disease (arrhythmia/chronic heart failure); 17.6% of non-fallers, p=<0.001	Unadjusted OR= 2.30 [1.89, 2.78]Adjusted OR=1.4 (1.1-1.8), p=<0.05	There was a significant difference in the prevalence of cardiac disease among fallers and non-fallers. There was a significant multivariate association between falls and cardiac disease	Significant multivariate association	7
Gamage 2019	Any fall	Retrospective (Self reported)	Self reported previous diagnosis by physician	Prevalence of falls was 34.3%; reoccurring falls 9.7%. 19.4% of fallers had cardiac diseases; 7.1% of non-fallers (p=0.001). 31% of reoccurring fallers had cardiac diseases; 9.2% of non-reoccurring (single falls, and non-fallers) (p=<0.001).	Falls (Crude OR=3.1 (1.5-6.5), p=0.002; reoccurring falls (Crude OR=2.8 (1.5-6.5), p=0.002	Significant association between falls and cardiovascular disease in univariate and multivariate analyses	Significant univariate association	8
Gebre 2021	Injurious falls	Prospective (self reported)	Derived from digitally enhanced lateral single-energy images of the thoraco-lumbar spine using a Hologic 4500A bone densitometer.		Multivariate HR: 1.4 (1.1–1.8)	Significant association between abdominal aortic calcification and injurious falls resulting in hospitalisation	Significant multivariate association	8
Gebre 2022	Injurious falls	Prospective (self reported)	Medical records	48 (14.0%) of people with CVD had previous falls, 121 (11.1%) of people without CVD had previous falls p>0.05	Unadjusted HR= 1.40 (1.17–1.69), adjusted HR= 1.29 (1.07–1.56)	CVD was associated with a 29% greater relative hazard	Significant multivariate association	7
Hartog 2015	Any fall	Self reported	Medical records	39.8% of participants had cardiovascular disease	adjusted OR= 0.4 (0.2-0.9), p=>0.05	Cardiovascular disease not significantly associated with falls	No significant multivariate association	7
Heckenbach 2014	Any fall	Retrospective (Medical records - ICD 10 codes)	Medical records	30% of fallers, 18% of non-fallers.	unadjusted OR= 1.90 [1.60, 2.26] adjusted OR= 1.5 (1.2–1.9)	Arterial disease was associated with falls	Significant multivariate association	6
Hosseini 2020	Any fall	Self reported	Through self-report, examination, laboratory tests, standard questionnaires, or observation of prescriptions and consumed medications	8.3% of fallers had heart disease	Unadjusted OR=1.7 (1.1-2.8), p=0.03; Adjusted OR=1.4 (0.8-2.3), p>0.05	Unadjusted: significant association between cardiovascular disease, and falls, adjusted, there is no significant associations	No significant multivariate association	8

First author, Year	**Falls outcome**	**Falls assessment method**	**Assessment method of general cardiovascular disease**	**Main findings and prevalence of CVD**	**OR/RR/HR**	**Conclusion**	**Association**	**NOS**
Hussain 2022	Any fall	Retrospective (self report)	Medical records	6.6% of fallers, and 2.7% of non-fallers had a history of cardiovascular disease (p=<0.05)	Unadjusted OR= 2.47 [1.96, 3.11]	There was a significant difference in the prevalence of cardiovascular disease between fallers and non-fallers	Significant difference	8
Jacob 2022	Any falls	Retrospective (self reported)	Self reported		adjusted OR= 1.01 (0.84, 1.20), p>0.05	No significant association between heart disease and falls	No significant multivariate association	7
Kao 2012	Reoccurring or Injurious falls	Retrospective	Self reported	37% of fallers and 26% of non fallers.	Unadjusted OR=1.69(1.05-2.73) adjusted OR= 1.5 (0.9–2.6)	CVD was not associated with falls	No significant multivariate association	6
Kario 2001	Any fall during 12 month FU	Prospective, retrospective	Self reported	15% of the sample had a history or heart disease (other than myocardial infarction)	RR= 1.3 (0.7-2.2)	There was no significant association between heart disease (other than myocardial infarction) and falls	No significant univariate association	9
Kelly 2003	Injurious falls	Retrospective (self reported)	Medical records	25% of fallers; 19% of non-fallers, p<0.05	Unadjusted OR= 1.4 (1.3-1.6); Adjusted OR= 1.1 (1.0-1.2)	Significant difference in the prevalence of cardiovascular disease between fallers and non-fallers; significant univariate association between cardiovascular disease and falls; non-significant multivariate association between cardiovascular disease and falls	No significant multivariate association	6
Lee 2006	Any fall	Retrospective	Self reported	Total prevalence 17%	Adjusted OR= 1.6 (1.4–2.0)	Heart disease was associated with single and reoccurring falls	Significant multivariate association	5
Menant 2016	Unexplained falls	Prospective	Self reported	17.5% of non-fallers had heart disease; 5.7% of unexplained fallers	Unadjusted OR = 0.29 [0.07, 1.26]	No significant difference in heart disease between fallers and non-fallers; no significant association between heart disease and falls	No significant multivariate association	10
Ooi 2021	Any fall	Retrospective and prospective (self reported)	Self reported	10.1% of non-fallers had cardiovascular disease; 9.4% of occasional fallers, p=0.05; 8.2% of reoccurring fallers, p>0.05	Unadjusted OR=0.89 [0.58, 1.35]	There were no significant differences in the prevalence of cardiovascular disease between non fallers and occasional fallers, and reoccurring fallers	No significant difference	7

First author, Year	**Falls outcome**	**Falls assessment method**	**Assessment method of general cardiovascular disease**	**Main findings and prevalence of CVD**	**OR/RR/HR**	**Conclusion**	**Association**	**NOS**
Paganini-Hill 2017	Any fall, previous 12 months	Retrospective (self reported)	Medical records	42% of non-fallers had cardiovascular disease; 47% among fallers, p=0.03	Unadjusted OR=1.25 [1.02, 1.53]	Significant difference in prevalence of cardiovascular disease between fallers and non-fallers	Significant difference	8
Prudham 1981	Any fall	Retrospective	Self reported, medical records	CVD 21% of fallers vs. 16% of non fallers (p<0.05)	Unadjusted OR=1.34 [1.07, 1.69]	CVD is more prevalent in fallers than non-fallers in the community	Significant difference	4
Rivan 2021	Any falls, injurious falls	retrospective	Self reported	15 (11.0%) had heart disease in fallers 16 (6.1%) in non-fallers P=0.113 in injurious fallers 11 (11.3%) had heart disease 26 (7.3%) did not p>0.05	Unadjusted OR=1.92 [0.92, 4.02]	No significant association between heart disease and falls or injurious falls	No significant difference	7
Sagawa 2018	Injurious falls	Prospective (self reported)	Self reported	17.1% of patients with fall injuries had cardiovascular disease, 17.0% of participants with a fall had cardiovascular disease, p=>0.05	Unadjusted OR=1.01 [0.78, 1.32]	No significant difference in cardiovascular disease between non-fallers and fallers	No significant difference	8
Sharma 2017	Any falls	Retrospective (self reported)	Self reported	47.3% of non-fallers had a history of cardiovascular disease; 70.8% of fallers, p=0.02	Unadjusted OR=3.21 [1.74, 5.91] adjusted OR=2.16(1.13-4.12)	Significant difference in the prevalence of cardiovascular disease between fallers and non-fallers	Significant difference	7
Sorock 1983	Any fall	Retrospective (medical records)	Self reported	7% of fallers had heart disease. 6.5% of non fallers had a heart disease	Unadjusted OR=1.09 [0.52, 2.25]	No difference between heart disease and falls	No significant difference	6
Tsai 2021	Any fall	Retrospective (self reported) interview	Medical records	483 (9.6%) of people with heart disease fell, 4,534 (90.4%) of people with heart disease did not fall. Unadjusted OR 1.65 (1.36-1.99) p<0.001	Unadjusted OR= 1.65 [1.36, 2.00] adjusted OR= 1.2 (1.0- 1.5), p>0.05	No significant multivariate relationship between heart disease and falls.	No significant multivariate association	6
Vieira 2018	Any falls, previous 12 months	Retrospective (self reported)	Self reported		Gross PR=1.5 (1.2-1.7), p=<0.001, Adjusted PR=1.2 (1-1.5), p=0.03	Significant difference in falls in those with and without heart problems	Significant multivariate association	8

Note. OR= odd ratio. RR: risk ratio. HR: hazard ratio. CVD: cardiovascular disease. NOS: Newcastle Ottawa Scale.

eTable 14. Peripheral arterial disease and falls

First author, Year	Falls outcome	Falls assessment method	Assessment method of Peripheral arterial disease	Main findings and prevalence of IHD	OR/RR/HR	Conclusion	Association	NOS
Arseven 2008	Non-syncopal falls	Prospective (self reported)	Ankle Brachial Index, Ankle Brachial Index, <0.90	37.8% of fallers had peripheral arterial disease; 55% of non-fallers, p=0.07	unadjusted OR=0.50 [0.24, 1.05] Adjusted HR= 0.4 (0.2-0.9), p=0.018	Significant negative relationship between peripheral arterial disease and falls	Significant (negative) multivariate association	10
Ha 2021	Any fall (with no external cause e.g., violence)	Retrospective (self reported)	Self reported	10.2% of fallers had peripheral arterial disease; 3.2% of non-fallers , p=0.001	unadjusted OR =3.46 [1.56, 7.67]Adjusted OR=2.2 (0.9-5.4), p=>0.05	There was a significant difference in the prevalence of peripheral arterial disease between fallers and non-fallers. There no significant multivariate association between falls and peripheral arterial disease	No significant multivariate association	8
Hung 2017	Single fall, reoccurring falls, previous 12 months	Retrospective (Self reported history of falls)	Self reported	2.9% of non-fallers had peripheral arterial disease; 3.1% of single fallers; 3.3% of reoccurring fallers, p=0.8	unadjusted OR=1.08 [0.45, 2.60]	No significant difference in peripheral arterial disease among non-fallers, single fallers, and reoccurring fallers	No significant difference	6
Magnuszewski 2020	Any fall	Retrospective (self reported)	Self reported	26.8% of fallers had history of peripheral arterial disease; 16.4% of non-fallers, p=0.02	unadjusted OR=1.86 [1.11, 3.11] adjusted OR=0.9 (0.4-2), p>0.05	Significant difference in the prevalence of peripheral arterial disease between fallers and non-fallers. No significant multivariate association	No significant multivariate association	8
Magnuszewski 2022	Any fall	Prospective (incident reports)	Medical records	21.4% of fallers had peripheral arterial disease, 15.2% of non-fallers did, p>0.05	unadjusted OR=1.52 [0.41, 5.62]	No significant difference in the prevalence of peripheral arterial disease between fallers and non-fallers	No significant difference	8
Sorock 1983	Any fall	Retrospective (medical records)	Medical records	8.7% of fallers had peripheral vascular disease .5.6% Of non fallers had a peripheral vascular disease.	unadjusted OR=1.62 [0.80, 3.27]	No difference between peripheral vascular disease and falls	No significant difference	6
Turusheva 2020	Any falls, injurious falls	Retrospective (self reported)	Medical records	16.7% of those who fell had peripheral arterial disease; 15.2% of those who did not fall, p=>0.05	unadjusted OR=1.12 [0.30, 4.19]	No significant difference in peripheral arterial disease among those fallers and non-fallers	No significant difference	8

Note. OR= odd ratio. RR: risk ratio. HR: hazard ratio. NOS: Newcastle Ottawa Scale.

eTable 15. Arterial stiffness and falls

First author, Year	Falls outcome	Falls assessment method	Assessment method of arterial stiffness	Main findings and prevalence of arterial stiffness	OR/RR/HR	Conclusion	Association	NOS
Turusheva 2020	Any falls, injurious falls	Retrospective (self reported)	Cardio-ankle vascular index	30.8% of those with arterial stiffness had a fall, 23.5% of those without, p=>0.05; 17.9% of those with arterial stiffness had an injurious fall, compared to 4.7% of those without arterial stiffness, p=<0.05	Falls and High CAVI (arterial Stiffness)Unadjusted OR=1.44 [0.72, 2.89] adjusted OR=3.52 [1.03, 12.03]	No significant difference in falls between those with and without arterial stiffness; Significant difference in injurious falls between those with and without arterial stiffness	Significant difference (injurious falls)	8
Wong 2014	Any fall	Prospective	Carotid–femoral PWV measured supine using a semi-automated pulse wave analysis system. High PWV was taken as the top quintile (>13 m/s)	Arterial stiffness	Pulse wave velocity 11.5 (2.6) m/s in fallers and 11.0 (2.2) m/s in non-fallers, RR= 1.05 (1.0–1.1). Top quintile of PWV & falls RR= 1.4 (1.1–1.8), adjusted for age, gender and other confounding factors	Arterial stiffness is an independent predictor of future falls	Significant multivariate association	9

Note. OR= odd ratio. RR: risk ratio. HR: hazard ratio. PWV: pulse wave velocity. NOS: Newcastle Ottawa Scale.

Study or Subgroup	log[Odds Ratio]	SE	Weight	Odds Ratio IV, Random, 95% CI
Turusheva 2020	-1.65	1.0475	0.1%	0.19 [0.02, 1.50]
Hartog 2015	-0.5621	0.463	0.3%	0.57 [0.23, 1.41]
Wong 2014	-0.2737	0.1873	1.4%	0.76 [0.53, 1.10]
Akande-Sholabi 2021	-0.0805	0.2091	1.1%	0.92 [0.61, 1.39]
Margolis 2019	-0.0728	0.1259	2.6%	0.93 [0.73, 1.19]
Svensson 1992	-0.0481	0.1606	1.8%	0.95 [0.70, 1.31]
Just 2021	-0.0169	0.1154	2.9%	0.98 [0.78, 1.23]
Kao 2012	-0.0101	0.2306	1.0%	0.99 [0.63, 1.56]
Kelly 2003	-0.0101	0.0543	6.3%	0.99 [0.89, 1.10]
Xu 2015	0.0235	0.2256	1.0%	1.02 [0.66, 1.59]
Lawlor 2003	0.0404	0.0838	4.3%	1.04 [0.88, 1.23]
Jansen 2015 (a)	0.0583	0.0559	6.2%	1.06 [0.95, 1.18]
Choi 2014	0.0676	0.0236	8.6%	1.07 [1.02, 1.12]
Bhangu 2017	0.077	0.0601	5.9%	1.08 [0.96, 1.22]
Prudham 1981	0.0876	0.1092	3.1%	1.09 [0.88, 1.35]
Goh 2017	0.0983	0.141	2.2%	1.10 [0.84, 1.45]
Mitchell 2013	0.1044	0.0688	5.2%	1.11 [0.97, 1.27]
Paganini-Hill 2017	0.1139	0.1022	3.4%	1.12 [0.92, 1.37]
Magnuszewski 2020	0.1231	0.2701	0.7%	1.13 [0.67, 1.92]
Chang 2015 (M)	0.131	0.093	3.8%	1.14 [0.95, 1.37]
Kamali 2022	0.1537	0.2482	0.8%	1.17 [0.72, 1.90]
Yoo 2016	0.1793	0.287	0.6%	1.20 [0.68, 2.10]
Chang 2015 (F)	0.1906	0.0922	3.9%	1.21 [1.01, 1.45]
Tsai 2021	0.1989	0.0914	3.9%	1.22 [1.02, 1.46]
Sibley 2014	0.2022	0.0394	7.4%	1.22 [1.13, 1.32]
Kang 2018	0.207	0.2069	1.2%	1.23 [0.82, 1.85]
Del Brutto 2019	0.2246	0.2247	1.0%	1.25 [0.81, 1.94]
Saedon 2016	0.2396	0.2485	0.8%	1.27 [0.78, 2.07]
Almegbel 2018	0.2442	0.1166	2.9%	1.28 [1.02, 1.60]
Sharma 2017	0.2631	0.2558	0.8%	1.30 [0.79, 2.15]
Teoh 2021	0.2717	0.1322	2.4%	1.31 [1.01, 1.70]
Ku 2013	0.3001	0.1739	1.6%	1.35 [0.96, 1.90]
Zia 2015	0.3047	0.2141	1.1%	1.36 [0.89, 2.06]
Lee 2021	0.361	0.2951	0.6%	1.43 [0.80, 2.56]
Brassington 2000	0.392	0.1332	2.4%	1.48 [1.14, 1.92]
Yu 2009	0.3937	0.1344	2.3%	1.48 [1.14, 1.93]
Liao 2012	0.4074	0.1557	1.9%	1.50 [1.11, 2.04]
Ha 2021	0.5423	0.2098	1.1%	1.72 [1.14, 2.59]
Chan 1997	0.5786	0.2662	0.7%	1.78 [1.06, 3.01]
Gamage 2019	0.7885	0.2684	0.7%	2.20 [1.30, 3.72]
**Total (95% CI)**			**100.0%**	**1.14 [1.09, 1.20]**

Heterogeneity: Tau² = 0.01; Chi² = 64.98, df = 39 (P = 0.006); I² = 40%
Test for overall effect: Z = 5.57 (P < 0.00001)

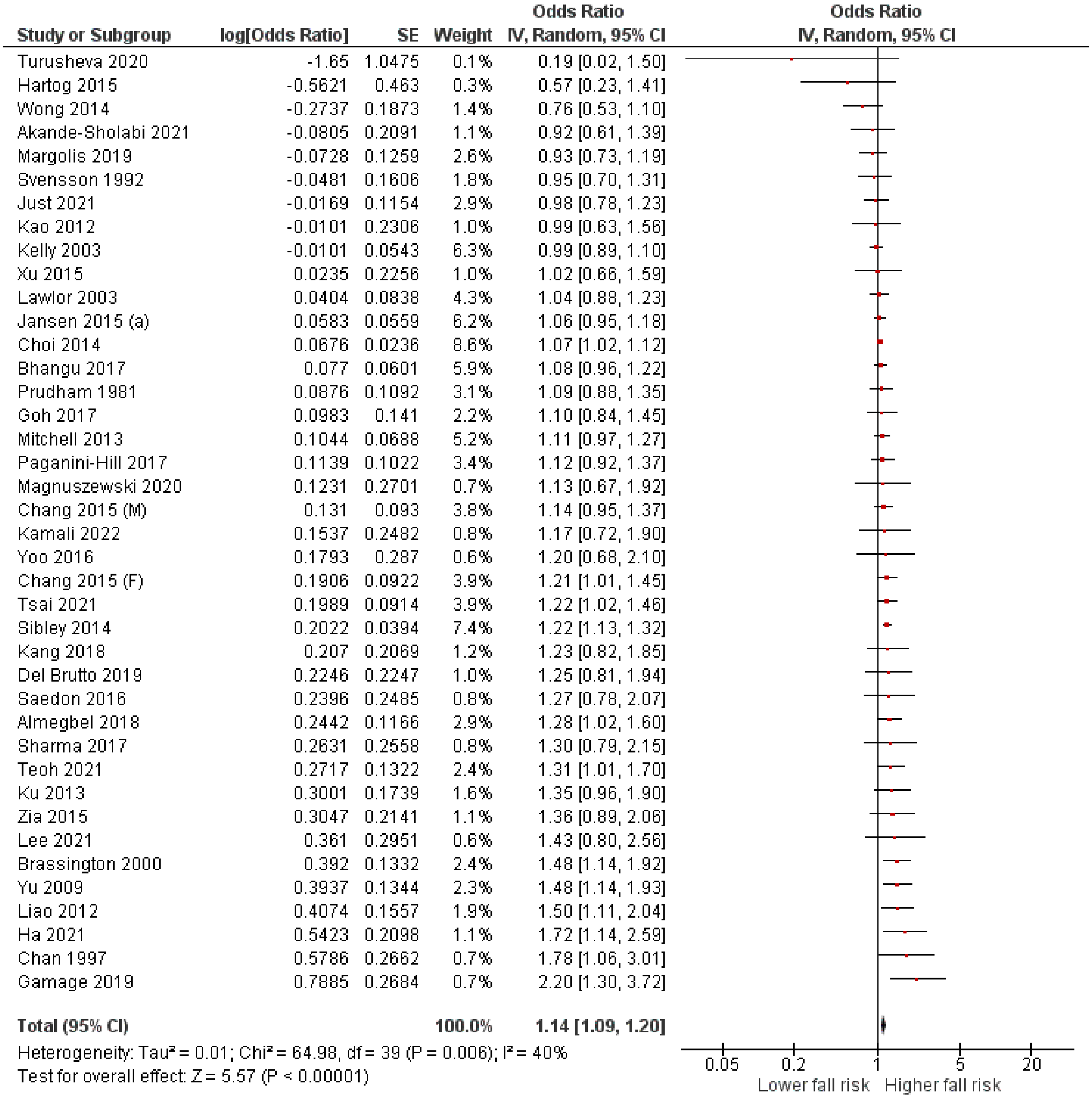

eFigure 1. Association between hypertension and falls (unadjusted OR)

Study or Subgroup	log[Odds Ratio]	SE	Weight	Odds Ratio IV, Random, 95% CI
Teoh 2021	-0.3433	0.4538	1.3%	0.71 [0.29, 1.73]
Lee 2021	-0.284	0.5314	1.0%	0.75 [0.27, 2.13]
Menant 2016	-0.1906	1.0644	0.3%	0.83 [0.10, 6.66]
Just 2021	-0.1071	0.2618	2.9%	0.90 [0.54, 1.50]
Subramanian 2020	-0.0913	0.8242	0.4%	0.91 [0.18, 4.59]
Callisaya 2015	0.2195	0.4372	1.3%	1.25 [0.53, 2.93]
Hung 2017	0.3428	0.2709	2.8%	1.41 [0.83, 2.40]
Akande-Sholabi 2021	0.3564	0.5657	0.9%	1.43 [0.47, 4.33]
Geng 2017	0.4055	0.1582	5.1%	1.50 [1.10, 2.05]
Rosendahl 2003	0.4108	0.5114	1.0%	1.51 [0.55, 4.11]
Kang 2018	0.4318	0.2684	2.8%	1.54 [0.91, 2.61]
Dolinis 1997	0.4326	0.1445	5.5%	1.54 [1.16, 2.05]
Choi 2014	0.4666	0.0458	8.4%	1.59 [1.46, 1.74]
Kallin 2004	0.5203	0.3113	2.3%	1.68 [0.91, 3.10]
Paganini-Hill 2017	0.5365	0.1171	6.3%	1.71 [1.36, 2.15]
Magnuszewski 2020	0.5568	0.3346	2.1%	1.75 [0.91, 3.36]
Sharma 2017	0.6047	0.3965	1.6%	1.83 [0.84, 3.98]
Del Brutto 2019	0.6205	0.448	1.3%	1.86 [0.77, 4.48]
Frankenthal 2021	0.6561	0.1353	5.8%	1.93 [1.48, 2.51]
Paliwal 2017	0.6729	0.0406	8.5%	1.96 [1.81, 2.12]
Prudham 1981	0.6774	0.1653	4.9%	1.97 [1.42, 2.72]
Yu 2009	0.683	0.2052	4.0%	1.98 [1.32, 2.96]
Chang 2015 (F)	0.7885	0.1886	4.3%	2.20 [1.52, 3.18]
Ku 2013	0.8372	0.2973	2.5%	2.31 [1.29, 4.14]
Chang 2015 (M)	0.8587	0.1795	4.6%	2.36 [1.66, 3.36]
Kelly 2003	0.8671	0.1096	6.6%	2.38 [1.92, 2.95]
Svensson 1992	0.9057	0.4285	1.4%	2.47 [1.07, 5.73]
Lukaszyk 2018	0.9538	0.283	2.6%	2.60 [1.49, 4.52]
Almegbel 2018	1.0116	0.4317	1.4%	2.75 [1.18, 6.41]
Yoo 2016	1.2787	0.6079	0.7%	3.59 [1.09, 11.82]
Downton 1991	1.2802	0.5109	1.0%	3.60 [1.32, 9.79]
Ha 2021	1.3324	0.4729	1.2%	3.79 [1.50, 9.58]
Shaw 2015	1.3863	0.858	0.4%	4.00 [0.74, 21.50]
Turusheva 2020	1.8641	0.5434	0.9%	6.45 [2.22, 18.71]
Xu 2015	2.1422	0.3467	2.0%	8.52 [4.32, 16.81]
**Total (95% CI)**			**100.0%**	**1.90 [1.70, 2.11]**

Heterogeneity: Tau² = 0.03; Chi² = 77.53, df = 34 (P < 0.0001); I² = 56%

Test for overall effect: Z = 11.66 (P < 0.00001)

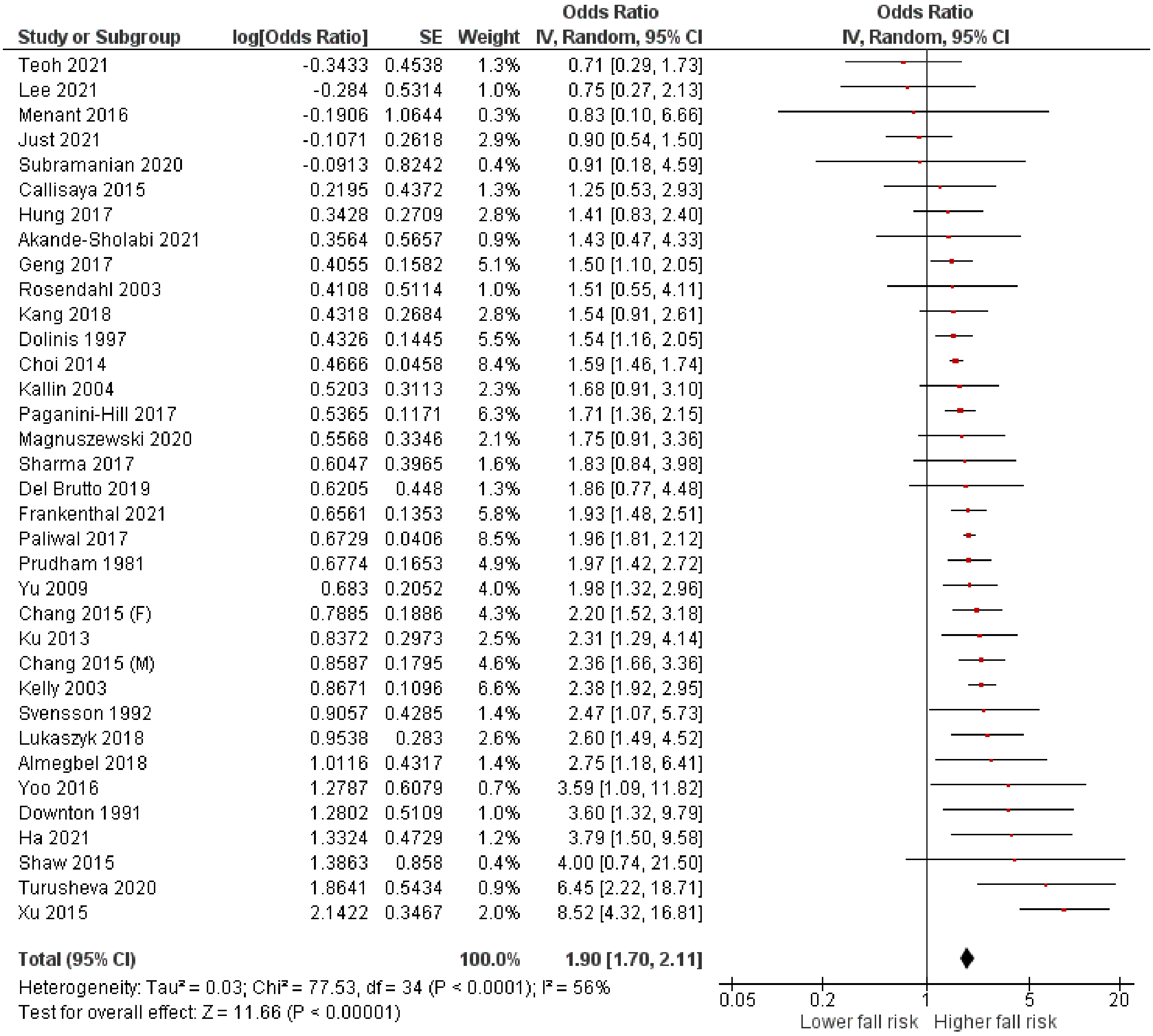

eFigure 2. Association between stroke and falls (unadjusted OR)

Study or Subgroup	log[Odds Ratio]	SE	Weight	Odds Ratio IV, Random, 95% CI
Mader 1987	-0.6466	0.5063	2.1%	0.52 [0.19, 1.41]
Hartog 2015	-0.478	0.3877	3.1%	0.62 [0.29, 1.33]
Chan 1997	-0.4111	0.4976	2.2%	0.66 [0.25, 1.76]
Campbell 1989 (M)	-0.3429	0.3272	3.8%	0.71 [0.37, 1.35]
Aydin 2017	-0.0402	0.2954	4.2%	0.96 [0.54, 1.71]
Almegbel 2018	0	0.1163	7.6%	1.00 [0.80, 1.26]
Lawlor 2003	0.038	0.1101	7.8%	1.04 [0.84, 1.29]
Wong 2014	0.1386	0.225	5.5%	1.15 [0.74, 1.79]
Luukinen 1996	0.2355	0.241	5.2%	1.27 [0.79, 2.03]
Magnuszewski 2020	0.2428	0.3029	4.1%	1.27 [0.70, 2.31]
Kocyigit 2020	0.3322	0.1986	6.0%	1.39 [0.94, 2.06]
Miu 1997	0.3418	0.3314	3.7%	1.41 [0.74, 2.69]
Shaw 2015	0.3773	0.6329	1.5%	1.46 [0.42, 5.04]
Campbell 1989 (F)	0.3857	0.2055	5.8%	1.47 [0.98, 2.20]
Kocyigit 2021	0.458	0.1675	6.6%	1.58 [1.14, 2.20]
Rutan 1992	0.5477	0.1894	6.2%	1.73 [1.19, 2.51]
Yu 2009	0.6084	0.2664	4.7%	1.84 [1.09, 3.10]
Van der Velde 2007 (a)	0.619	0.4191	2.8%	1.86 [0.82, 4.22]
Susman 1989	0.7541	0.6047	1.6%	2.13 [0.65, 6.95]
Menant 2016	0.8109	0.384	3.1%	2.25 [1.06, 4.78]
Soysal 2016	0.9415	0.2486	5.0%	2.56 [1.57, 4.17]
Saedon 2016	1.0613	0.3146	4.0%	2.89 [1.56, 5.35]
Turusheva 2020	1.0633	0.8581	0.9%	2.90 [0.54, 15.57]
Heitterachi 2002	1.5198	0.8318	0.9%	4.57 [0.90, 23.34]
McDonald 2017	1.564	0.5734	1.7%	4.78 [1.55, 14.70]
**Total (95% CI)**			**100.0%**	**1.39 [1.18, 1.64]**

Heterogeneity: $Tau^2 = 0.08$; $Chi^2 = 54.26$, df = 24 (P = 0.0004); $I^2 = 56\%$
Test for overall effect: Z = 3.91 (P < 0.0001)

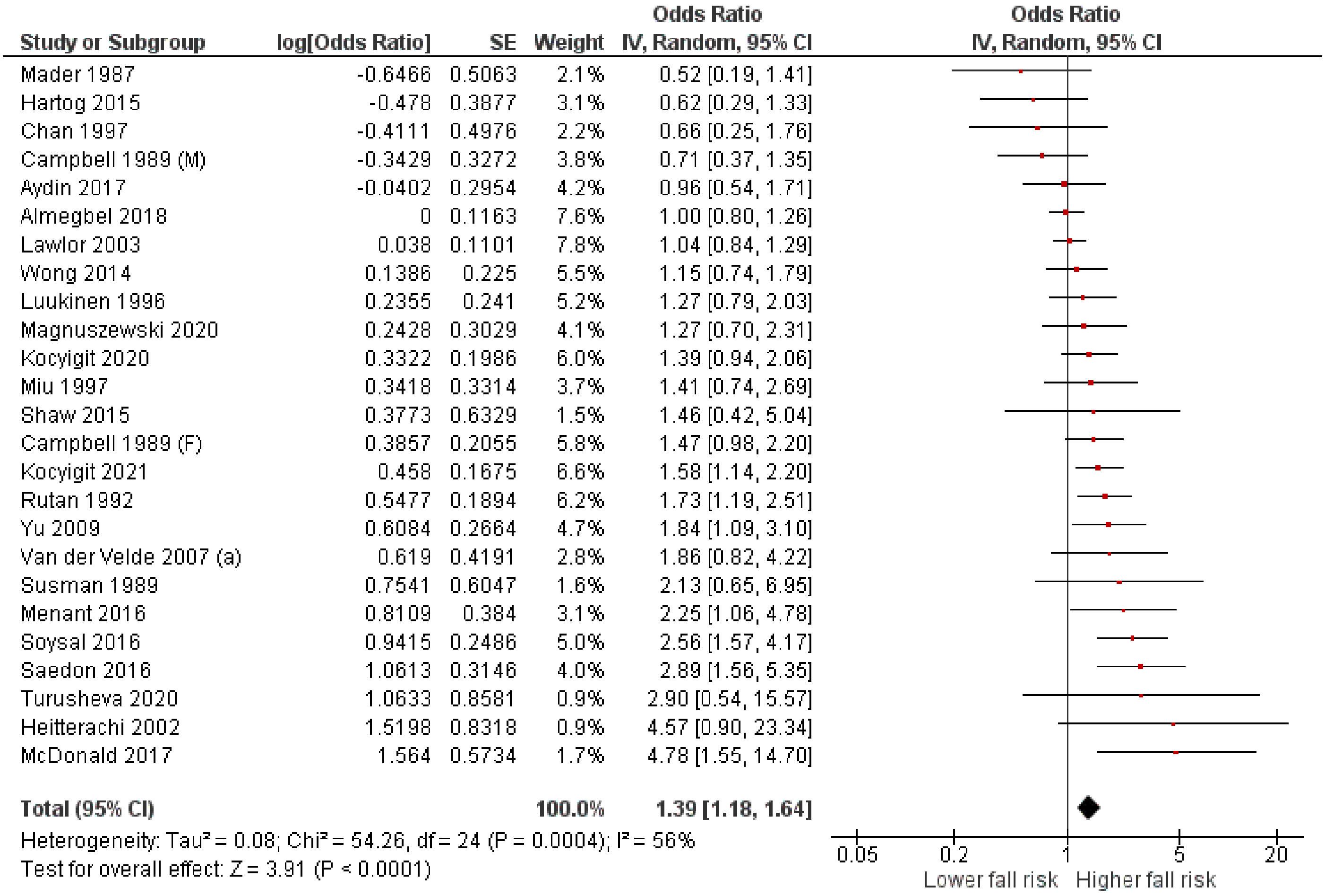

eFigure 3. Association between orthostatic hypotension and falls (unadjusted OR)

Study or Subgroup	log[Odds Ratio]	SE	Weight	Odds Ratio IV, Random, 95% CI
Menant 2016	-0.6539	0.7481	0.3%	0.52 [0.12, 2.25]
Teoh 2021	-0.3433	0.4538	0.8%	0.71 [0.29, 1.73]
Svensson 1992	-0.2984	0.3027	1.6%	0.74 [0.41, 1.34]
Jansen 2015 (a)	-0.0202	0.1365	5.2%	0.98 [0.75, 1.28]
Goh 2017	-0.0186	0.282	1.8%	0.98 [0.56, 1.71]
Turusheva 2020	0.0591	0.6721	0.4%	1.06 [0.28, 3.96]
Almegbel 2018	0.0746	0.1164	6.2%	1.08 [0.86, 1.35]
Wong 2014	0.075	0.3208	1.4%	1.08 [0.57, 2.02]
Magnuszewski 2020	0.1511	0.3523	1.2%	1.16 [0.58, 2.32]
Chang 2015 (M)	0.1655	0.1	7.1%	1.18 [0.97, 1.44]
Choi 2014	0.2191	0.0428	10.9%	1.24 [1.14, 1.35]
Frankenthal 2021	0.2319	0.1157	6.2%	1.26 [1.01, 1.58]
Downton 1991	0.2479	0.2988	1.6%	1.28 [0.71, 2.30]
Kang 2018	0.2776	0.2429	2.3%	1.32 [0.82, 2.12]
Mitchell 2013	0.2852	0.0877	7.9%	1.33 [1.12, 1.58]
Kallin 2004	0.3007	0.2906	1.7%	1.35 [0.76, 2.39]
Sibley 2014	0.3519	0.0446	10.8%	1.42 [1.30, 1.55]
Paliwal 2017	0.392	0.032	11.5%	1.48 [1.39, 1.58]
Ku 2013	0.3988	0.1984	3.2%	1.49 [1.01, 2.20]
Chang 2015 (F)	0.5128	0.0973	7.3%	1.67 [1.38, 2.02]
Saedon 2016	0.5462	0.4712	0.7%	1.73 [0.69, 4.35]
Lawlor 2003	0.5988	0.109	6.6%	1.82 [1.47, 2.25]
Xu 2015	0.6487	0.2612	2.1%	1.91 [1.15, 3.19]
Gamage 2019	1.1314	0.3704	1.1%	3.10 [1.50, 6.41]
Shaw 2015	2.3503	1.509	0.1%	10.49 [0.54, 201.92]
**Total (95% CI)**			**100.0%**	**1.34 [1.23, 1.45]**

Heterogeneity: Tau² = 0.01; Chi² = 54.46, df = 24 (P = 0.0004); I² = 56%
Test for overall effect: Z = 7.05 (P < 0.00001)

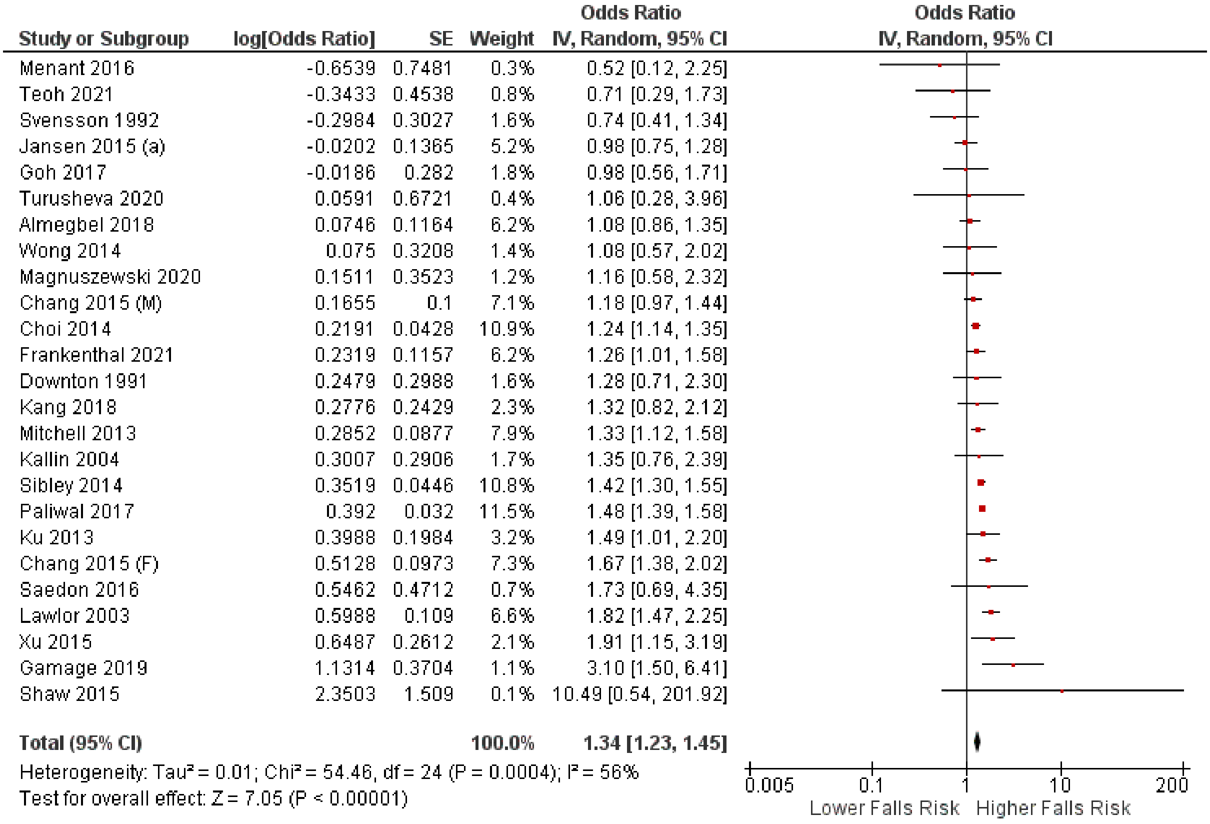

eFigure 4. Association between coronary artery disease and falls (unadjusted OR)

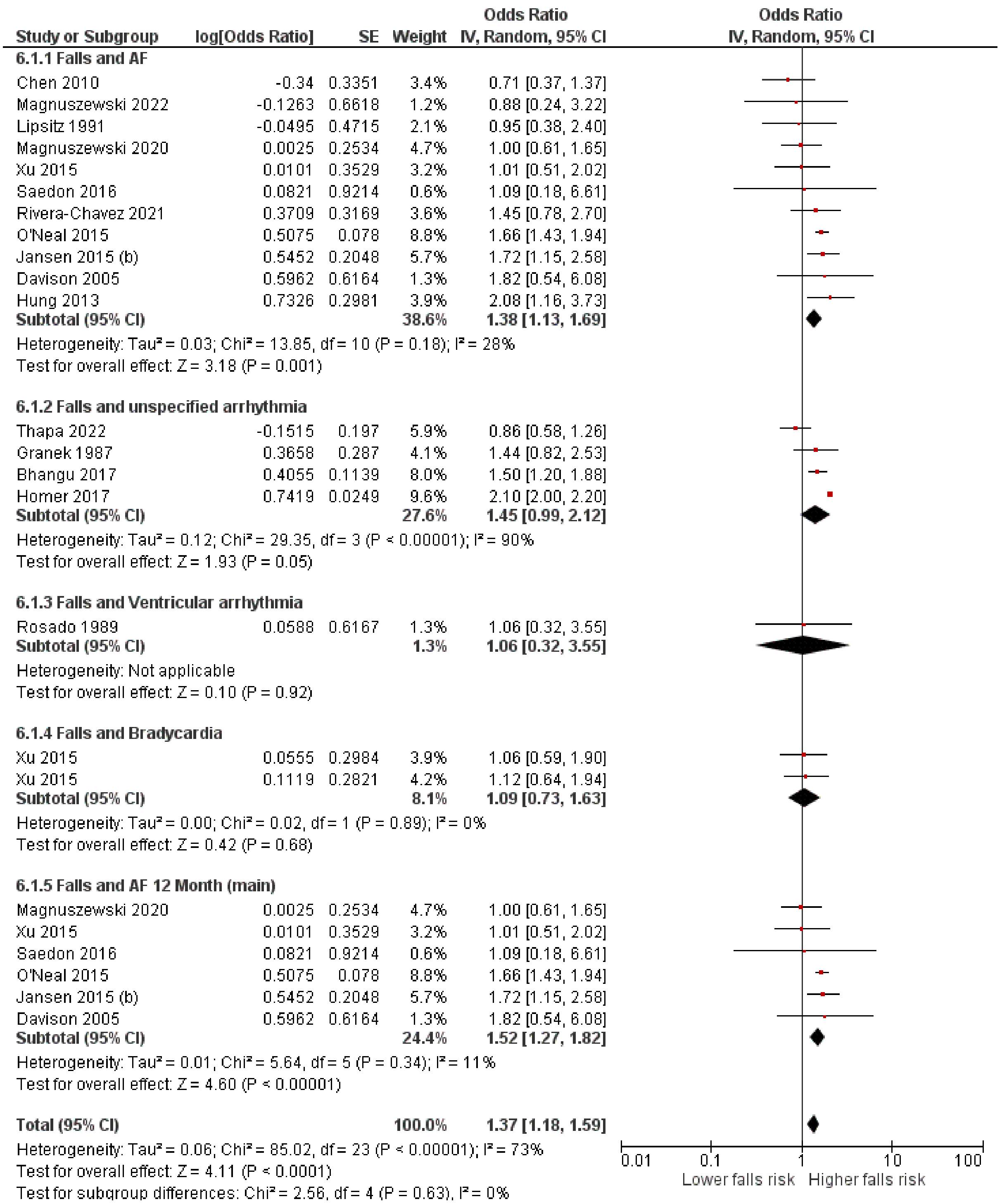

eFigure 5. Association between atrial fibrillation and falls (unadjusted OR)

Study or Subgroup	log[Odds Ratio]	SE	Weight	Odds Ratio IV, Random, 95% CI
Saedon 2016	-0.1054	0.6826	2.6%	0.90 [0.24, 3.43]
Just 2021	0.2506	0.1586	48.8%	1.28 [0.94, 1.75]
Magnuszewski 2020	0.3275	0.2196	25.4%	1.39 [0.90, 2.13]
Jansen 2015 (a)	0.5247	0.238	21.7%	1.69 [1.06, 2.69]
Shaw 2015	0.7621	0.8962	1.5%	2.14 [0.37, 12.41]
**Total (95% CI)**			**100.0%**	**1.39 [1.12, 1.72]**

Heterogeneity: Tau² = 0.00; Chi² = 1.56, df = 4 (P = 0.82); I² = 0%
Test for overall effect: Z = 2.96 (P = 0.003)

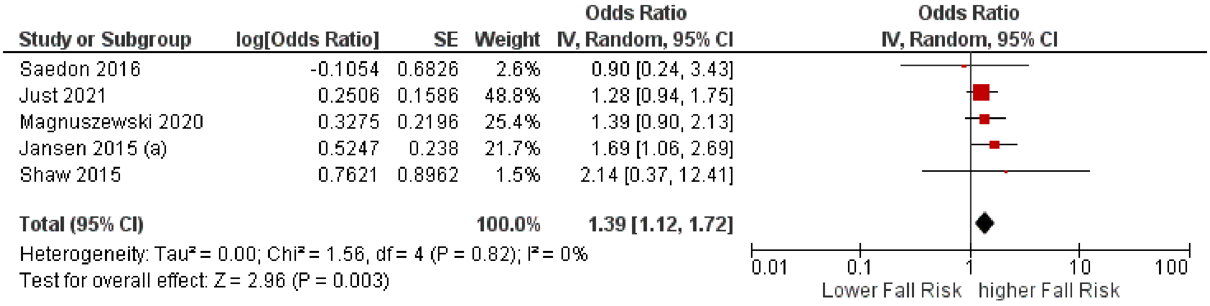

eFigure 6. Association between heart failure and falls (unadjusted OR)

Study or Subgroup	log[Odds Ratio]	SE	Weight	Odds Ratio IV, Random, 95% CI
Hung 2017	0.0766	0.4491	21.8%	1.08 [0.45, 2.60]
Turusheva 2020	0.1117	0.6735	11.5%	1.12 [0.30, 4.19]
Magnuszewski 2020	0.6202	0.2622	41.7%	1.86 [1.11, 3.11]
Ha 2021	1.2413	0.4064	25.1%	3.46 [1.56, 7.67]
**Total (95% CI)**			**100.0%**	**1.82 [1.12, 2.95]**

Heterogeneity: Tau² = 0.08; Chi² = 4.37, df = 3 (P = 0.22); I² = 31%
Test for overall effect: Z = 2.43 (P = 0.02)

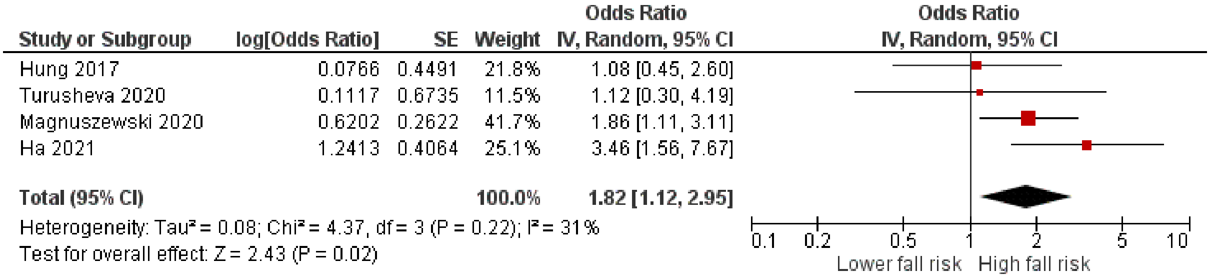

eFigure 7. Association between peripheral artery disease and falls (unadjusted OR)

Study or Subgroup	log[Odds Ratio]	SE	Weight	Odds Ratio IV, Random, 95% CI
Swanenburg 2010	-0.3415	0.3771	14.9%	0.71 [0.34, 1.49]
Song 2021	0.0604	0.167	38.7%	1.06 [0.77, 1.47]
Sagawa 2018	0.3639	0.1269	46.4%	1.44 [1.12, 1.85]
**Total (95% CI)**			**100.0%**	**1.15 [0.83, 1.60]**

Heterogeneity: Tau² = 0.04; Chi² = 4.41, df = 2 (P = 0.11); I² = 55%
Test for overall effect: Z = 0.85 (P = 0.39)

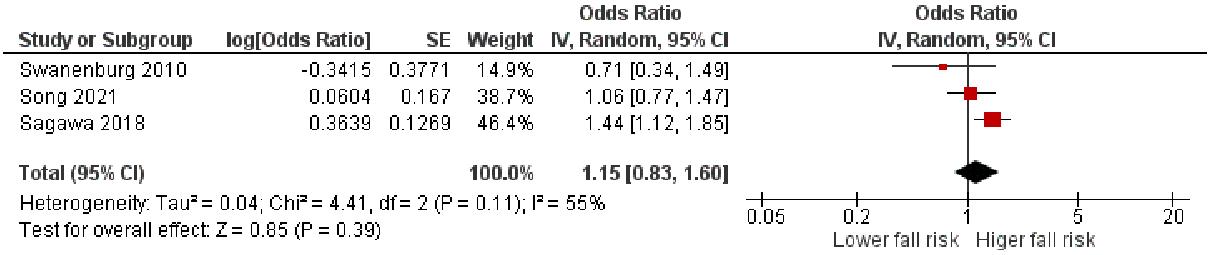

eFigure 8. Association between low blood pressure and falls (unadjusted OR)

Study or Subgroup	log[Odds Ratio]	SE	Weight	Odds Ratio IV, Random, 95% CI
**1.3.1 50-64 years**				
Jansen 2015 (a)	0.0583	0.0559	8.3%	1.06 [0.95, 1.18]
Bhangu 2017	0.077	0.0601	7.9%	1.08 [0.96, 1.22]
Tsai 2021	0.1989	0.0914	5.5%	1.22 [1.02, 1.46]
Lee 2021	0.361	0.2951	0.9%	1.43 [0.80, 2.56]
Liao 2012	0.4074	0.1557	2.7%	1.50 [1.11, 2.04]
**Subtotal (95% CI)**			**25.4%**	**1.14 [1.03, 1.27]**
Heterogeneity: Tau² = 0.00; Chi² = 6.47, df = 4 (P = 0.17); I² = 38%				
Test for overall effect: Z = 2.59 (P = 0.010)				
**1.3.2 65-79 years**				
Turusheva 2020	-1.65	1.0475	0.1%	0.19 [0.02, 1.50]
Akande-Sholabi 2021	-0.0805	0.2091	1.7%	0.92 [0.61, 1.39]
Margolis 2019	-0.0728	0.1259	3.7%	0.93 [0.73, 1.19]
Just 2021	-0.0169	0.1154	4.2%	0.98 [0.78, 1.23]
Kao 2012	-0.0101	0.2306	1.4%	0.99 [0.63, 1.56]
Kelly 2003	-0.0101	0.0543	8.4%	0.99 [0.89, 1.10]
Lawlor 2003	0.0404	0.0838	6.0%	1.04 [0.88, 1.23]
Choi 2014	0.0676	0.0236	11.0%	1.07 [1.02, 1.12]
Kamali 2022	0.1537	0.2482	1.2%	1.17 [0.72, 1.90]
Kang 2018	0.207	0.2069	1.7%	1.23 [0.82, 1.85]
Del Brutto 2019	0.2246	0.2247	1.5%	1.25 [0.81, 1.94]
Saedon 2016	0.2396	0.2485	1.2%	1.27 [0.78, 2.07]
Almegbel 2018	0.2442	0.1166	4.1%	1.28 [1.02, 1.60]
Sharma 2017	0.2631	0.2558	1.2%	1.30 [0.79, 2.15]
Teoh 2021	0.2717	0.1322	3.5%	1.31 [1.01, 1.70]
Zia 2015	0.3047	0.2141	1.6%	1.36 [0.89, 2.06]
Yu 2009	0.3937	0.1344	3.4%	1.48 [1.14, 1.93]
Ha 2021	0.5423	0.2098	1.7%	1.72 [1.14, 2.59]
Chan 1997	0.5786	0.2662	1.1%	1.78 [1.06, 3.01]
Gamage 2019	0.7885	0.2684	1.1%	2.20 [1.30, 3.72]
**Subtotal (95% CI)**			**59.9%**	**1.15 [1.06, 1.25]**
Heterogeneity: Tau² = 0.01; Chi² = 36.67, df = 19 (P = 0.009); I² = 48%				
Test for overall effect: Z = 3.43 (P = 0.0006)				
**1.3.3 80 years+**				
Hartog 2015	-0.5621	0.463	0.4%	0.57 [0.23, 1.41]
Wong 2014	-0.2737	0.1873	2.0%	0.76 [0.53, 1.10]
Svensson 1992	-0.0481	0.1606	2.6%	0.95 [0.70, 1.31]
Xu 2015	0.0235	0.2256	1.5%	1.02 [0.66, 1.59]
Paganini-Hill 2017	0.1139	0.1022	4.9%	1.12 [0.92, 1.37]
Magnuszewski 2020	0.1231	0.2701	1.1%	1.13 [0.67, 1.92]
Ku 2013	0.3001	0.1739	2.3%	1.35 [0.96, 1.90]
**Subtotal (95% CI)**			**14.7%**	**1.03 [0.88, 1.21]**
Heterogeneity: Tau² = 0.01; Chi² = 7.65, df = 6 (P = 0.26); I² = 22%				
Test for overall effect: Z = 0.41 (P = 0.68)				
**Total (95% CI)**			**100.0%**	**1.13 [1.07, 1.20]**
Heterogeneity: Tau² = 0.01; Chi² = 51.69, df = 31 (P = 0.01); I² = 40%				
Test for overall effect: Z = 4.13 (P < 0.0001)				
Test for subgroup differences: Chi² = 1.60, df = 2 (P = 0.45), I² = 0%				

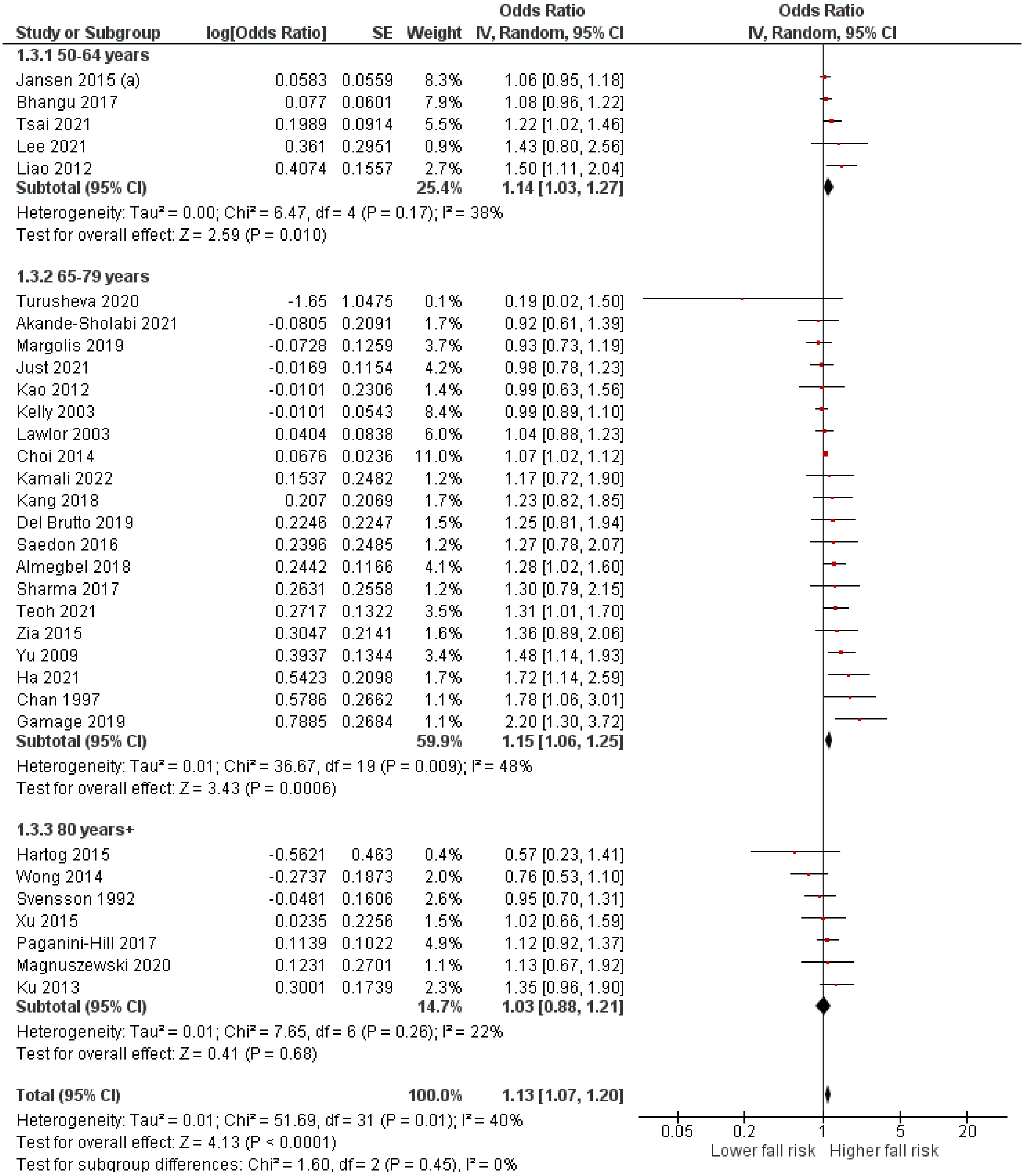

eFigure 9. Association between hypertension and falls (unadjusted OR) stratified by age

Study or Subgroup	log[Odds Ratio]	SE	Weight	Odds Ratio IV, Random, 95% CI
**7.3.1 50-64 years**				
**Subtotal (95% CI)**				**Not estimable**
Heterogeneity: Not applicable				
Test for overall effect: Not applicable				
**7.3.2 65-79 years**				
Teoh 2021	-0.3433	0.4538	2.1%	0.71 [0.29, 1.73]
Lee 2021	-0.284	0.5314	1.6%	0.75 [0.27, 2.13]
Menant 2016	-0.1906	1.0644	0.5%	0.83 [0.10, 6.66]
Just 2021	-0.1071	0.2618	4.4%	0.90 [0.54, 1.50]
Subramanian 2020	-0.0913	0.8242	0.8%	0.91 [0.18, 4.59]
Callisaya 2015	0.2195	0.4372	2.2%	1.25 [0.53, 2.93]
Akande-Sholabi 2021	0.3564	0.5657	1.5%	1.43 [0.47, 4.33]
Geng 2017	0.4055	0.1582	6.6%	1.50 [1.10, 2.05]
Kang 2018	0.4318	0.2684	4.3%	1.54 [0.91, 2.61]
Choi 2014	0.4666	0.0458	9.0%	1.59 [1.46, 1.74]
Sharma 2017	0.6047	0.3965	2.6%	1.83 [0.84, 3.98]
Del Brutto 2019	0.6205	0.448	2.2%	1.86 [0.77, 4.48]
Frankenthal 2021	0.6561	0.1353	7.1%	1.93 [1.48, 2.51]
Yu 2009	0.683	0.2052	5.5%	1.98 [1.32, 2.96]
Kelly 2003	0.8671	0.1096	7.8%	2.38 [1.92, 2.95]
Lukaszyk 2018	0.9538	0.283	4.0%	2.60 [1.49, 4.52]
Almegbel 2018	1.0116	0.4317	2.3%	2.75 [1.18, 6.41]
Ha 2021	1.3324	0.4729	2.0%	3.79 [1.50, 9.58]
Turusheva 2020	1.8641	0.5434	1.6%	6.45 [2.22, 18.71]
**Subtotal (95% CI)**			**67.9%**	**1.75 [1.48, 2.06]**
Heterogeneity: Tau^2 = 0.05; Chi^2 = 39.62, df = 18 (P = 0.002); I^2 = 55%				
Test for overall effect: Z = 6.52 (P < 0.00001)				
**7.3.3 80+ years**				
Hung 2017	0.3428	0.2709	4.2%	1.41 [0.83, 2.40]
Rosendahl 2003	0.4108	0.5114	1.8%	1.51 [0.55, 4.11]
Kallin 2004	0.5203	0.3113	3.6%	1.68 [0.91, 3.10]
Paganini-Hill 2017	0.5365	0.1171	7.6%	1.71 [1.36, 2.15]
Magnuszewski 2020	0.5568	0.3346	3.3%	1.75 [0.91, 3.36]
Ku 2013	0.8372	0.2973	3.8%	2.31 [1.29, 4.14]
Svensson 1992	0.9057	0.4285	2.3%	2.47 [1.07, 5.73]
Downton 1991	1.2802	0.5109	1.8%	3.60 [1.32, 9.79]
Shaw 2015	1.3863	0.858	0.7%	4.00 [0.74, 21.50]
Xu 2015	2.1422	0.3467	3.1%	8.52 [4.32, 16.81]
**Subtotal (95% CI)**			**32.1%**	**2.27 [1.62, 3.16]**
Heterogeneity: Tau^2 = 0.16; Chi^2 = 24.13, df = 9 (P = 0.004); I^2 = 63%				
Test for overall effect: Z = 4.81 (P < 0.00001)				
**Total (95% CI)**			**100.0%**	**1.87 [1.62, 2.17]**
Heterogeneity: Tau^2 = 0.06; Chi^2 = 66.36, df = 28 (P < 0.0001); I^2 = 58%				
Test for overall effect: Z = 8.35 (P < 0.00001)				
Test for subgroup differences: Chi^2 = 1.88, df = 1 (P = 0.17), I^2 = 46.8%				

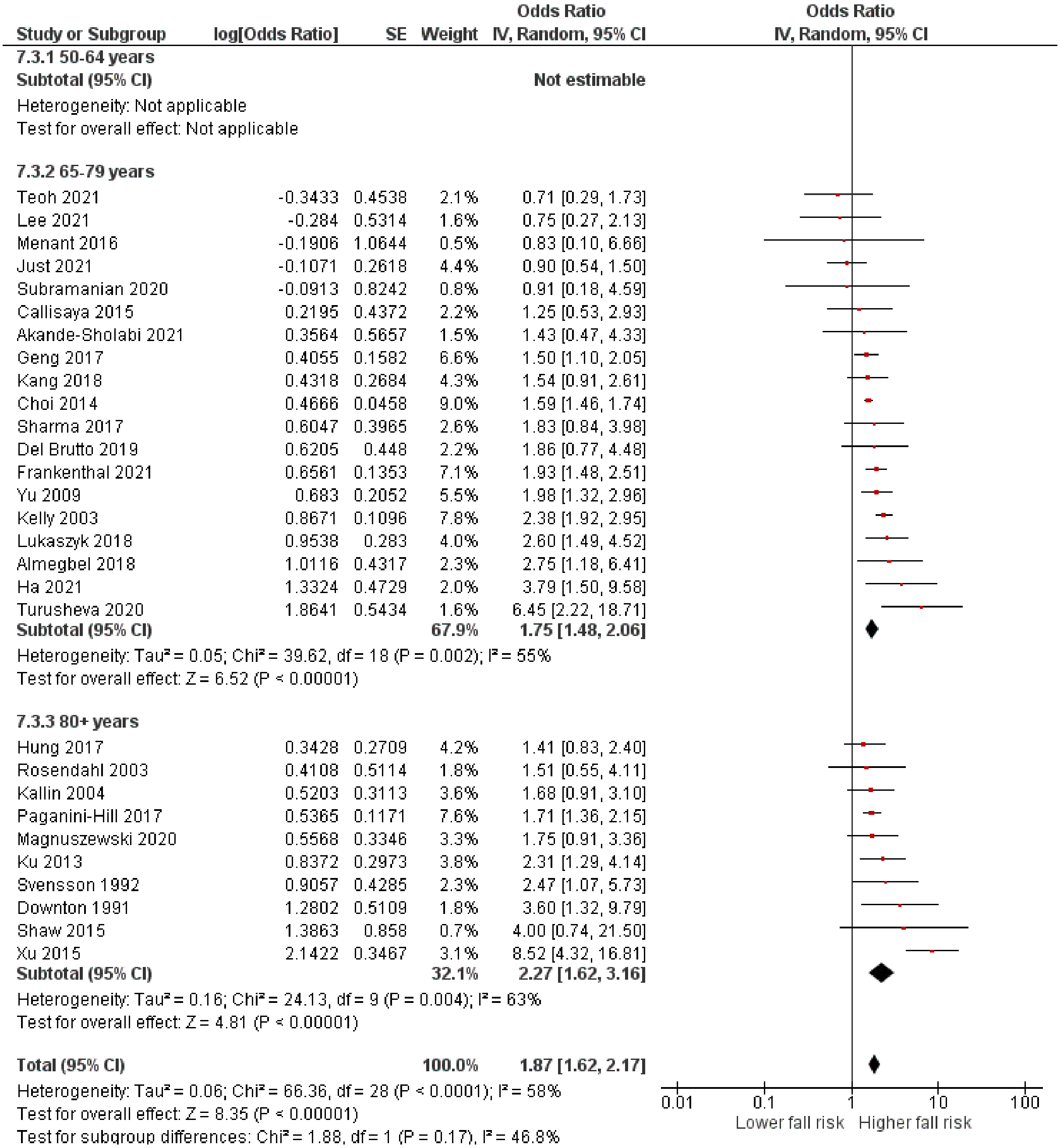

eFigure 10. Association between stroke and falls (unadjusted OR) stratified by age

Study or Subgroup	log[Odds Ratio]	SE	Weight	Odds Ratio IV, Random, 95% CI
**2.4.2 65-80 years**				
Mader 1987	-0.6466	0.5063	2.4%	0.52 [0.19, 1.41]
Chan 1997	-0.4111	0.4976	2.5%	0.66 [0.25, 1.76]
Almegbel 2018	0	0.1163	9.3%	1.00 [0.80, 1.26]
Lawlor 2003	0.038	0.1101	9.4%	1.04 [0.84, 1.29]
Luukinen 1996	0.2355	0.241	6.1%	1.27 [0.79, 2.03]
Kocyigit 2020	0.3322	0.1986	7.1%	1.39 [0.94, 2.06]
Miu 1997	0.3418	0.3314	4.4%	1.41 [0.74, 2.69]
Kocyigit 2021	0.458	0.1675	7.9%	1.58 [1.14, 2.20]
Rutan 1992	0.5477	0.1894	7.4%	1.73 [1.19, 2.51]
Yu 2009	0.6084	0.2664	5.6%	1.84 [1.09, 3.10]
Van der Velde 2007 (a)	0.619	0.4191	3.2%	1.86 [0.82, 4.22]
Susman 1989	0.7541	0.6047	1.8%	2.13 [0.65, 6.95]
Menant 2016	0.8109	0.384	3.6%	2.25 [1.06, 4.78]
Soysal 2016	0.9415	0.2486	5.9%	2.56 [1.57, 4.17]
Saedon 2016	1.0613	0.3146	4.7%	2.89 [1.56, 5.35]
Turusheva 2020	1.0633	0.8581	1.0%	2.90 [0.54, 15.57]
Heitterachi 2002	1.5198	0.8318	1.0%	4.57 [0.90, 23.34]
**Subtotal (95% CI)**			**83.4%**	**1.50 [1.23, 1.83]**
Heterogeneity: Tau² = 0.08; Chi² = 40.17, df = 16 (P = 0.0007); I² = 60%				
Test for overall effect: Z = 3.99 (P < 0.0001)				
**2.4.3 80+ years**				
Hartog 2015	-0.478	0.3877	3.6%	0.62 [0.29, 1.33]
Wong 2014	0.1386	0.225	6.5%	1.15 [0.74, 1.79]
Magnuszewski 2020	0.2428	0.3029	4.9%	1.27 [0.70, 2.31]
Shaw 2015	0.3773	0.6329	1.7%	1.46 [0.42, 5.04]
**Subtotal (95% CI)**			**16.6%**	**1.08 [0.79, 1.48]**
Heterogeneity: Tau² = 0.00; Chi² = 2.65, df = 3 (P = 0.45); I² = 0%				
Test for overall effect: Z = 0.50 (P = 0.62)				
**Total (95% CI)**			**100.0%**	**1.41 [1.19, 1.68]**
Heterogeneity: Tau² = 0.07; Chi² = 44.29, df = 20 (P = 0.001); I² = 55%				
Test for overall effect: Z = 3.87 (P = 0.0001)				
Test for subgroup differences: Chi² = 3.03, df = 1 (P = 0.08), I² = 67.0%				

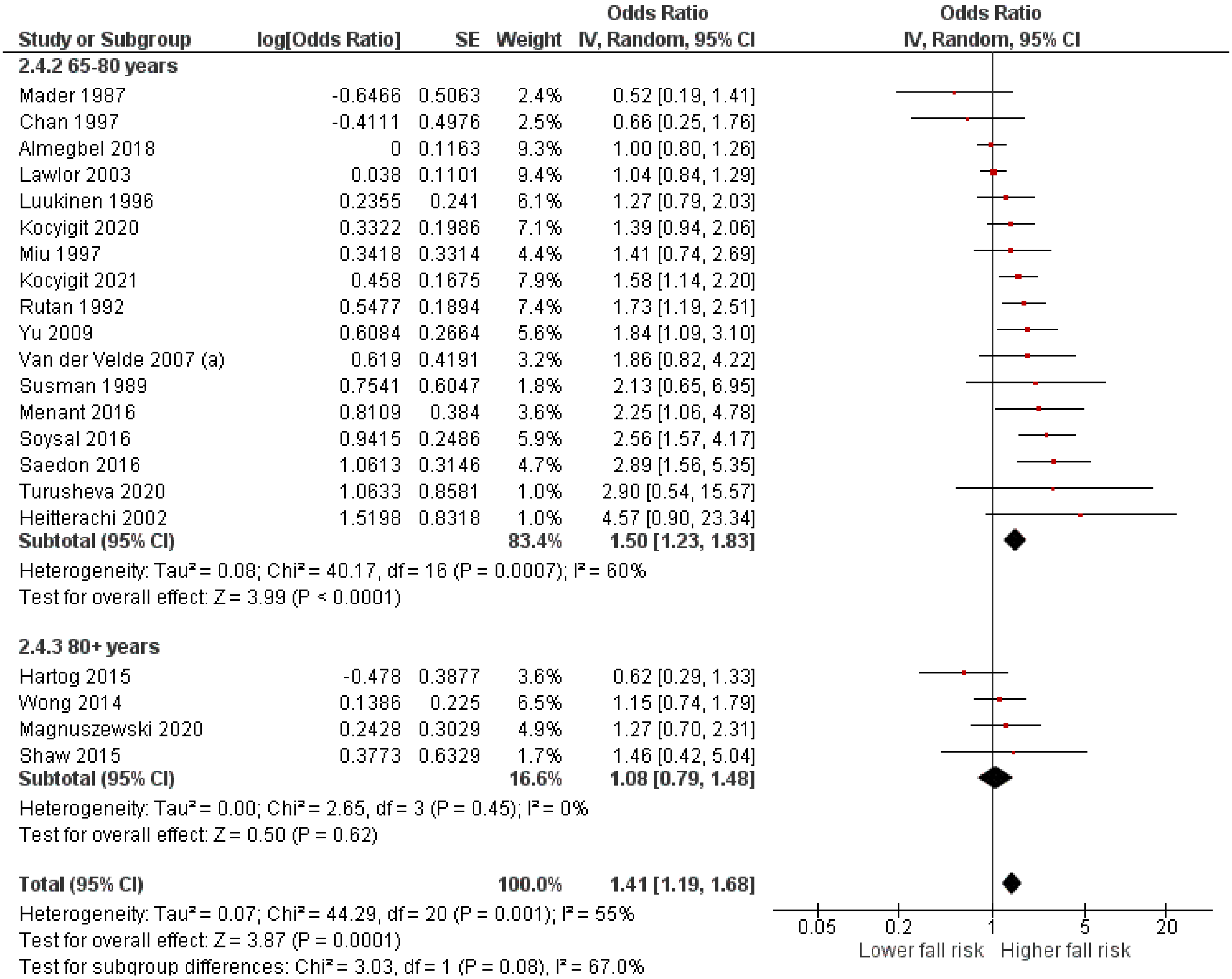

eFigure 11. Association between orthostatic hypotension and falls (unadjusted OR) stratified by age

Study or Subgroup	log[Odds Ratio]	SE	Weight	Odds Ratio IV, Random, 95% CI
**4.3.1 50-64 years**				
**Subtotal (95% CI)**				**Not estimable**
Heterogeneity: Not applicable				
Test for overall effect: Not applicable				
**4.3.2 65-79 years**				
Menant 2016	-0.6539	0.7481	0.9%	0.52 [0.12, 2.25]
Teoh 2021	-0.3433	0.4538	2.2%	0.71 [0.29, 1.73]
Jansen 2015 (a)	-0.0202	0.1365	10.7%	0.98 [0.75, 1.28]
Turusheva 2020	0.0591	0.6721	1.1%	1.06 [0.28, 3.96]
Almegbel 2018	0.0814	21.6494	0.0%	1.08 [0.00, 2.906E18]
Choi 2014	0.2191	0.0428	16.5%	1.24 [1.14, 1.35]
Frankenthal 2021	0.2319	0.1157	12.0%	1.26 [1.01, 1.58]
Kang 2018	0.2776	0.2429	5.8%	1.32 [0.82, 2.12]
Saedon 2016	0.5462	0.4712	2.0%	1.73 [0.69, 4.35]
Lawlor 2003	0.5988	0.109	12.5%	1.82 [1.47, 2.25]
Gamage 2019	1.1314	0.3704	3.1%	3.10 [1.50, 6.41]
**Subtotal (95% CI)**			**66.8%**	**1.32 [1.10, 1.59]**
Heterogeneity: Tau² = 0.04; Chi² = 24.02, df = 9 (P = 0.004); I² = 63%				
Test for overall effect: Z = 2.94 (P = 0.003)				
**4.3.3 80+ years**				
Svensson 1992	-0.2984	0.3027	4.2%	0.74 [0.41, 1.34]
Wong 2014	0.075	0.3208	3.9%	1.08 [0.57, 2.02]
Magnuszewski 2020	0.1511	0.3523	3.3%	1.16 [0.58, 2.32]
Downton 1991	0.2479	0.2988	4.3%	1.28 [0.71, 2.30]
Kallin 2004	0.3007	0.2906	4.5%	1.35 [0.76, 2.39]
Ku 2013	0.3988	0.1984	7.5%	1.49 [1.01, 2.20]
Xu 2015	0.6487	0.2612	5.3%	1.91 [1.15, 3.19]
Shaw 2015	2.3503	1.509	0.2%	10.49 [0.54, 201.92]
**Subtotal (95% CI)**			**33.2%**	**1.31 [1.04, 1.65]**
Heterogeneity: Tau² = 0.02; Chi² = 8.44, df = 7 (P = 0.30); I² = 17%				
Test for overall effect: Z = 2.30 (P = 0.02)				
**Total (95% CI)**			**100.0%**	**1.32 [1.14, 1.51]**
Heterogeneity: Tau² = 0.03; Chi² = 32.54, df = 17 (P = 0.01); I² = 48%				
Test for overall effect: Z = 3.84 (P = 0.0001)				
Test for subgroup differences: Chi² = 0.00, df = 1 (P = 0.95), I² = 0%				

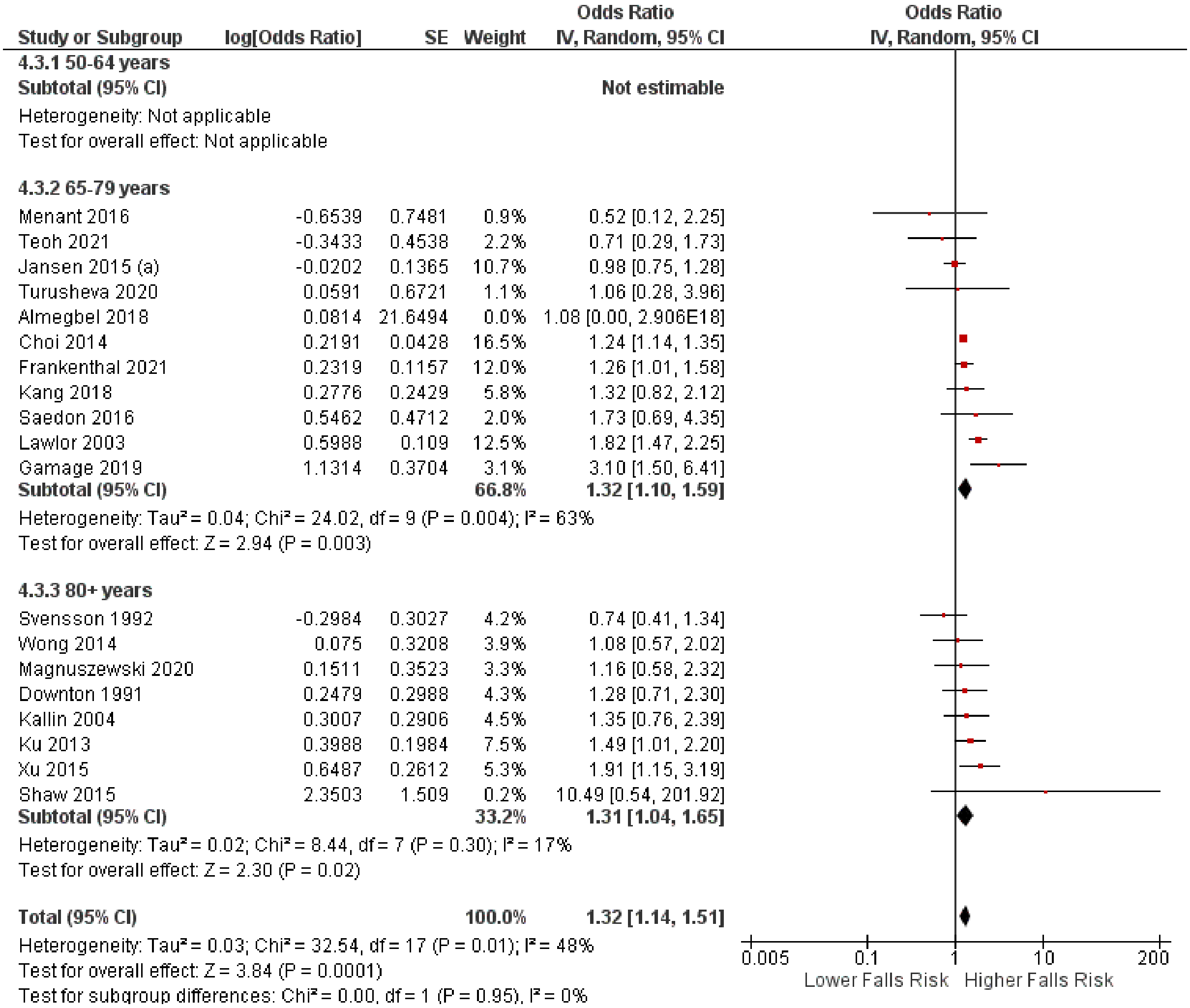

eFigure 12. Association between coronary artery disease and falls (unadjusted OR) stratified by age

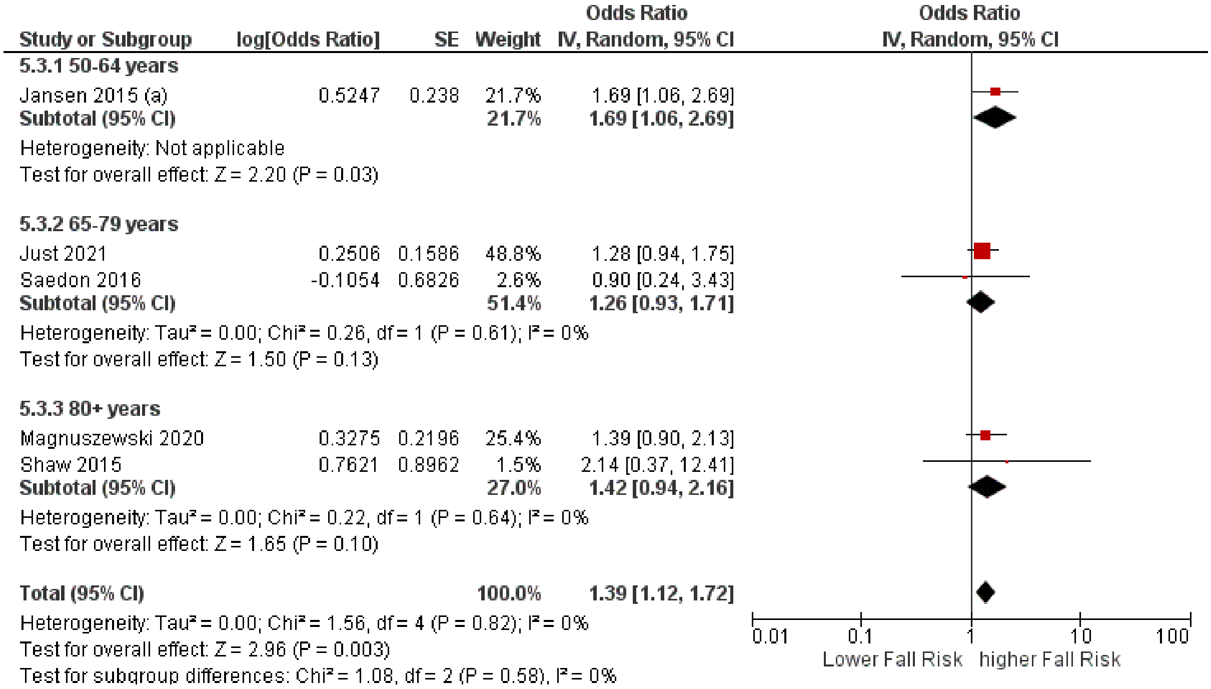

eFigure 13. Association between heart failure and falls (unadjusted OR) stratified by age

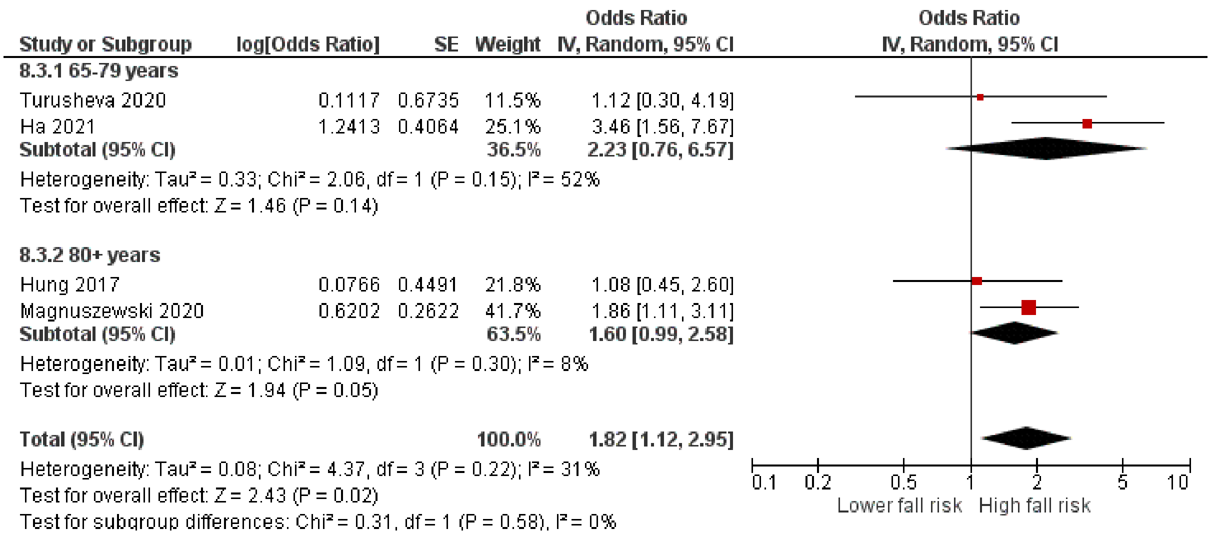

eFigure 14. Association between peripheral artery disease and falls (unadjusted OR) stratified by age

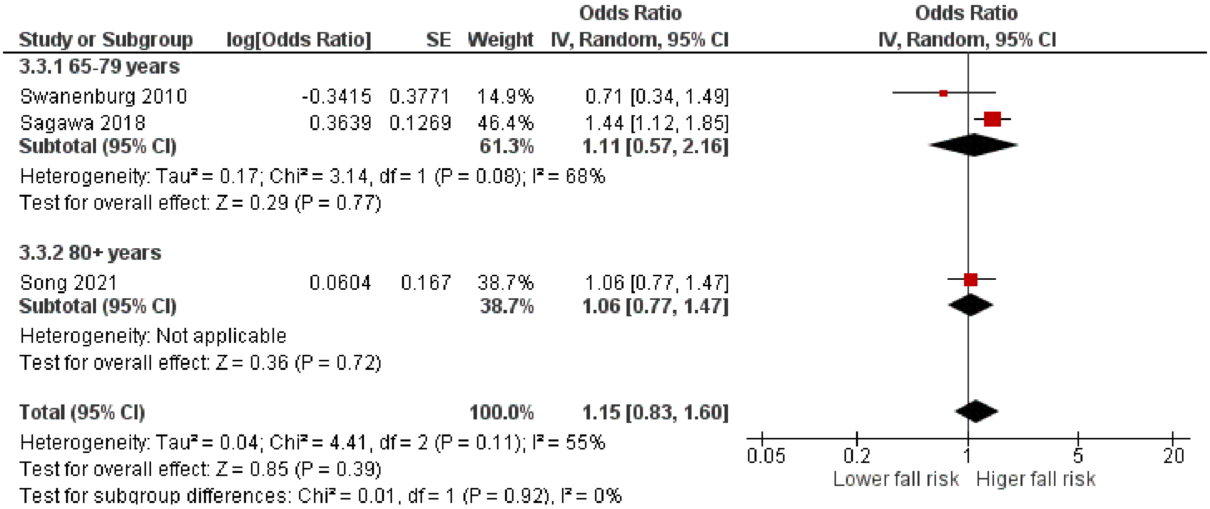

eFigure 15. Association between low blood pressure and falls (unadjusted OR) stratified by age

Study or Subgroup	log[Odds Ratio]	SE	Weight	Odds Ratio IV, Random, 95% CI
**1.4.1 Community**				
Turusheva 2020	-1.65	1.0475	0.1%	0.19 [0.02, 1.50]
Wong 2014	-0.2737	0.1873	1.4%	0.76 [0.53, 1.10]
Margolis 2019	-0.0728	0.1259	2.6%	0.93 [0.73, 1.19]
Svensson 1992	-0.0481	0.1606	1.8%	0.95 [0.70, 1.31]
Just 2021	-0.0169	0.1154	2.9%	0.98 [0.78, 1.23]
Kao 2012	-0.0101	0.2306	1.0%	0.99 [0.63, 1.56]
Xu 2015	0.0235	0.2256	1.0%	1.02 [0.66, 1.59]
Lawlor 2003	0.0404	0.0838	4.3%	1.04 [0.88, 1.23]
Jansen 2015 (a)	0.0583	0.0559	6.2%	1.06 [0.95, 1.18]
Choi 2014	0.0676	0.0236	8.6%	1.07 [1.02, 1.12]
Bhangu 2017	0.077	0.0601	5.9%	1.08 [0.96, 1.22]
Prudham 1981	0.0876	0.1092	3.1%	1.09 [0.88, 1.35]
Goh 2017	0.0983	0.141	2.2%	1.10 [0.84, 1.45]
Mitchell 2013	0.1044	0.0688	5.2%	1.11 [0.97, 1.27]
Chang 2015 (M)	0.131	0.093	3.8%	1.14 [0.95, 1.37]
Kamali 2022	0.1537	0.2482	0.8%	1.17 [0.72, 1.90]
Yoo 2016	0.1793	0.287	0.6%	1.20 [0.68, 2.10]
Chang 2015 (F)	0.1906	0.0922	3.9%	1.21 [1.01, 1.45]
Tsai 2021	0.1989	0.0914	3.9%	1.22 [1.02, 1.46]
Sibley 2014	0.2022	0.0394	7.4%	1.22 [1.13, 1.32]
Kang 2018	0.207	0.2069	1.2%	1.23 [0.82, 1.85]
Del Brutto 2019	0.2246	0.2247	1.0%	1.25 [0.81, 1.94]
Almegbel 2018	0.2442	0.1166	2.9%	1.28 [1.02, 1.60]
Sharma 2017	0.2631	0.2558	0.8%	1.30 [0.79, 2.15]
Teoh 2021	0.2717	0.1322	2.4%	1.31 [1.01, 1.70]
Ku 2013	0.3001	0.1739	1.6%	1.35 [0.96, 1.90]
Lee 2021	0.361	0.2951	0.6%	1.43 [0.80, 2.56]
Brassington 2000	0.392	0.1332	2.4%	1.48 [1.14, 1.92]
Yu 2009	0.3937	0.1344	2.3%	1.48 [1.14, 1.93]
Liao 2012	0.4074	0.1557	1.9%	1.50 [1.11, 2.04]
Chan 1997	0.5786	0.2662	0.7%	1.78 [1.06, 3.01]
Gamage 2019	0.7885	0.2684	0.7%	2.20 [1.30, 3.72]
**Subtotal (95% CI)**			**85.1%**	**1.15 [1.09, 1.21]**
Heterogeneity: Tau² = 0.01; Chi² = 51.75, df = 31 (P = 0.01); I² = 40%				
Test for overall effect: Z = 5.51 (P < 0.00001)				
**1.4.2 Residential care**				
Hartog 2015	-0.5621	0.463	0.3%	0.57 [0.23, 1.41]
Paganini-Hill 2017	0.1139	0.1022	3.4%	1.12 [0.92, 1.37]
**Subtotal (95% CI)**			**3.7%**	**0.93 [0.51, 1.68]**
Heterogeneity: Tau² = 0.12; Chi² = 2.03, df = 1 (P = 0.15); I² = 51%				
Test for overall effect: Z = 0.24 (P = 0.81)				
**1.4.3 Hospital (outpatient)**				
Akande-Sholabi 2021	-0.0805	0.2091	1.1%	0.92 [0.61, 1.39]
Zia 2015	0.3047	0.2141	1.1%	1.36 [0.89, 2.06]
Ha 2021	0.5423	0.2098	1.1%	1.72 [1.14, 2.59]
**Subtotal (95% CI)**			**3.4%**	**1.29 [0.90, 1.85]**
Heterogeneity: Tau² = 0.06; Chi² = 4.50, df = 2 (P = 0.11); I² = 56%				
Test for overall effect: Z = 1.39 (P = 0.16)				
**1.4.4 Hospital (inpatient)**				
Kelly 2003	-0.0101	0.0543	6.3%	0.99 [0.89, 1.10]
Magnuszewski 2020	0.1231	0.2701	0.7%	1.13 [0.67, 1.92]
Saedon 2016	0.2396	0.2485	0.8%	1.27 [0.78, 2.07]
**Subtotal (95% CI)**			**7.8%**	**1.01 [0.91, 1.11]**
Heterogeneity: Tau² = 0.00; Chi² = 1.16, df = 2 (P = 0.56); I² = 0%				
Test for overall effect: Z = 0.11 (P = 0.91)				
**Total (95% CI)**			**100.0%**	**1.14 [1.09, 1.20]**
Heterogeneity: Tau² = 0.01; Chi² = 64.98, df = 39 (P = 0.006); I² = 40%				
Test for overall effect: Z = 5.57 (P < 0.00001)				
Test for subgroup differences: Chi² = 6.31, df = 3 (P = 0.10), I² = 52.4%				

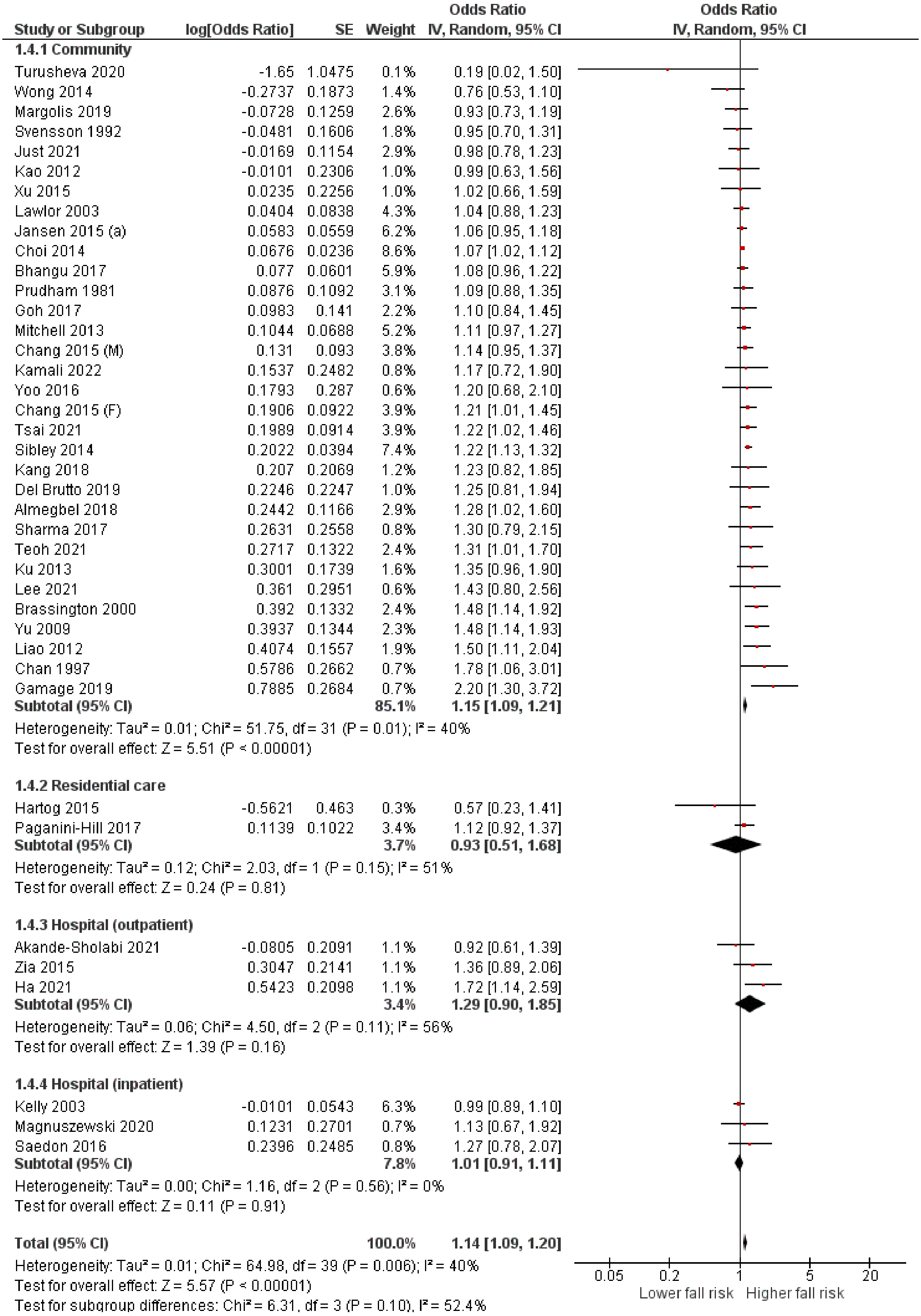

eFigure 16. Association between hypertension and falls (unadjusted OR) stratified by setting

Study or Subgroup	log[Odds Ratio]	SE	Weight	Odds Ratio IV, Random, 95% CI
**7.4.1 Community**				
Teoh 2021	-0.3433	0.4538	1.3%	0.71 [0.29, 1.73]
Lee 2021	-0.284	0.5314	1.0%	0.75 [0.27, 2.13]
Menant 2016	-0.1906	1.0644	0.3%	0.83 [0.10, 6.66]
Just 2021	-0.1071	0.2618	2.9%	0.90 [0.54, 1.50]
Subramanian 2020	-0.0913	0.8242	0.4%	0.91 [0.18, 4.59]
Callisaya 2015	0.2195	0.4372	1.3%	1.25 [0.53, 2.93]
Geng 2017	0.4055	0.1582	5.1%	1.50 [1.10, 2.05]
Kang 2018	0.4318	0.2684	2.8%	1.54 [0.91, 2.61]
Dolinis 1997	0.4326	0.1445	5.5%	1.54 [1.16, 2.05]
Choi 2014	0.4666	0.0458	8.4%	1.59 [1.46, 1.74]
Sharma 2017	0.6047	0.3965	1.6%	1.83 [0.84, 3.98]
Del Brutto 2019	0.6205	0.448	1.3%	1.86 [0.77, 4.48]
Frankenthal 2021	0.6561	0.1353	5.8%	1.93 [1.48, 2.51]
Prudham 1981	0.6774	0.1653	4.9%	1.97 [1.42, 2.72]
Yu 2009	0.683	0.2052	4.0%	1.98 [1.32, 2.96]
Chang 2015 (F)	0.7885	0.1886	4.3%	2.20 [1.52, 3.18]
Chang 2015 (M)	0.8587	0.1795	4.6%	2.36 [1.66, 3.36]
Svensson 1992	0.9057	0.4285	1.4%	2.47 [1.07, 5.73]
Lukaszyk 2018	0.9538	0.283	2.6%	2.60 [1.49, 4.52]
Almegbel 2018	1.0116	0.4317	1.4%	2.75 [1.18, 6.41]
Yoo 2016	1.2787	0.6079	0.7%	3.59 [1.09, 11.82]
Downton 1991	1.2802	0.5109	1.0%	3.60 [1.32, 9.79]
Turusheva 2020	1.8641	0.5434	0.9%	6.45 [2.22, 18.71]
Xu 2015	2.1422	0.3467	2.0%	8.52 [4.32, 16.81]
**Subtotal (95% CI)**			**65.5%**	**1.90 [1.62, 2.22]**
Heterogeneity: Tau² = 0.07; Chi² = 59.69, df = 23 (P < 0.0001); I² = 61%				
Test for overall effect: Z = 7.88 (P < 0.00001)				
**7.4.2 Hospital inpatient**				
Magnuszewski 2020	0.5568	0.3346	2.1%	1.75 [0.91, 3.36]
Kelly 2003	0.8671	0.1096	6.6%	2.38 [1.92, 2.95]
**Subtotal (95% CI)**			**8.6%**	**2.31 [1.88, 2.83]**
Heterogeneity: Tau² = 0.00; Chi² = 0.78, df = 1 (P = 0.38); I² = 0%				
Test for overall effect: Z = 8.04 (P < 0.00001)				
**7.4.3 Hospital outpatient**				
Akande-Sholabi 2021	0.3564	0.5657	0.9%	1.43 [0.47, 4.33]
Ha 2021	1.3324	0.4729	1.2%	3.79 [1.50, 9.58]
**Subtotal (95% CI)**			**2.0%**	**2.44 [0.94, 6.33]**
Heterogeneity: Tau² = 0.20; Chi² = 1.75, df = 1 (P = 0.19); I² = 43%				
Test for overall effect: Z = 1.84 (P = 0.07)				
**7.4.4 Residential care**				
Hung 2017	0.3428	0.2709	2.8%	1.41 [0.83, 2.40]
Rosendahl 2003	0.4108	0.5114	1.0%	1.51 [0.55, 4.11]
Kallin 2004	0.5203	0.3113	2.3%	1.68 [0.91, 3.10]
Paganini-Hill 2017	0.5365	0.1171	6.3%	1.71 [1.36, 2.15]
Paliwal 2017	0.6729	0.0406	8.5%	1.96 [1.81, 2.12]
Ku 2013	0.8372	0.2973	2.5%	2.31 [1.29, 4.14]
Shaw 2015	1.3863	0.858	0.4%	4.00 [0.74, 21.50]
**Subtotal (95% CI)**			**23.8%**	**1.92 [1.79, 2.07]**
Heterogeneity: Tau² = 0.00; Chi² = 4.06, df = 6 (P = 0.67); I² = 0%				
Test for overall effect: Z = 17.53 (P < 0.00001)				
**Total (95% CI)**			**100.0%**	**1.90 [1.70, 2.11]**
Heterogeneity: Tau² = 0.03; Chi² = 77.53, df = 34 (P < 0.0001); I² = 56%				
Test for overall effect: Z = 11.66 (P < 0.00001)				
Test for subgroup differences: Chi² = 3.13, df = 3 (P = 0.37), I² = 4.2%				

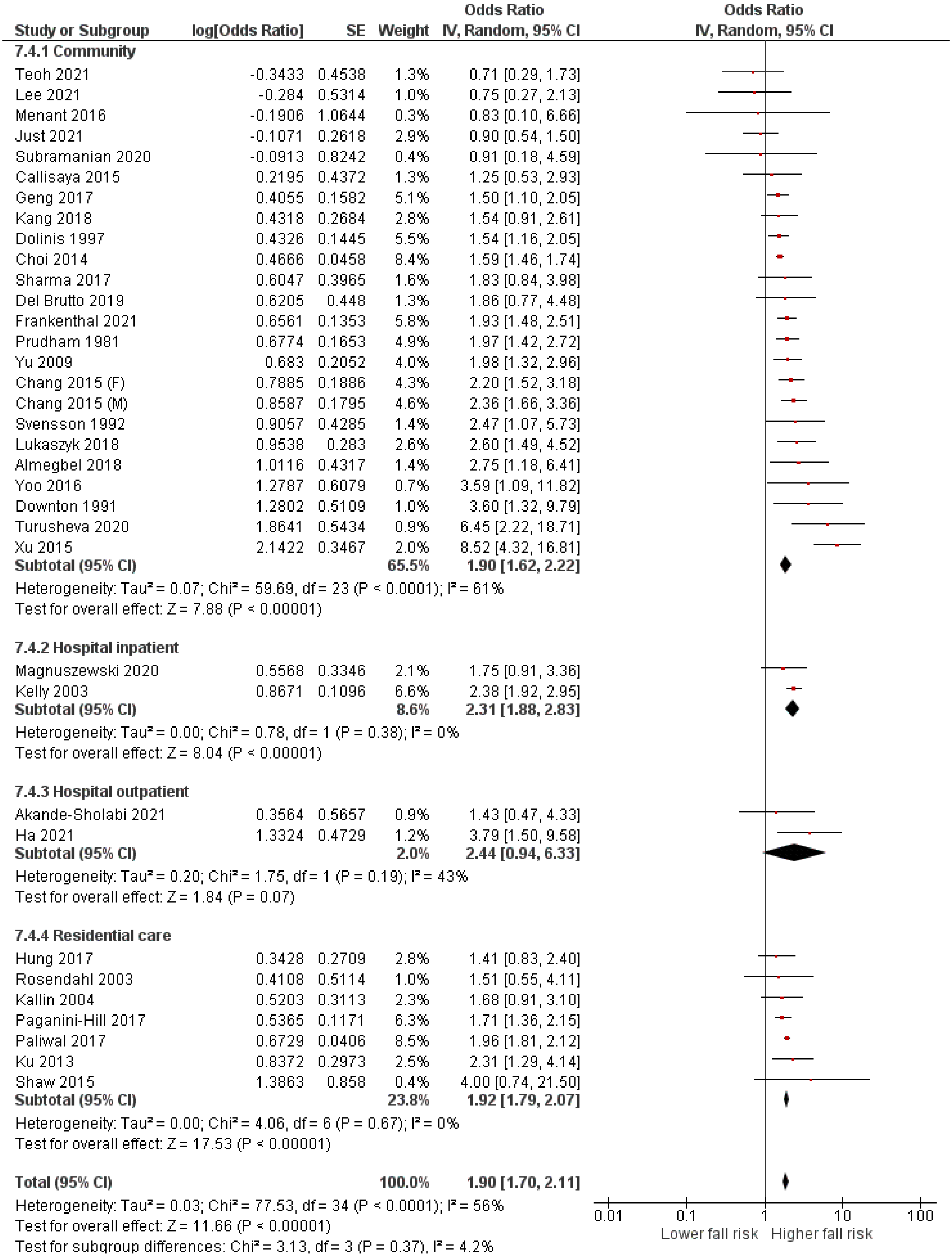

eFigure 17. Association between stroke and falls (unadjusted OR) stratified by setting

Study or Subgroup	log[Odds Ratio]	SE	Weight	Odds Ratio IV, Random, 95% CI
**2.5.1 Community**				
Chan 1997	-0.4111	0.4976	2.4%	0.66 [0.25, 1.76]
Almegbel 2018	0	0.1163	8.4%	1.00 [0.80, 1.26]
Lawlor 2003	0.038	0.1101	8.5%	1.04 [0.84, 1.29]
Wong 2014	0.1386	0.225	6.0%	1.15 [0.74, 1.79]
Luukinen 1996	0.2355	0.241	5.7%	1.27 [0.79, 2.03]
Miu 1997	0.3418	0.3314	4.2%	1.41 [0.74, 2.69]
Rutan 1992	0.5477	0.1894	6.8%	1.73 [1.19, 2.51]
Yu 2009	0.6084	0.2664	5.2%	1.84 [1.09, 3.10]
Susman 1989	0.7541	0.6047	1.8%	2.13 [0.65, 6.95]
Menant 2016	0.8109	0.384	3.5%	2.25 [1.06, 4.78]
Soysal 2016	0.9415	0.2486	5.6%	2.56 [1.57, 4.17]
Saedon 2016	1.0613	0.3146	4.4%	2.89 [1.56, 5.35]
Turusheva 2020	1.0633	0.8581	1.0%	2.90 [0.54, 15.57]
Heitterachi 2002	1.5198	0.8318	1.0%	4.57 [0.90, 23.34]
McDonald 2017	1.564	0.5734	1.9%	4.78 [1.55, 14.70]
**Subtotal (95% CI)**			**66.3%**	**1.59 [1.25, 2.01]**
Heterogeneity: Tau² = 0.11; Chi² = 40.32, df = 14 (P = 0.0002); I² = 65%				
Test for overall effect: Z = 3.85 (P = 0.0001)				
**2.5.2 Hospital outpatient**				
Mader 1987	-0.6466	0.5063	2.4%	0.52 [0.19, 1.41]
Aydin 2017	-0.0402	0.2954	4.7%	0.96 [0.54, 1.71]
Kocyigit 2021	0.458	0.1675	7.3%	1.58 [1.14, 2.20]
Van der Velde 2007 (a)	0.619	0.4191	3.1%	1.86 [0.82, 4.22]
**Subtotal (95% CI)**			**17.4%**	**1.21 [0.77, 1.90]**
Heterogeneity: Tau² = 0.11; Chi² = 6.29, df = 3 (P = 0.10); I² = 52%				
Test for overall effect: Z = 0.83 (P = 0.40)				
**2.5.3 Hospital inpatient**				
Magnuszewski 2020	0.2428	0.3029	4.6%	1.27 [0.70, 2.31]
Kocyigit 2020	0.3322	0.1986	6.6%	1.39 [0.94, 2.06]
**Subtotal (95% CI)**			**11.2%**	**1.36 [0.98, 1.88]**
Heterogeneity: Tau² = 0.00; Chi² = 0.06, df = 1 (P = 0.81); I² = 0%				
Test for overall effect: Z = 1.84 (P = 0.07)				
**2.5.4 Residential care**				
Hartog 2015	-0.478	0.3877	3.4%	0.62 [0.29, 1.33]
Shaw 2015	0.3773	0.6329	1.7%	1.46 [0.42, 5.04]
**Subtotal (95% CI)**			**5.1%**	**0.82 [0.37, 1.81]**
Heterogeneity: Tau² = 0.09; Chi² = 1.33, df = 1 (P = 0.25); I² = 25%				
Test for overall effect: Z = 0.49 (P = 0.62)				
**Total (95% CI)**			**100.0%**	**1.42 [1.20, 1.70]**
Heterogeneity: Tau² = 0.08; Chi² = 50.49, df = 22 (P = 0.0005); I² = 56%				
Test for overall effect: Z = 3.96 (P < 0.0001)				
Test for subgroup differences: Chi² = 3.27, df = 3 (P = 0.35), I² = 8.3%				

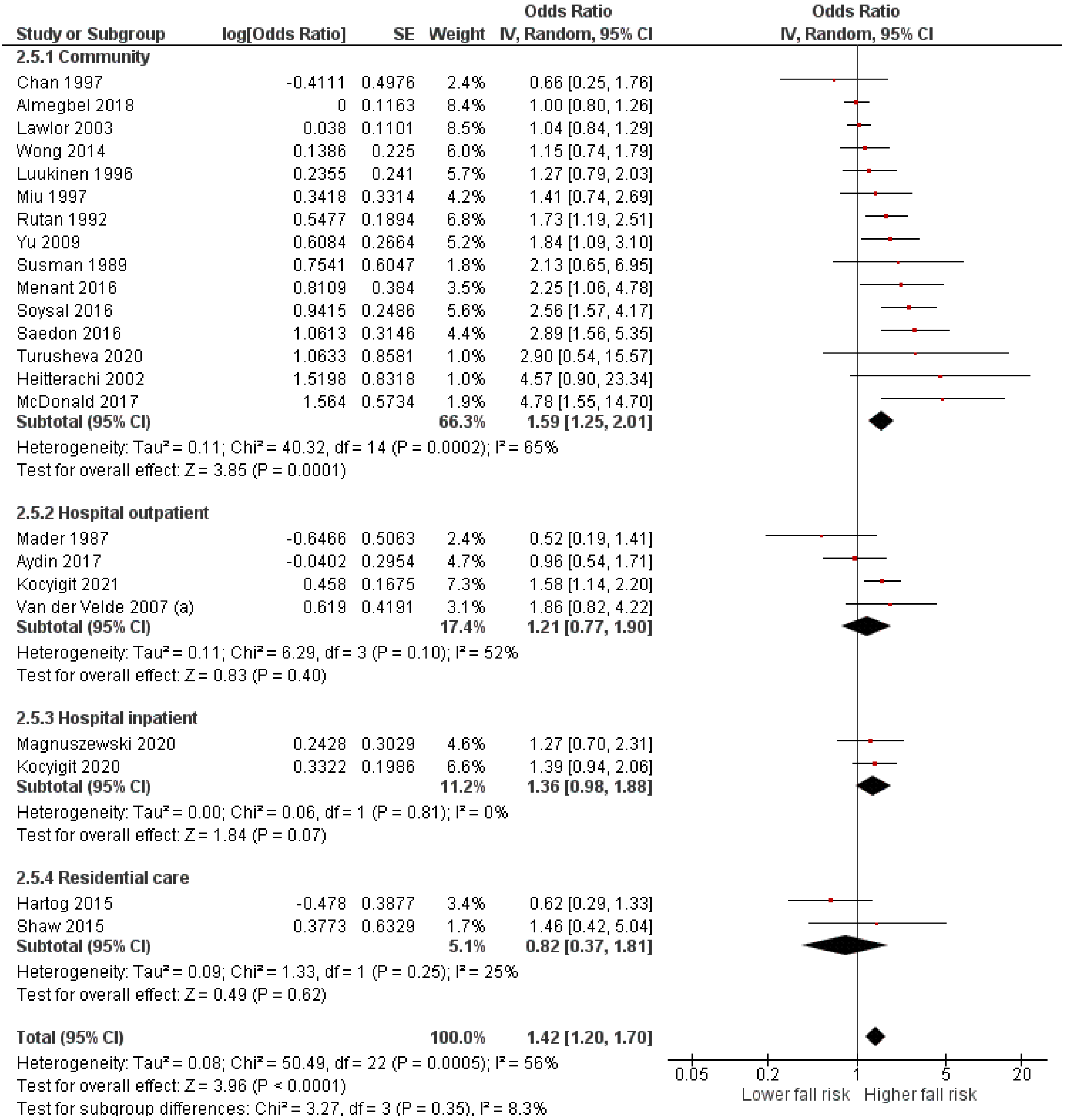

eFigure 18. Association between orthostatic hypotension and falls (unadjusted OR) stratified by setting

Study or Subgroup	log[Odds Ratio]	SE	Weight	Odds Ratio IV, Random, 95% CI
**4.4.1 Community**				
Menant 2016	-0.6539	0.7481	0.3%	0.52 [0.12, 2.25]
Teoh 2021	-0.3433	0.4538	0.8%	0.71 [0.29, 1.73]
Svensson 1992	-0.2984	0.3027	1.6%	0.74 [0.41, 1.34]
Jansen 2015 (a)	-0.0202	0.1365	5.5%	0.98 [0.75, 1.28]
Goh 2017	-0.0186	0.282	1.9%	0.98 [0.56, 1.71]
Turusheva 2020	0.0591	0.6721	0.4%	1.06 [0.28, 3.96]
Wong 2014	0.075	0.3208	1.5%	1.08 [0.57, 2.02]
Almegbel 2018	0.0814	21.6494	0.0%	1.08 [0.00, 2.906E18]
Chang 2015 (M)	0.1655	0.1	7.6%	1.18 [0.97, 1.44]
Choi 2014	0.2191	0.0428	11.9%	1.24 [1.14, 1.35]
Frankenthal 2021	0.2319	0.1157	6.6%	1.26 [1.01, 1.58]
Downton 1991	0.2479	0.2988	1.7%	1.28 [0.71, 2.30]
Kang 2018	0.2776	0.2429	2.4%	1.32 [0.82, 2.12]
Mitchell 2013	0.2852	0.0877	8.4%	1.33 [1.12, 1.58]
Sibley 2014	0.3519	0.0446	11.8%	1.42 [1.30, 1.55]
Chang 2015 (F)	0.5128	0.0973	7.8%	1.67 [1.38, 2.02]
Lawlor 2003	0.5988	0.109	7.0%	1.82 [1.47, 2.25]
Xu 2015	0.6487	0.2612	2.1%	1.91 [1.15, 3.19]
Gamage 2019	1.1314	0.3704	1.1%	3.10 [1.50, 6.41]
**Subtotal (95% CI)**			**80.3%**	**1.33 [1.20, 1.47]**
Heterogeneity: Tau² = 0.02; Chi² = 41.06, df = 17 (P = 0.0009); I² = 59%				
Test for overall effect: Z = 5.48 (P < 0.00001)				
**4.4.2 Residential care**				
Kallin 2004	0.3007	0.2906	1.8%	1.35 [0.76, 2.39]
Paliwal 2017	0.392	0.032	12.6%	1.48 [1.39, 1.58]
Ku 2013	0.3988	0.1984	3.3%	1.49 [1.01, 2.20]
Shaw 2015	2.3503	1.509	0.1%	10.49 [0.54, 201.92]
**Subtotal (95% CI)**			**17.7%**	**1.48 [1.39, 1.57]**
Heterogeneity: Tau² = 0.00; Chi² = 1.78, df = 3 (P = 0.62); I² = 0%				
Test for overall effect: Z = 12.48 (P < 0.00001)				
**4.4.3 Hospital inpatient**				
Magnuszewski 2020	0.1511	0.3523	1.3%	1.16 [0.58, 2.32]
Saedon 2016	0.5462	0.4712	0.7%	1.73 [0.69, 4.35]
**Subtotal (95% CI)**			**2.0%**	**1.34 [0.77, 2.33]**
Heterogeneity: Tau² = 0.00; Chi² = 0.45, df = 1 (P = 0.50); I² = 0%				
Test for overall effect: Z = 1.04 (P = 0.30)				
**Total (95% CI)**			**100.0%**	**1.36 [1.25, 1.47]**
Heterogeneity: Tau² = 0.01; Chi² = 49.83, df = 23 (P = 0.0010); I² = 54%				
Test for overall effect: Z = 7.36 (P < 0.00001)				
Test for subgroup differences: Chi² = 3.43, df = 2 (P = 0.18), I² = 41.7%				

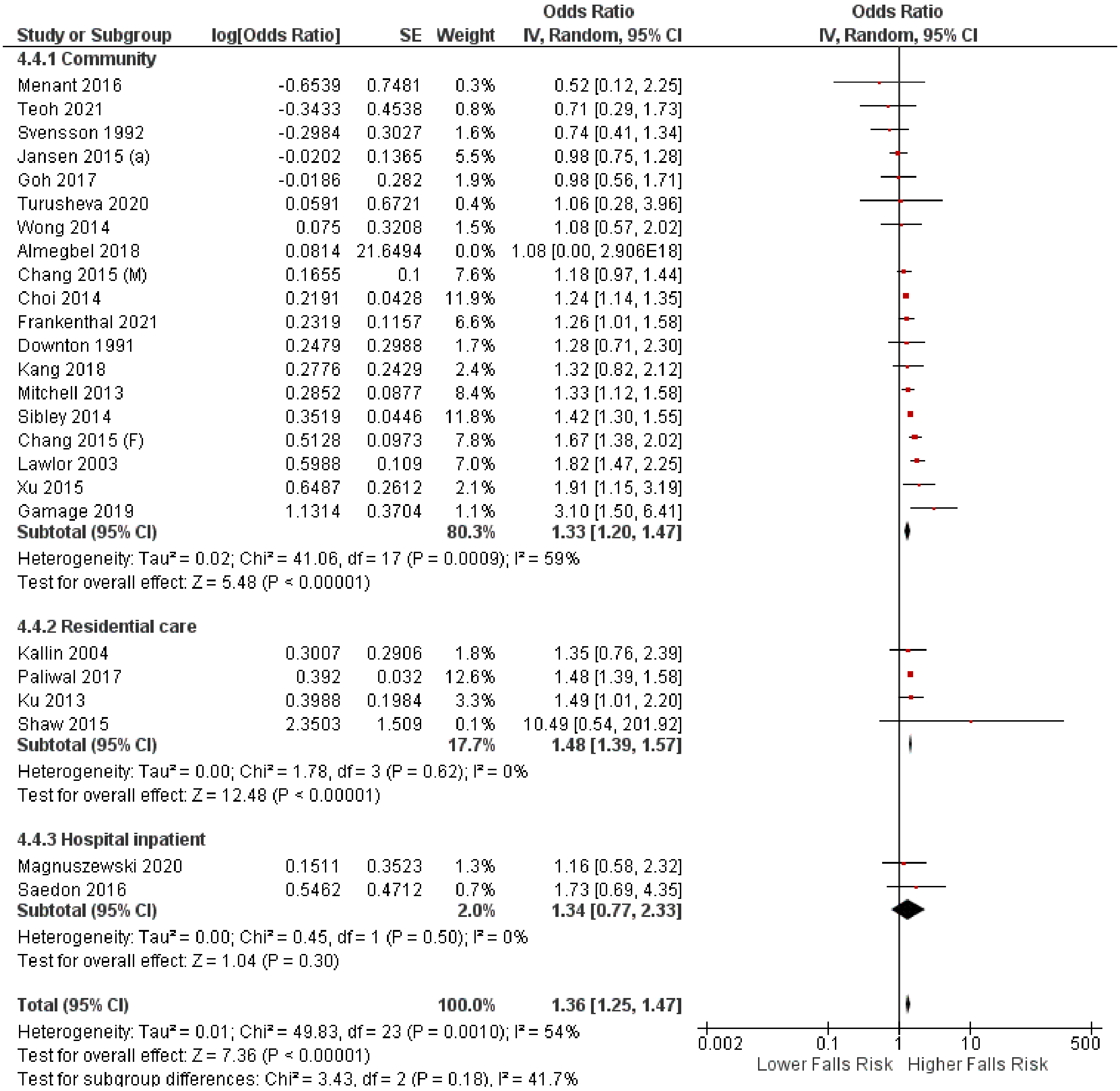

eFigure 19. Association between coronary artery disease and falls (unadjusted OR) stratified by setting

Study or Subgroup	log[Odds Ratio]	SE	Weight	Odds Ratio IV, Random, 95% CI
**5.4.1 Community**				
Just 2021	0.2506	0.1586	48.8%	1.28 [0.94, 1.75]
Jansen 2015 (a)	0.5247	0.238	21.7%	1.69 [1.06, 2.69]
**Subtotal (95% CI)**			**70.4%**	**1.40 [1.08, 1.81]**
Heterogeneity: Tau² = 0.00; Chi² = 0.92, df = 1 (P = 0.34); I² = 0%				
Test for overall effect: Z = 2.54 (P = 0.01)				
**5.4.2 Hospital inpatient**				
Saedon 2016	-0.1054	0.6826	2.6%	0.90 [0.24, 3.43]
Magnuszewski 2020	0.3275	0.2196	25.4%	1.39 [0.90, 2.13]
**Subtotal (95% CI)**			**28.1%**	**1.33 [0.88, 2.01]**
Heterogeneity: Tau² = 0.00; Chi² = 0.36, df = 1 (P = 0.55); I² = 0%				
Test for overall effect: Z = 1.37 (P = 0.17)				
**5.4.3 Residential care**				
Shaw 2015	0.7621	0.8962	1.5%	2.14 [0.37, 12.41]
**Subtotal (95% CI)**			**1.5%**	**2.14 [0.37, 12.41]**
Heterogeneity: Not applicable				
Test for overall effect: Z = 0.85 (P = 0.40)				
**Total (95% CI)**			**100.0%**	**1.39 [1.12, 1.72]**
Heterogeneity: Tau² = 0.00; Chi² = 1.56, df = 4 (P = 0.82); I² = 0%				
Test for overall effect: Z = 2.96 (P = 0.003)				
Test for subgroup differences: Chi² = 0.28, df = 2 (P = 0.87), I² = 0%				

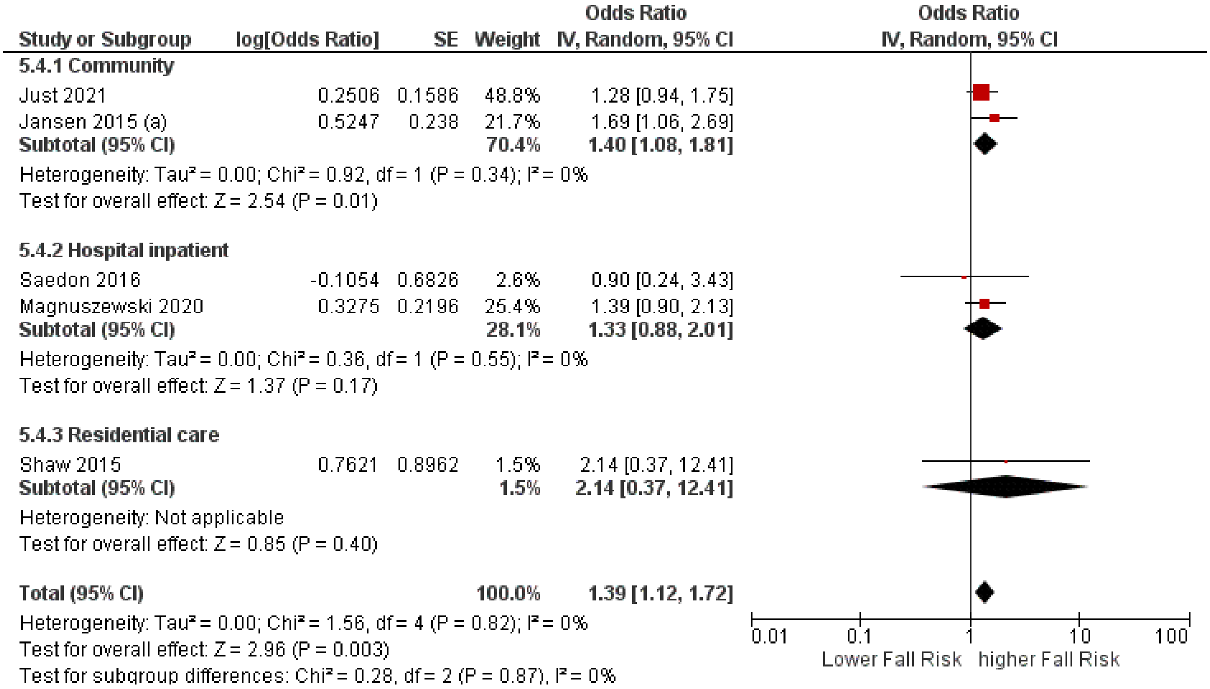

eFigure 20. Association between heart failure and falls (unadjusted OR) stratified by setting

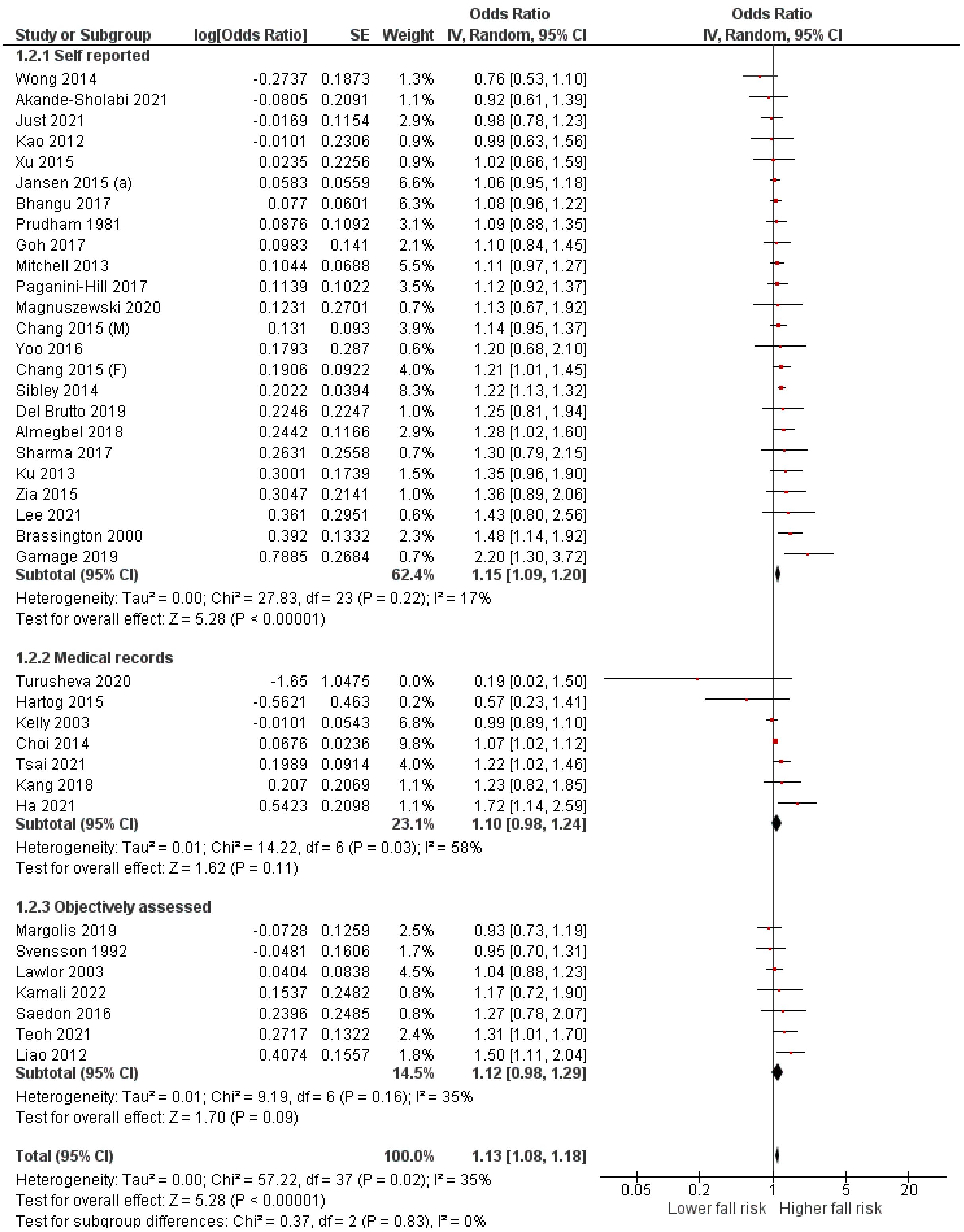

eFigure 21. Association between hypertension and falls (unadjusted OR) stratified by assessment method

Study or Subgroup	log[Odds Ratio]	SE	Weight	Odds Ratio IV, Random, 95% CI
**7.2.1 Self reported**				
Teoh 2021	-0.3433	0.4538	1.3%	0.71 [0.29, 1.73]
Lee 2021	-0.284	0.5314	1.0%	0.75 [0.27, 2.13]
Menant 2016	-0.1906	1.0644	0.3%	0.83 [0.10, 6.66]
Just 2021	-0.1071	0.2618	3.1%	0.90 [0.54, 1.50]
Subramanian 2020	-0.0913	0.8242	0.4%	0.91 [0.18, 4.59]
Callisaya 2015	0.2195	0.4372	1.4%	1.25 [0.53, 2.93]
Hung 2017	0.3428	0.2709	3.0%	1.41 [0.83, 2.40]
Akande-Sholabi 2021	0.3564	0.5657	0.9%	1.43 [0.47, 4.33]
Geng 2017	0.4055	0.1582	5.3%	1.50 [1.10, 2.05]
Kang 2018	0.4318	0.2684	3.0%	1.54 [0.91, 2.61]
Dolinis 1997	0.4326	0.1445	5.7%	1.54 [1.16, 2.05]
Choi 2014	0.4666	0.0458	8.6%	1.59 [1.46, 1.74]
Magnuszewski 2020	0.5568	0.3346	2.2%	1.75 [0.91, 3.36]
Del Brutto 2019	0.6205	0.448	1.4%	1.86 [0.77, 4.48]
Frankenthal 2021	0.6561	0.1353	6.0%	1.93 [1.48, 2.51]
Prudham 1981	0.6774	0.1653	5.1%	1.97 [1.42, 2.72]
Chang 2015 (F)	0.7885	0.1886	4.5%	2.20 [1.52, 3.18]
Ku 2013	0.8372	0.2973	2.6%	2.31 [1.29, 4.14]
Chang 2015 (M)	0.8587	0.1795	4.8%	2.36 [1.66, 3.36]
Svensson 1992	0.9057	0.4285	1.5%	2.47 [1.07, 5.73]
Lukaszyk 2018	0.9538	0.283	2.8%	2.60 [1.49, 4.52]
Almegbel 2018	1.0116	0.4317	1.4%	2.75 [1.18, 6.41]
Yoo 2016	1.2787	0.6079	0.8%	3.59 [1.09, 11.82]
Downton 1991	1.2802	0.5109	1.1%	3.60 [1.32, 9.79]
Ha 2021	1.3324	0.4729	1.2%	3.79 [1.50, 9.58]
Turusheva 2020	1.8641	0.5434	1.0%	6.45 [2.22, 18.71]
Xu 2015	2.1422	0.3467	2.1%	8.52 [4.32, 16.81]
**Subtotal (95% CI)**			**72.4%**	**1.90 [1.63, 2.21]**
Heterogeneity: Tau² = 0.07; Chi² = 63.62, df = 26 (P < 0.0001); I² = 59%				
Test for overall effect: Z = 8.19 (P < 0.00001)				
**7.2.2 Medical records**				
Rosendahl 2003	0.4108	0.5114	1.1%	1.51 [0.55, 4.11]
Kallin 2004	0.5203	0.3113	2.4%	1.68 [0.91, 3.10]
Paganini-Hill 2017	0.5365	0.1171	6.5%	1.71 [1.36, 2.15]
Sharma 2017	0.6047	0.3965	1.7%	1.83 [0.84, 3.98]
Paliwal 2017	0.6729	0.0406	8.7%	1.96 [1.81, 2.12]
Kelly 2003	0.8671	0.1096	6.8%	2.38 [1.92, 2.95]
Shaw 2015	1.3863	0.858	0.4%	4.00 [0.74, 21.50]
**Subtotal (95% CI)**			**27.6%**	**1.97 [1.84, 2.11]**
Heterogeneity: Tau² = 0.00; Chi² = 5.70, df = 6 (P = 0.46); I² = 0%				
Test for overall effect: Z = 19.00 (P < 0.00001)				
**Total (95% CI)**			**100.0%**	**1.90 [1.70, 2.12]**
Heterogeneity: Tau² = 0.04; Chi² = 77.39, df = 33 (P < 0.0001); I² = 57%				
Test for overall effect: Z = 11.28 (P < 0.00001)				
Test for subgroup differences: Chi² = 0.20, df = 1 (P = 0.66), I² = 0%				

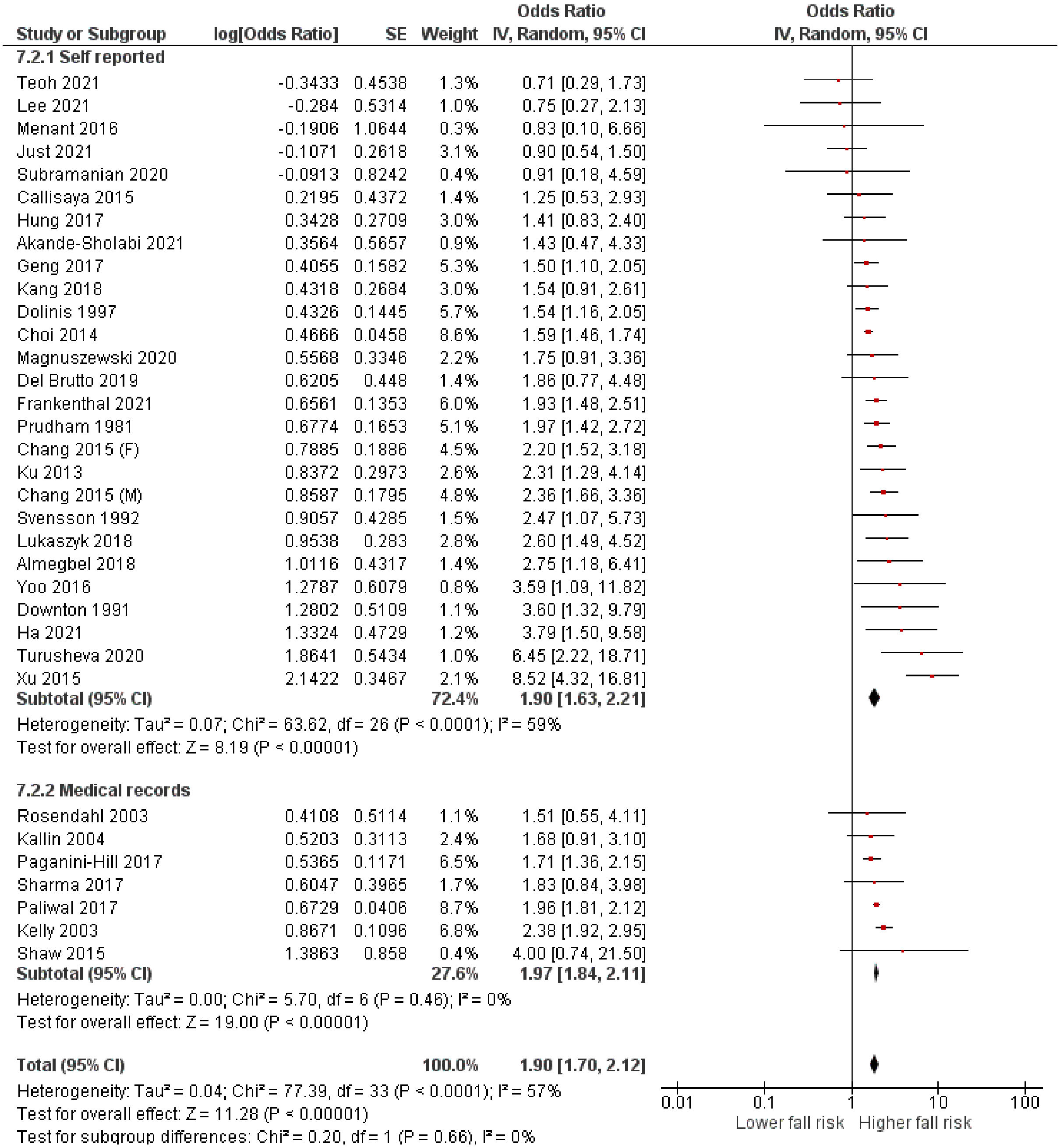

eFigure 22. Association between stroke and falls (unadjusted OR) stratified by assessment method

Study or Subgroup	log[Odds Ratio]	SE	Weight	Odds Ratio IV, Random, 95% CI
**4.2.1 Self reported**				
Menant 2016	-0.6539	0.7481	0.3%	0.52 [0.12, 2.25]
Teoh 2021	-0.3433	0.4538	0.8%	0.71 [0.29, 1.73]
Svensson 1992	-0.2984	0.3027	1.6%	0.74 [0.41, 1.34]
Jansen 2015 (a)	-0.0202	0.1365	5.7%	0.98 [0.75, 1.28]
Goh 2017	-0.0186	0.282	1.8%	0.98 [0.56, 1.71]
Turusheva 2020	0.0591	0.6721	0.4%	1.06 [0.28, 3.96]
Wong 2014	0.075	0.3208	1.5%	1.08 [0.57, 2.02]
Almegbel 2018	0.0814	21.6494	0.0%	1.08 [0.00, 2.906E18]
Magnuszewski 2020	0.1511	0.3523	1.2%	1.16 [0.58, 2.32]
Chang 2015 (M)	0.1655	0.1	8.1%	1.18 [0.97, 1.44]
Choi 2014	0.2191	0.0428	13.4%	1.24 [1.14, 1.35]
Frankenthal 2021	0.2319	0.1157	6.9%	1.26 [1.01, 1.58]
Downton 1991	0.2479	0.2988	1.7%	1.28 [0.71, 2.30]
Kang 2018	0.2776	0.2429	2.4%	1.32 [0.82, 2.12]
Mitchell 2013	0.2852	0.0877	9.1%	1.33 [1.12, 1.58]
Sibley 2014	0.3519	0.0446	13.3%	1.42 [1.30, 1.55]
Paliwal 2017	0.392	0.032	14.4%	1.48 [1.39, 1.58]
Chang 2015 (F)	0.5128	0.0973	8.3%	1.67 [1.38, 2.02]
Saedon 2016	0.5462	0.4712	0.7%	1.73 [0.69, 4.35]
Xu 2015	0.6487	0.2612	2.1%	1.91 [1.15, 3.19]
Gamage 2019	1.1314	0.3704	1.1%	3.10 [1.50, 6.41]
Shaw 2015	2.3503	1.509	0.1%	10.49 [0.54, 201.92]
**Subtotal (95% CI)**			**94.9%**	**1.32 [1.21, 1.44]**
Heterogeneity: Tau² = 0.01; Chi² = 43.31, df = 20 (P = 0.002); I² = 54%				
Test for overall effect: Z = 6.45 (P < 0.00001)				
**4.2.2 Medical records**				
Kallin 2004	0.3007	0.2906	1.7%	1.35 [0.76, 2.39]
Ku 2013	0.3988	0.1984	3.3%	1.49 [1.01, 2.20]
**Subtotal (95% CI)**			**5.1%**	**1.44 [1.05, 1.99]**
Heterogeneity: Tau² = 0.00; Chi² = 0.08, df = 1 (P = 0.78); I² = 0%				
Test for overall effect: Z = 2.24 (P = 0.02)				
**Total (95% CI)**			**100.0%**	**1.33 [1.23, 1.44]**
Heterogeneity: Tau² = 0.01; Chi² = 43.48, df = 22 (P = 0.004); I² = 49%				
Test for overall effect: Z = 6.98 (P < 0.00001)				
Test for subgroup differences: Chi² = 0.28, df = 1 (P = 0.60), I² = 0%				

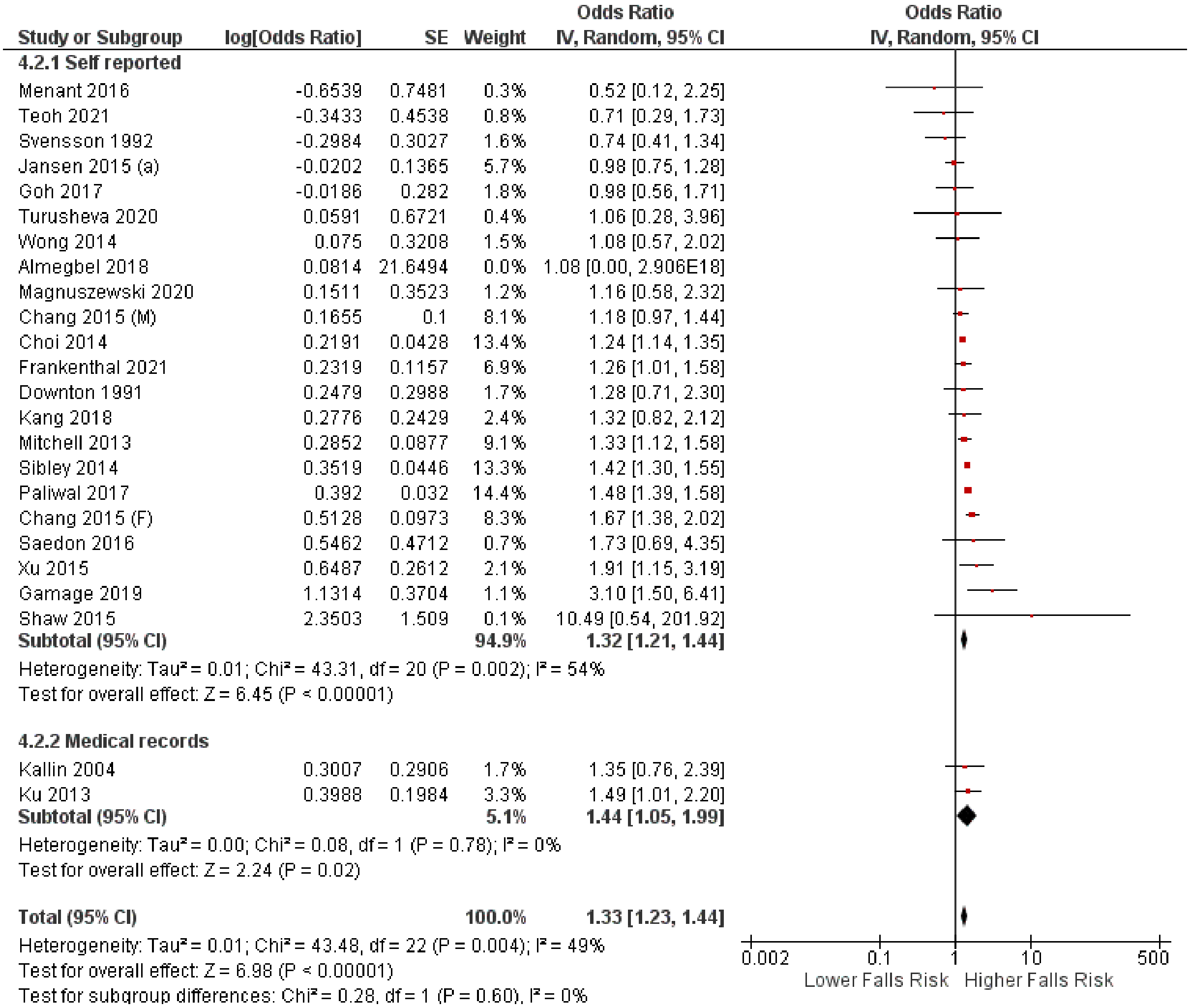

eFigure 23. Association between coronary artery disease and falls (unadjusted OR) stratified by assessment method

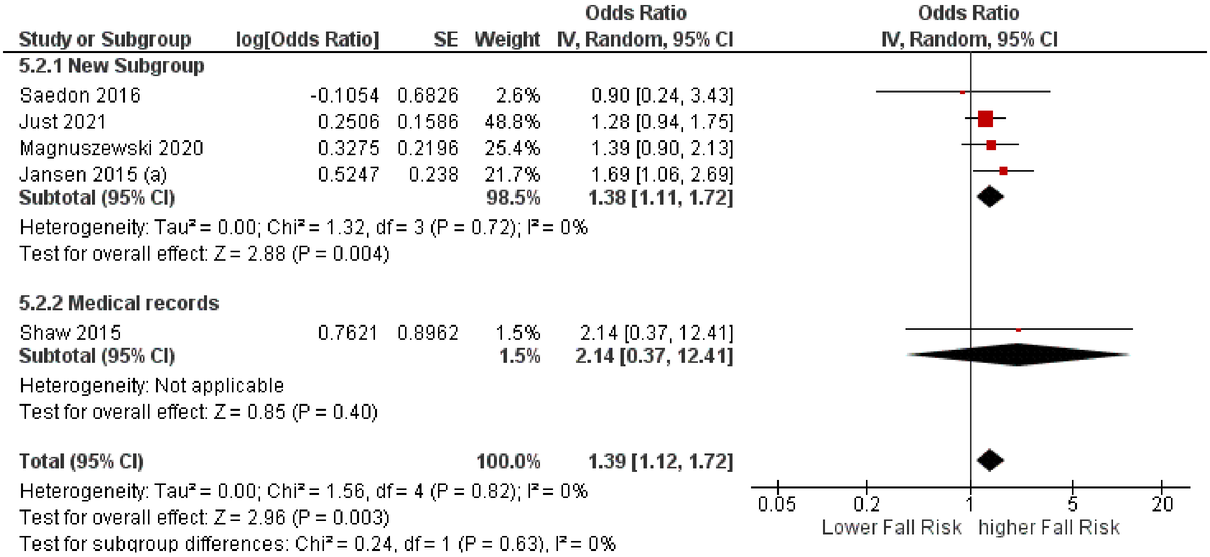

eFigure 24. Association between heart failure and falls (unadjusted OR) stratified by assessment method

Study or Subgroup	log[Odds Ratio]	SE	Weight	Odds Ratio IV, Random, 95% CI
**8.2.1 Self reported**				
Hung 2017	0.0766	0.4491	21.8%	1.08 [0.45, 2.60]
Magnuszewski 2020	0.6202	0.2622	41.7%	1.86 [1.11, 3.11]
Ha 2021	1.2413	0.4064	25.1%	3.46 [1.56, 7.67]
**Subtotal (95% CI)**			**88.5%**	**1.94 [1.10, 3.41]**
Heterogeneity: Tau² = 0.12; Chi² = 3.76, df = 2 (P = 0.15); I² = 47%				
Test for overall effect: Z = 2.29 (P = 0.02)				
**8.2.2 Medical records**				
Turusheva 2020	0.1117	0.6735	11.5%	1.12 [0.30, 4.19]
**Subtotal (95% CI)**			**11.5%**	**1.12 [0.30, 4.19]**
Heterogeneity: Not applicable				
Test for overall effect: Z = 0.17 (P = 0.87)				
**Total (95% CI)**			**100.0%**	**1.82 [1.12, 2.95]**
Heterogeneity: Tau² = 0.08; Chi² = 4.37, df = 3 (P = 0.22); I² = 31%				
Test for overall effect: Z = 2.43 (P = 0.02)				
Test for subgroup differences: Chi² = 0.56, df = 1 (P = 0.45), I² = 0%				

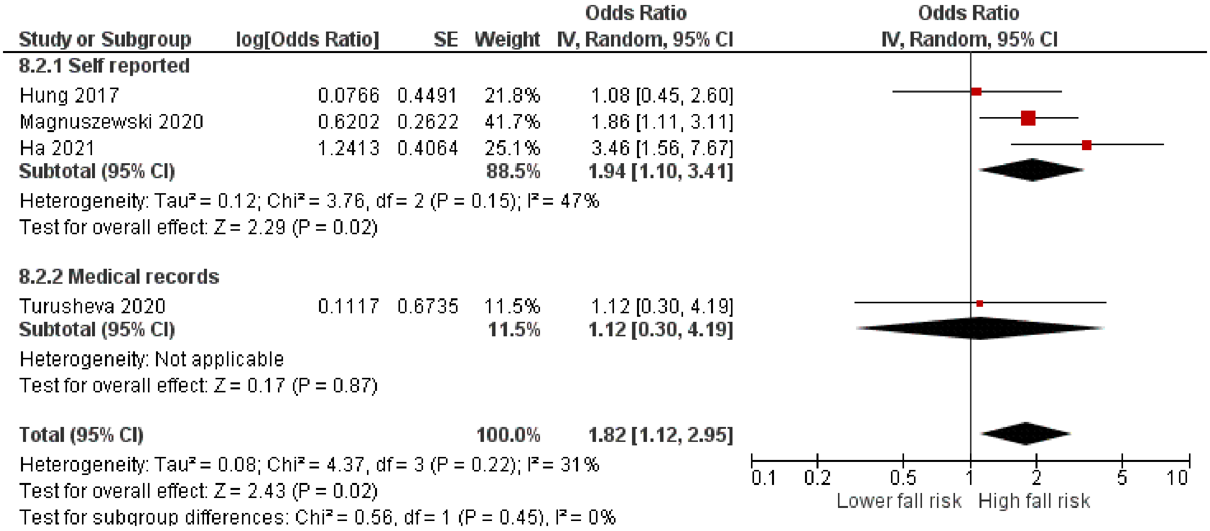

eFigure 25. Association between peripheral artery disease and falls (unadjusted OR) stratified by assessment method

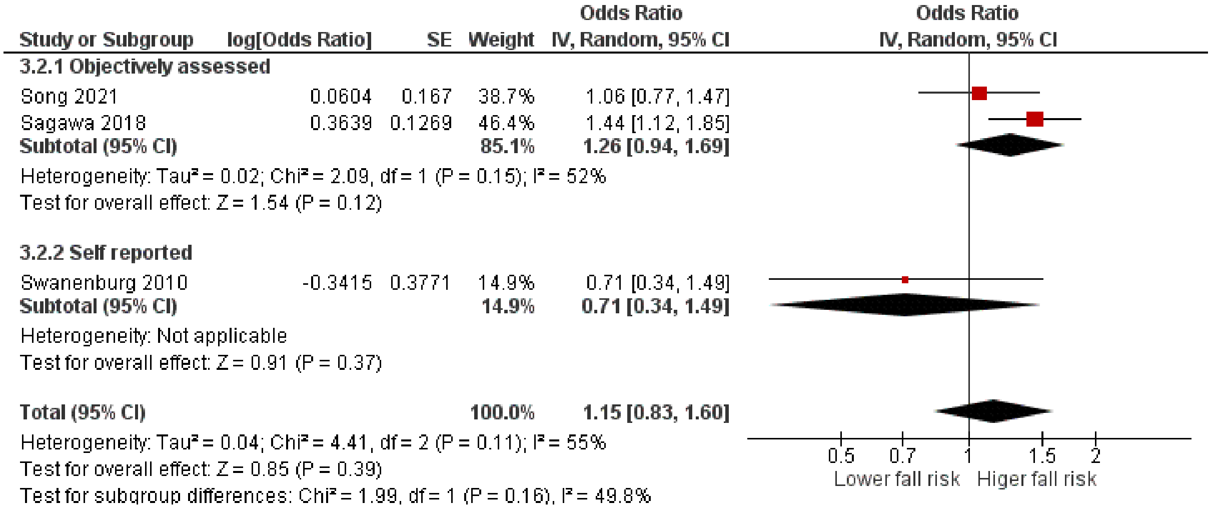

eFigure 26. Association between low blood pressure and falls (unadjusted OR) stratified by assessment method

Study or Subgroup	log[Odds Ratio]	SE	Weight	Odds Ratio IV, Random, 95% CI
**1.5.1 12 month follow up**				
Turusheva 2020	-1.65	1.0475	0.1%	0.19 [0.02, 1.50]
Hartog 2015	-0.5621	0.463	0.4%	0.57 [0.23, 1.41]
Wong 2014	-0.2737	0.1873	1.4%	0.76 [0.53, 1.10]
Akande-Sholabi 2021	-0.0805	0.2091	1.3%	0.92 [0.61, 1.39]
Margolis 2019	-0.0728	0.1259	2.0%	0.93 [0.73, 1.19]
Svensson 1992	-0.0481	0.1606	1.7%	0.95 [0.70, 1.31]
Just 2021	-0.0169	0.1154	2.2%	0.98 [0.78, 1.23]
Kao 2012	-0.0101	0.2306	1.1%	0.99 [0.63, 1.56]
Kelly 2003	-0.0101	0.0543	2.9%	0.99 [0.89, 1.10]
Xu 2015	0.0235	0.2256	1.2%	1.02 [0.66, 1.59]
Lawlor 2003	0.0404	0.0838	2.5%	1.04 [0.88, 1.23]
Jansen 2015 (a)	0.0583	0.0559	2.8%	1.06 [0.95, 1.18]
Choi 2014	0.0676	0.0236	3.1%	1.07 [1.02, 1.12]
Bhangu 2017	0.077	0.0601	2.8%	1.08 [0.96, 1.22]
Prudham 1981	0.0876	0.1092	2.2%	1.09 [0.88, 1.35]
Goh 2017	0.0983	0.141	1.9%	1.10 [0.84, 1.45]
Mitchell 2013	0.1044	0.0688	2.7%	1.11 [0.97, 1.27]
Paganini-Hill 2017	0.1139	0.1022	2.3%	1.12 [0.92, 1.37]
Magnuszewski 2020	0.1231	0.2701	0.9%	1.13 [0.67, 1.92]
Chang 2015 (M)	0.131	0.093	2.4%	1.14 [0.95, 1.37]
Kamali 2022	0.1537	0.2482	1.0%	1.17 [0.72, 1.90]
Yoo 2016	0.1793	0.287	0.8%	1.20 [0.68, 2.10]
Chang 2015 (F)	0.1906	0.0922	2.4%	1.21 [1.01, 1.45]
Tsai 2021	0.1989	0.0914	2.5%	1.22 [1.02, 1.46]
Sibley 2014	0.2022	0.0394	3.0%	1.22 [1.13, 1.32]
Kang 2018	0.207	0.2069	1.3%	1.23 [0.82, 1.85]
Del Brutto 2019	0.2246	0.2247	1.2%	1.25 [0.81, 1.94]
Saedon 2016	0.2396	0.2485	1.0%	1.27 [0.78, 2.07]
Almegbel 2018	0.2442	0.1166	2.2%	1.28 [1.02, 1.60]
Sharma 2017	0.2631	0.2558	1.0%	1.30 [0.79, 2.15]
Teoh 2021	0.2717	0.1322	2.0%	1.31 [1.01, 1.70]
Ku 2013	0.3001	0.1739	1.6%	1.35 [0.96, 1.90]
Zia 2015	0.3047	0.2141	1.2%	1.36 [0.89, 2.06]
Lee 2021	0.361	0.2951	0.8%	1.43 [0.80, 2.56]
Brassington 2000	0.392	0.1332	2.0%	1.48 [1.14, 1.92]
Yu 2009	0.3937	0.1344	2.0%	1.48 [1.14, 1.93]
Liao 2012	0.4074	0.1557	1.7%	1.50 [1.11, 2.04]
Ha 2021	0.5423	0.2098	1.3%	1.72 [1.14, 2.59]
Chan 1997	0.5786	0.2662	0.9%	1.78 [1.06, 3.01]
Gamage 2019	0.7885	0.2684	0.9%	2.20 [1.30, 3.72]
**Subtotal (95% CI)**			**68.6%**	**1.14 [1.09, 1.20]**
Heterogeneity: Tau² = 0.01; Chi² = 64.98, df = 39 (P = 0.006); I² = 40%				
Test for overall effect: Z = 5.57 (P < 0.00001)				
**1.5.2 6 month follow up**				
Lipsitz 1991	0.089	0.3657	0.6%	1.09 [0.53, 2.24]
Chen 2008	0.2327	0.3035	0.8%	1.26 [0.70, 2.29]
Jonsson 1990	0.619	0.5701	0.3%	1.86 [0.61, 5.68]
**Subtotal (95% CI)**			**1.6%**	**1.27 [0.83, 1.94]**
Heterogeneity: Tau² = 0.00; Chi² = 0.61, df = 2 (P = 0.74); I² = 0%				
Test for overall effect: Z = 1.10 (P = 0.27)				
**1.5.3 One month follow up**				
Juraschek 2019	0.1587	0.1129	2.2%	1.17 [0.94, 1.46]
Zhao 2019	0.2285	0.1835	1.5%	1.26 [0.88, 1.80]
Heckenbach 2014	0.2624	0.1339	2.0%	1.30 [1.00, 1.69]
**Subtotal (95% CI)**			**5.6%**	**1.23 [1.06, 1.43]**
Heterogeneity: Tau² = 0.00; Chi² = 0.37, df = 2 (P = 0.83); I² = 0%				
Test for overall effect: Z = 2.65 (P = 0.008)				
**1.5.4 During hospitalisaiton follow up**				
Chang 2011	-0.3711	0.2296	1.1%	0.69 [0.44, 1.08]
Rivera-Chavez 2021	-0.0316	0.1738	1.6%	0.97 [0.69, 1.36]
Chen 2010	0.3166	0.3406	0.6%	1.37 [0.70, 2.68]
**Subtotal (95% CI)**			**3.3%**	**0.92 [0.67, 1.28]**
Heterogeneity: Tau² = 0.03; Chi² = 3.04, df = 2 (P = 0.22); I² = 34%				
Test for overall effect: Z = 0.48 (P = 0.63)				

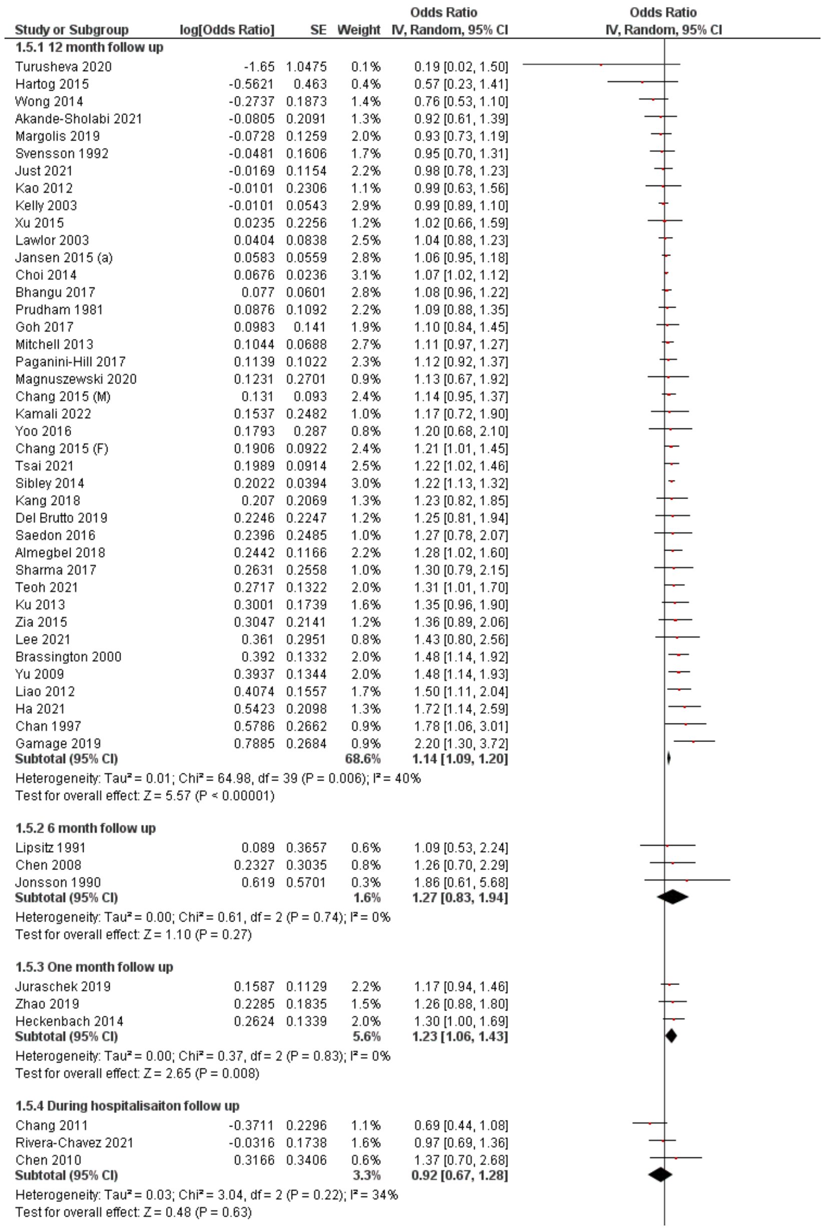

Study or Subgroup	log[Odds Ratio]	SE	Weight	Odds Ratio IV, Random, 95% CI
**1.5.5 36 month follow up**				
Hung 2013	-0.1586	0.2421	1.1%	0.85 [0.53, 1.37]
Banu 2018	0.0488	0.4101	0.5%	1.05 [0.47, 2.35]
**Subtotal (95% CI)**			**1.5%**	**0.90 [0.60, 1.35]**
Heterogeneity: Tau² = 0.00; Chi² = 0.19, df = 1 (P = 0.66); I² = 0%				
Test for overall effect: Z = 0.50 (P = 0.61)				
**1.5.6 24 month follow up**				
Valderrama-Hinds 2018	0.0296	0.0467	2.9%	1.03 [0.94, 1.13]
Lee 2020	0.3507	0.0258	3.1%	1.42 [1.35, 1.49]
**Subtotal (95% CI)**			**6.0%**	**1.21 [0.89, 1.66]**
Heterogeneity: Tau² = 0.05; Chi² = 36.22, df = 1 (P < 0.00001); I² = 97%				
Test for overall effect: Z = 1.20 (P = 0.23)				
**1.5.7 10 month follow up**				
Granek 1987	0.0716	0.2185	1.2%	1.07 [0.70, 1.65]
**Subtotal (95% CI)**			**1.2%**	**1.07 [0.70, 1.65]**
Heterogeneity: Not applicable				
Test for overall effect: Z = 0.33 (P = 0.74)				
**1.5.8 8 month follow up**				
Magnuszewski 2022	0.5051	0.7733	0.1%	1.66 [0.36, 7.54]
**Subtotal (95% CI)**			**0.1%**	**1.66 [0.36, 7.54]**
Heterogeneity: Not applicable				
Test for overall effect: Z = 0.65 (P = 0.51)				
**1.5.9 137 month follow up**				
Ham 2017 (B-PROOF)	-0.0382	0.0817	2.6%	0.96 [0.82, 1.13]
Ham 2017 (Rotterdam)	0.0695	0.0544	2.9%	1.07 [0.96, 1.19]
**Subtotal (95% CI)**			**5.4%**	**1.03 [0.94, 1.14]**
Heterogeneity: Tau² = 0.00; Chi² = 1.20, df = 1 (P = 0.27); I² = 17%				
Test for overall effect: Z = 0.64 (P = 0.52)				
**1.5.10 72 month follow up**				
Oren 2022	2.1782	0.1971	1.4%	8.83 [6.00, 12.99]
**Subtotal (95% CI)**			**1.4%**	**8.83 [6.00, 12.99]**
Heterogeneity: Not applicable				
Test for overall effect: Z = 11.05 (P < 0.00001)				
**1.5.11 60 month follow up**				
Rivan 2021	0.1366	0.2113	1.3%	1.15 [0.76, 1.73]
**Subtotal (95% CI)**			**1.3%**	**1.15 [0.76, 1.73]**
Heterogeneity: Not applicable				
Test for overall effect: Z = 0.65 (P = 0.52)				
**1.5.12 Not reported**				
Subramanian 2020	-0.0589	0.3722	0.6%	0.94 [0.45, 1.96]
**Subtotal (95% CI)**			**0.6%**	**0.94 [0.45, 1.96]**
Heterogeneity: Not applicable				
Test for overall effect: Z = 0.16 (P = 0.87)				
**1.5.13 168 month follow up**				
Thapa 2022	-0.3374	0.1759	1.5%	0.71 [0.51, 1.01]
**Subtotal (95% CI)**			**1.5%**	**0.71 [0.51, 1.01]**
Heterogeneity: Not applicable				
Test for overall effect: Z = 1.92 (P = 0.06)				
**1.5.14 18 month follow up**				
Ooi 2021	0.1072	0.1436	1.9%	1.11 [0.84, 1.47]
**Subtotal (95% CI)**			**1.9%**	**1.11 [0.84, 1.47]**
Heterogeneity: Not applicable				
Test for overall effect: Z = 0.75 (P = 0.46)				
**Total (95% CI)**			**100.0%**	**1.17 [1.10, 1.24]**
Heterogeneity: Tau² = 0.03; Chi² = 272.95, df = 61 (P < 0.00001); I² = 78%				
Test for overall effect: Z = 5.10 (P < 0.00001)				
Test for subgroup differences: Chi² = 123.48, df = 13 (P < 0.00001), I² = 89.5%				

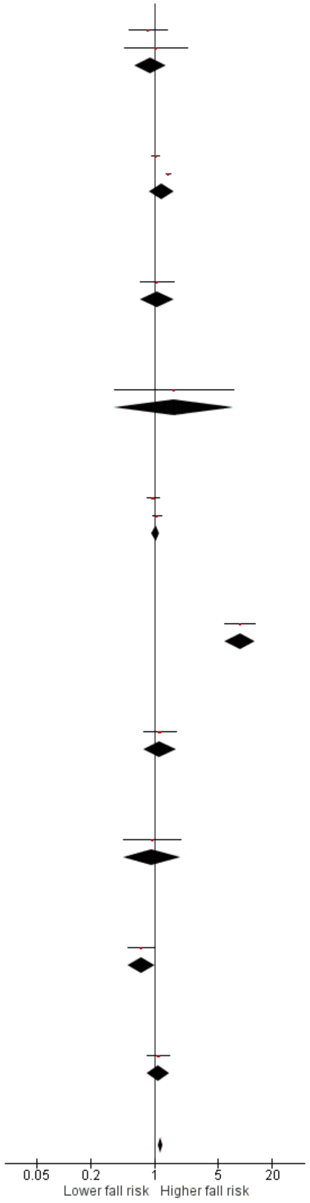

eFigure 27. Association between hypertension and falls (unadjusted OR) stratified by reporting interval

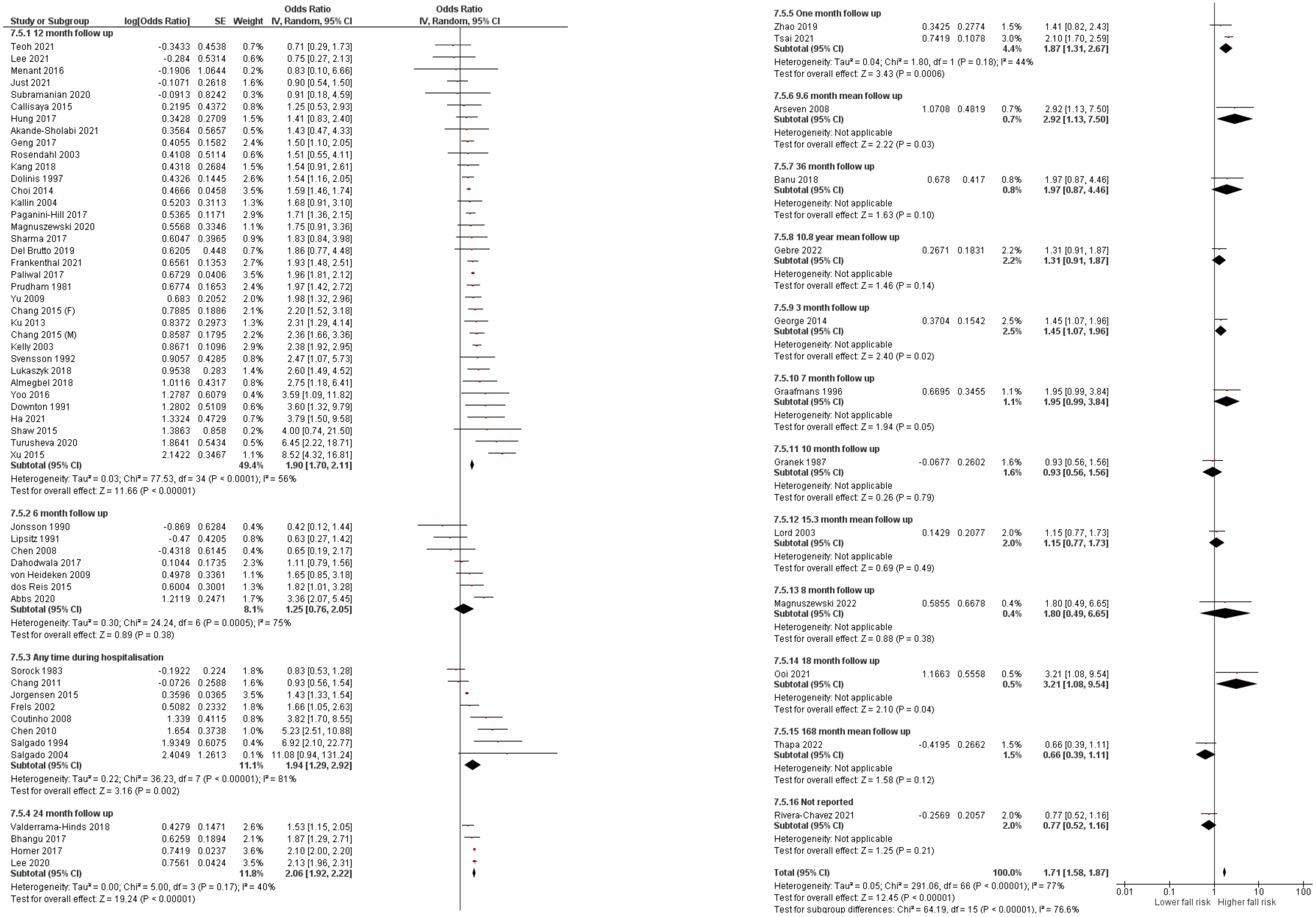

eFigure 28. Association between stroke and falls (unadjusted OR) stratified by reporting interval

Study or Subgroup	log[Odds Ratio]	SE	Weight	Odds Ratio IV, Random, 95% CI
**2.6.1 12 month follow up**				
Mader 1987	-0.6466	0.5063	1.3%	0.52 [0.19, 1.41]
Hartog 2015	-0.478	0.3877	1.8%	0.62 [0.29, 1.33]
Chan 1997	-0.4111	0.4976	1.3%	0.66 [0.25, 1.76]
Campbell 1989 (M)	-0.3429	0.3272	2.2%	0.71 [0.37, 1.35]
Aydin 2017	-0.0402	0.2954	2.4%	0.96 [0.54, 1.71]
Almegbel 2018	0	0.1163	3.9%	1.00 [0.80, 1.26]
Lawlor 2003	0.038	0.1101	4.0%	1.04 [0.84, 1.29]
Wong 2014	0.1386	0.225	3.0%	1.15 [0.74, 1.79]
Luukinen 1996	0.2355	0.241	2.8%	1.27 [0.79, 2.03]
Magnuszewski 2020	0.2428	0.3029	2.3%	1.27 [0.70, 2.31]
Kocyigit 2020	0.3322	0.1986	3.2%	1.39 [0.94, 2.06]
Miu 1997	0.3418	0.3314	2.1%	1.41 [0.74, 2.69]
Shaw 2015	0.3773	0.6329	0.9%	1.46 [0.42, 5.04]
Campbell 1989 (F)	0.3857	0.2055	3.1%	1.47 [0.98, 2.20]
Kocyigit 2021	0.458	0.1675	3.5%	1.58 [1.14, 2.20]
Rutan 1992	0.5477	0.1894	3.3%	1.73 [1.19, 2.51]
Yu 2009	0.6084	0.2664	2.6%	1.84 [1.09, 3.10]
Van der Velde 2007 (a)	0.619	0.4191	1.6%	1.86 [0.82, 4.22]
Susman 1989	0.7541	0.6047	1.0%	2.13 [0.65, 6.95]
Menant 2016	0.8109	0.384	1.8%	2.25 [1.06, 4.78]
Soysal 2016	0.9415	0.2486	2.8%	2.56 [1.57, 4.17]
Saedon 2016	1.0613	0.3146	2.2%	2.89 [1.56, 5.35]
Turusheva 2020	1.0633	0.8581	0.5%	2.90 [0.54, 15.57]
Heitterachi 2002	1.5198	0.8318	0.6%	4.57 [0.90, 23.34]
McDonald 2017	1.564	0.5734	1.0%	4.78 [1.55, 14.70]
**Subtotal (95% CI)**			**55.1%**	**1.39 [1.18, 1.64]**
Heterogeneity: Tau² = 0.08; Chi² = 54.26, df = 24 (P = 0.0004); I² = 56%				
Test for overall effect: Z = 3.91 (P < 0.0001)				
**2.6.2 6 month follow up**				
Coutaz 2012	0.2536	0.2216	3.0%	1.29 [0.83, 1.99]
Roca 2022	0.372	0.2284	2.9%	1.45 [0.93, 2.27]
Jodaitis 2015	1.0006	0.255	2.7%	2.72 [1.65, 4.48]
Romero-Ortuno 2011	1.043	0.3056	2.3%	2.84 [1.56, 5.17]
Lipsitz 1991	1.204	1.1328	0.3%	3.33 [0.36, 30.70]
**Subtotal (95% CI)**			**11.3%**	**1.91 [1.32, 2.78]**
Heterogeneity: Tau² = 0.09; Chi² = 8.29, df = 4 (P = 0.08); I² = 52%				
Test for overall effect: Z = 3.40 (P = 0.0007)				
**2.6.3 Any time in the past**				
Press 2016	-0.0592	0.2162	3.0%	0.94 [0.62, 1.44]
Bumin 2002	1.2891	0.8116	0.6%	3.63 [0.74, 17.81]
**Subtotal (95% CI)**			**3.6%**	**1.47 [0.42, 5.12]**
Heterogeneity: Tau² = 0.56; Chi² = 2.58, df = 1 (P = 0.11); I² = 61%				
Test for overall effect: Z = 0.61 (P = 0.54)				
**2.6.4 One week follow up**				
Davies 2001	0.6707	0.5507	1.1%	1.96 [0.66, 5.75]
**Subtotal (95% CI)**			**1.1%**	**1.96 [0.66, 5.75]**
Heterogeneity: Not applicable				
Test for overall effect: Z = 1.22 (P = 0.22)				
**2.6.5 24 month follow up**				
Finucane 2017	0.3095	0.1562	3.6%	1.36 [1.00, 1.85]
**Subtotal (95% CI)**			**3.6%**	**1.36 [1.00, 1.85]**
Heterogeneity: Not applicable				
Test for overall effect: Z = 1.98 (P = 0.05)				
**2.6.6 Follow up during hospitalisation**				
Frels 2002	1.2811	0.8088	0.6%	3.60 [0.74, 17.57]
**Subtotal (95% CI)**			**0.6%**	**3.60 [0.74, 17.57]**
Heterogeneity: Not applicable				
Test for overall effect: Z = 1.58 (P = 0.11)				
**2.6.7 6-12 month follow up**				
Gangavati 2011	-0.4618	0.2278	2.9%	0.63 [0.40, 0.98]
**Subtotal (95% CI)**			**2.9%**	**0.63 [0.40, 0.98]**
Heterogeneity: Not applicable				
Test for overall effect: Z = 2.03 (P = 0.04)				
**2.6.8 137 month median follow up**				
Ham 2017 (Rotterdam)	0.0334	0.0853	4.1%	1.03 [0.87, 1.22]
**Subtotal (95% CI)**			**4.1%**	**1.03 [0.87, 1.22]**
Heterogeneity: Not applicable				
Test for overall effect: Z = 0.39 (P = 0.70)				
**2.6.9 54 week median follow up**				
Juraschek 2022	0.1712	0.2256	3.0%	1.19 [0.76, 1.85]
Juraschek 2022	0.6575	0.5204	1.2%	1.93 [0.70, 5.35]
**Subtotal (95% CI)**			**4.2%**	**1.28 [0.85, 1.92]**
Heterogeneity: Tau² = 0.00; Chi² = 0.74, df = 1 (P = 0.39); I² = 0%				
Test for overall effect: Z = 1.20 (P = 0.23)				
**2.6.10 8 month follow up**				
Magnuszewski 2022	1.6413	0.5605	1.1%	5.16 [1.72, 15.48]
**Subtotal (95% CI)**			**1.1%**	**5.16 [1.72, 15.48]**
Heterogeneity: Not applicable				
Test for overall effect: Z = 2.93 (P = 0.003)				
**2.6.11 18 month follow up**				
Ooi 2000	1.0008	0.1765	3.4%	2.72 [1.92, 3.84]
**Subtotal (95% CI)**			**3.4%**	**2.72 [1.92, 3.84]**
Heterogeneity: Not applicable				
Test for overall effect: Z = 5.67 (P < 0.00001)				
**2.6.12 3 month follow up**				
Tinetti 1986	2.8306	1.5325	0.2%	16.96 [0.84, 341.81]
**Subtotal (95% CI)**			**0.2%**	**16.96 [0.84, 341.81]**
Heterogeneity: Not applicable				
Test for overall effect: Z = 1.85 (P = 0.06)				
**2.6.13 Not reported**				
Subramanian 2020	-0.3193	0.5897	1.0%	0.73 [0.23, 2.31]
Rivera-Chavez 2021	0.1166	0.2172	3.0%	1.12 [0.73, 1.72]
Zhu 2016	0.5559	0.3953	1.7%	1.74 [0.80, 3.78]
**Subtotal (95% CI)**			**5.8%**	**1.18 [0.83, 1.69]**
Heterogeneity: Tau² = 0.00; Chi² = 1.70, df = 2 (P = 0.43); I² = 0%				
Test for overall effect: Z = 0.93 (P = 0.35)				
**2.6.14 9.6 month average follow up**				
Arseven 2008	-0.4726	0.7923	0.6%	0.62 [0.13, 2.95]
**Subtotal (95% CI)**			**0.6%**	**0.62 [0.13, 2.95]**
Heterogeneity: Not applicable				
Test for overall effect: Z = 0.60 (P = 0.55)				
**2.6.15 7 month follow up**				
Graafmans 1996	0.2897	0.2904	2.4%	1.34 [0.76, 2.36]
**Subtotal (95% CI)**			**2.4%**	**1.34 [0.76, 2.36]**
Heterogeneity: Not applicable				
Test for overall effect: Z = 1.00 (P = 0.32)				
**Total (95% CI)**			**100.0%**	**1.43 [1.26, 1.63]**
Heterogeneity: Tau² = 0.10; Chi² = 123.40, df = 46 (P < 0.00001); I² = 63%				
Test for overall effect: Z = 5.38 (P < 0.00001)				
Test for subgroup differences: Chi² = 51.75, df = 14 (P < 0.00001), I² = 72.9%				

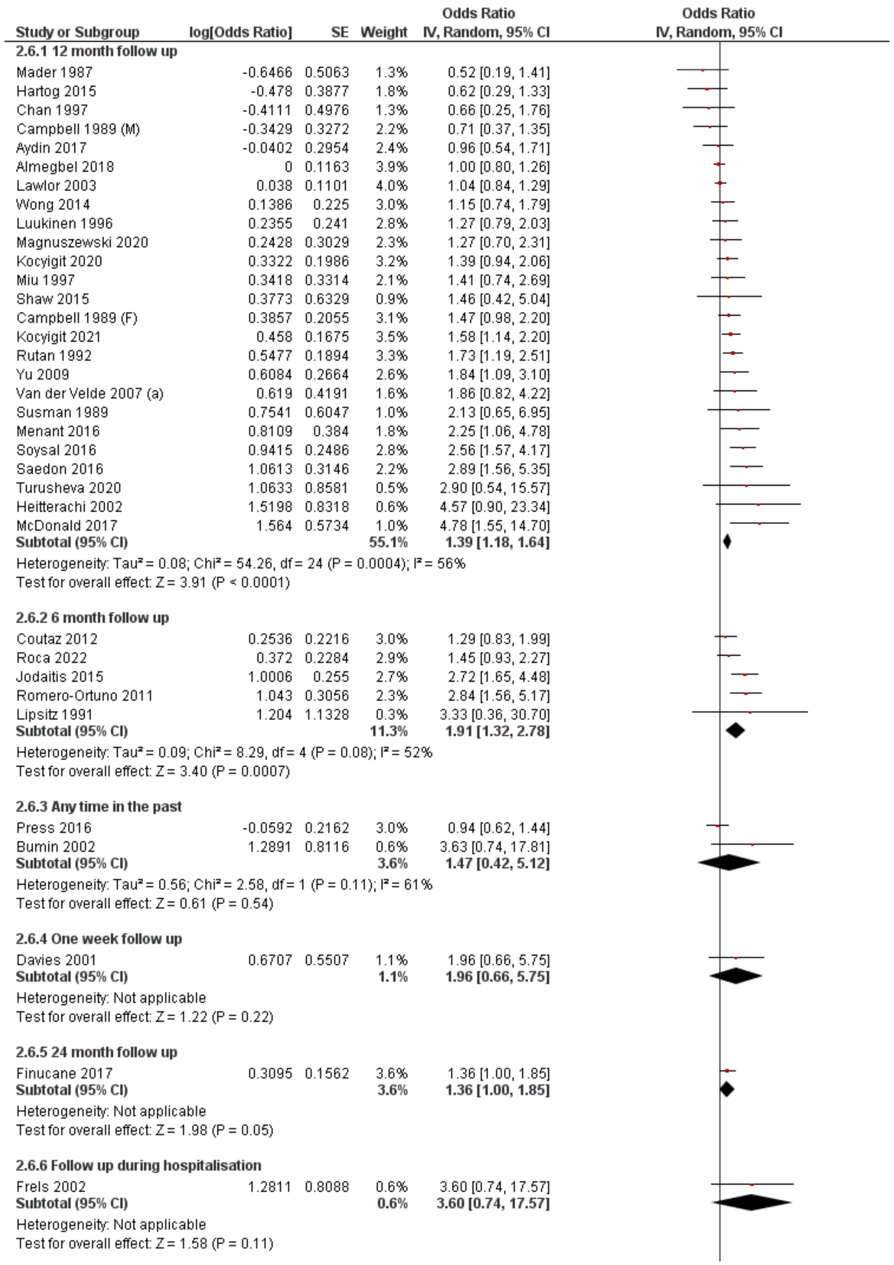

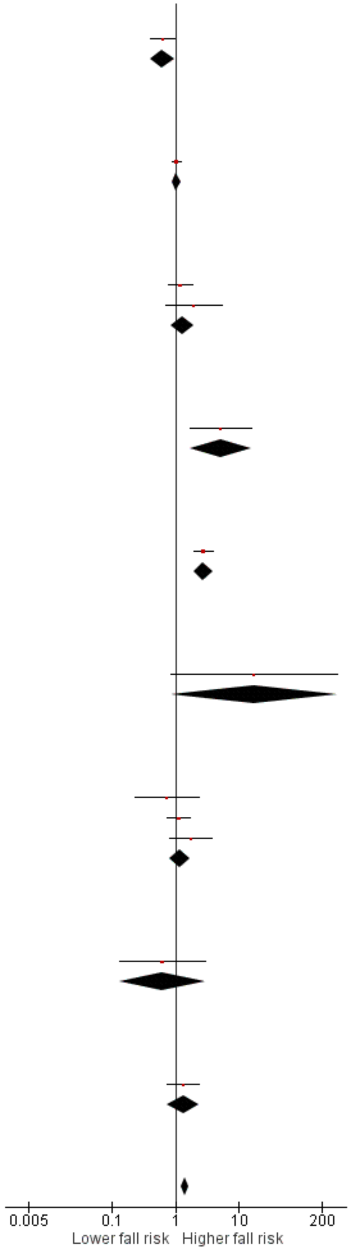

eFigure 29. Association between orthostatic hypotension and falls (unadjusted OR) stratified by reporting interval

Study or Subgroup	log[Odds Ratio]	SE	Weight	Odds Ratio IV, Random, 95% CI
**4.5.1 12 month follow up**				
Menant 2016	-0.6539	0.7481	0.3%	0.52 [0.12, 2.25]
Teoh 2021	-0.3433	0.4538	0.6%	0.71 [0.29, 1.73]
Svensson 1992	-0.2984	0.3027	1.3%	0.74 [0.41, 1.34]
Jansen 2015 (a)	-0.0202	0.1365	3.4%	0.98 [0.75, 1.28]
Goh 2017	-0.0186	0.282	1.4%	0.98 [0.56, 1.71]
Turusheva 2020	0.0591	0.6721	0.3%	1.06 [0.28, 3.96]
Wong 2014	0.075	0.3208	1.2%	1.08 [0.57, 2.02]
Almegbel 2018	0.0814	21.6494	0.0%	1.08 [0.00, 2.906E18]
Magnuszewski 2020	0.1511	0.3523	1.0%	1.16 [0.58, 2.32]
Chang 2015 (M)	0.1655	0.1	4.2%	1.18 [0.97, 1.44]
Choi 2014	0.2191	0.0428	5.4%	1.24 [1.14, 1.35]
Frankenthal 2021	0.2319	0.1157	3.8%	1.26 [1.01, 1.58]
Downton 1991	0.2479	0.2988	1.3%	1.28 [0.71, 2.30]
Kang 2018	0.2776	0.2429	1.8%	1.32 [0.82, 2.12]
Mitchell 2013	0.2852	0.0877	4.5%	1.33 [1.12, 1.58]
Kallin 2004	0.3007	0.2906	1.4%	1.35 [0.76, 2.39]
Sibley 2014	0.3519	0.0446	5.4%	1.42 [1.30, 1.55]
Paliwal 2017	0.392	0.032	5.6%	1.48 [1.39, 1.58]
Ku 2013	0.3988	0.1984	2.3%	1.49 [1.01, 2.20]
Chang 2015 (F)	0.5128	0.0973	4.2%	1.67 [1.38, 2.02]
Saedon 2016	0.5462	0.4712	0.6%	1.73 [0.69, 4.35]
Lawlor 2003	0.5988	0.109	4.0%	1.82 [1.47, 2.25]
Xu 2015	0.6487	0.2612	1.6%	1.91 [1.15, 3.19]
Gamage 2019	1.1314	0.3704	0.9%	3.10 [1.50, 6.41]
Shaw 2015	2.3503	1.509	0.1%	10.49 [0.54, 201.92]
**Subtotal (95% CI)**			**56.4%**	**1.36 [1.25, 1.47]**
Heterogeneity: Tau² = 0.01; Chi² = 49.83, df = 23 (P = 0.0010); I² = 54%				
Test for overall effect: Z = 7.36 (P < 0.00001)				
**4.5.2 6 month follow up**				
von Heideken 2009	0.1243	0.3147	1.2%	1.13 [0.61, 2.10]
Lipsitz 1991	0.3582	0.3683	0.9%	1.43 [0.70, 2.94]
Abbs 2020	0.7655	0.2567	1.6%	2.15 [1.30, 3.56]
Jonsson 1990	1.4917	0.6466	0.3%	4.44 [1.25, 15.78]
**Subtotal (95% CI)**			**4.1%**	**1.74 [1.11, 2.73]**
Heterogeneity: Tau² = 0.08; Chi² = 4.93, df = 3 (P = 0.18); I² = 39%				
Test for overall effect: Z = 2.42 (P = 0.02)				
**4.5.3 36 follow up**				
Banu 2018	0.0165	0.3799	0.9%	1.02 [0.48, 2.14]
Hung 2017	0.4813	0.1928	2.4%	1.62 [1.11, 2.36]
**Subtotal (95% CI)**			**3.3%**	**1.44 [0.97, 2.14]**
Heterogeneity: Tau² = 0.02; Chi² = 1.19, df = 1 (P = 0.28); I² = 16%				
Test for overall effect: Z = 1.80 (P = 0.07)				
**4.5.4 24 follow up**				
Bhangu 2017	-0.0202	0.1365	3.4%	0.98 [0.75, 1.28]
Valderrama-Hinds 2018	0.2297	0.1285	3.5%	1.26 [0.98, 1.62]
**Subtotal (95% CI)**			**6.9%**	**1.12 [0.87, 1.42]**
Heterogeneity: Tau² = 0.01; Chi² = 1.78, df = 1 (P = 0.18); I² = 44%				
Test for overall effect: Z = 0.87 (P = 0.38)				
**4.5.5 3 month follow up**				
George 2014	0.5295	0.2204	2.0%	1.70 [1.10, 2.62]
**Subtotal (95% CI)**			**2.0%**	**1.70 [1.10, 2.62]**
Heterogeneity: Not applicable				
Test for overall effect: Z = 2.40 (P = 0.02)				
**4.5.6 10 month follow up**				
Granek 1987	0.2177	0.2089	2.2%	1.24 [0.83, 1.87]
**Subtotal (95% CI)**			**2.2%**	**1.24 [0.83, 1.87]**
Heterogeneity: Not applicable				
Test for overall effect: Z = 1.04 (P = 0.30)				
**4.5.7 Any time during hospitalisation**				
Jorgensen 2015	-0.3425	0.0945	4.3%	0.71 [0.59, 0.85]
Chen 2010	0.2835	0.3095	1.2%	1.33 [0.72, 2.44]
**Subtotal (95% CI)**			**5.5%**	**0.91 [0.50, 1.65]**
Heterogeneity: Tau² = 0.14; Chi² = 3.74, df = 1 (P = 0.05); I² = 73%				
Test for overall effect: Z = 0.32 (P = 0.75)				
**4.5.8 One week follow up**				
Herndon 1997	0.1823	0.1777	2.6%	1.20 [0.85, 1.70]
**Subtotal (95% CI)**			**2.6%**	**1.20 [0.85, 1.70]**
Heterogeneity: Not applicable				
Test for overall effect: Z = 1.03 (P = 0.30)				
**4.5.9 9.6 month mean follow up**				
Arseven 2008	-0.0324	0.3896	0.8%	0.97 [0.45, 2.08]
**Subtotal (95% CI)**			**0.8%**	**0.97 [0.45, 2.08]**
Heterogeneity: Not applicable				
Test for overall effect: Z = 0.08 (P = 0.93)				
**4.5.10 8 month follow up**				
Magnuszewski 2022	0.7967	0.6	0.4%	2.22 [0.68, 7.19]
**Subtotal (95% CI)**			**0.4%**	**2.22 [0.68, 7.19]**
Heterogeneity: Not applicable				
Test for overall effect: Z = 1.33 (P = 0.18)				
**4.5.11 30 month mean follow up**				
Rafiq 2014	0.1484	0.0225	5.7%	1.16 [1.11, 1.21]
**Subtotal (95% CI)**			**5.7%**	**1.16 [1.11, 1.21]**
Heterogeneity: Not applicable				
Test for overall effect: Z = 6.60 (P < 0.00001)				
**4.5.12 Not reported**				
Rivera-Chavez 2021	0.0488	0.2217	2.0%	1.05 [0.68, 1.62]
Subramanian 2020	0.5691	0.4799	0.6%	1.77 [0.69, 4.53]
**Subtotal (95% CI)**			**2.6%**	**1.15 [0.78, 1.71]**
Heterogeneity: Tau² = 0.00; Chi² = 0.97, df = 1 (P = 0.33); I² = 0%				
Test for overall effect: Z = 0.70 (P = 0.49)				
**4.5.13 72 month follow up**				
Stenhagen 2013	0.5306	0.1448	3.2%	1.70 [1.28, 2.26]
**Subtotal (95% CI)**			**3.2%**	**1.70 [1.28, 2.26]**
Heterogeneity: Not applicable				
Test for overall effect: Z = 3.66 (P = 0.0002)				
**4.5.14 168 month mean follow up**				
Thapa 2022	-0.4598	0.6009	0.4%	0.63 [0.19, 2.05]
**Subtotal (95% CI)**			**0.4%**	**0.63 [0.19, 2.05]**
Heterogeneity: Not applicable				
Test for overall effect: Z = 0.77 (P = 0.44)				
**4.5.15 One month follow up**				
Tsai 2021	0.4187	0.108	4.0%	1.52 [1.23, 1.88]
**Subtotal (95% CI)**			**4.0%**	**1.52 [1.23, 1.88]**
Heterogeneity: Not applicable				
Test for overall effect: Z = 3.88 (P = 0.0001)				
**Total (95% CI)**			**100.0%**	**1.30 [1.21, 1.41]**
Heterogeneity: Tau² = 0.03; Chi² = 151.80, df = 44 (P < 0.00001); I² = 71%				
Test for overall effect: Z = 6.86 (P < 0.00001)				
Test for subgroup differences: Chi² = 30.39, df = 14 (P = 0.007), I² = 53.9%				

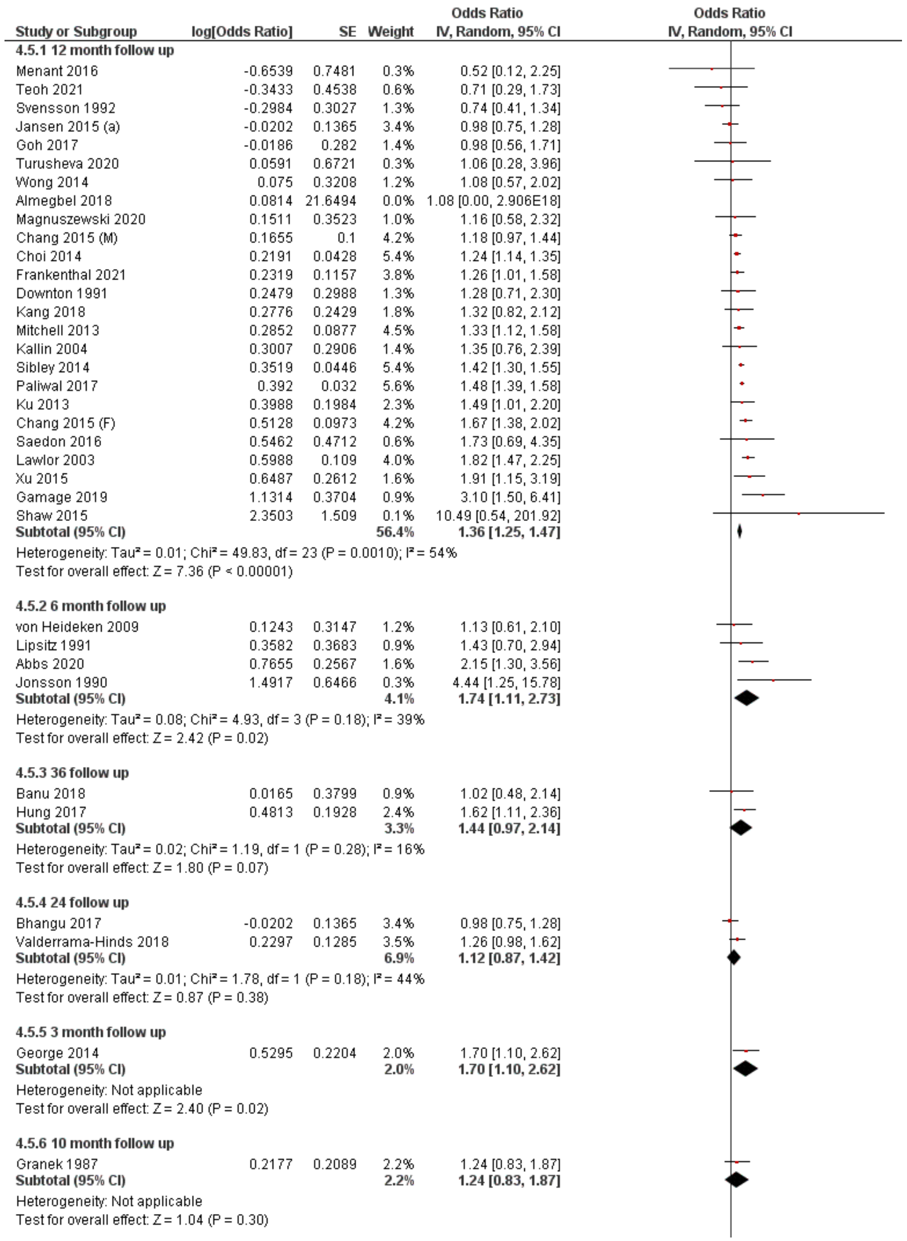

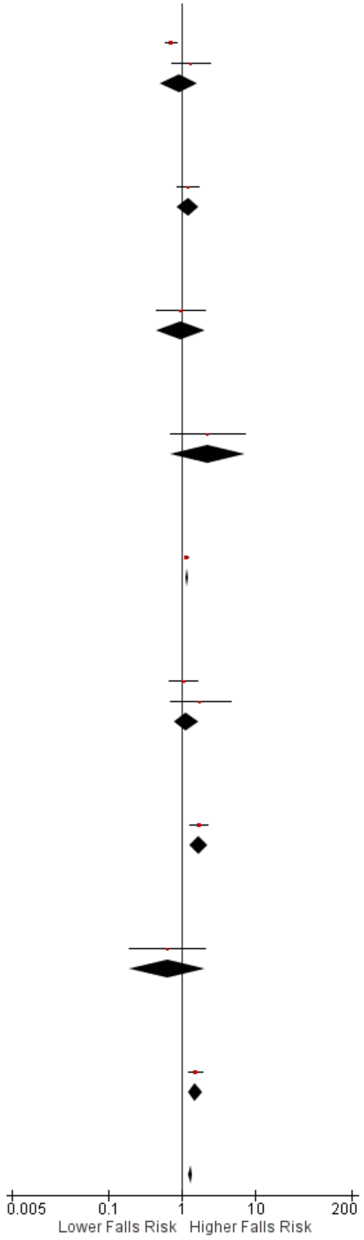

eFigure 30. Association between coronary artery disease and falls (unadjusted OR) stratified by reporting interval

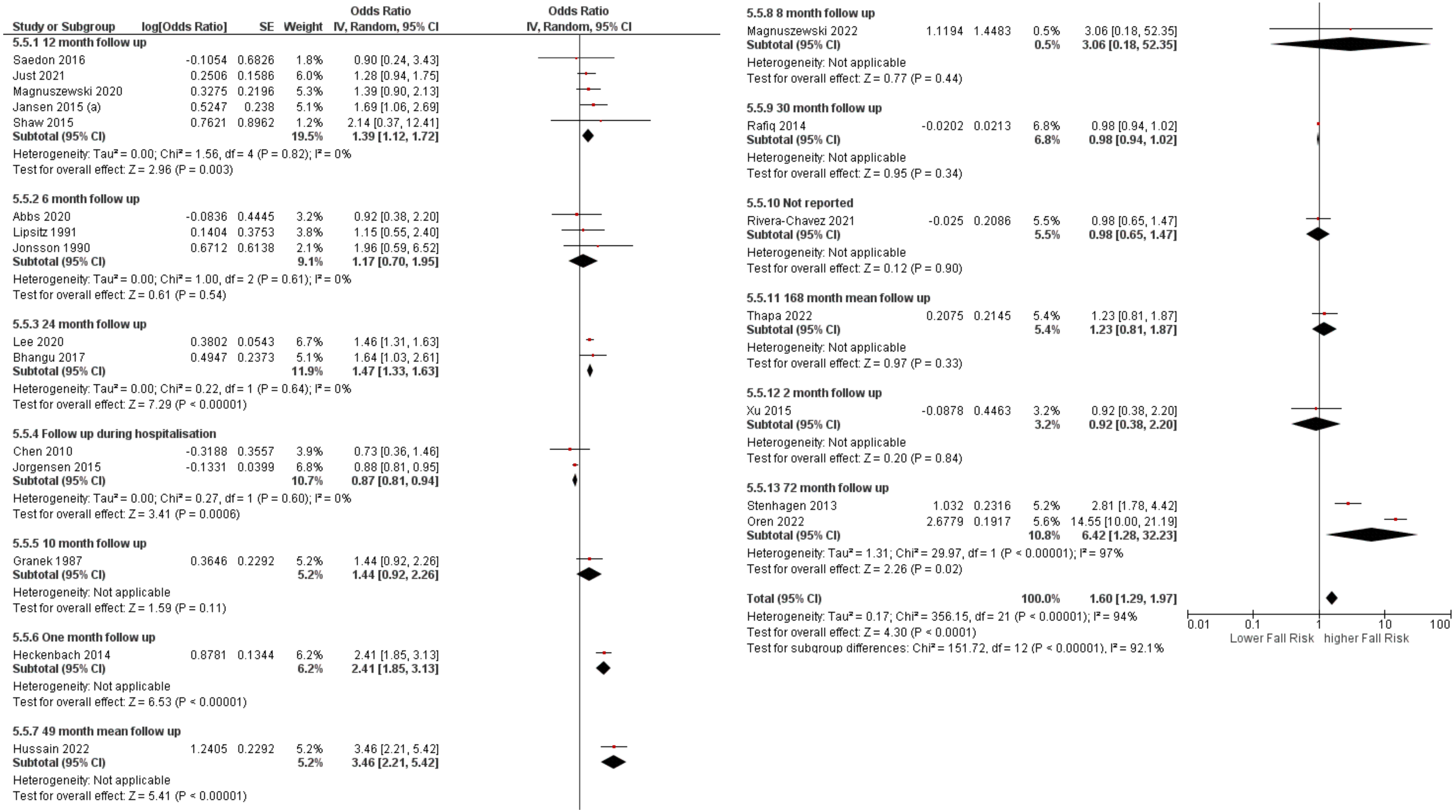

Study or Subgroup	log[Odds Ratio]	SE	Weight	Odds Ratio IV, Random, 95% CI
**5.5.1 12 month follow up**				
Saedon 2016	-0.1054	0.6826	1.8%	0.90 [0.24, 3.43]
Just 2021	0.2506	0.1586	6.0%	1.28 [0.94, 1.75]
Magnuszewski 2020	0.3275	0.2196	5.3%	1.39 [0.90, 2.13]
Jansen 2015 (a)	0.5247	0.238	5.1%	1.69 [1.06, 2.69]
Shaw 2015	0.7621	0.8962	1.2%	2.14 [0.37, 12.41]
**Subtotal (95% CI)**			**19.5%**	**1.39 [1.12, 1.72]**
Heterogeneity: Tau² = 0.00; Chi² = 1.56, df = 4 (P = 0.82); I² = 0%				
Test for overall effect: Z = 2.96 (P = 0.003)				
**5.5.2 6 month follow up**				
Abbs 2020	-0.0836	0.4445	3.2%	0.92 [0.38, 2.20]
Lipsitz 1991	0.1404	0.3753	3.8%	1.15 [0.55, 2.40]
Jonsson 1990	0.6712	0.6138	2.1%	1.96 [0.59, 6.52]
**Subtotal (95% CI)**			**9.1%**	**1.17 [0.70, 1.95]**
Heterogeneity: Tau² = 0.00; Chi² = 1.00, df = 2 (P = 0.61); I² = 0%				
Test for overall effect: Z = 0.61 (P = 0.54)				
**5.5.3 24 month follow up**				
Lee 2020	0.3802	0.0543	6.7%	1.46 [1.31, 1.63]
Bhangu 2017	0.4947	0.2373	5.1%	1.64 [1.03, 2.61]
**Subtotal (95% CI)**			**11.9%**	**1.47 [1.33, 1.63]**
Heterogeneity: Tau² = 0.00; Chi² = 0.22, df = 1 (P = 0.64); I² = 0%				
Test for overall effect: Z = 7.29 (P < 0.00001)				
**5.5.4 Follow up during hospitalisation**				
Chen 2010	-0.3188	0.3557	3.9%	0.73 [0.36, 1.46]
Jorgensen 2015	-0.1331	0.0399	6.8%	0.88 [0.81, 0.95]
**Subtotal (95% CI)**			**10.7%**	**0.87 [0.81, 0.94]**
Heterogeneity: Tau² = 0.00; Chi² = 0.27, df = 1 (P = 0.60); I² = 0%				
Test for overall effect: Z = 3.41 (P = 0.0006)				
**5.5.5 10 month follow up**				
Granek 1987	0.3646	0.2292	5.2%	1.44 [0.92, 2.26]
**Subtotal (95% CI)**			**5.2%**	**1.44 [0.92, 2.26]**
Heterogeneity: Not applicable				
Test for overall effect: Z = 1.59 (P = 0.11)				
**5.5.6 One month follow up**				
Heckenbach 2014	0.8781	0.1344	6.2%	2.41 [1.85, 3.13]
**Subtotal (95% CI)**			**6.2%**	**2.41 [1.85, 3.13]**
Heterogeneity: Not applicable				
Test for overall effect: Z = 6.53 (P < 0.00001)				
**5.5.7 49 month mean follow up**				
Hussain 2022	1.2405	0.2292	5.2%	3.46 [2.21, 5.42]
**Subtotal (95% CI)**			**5.2%**	**3.46 [2.21, 5.42]**
Heterogeneity: Not applicable				
Test for overall effect: Z = 5.41 (P < 0.00001)				
**5.5.8 8 month follow up**				
Magnuszewski 2022	1.1194	1.4483	0.5%	3.06 [0.18, 52.35]
**Subtotal (95% CI)**			**0.5%**	**3.06 [0.18, 52.35]**
Heterogeneity: Not applicable				
Test for overall effect: Z = 0.77 (P = 0.44)				
**5.5.9 30 month follow up**				
Rafiq 2014	-0.0202	0.0213	6.8%	0.98 [0.94, 1.02]
**Subtotal (95% CI)**			**6.8%**	**0.98 [0.94, 1.02]**
Heterogeneity: Not applicable				
Test for overall effect: Z = 0.95 (P = 0.34)				
**5.5.10 Not reported**				
Rivera-Chavez 2021	-0.025	0.2086	5.5%	0.98 [0.65, 1.47]
**Subtotal (95% CI)**			**5.5%**	**0.98 [0.65, 1.47]**
Heterogeneity: Not applicable				
Test for overall effect: Z = 0.12 (P = 0.90)				
**5.5.11 168 month mean follow up**				
Thapa 2022	0.2075	0.2145	5.4%	1.23 [0.81, 1.87]
**Subtotal (95% CI)**			**5.4%**	**1.23 [0.81, 1.87]**
Heterogeneity: Not applicable				
Test for overall effect: Z = 0.97 (P = 0.33)				
**5.5.12 2 month follow up**				
Xu 2015	-0.0878	0.4463	3.2%	0.92 [0.38, 2.20]
**Subtotal (95% CI)**			**3.2%**	**0.92 [0.38, 2.20]**
Heterogeneity: Not applicable				
Test for overall effect: Z = 0.20 (P = 0.84)				
**5.5.13 72 month follow up**				
Stenhagen 2013	1.032	0.2316	5.2%	2.81 [1.78, 4.42]
Oren 2022	2.6779	0.1917	5.6%	14.55 [10.00, 21.19]
**Subtotal (95% CI)**			**10.8%**	**6.42 [1.28, 32.23]**
Heterogeneity: Tau² = 1.31; Chi² = 29.97, df = 1 (P < 0.00001); I² = 97%				
Test for overall effect: Z = 2.26 (P = 0.02)				
**Total (95% CI)**			**100.0%**	**1.60 [1.29, 1.97]**
Heterogeneity: Tau² = 0.17; Chi² = 356.15, df = 21 (P < 0.00001); I² = 94%				
Test for overall effect: Z = 4.30 (P < 0.0001)				
Test for subgroup differences: Chi² = 151.72, df = 12 (P < 0.00001), I² = 92.1%				

eFigure 31. Association between heart failure and falls (unadjusted OR) stratified by reporting interval

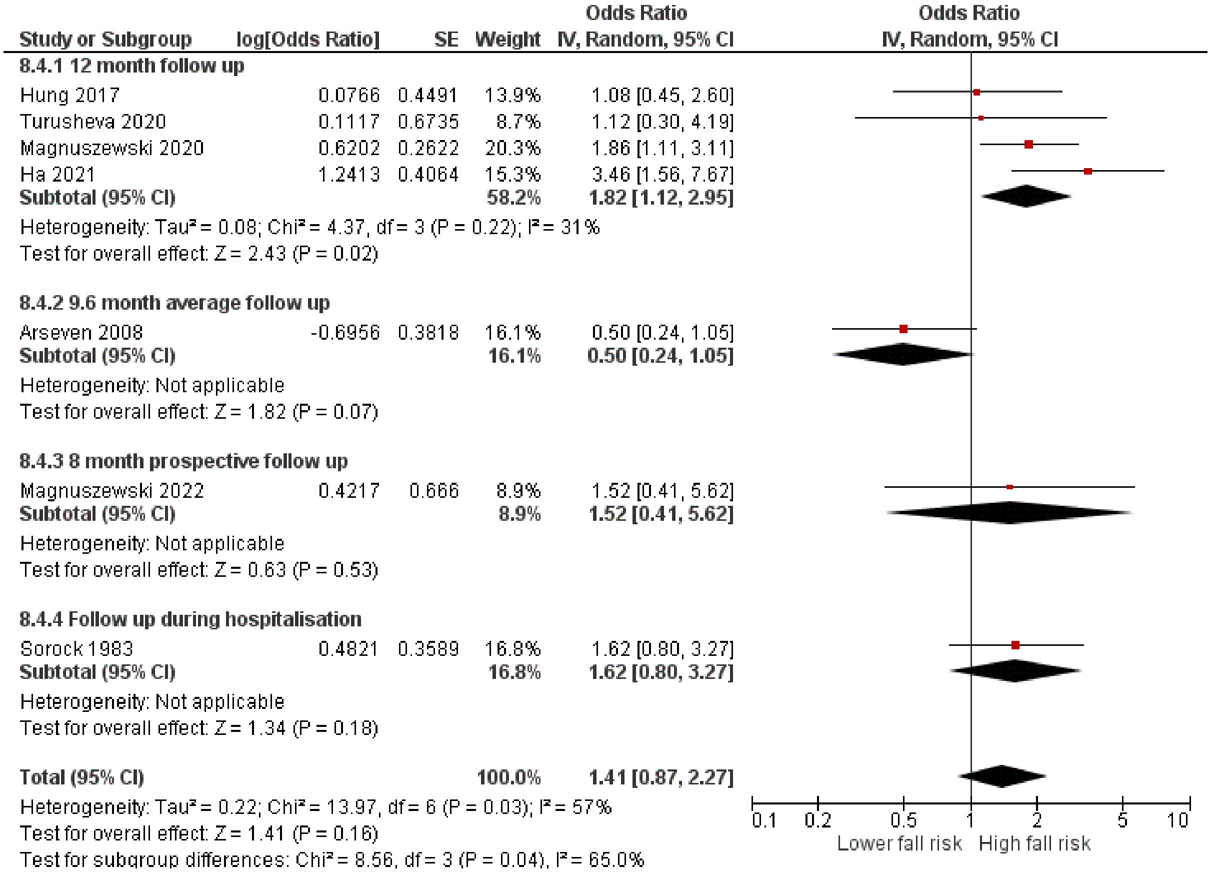

eFigure 32. Association between peripheral artery disease and falls (unadjusted OR) stratified by reporting interval

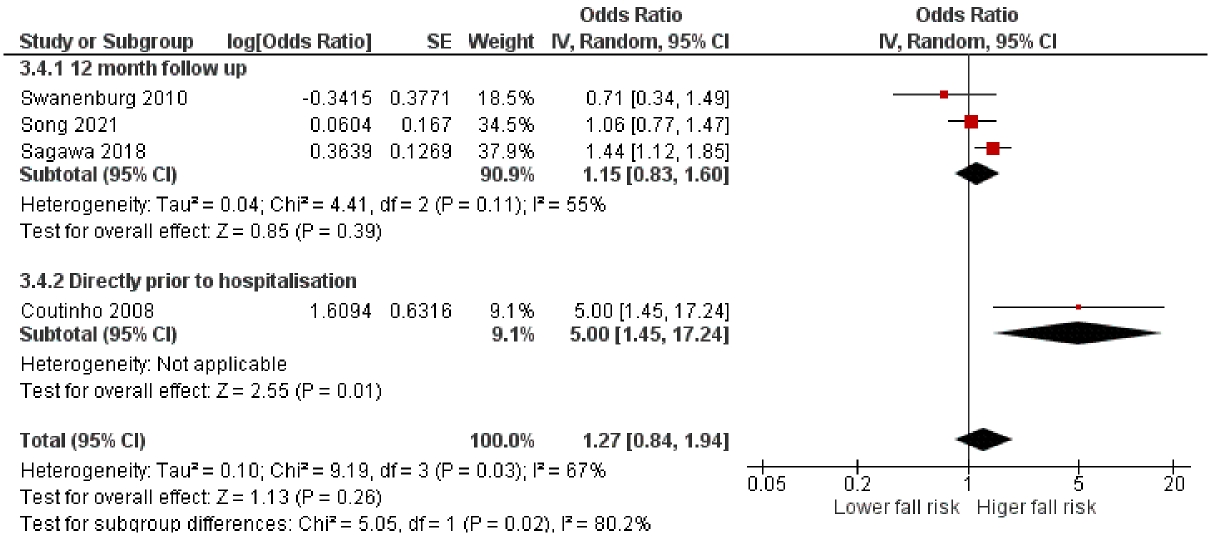

eFigure 33. Association between low blood pressure and falls (unadjusted OR) stratified by reporting interval

Study or Subgroup	log[Odds Ratio]	SE	Weight	Odds Ratio IV, Random, 95% CI
Banu 2018	-1.204	0.9142	0.3%	0.30 [0.05, 1.80]
Klein 2013 (F)	-0.5108	0.2069	3.1%	0.60 [0.40, 0.90]
Herndon 1997	-0.3567	0.1717	3.7%	0.70 [0.50, 0.98]
Kao 2012	-0.2231	0.2398	2.6%	0.80 [0.50, 1.28]
Choi 2014	-0.1278	0.0298	6.6%	0.88 [0.83, 0.93]
Jansen 2015 (a)	-0.1054	0.0601	6.1%	0.90 [0.80, 1.01]
Kelly 2003	-0.1054	0.0601	6.1%	0.90 [0.80, 1.01]
Klein 2013 (M)	-0.1054	0.2999	2.0%	0.90 [0.50, 1.62]
Tsai 2021	0	0.1139	5.0%	1.00 [0.80, 1.25]
Bhangu 2017	0	0.1139	5.0%	1.00 [0.80, 1.25]
Lee 2020	0.01	0.0259	6.6%	1.01 [0.96, 1.06]
Del Brutto 2019	0.0953	0.2306	2.8%	1.10 [0.70, 1.73]
Granek 1987	0.0953	0.2872	2.1%	1.10 [0.63, 1.93]
Heckenbach 2014	0.0953	0.1024	5.2%	1.10 [0.90, 1.34]
Jacob 2022	0.0953	0.0486	6.3%	1.10 [1.00, 1.21]
Kamali 2022	0.0953	0.6629	0.5%	1.10 [0.30, 4.03]
Sibley 2014	0.1823	0.0922	5.5%	1.20 [1.00, 1.44]
Song 2021	0.1823	0.0786	5.8%	1.20 [1.03, 1.40]
Dokuzlar 2020	0.3365	0.1717	3.7%	1.40 [1.00, 1.96]
Yasumura 1994	0.3365	0.3537	1.5%	1.40 [0.70, 2.80]
Zhao 2019	0.47	0.2277	2.8%	1.60 [1.02, 2.50]
Assantachai 2003	0.47	0.1912	3.4%	1.60 [1.10, 2.33]
Yu 2009	0.5878	0.2513	2.5%	1.80 [1.10, 2.95]
Bergland 2003	0.5878	0.2527	2.5%	1.80 [1.10, 2.95]
Chan 1997	0.5878	0.2513	2.5%	1.80 [1.10, 2.95]
Ha 2021	0.6419	0.2345	2.7%	1.90 [1.20, 3.01]
Kojima 2011	0.6419	0.5881	0.7%	1.90 [0.60, 6.02]
Gamage 2019	1.3083	0.6675	0.5%	3.70 [1.00, 13.69]
Oren 2022	2.3321	0.293	2.0%	10.30 [5.80, 18.29]
**Total (95% CI)**			**100.0%**	**1.15 [1.04, 1.27]**

Heterogeneity: $Tau^2 = 0.04$; $Chi^2 = 159.40$, df = 28 ($P < 0.00001$); $I^2 = 82\%$
Test for overall effect: Z = 2.83 (P = 0.005)

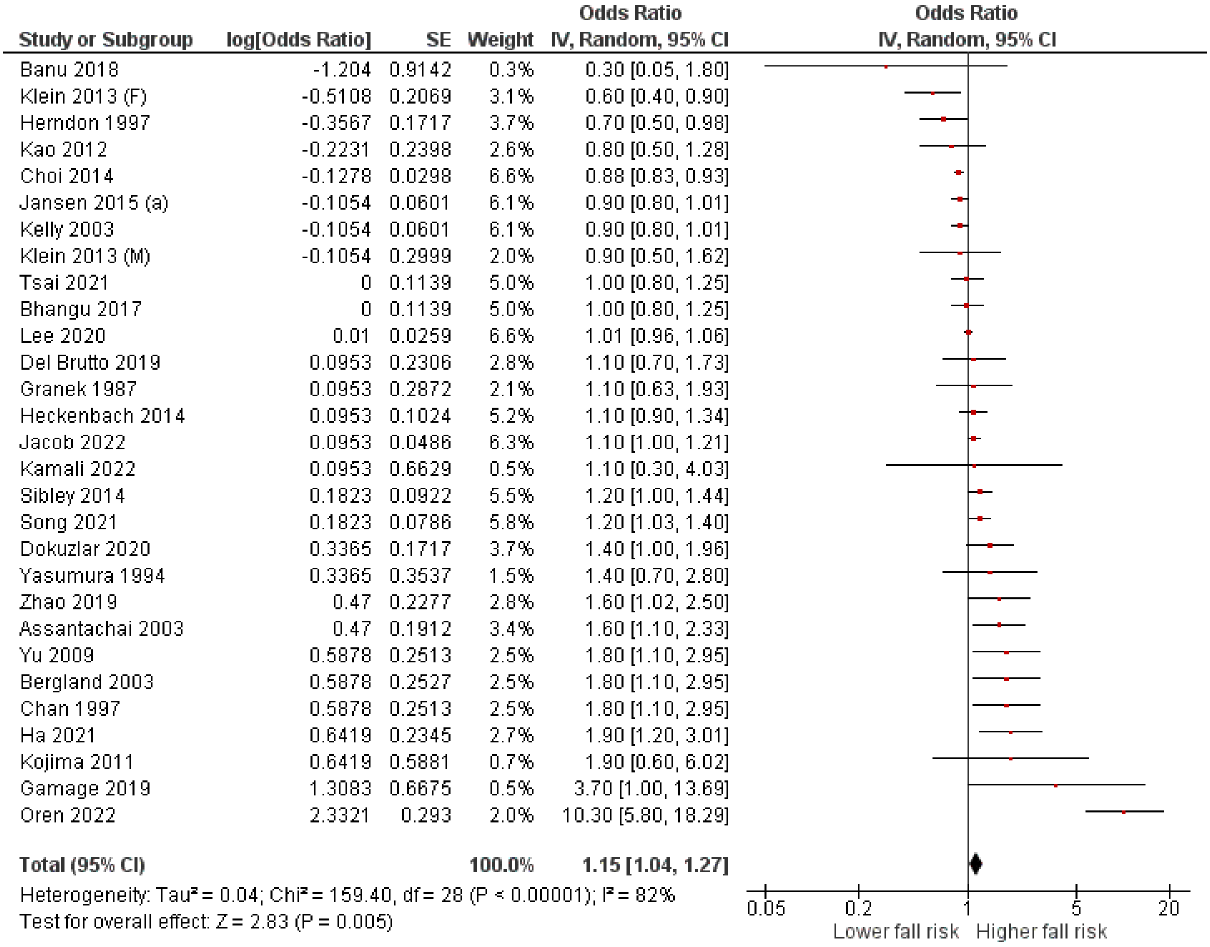

eFigure 34. Association between hypertension and falls (adjusted OR)

Study or Subgroup	log[Odds Ratio]	SE	Weight	Odds Ratio IV, Random, 95% CI
Menant 2016	-0.2231	1.061	0.2%	0.80 [0.10, 6.40]
Ek 2019 (M)	-0.1054	0.4137	0.9%	0.90 [0.40, 2.02]
Granek 1987	-0.0726	0.2864	1.6%	0.93 [0.53, 1.63]
Dahodwala 2017	0.0953	0.1625	3.3%	1.10 [0.80, 1.51]
Bhangu 2017	0.1823	0.2069	2.5%	1.20 [0.80, 1.80]
Ek 2019 (F)	0.1823	0.2069	2.5%	1.20 [0.80, 1.80]
Jia 2019	0.1823	0.0408	6.1%	1.20 [1.11, 1.30]
Grundstrom 2012	0.2624	0.0852	5.1%	1.30 [1.10, 1.54]
Lee 2020	0.2624	0.0408	6.1%	1.30 [1.20, 1.41]
Choi 2014	0.3365	0.0378	6.2%	1.40 [1.30, 1.51]
Kojima 2011	0.3365	0.6392	0.4%	1.40 [0.40, 4.90]
Valderrama-Hinds 2018	0.3365	0.1717	3.1%	1.40 [1.00, 1.96]
Dolinis 1997	0.4055	0.1582	3.4%	1.50 [1.10, 2.05]
Kelly 2003	0.4055	0.1139	4.4%	1.50 [1.20, 1.88]
Tsai 2021	0.4055	0.1139	4.4%	1.50 [1.20, 1.88]
Friedman 2002	0.47	0.1468	3.6%	1.60 [1.20, 2.13]
Jorgensen 2015	0.47	0.0681	5.6%	1.60 [1.40, 1.83]
Paliwal 2017	0.47	0.0329	6.3%	1.60 [1.50, 1.71]
Claffey 2022	0.5306	0.4527	0.8%	1.70 [0.70, 4.13]
Himes 2012	0.5306	0.0639	5.7%	1.70 [1.50, 1.93]
Magnuszewski 2020	0.5878	0.4819	0.7%	1.80 [0.70, 4.63]
Yi 2021 (F)	0.5878	0.0601	5.7%	1.80 [1.60, 2.03]
Ho 1996	0.6419	0.2345	2.1%	1.90 [1.20, 3.01]
Yu 2009	0.6419	0.2345	2.1%	1.90 [1.20, 3.01]
Abbs 2020	0.7885	0.2306	2.2%	2.20 [1.40, 3.46]
Del Brutto 2019	0.7885	0.456	0.8%	2.20 [0.90, 5.38]
Ku 2013	0.7885	0.3093	1.4%	2.20 [1.20, 4.03]
Chen 2010	0.8329	0.2911	1.6%	2.30 [1.30, 4.07]
Yi 2021 (M)	0.8329	0.0713	5.5%	2.30 [2.00, 2.64]
Ha 2021	0.9163	0.5213	0.6%	2.50 [0.90, 6.95]
Jacob 2022	0.9163	0.2606	1.9%	2.50 [1.50, 4.17]
Svensson 1992	0.977	0.453	0.8%	2.66 [1.09, 6.46]
Almegbel 2018	1.0116	0.4317	0.8%	2.75 [1.18, 6.41]
Yasumura 1994	1.0647	0.5432	0.5%	2.90 [1.00, 8.41]
Coutinho 2008	1.4586	0.4443	0.8%	4.30 [1.80, 10.27]
Salgado 1994	1.5041	0.6744	0.4%	4.50 [1.20, 16.88]
**Total (95% CI)**			**100.0%**	**1.58 [1.46, 1.72]**

Heterogeneity: Tau² = 0.03; Chi² = 140.44, df = 35 (P < 0.00001); I² = 75%
Test for overall effect: Z = 10.92 (P < 0.00001)

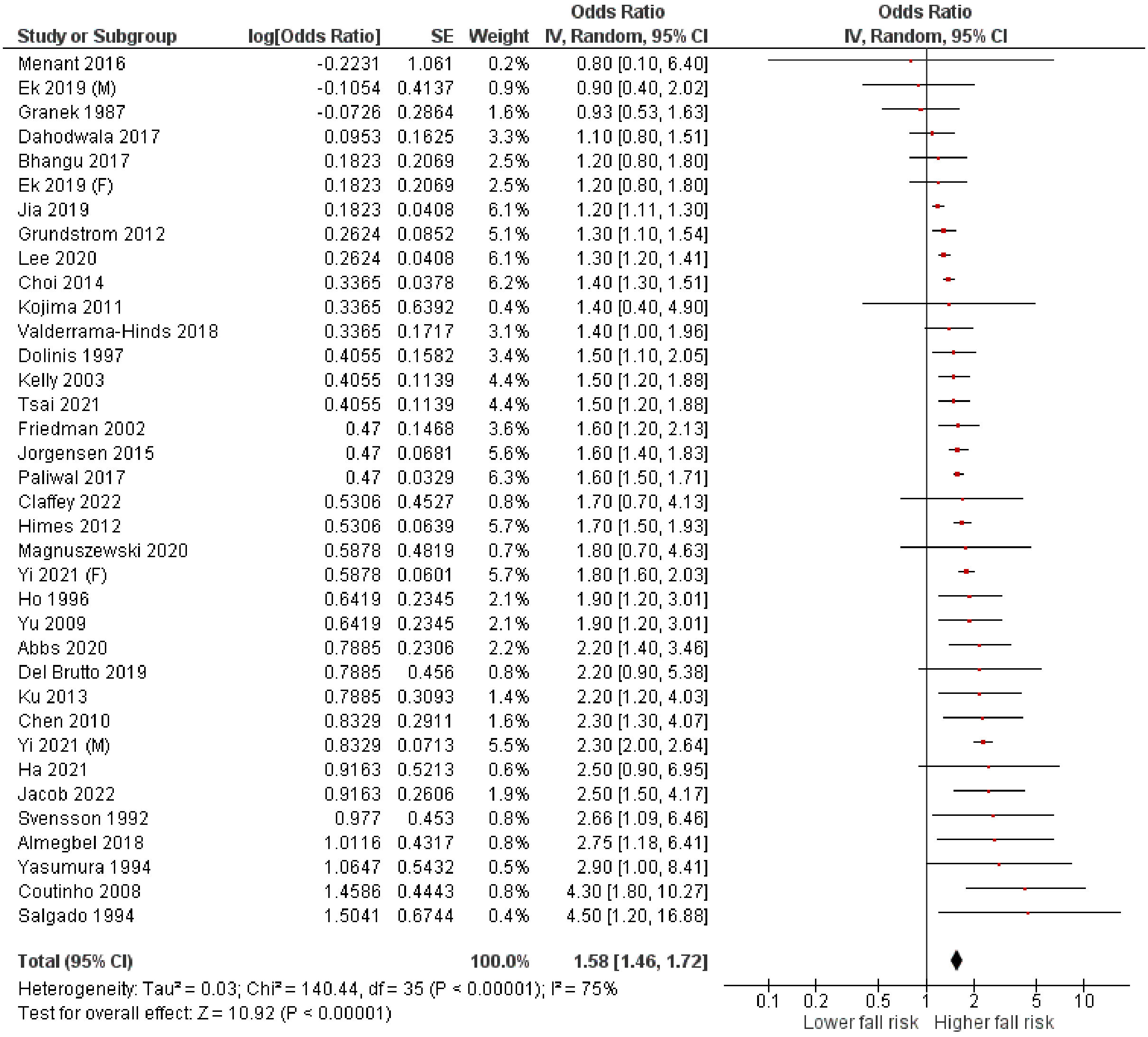

eFigure 35. Association between stroke and falls (adjusted OR)

Study or Subgroup	log[Odds Ratio]	SE	Weight	Odds Ratio IV, Random, 95% CI
Chan 1997	-0.3567	0.4323	3.4%	0.70 [0.30, 1.63]
Dokuzlar 2020	-0.1497	0.2993	5.1%	0.86 [0.48, 1.55]
Ensrud 1992	0	0.0538	9.6%	1.00 [0.90, 1.11]
Hartog 2017	0.0953	0.1625	7.8%	1.10 [0.80, 1.51]
Kamali 2022	0.0953	0.2306	6.4%	1.10 [0.70, 1.73]
Sasidharan 2022	0.2624	0.4875	2.9%	1.30 [0.50, 3.38]
Schell 2021	0.2624	0.2477	6.1%	1.30 [0.80, 2.11]
Kocyigit 2020	0.3075	0.2106	6.8%	1.36 [0.90, 2.05]
Graafmans 1996	0.3365	0.2855	5.4%	1.40 [0.80, 2.45]
Moloney 2021	0.4055	0.1139	8.8%	1.50 [1.20, 1.88]
Rutan 1992	0.4055	0.2069	6.9%	1.50 [1.00, 2.25]
Zia 2015	0.4055	0.2606	5.8%	1.50 [0.90, 2.50]
Yu 2009	0.6419	0.2789	5.5%	1.90 [1.10, 3.28]
Claffey 2022	0.6931	0.305	5.1%	2.00 [1.10, 3.64]
Menant 2016	0.8329	0.3763	4.0%	2.30 [1.10, 4.81]
Pasma 2014	0.8961	0.604	2.1%	2.45 [0.75, 8.00]
Saedon 2016	1.2809	0.3828	3.9%	3.60 [1.70, 7.62]
Zhao 2020	1.7228	0.5004	2.8%	5.60 [2.10, 14.93]
McDonald 2017	2.3321	0.9192	1.0%	10.30 [1.70, 62.41]
Magnuszewski 2022	3.0681	1.1404	0.7%	21.50 [2.30, 200.99]
**Total (95% CI)**			**100.0%**	**1.50 [1.24, 1.82]**

Heterogeneity: $Tau^2 = 0.10$; $Chi^2 = 60.65$, df = 19 ($P < 0.00001$); $I^2 = 69\%$
Test for overall effect: Z = 4.17 ($P < 0.0001$)

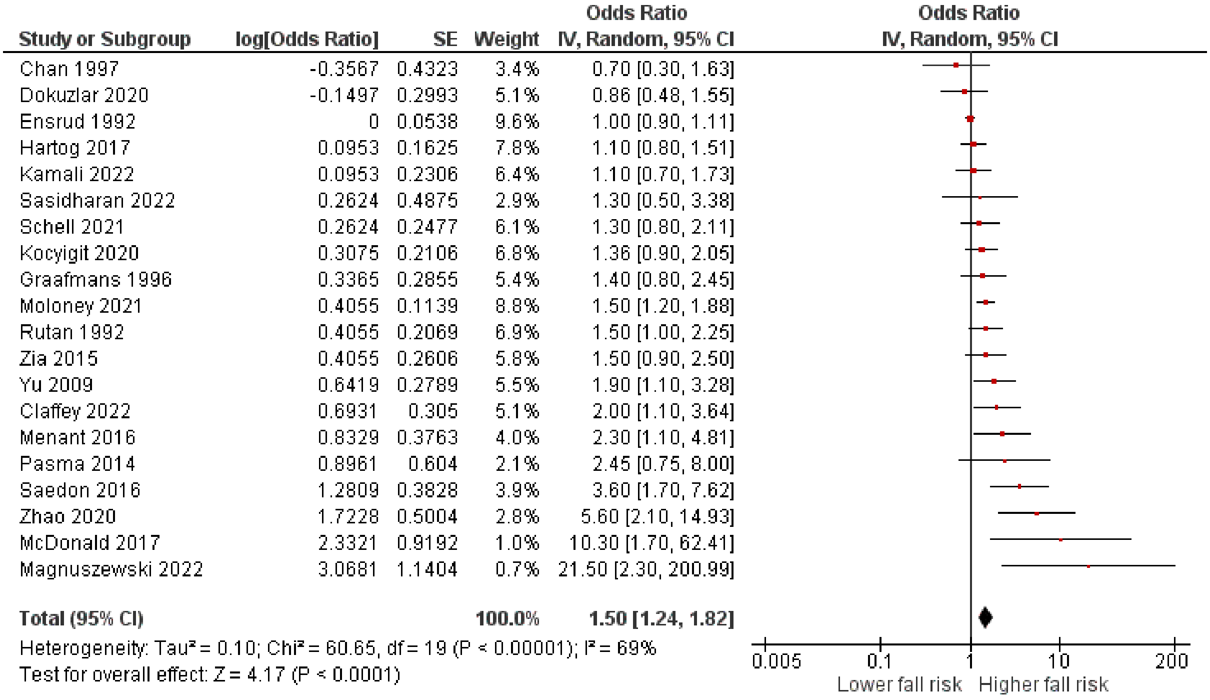

eFigure 36. Association between orthostatic hypotension and falls (adjusted OR)

Study or Subgroup	log[Odds Ratio]	SE	Weight	Odds Ratio IV, Random, 95% CI
Zhao 2019	-0.4308	0.2228	2.8%	0.65 [0.42, 1.01]
Bhangu 2017	-0.1744	0.1387	6.1%	0.84 [0.64, 1.10]
Valderrama-Hinds 2018	-0.0101	0.1087	8.5%	0.99 [0.80, 1.23]
Choi 2014	0.0953	0.0538	16.2%	1.10 [0.99, 1.22]
Tsai 2021	0.0953	0.1024	9.2%	1.10 [0.90, 1.34]
Paliwal 2017	0.157	0.0361	19.4%	1.17 [1.09, 1.26]
Damian 2013	0.174	0.4323	0.8%	1.19 [0.51, 2.78]
Rafiq 2014	0.1823	0.0444	17.9%	1.20 [1.10, 1.31]
Ku 2013	0.2469	0.2029	3.3%	1.28 [0.86, 1.91]
George 2014	0.2624	0.1876	3.8%	1.30 [0.90, 1.88]
Stenhagen 2013	0.3075	0.1514	5.3%	1.36 [1.01, 1.83]
Lawlor 2003	0.4055	0.1311	6.6%	1.50 [1.16, 1.94]
**Total (95% CI)**			**100.0%**	**1.13 [1.05, 1.23]**

Heterogeneity: Tau² = 0.01; Chi² = 21.65, df = 11 (P = 0.03); I² = 49%

Test for overall effect: Z = 3.17 (P = 0.002)

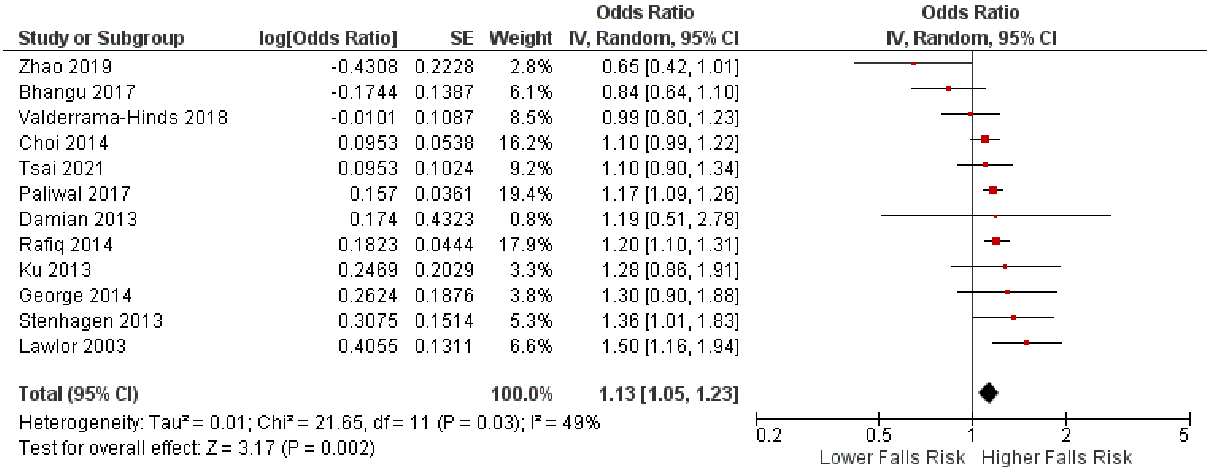

eFigure 37. Association between coronary artery disease and falls (adjusted OR)

Study or Subgroup	log[Odds Ratio]	SE	Weight	Odds Ratio IV, Random, 95% CI
O'Neal 2015	0.1823	0.093	29.7%	1.20 [1.00, 1.44]
Sanders 2012	0.1823	0.093	29.7%	1.20 [1.00, 1.44]
Bhangu 2017	0.2624	0.0852	35.4%	1.30 [1.10, 1.54]
Jansen 2015 (b)	0.3365	0.2254	5.1%	1.40 [0.90, 2.18]
**Total (95% CI)**			**100.0%**	**1.24 [1.13, 1.37]**

Heterogeneity: Tau² = 0.00; Chi² = 0.84, df = 3 (P = 0.84); I² = 0%

Test for overall effect: Z = 4.31 (P < 0.0001)

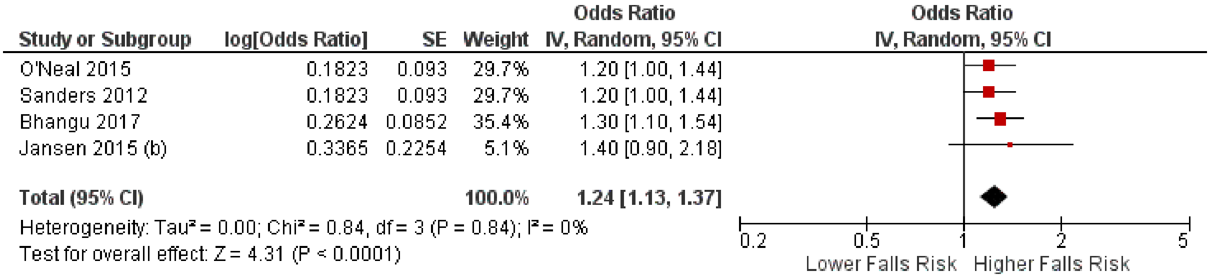

eFigure 38. Association between arrhythmia and falls (adjusted OR)

Study or Subgroup	log[Odds Ratio]	SE	Weight	Odds Ratio IV, Random, 95% CI
Lee 2020	0.0953	0.0852	15.6%	1.10 [0.93, 1.30]
Bhangu 2017	0.2624	0.2477	14.1%	1.30 [0.80, 2.11]
Jansen 2015 (a)	0.3221	0.2473	14.1%	1.38 [0.85, 2.24]
Heckenbach 2014	0.5306	0.1369	15.3%	1.70 [1.30, 2.22]
Magnuszewski 2020	0.5306	0.3245	13.1%	1.70 [0.90, 3.21]
Stenhagen 2013	0.6419	0.2345	14.3%	1.90 [1.20, 3.01]
Oren 2022	2.9392	0.2903	13.5%	18.90 [10.70, 33.39]
**Total (95% CI)**			**100.0%**	**2.09 [1.21, 3.60]**

Heterogeneity: Tau² = 0.49; Chi² = 91.81, df = 6 (P < 0.00001); I² = 93%
Test for overall effect: Z = 2.65 (P = 0.008)

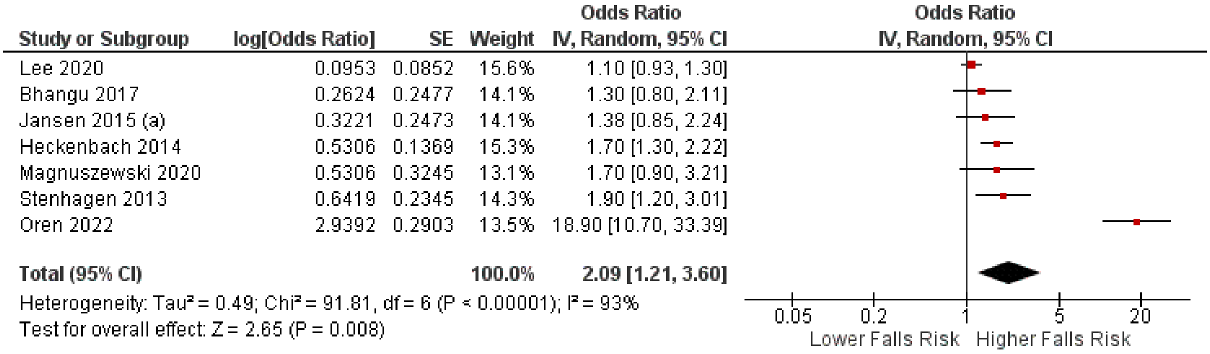

eFigure 39. Association between heart failure and falls (adjusted OR)

Appendices

Appendix 1. Systematic search strategy

Observational studies search strategy

Medline (09/01/2023)

1. accidental falls/
2. Geriatric assessment/ OR aging/ OR frail elderly/ OR exp aged/ OR middle aged/
3. 1 and 2
4. ((fall? OR fell OR falling OR fallen OR faller OR stumble? OR stumbling OR stumbles OR slip OR slips OR slipping OR slipped OR trip OR tripped or Syncope or "non-accidental falls" or "non accidental falls" or "unexplained falls") adj3 (old OR older OR senior OR elder OR elderly OR aged OR geriatric* OR middle-age? OR geriatric OR frailty OR Ageing OR elders OR Mci OR postmenopausal women OR Geriatric assessment OR aging)).ab,kw,ti
5. 3 or 4 [population]
6. exp cardiovascular diseases/ or exp hypertension/ or hypotension/ OR exp cardiac arrhythmias/ OR heart diseases/ or cardiac output, low/ or cardiomegaly/ or cardiomyopathies/ or heart failure/ or heart valve diseases/ or myocardial ischemia/ or ventricular dysfunction/ or ventricular outflow obstruction/ OR aortic valve insufficiency/ OR aortic valve stenosis/ OR mitral valve insufficiency/ OR mitral valve stenosis/ OR pulmonary valve insufficiency/ OR pulmonary valve stenosis/ OR tricuspid valve insufficiency/ OR tricuspid valve stenosis/ OR heart murmurs/ OR systolic murmurs/ OR Peripheral Arterial Disease/ OR Intermittent Claudication/ OR Peripheral Vascular Diseases/ OR Angina Pectoris/ OR exp Stroke/ OR Ischemic Attack, Transient/ OR Vascular Stiffness/ or Antihypertensive Agents/
7. (cardiovascular disease? or hypertension or hypotension or circulatory disease?).ab,kw,ti
8. blood pressure/ or myocardial ischemia/ or prehypertension/ OR Hypotension/
9. (blood pressure or systolic pressure or diastolic pressure).ab,kw,ti
10. ((cardiac OR cardiovascular OR heart) adj3 (disorder? or disease? or abnormalit* or failure or dysfunction*)).ab,kw,ti
11. (irregular heartbeat OR Sinus node disease OR Atrial fibrillation OR Bradycardia OR valve disease*).ab,kw,ti
12. ((valv* OR mitral OR tricuspid OR pulmonary OR aortic) adj3 (insuffic* OR incompet* or stenos* or disease? or regurgitation)).ab,kw,ti
13. (cardiomyopath* OR Myocardial ischemia OR Myocardial infarction OR carotid sinus OR orthostasis OR orthostatic hypotension OR postural hypotension OR postprandial hypotension OR vasovagal syncope OR Neurocardiogenic syncope OR arrhythmia OR ventricular dysfunction OR heart murmur* OR cardiac murmur* OR peripheral arterial disease OR peripheral vascular disease OR angina OR stroke OR TIA OR transient isch?emic attack OR arterial stiffness OR Structural cardiovascular abnormalit*).ab,kw,ti
14. or/6-13 [cardiovascular diseases and -parameters]
15. 5 and 14

16. 14 and (fall? OR fell OR falling OR fallen OR faller OR stumble? OR stumbling OR stumbles OR slip OR slips OR slipping OR slipped OR trip OR tripped).ab,kw,ti
17. (older adult? or elderly).ab,kw,ti.
18. 16 and 17
19. 15 or 18

Embase (09/01/2023)

1. Falling/exp
2. Geriatric assessment/de OR aging/exp OR frail elderly/de OR aged/exp OR middle aged/de
3. #1 and #2
4. ((fall? OR fell OR falling OR fallen OR faller OR stumble? OR stumbling OR stumbles OR slip OR slips OR slipping OR slipped OR trip OR tripped OR 'faintness' OR "non-accidental falls" OR "non accidental falls" OR "unexplained falls") NEAR/3 (old OR older OR senior OR elder OR elderly OR aged OR geriatric* OR middle-age? OR geriatric OR frailty OR Ageing OR elders OR Mci OR 'postmenopausal wom?n' OR 'Geriatric assessment*' OR aging)):ti,ab,kw
5. #3 or #4 [population]
6. cardiovascular disease/de or hypertension/exp or ecg abnormality/de or 'heart arrhythmia'/exp or 'heart failure'/exp or 'ischemic heart disease'/exp or 'myocardial disease'/exp or 'valvular heart disease'/exp or 'coronary artery disease'/exp or 'aortic valve stenosis'/exp OR 'mitral valve stenosis'/exp OR 'pulmonary valve stenosis'/exp OR 'tricuspid valve stenosis'/exp OR 'aortic valve disease'/exp OR 'mitral valve disease'/exp OR 'pulmonary valve disease'/exp OR 'tricuspid valve disease'/exp OR 'aortic regurgitation'/exp OR 'mitral valve regurgitation'/exp OR 'pulmonary valve insufficiency'/exp OR 'tricuspid valve regurgitation'/exp OR 'heart murmur'/exp OR 'systolic heart murmur'/exp OR 'peripheral vascular disease'/exp OR 'peripheral occlusive artery disease'/exp OR 'hypotension'/exp or 'angina pectoris'/exp OR 'cerebrovascular accident'/exp OR 'transient ischemic attack'/exp OR 'arterial stiffness'/exp OR 'antihypertensive agent'/mj
7. (cardiovascular disease? or hypertension or hypotension or circulatory disease?):ti,ab,kw
8. 'blood pressure'/de
9. ('blood pressure' or 'systolic pressure' or 'diastolic pressure'):ti,ab,kw
10. (((cardiac OR cardiovascular OR heart) NEAR/3 (disorder? or disease? or abnormalit* or failure or dysfunction*)) OR irregular heartbeat OR 'Sinus node disease' OR 'Atrial fibrillation' OR Bradycardia OR 'valve disease*' OR ((valv* OR mitral OR tricuspid OR pulmonary OR aortic) NEAR/3 (insuffic* OR incompet* or stenos* or disease? or regurgitation)) OR cardiomyopath* OR 'Myocardial ischemia' OR 'Myocardial infarction' OR 'carotid sinus' OR orthostasis OR 'orthostatic hypotension' OR 'postural hypotension' OR 'postprandial hypotension' OR 'vasovagal syncope' OR 'Neurocardiogenic syncope' OR arrhythmia or 'ventricular dysfunction' OR 'heart murmur*' OR 'cardiac murmur*' OR 'peripheral arterial disease' OR 'peripheral vascular disease' OR angina OR stroke OR TIA

OR ‘transient isch*emic attack’ OR ‘arterial stiffness’ OR ‘structural cardiovascular abnormalit*’):ti,ab,kw
11. #6 OR #7 OR #8 OR #9 OR #10 [cardiovascular diseases and -parameters]
12. #5 and #11
13. #11 and (fall? OR fell OR falling OR fallen OR faller OR stumble? OR stumbling OR stumbles OR slip OR slips OR slipping OR slipped OR trip OR tripped):ti,ab,kw
14. (‘older adult?’ or elderly or aged):ti,ab,kw
15. #13 and #14
16. #12 OR #15

Interventional studies search strategy

Medline (09/01/2023)

1. accidental falls/
2. Geriatric assessment/ OR aging/ OR frail elderly/ OR exp aged/ OR middle aged/
3. 1 and 2
4. ((fall? OR fell OR falling OR fallen OR faller OR stumble? OR stumbling OR stumbles OR slip OR slips OR slipping OR slipped OR trip OR tripped or Syncope or "non-accidental falls" or "non accidental falls" or "unexplained falls") adj3 (old OR older OR senior OR elder OR elderly OR aged OR geriatric* OR middle-age? OR geriatric OR frailty OR Ageing OR elders OR Mci OR postmenopausal women OR Geriatric assessment OR aging)).ab,kw,ti
5. 3 or 4 [population]
6. exp cardiovascular diseases/ or exp hypertension/ or hypotension/ OR exp cardiac arrhythmias/ OR heart diseases/ or cardiac output, low/ or cardiomegaly/ or cardiomyopathies/ or heart failure/ or heart valve diseases/ or myocardial ischemia/ or ventricular dysfunction/ or ventricular outflow obstruction/ OR aortic valve insufficiency/ OR aortic valve stenosis/ OR mitral valve insufficiency/ OR mitral valve stenosis/ OR pulmonary valve insufficiency/ OR pulmonary valve stenosis/ OR tricuspid valve insufficiency/ OR tricuspid valve stenosis/ OR heart murmurs/ OR systolic murmurs/ OR Peripheral Arterial Disease/ OR Intermittent Claudication/ OR Peripheral Vascular Diseases/ OR Angina Pectoris/ OR exp Stroke/ OR Ischemic Attack, Transient/ OR Vascular Stiffness/ or Antihypertensive Agents/
7. (cardiovascular disease? or hypertension or hypotension or circulatory disease?).ab,kw,ti
8. blood pressure/ or myocardial ischemia/ or prehypertension/ OR Hypotension/
9. (blood pressure or systolic pressure or diastolic pressure).ab,kw,ti
10. ((cardiac OR cardiovascular OR heart) adj3 (disorder? or disease? or abnormalit* or failure or dysfunction*)).ab,kw,ti
11. (irregular heartbeat OR Sinus node disease OR Atrial fibrillation OR Bradycardia OR valve disease*).ab,kw,ti
12. ((valv* OR mitral OR tricuspid OR pulmonary OR aortic) adj3 (insuffic* OR incompet* or stenos* or disease? or regurgitation)).ab,kw,ti

13. (cardiomyopath* OR Myocardial ischemia OR Myocardial infarction OR carotid sinus OR orthostasis OR orthostatic hypotension OR postural hypotension OR postprandial hypotension OR vasovagal syncope OR Neurocardiogenic syncope OR arrhythmia or ventricular dysfunction OR heart murmur OR cardiac murmur OR peripheral arterial disease OR peripheral vascular disease OR angina OR stroke OR TIA OR transient isch?emic attack OR arterial stiffness OR Structural cardiovascular abnormalit*).ab,kw,ti
14. or/6-13 [cardiovascular diseases and -parameters]
15. 5 and 14
16. 14 and (fall? OR fell OR falling OR fallen OR faller OR stumble? OR stumbling OR stumbles OR slip OR slips OR slipping OR slipped OR trip OR tripped).ab,kw,ti
17(older adult? or elderly).ab,kw,ti.
18. 16 and 17
19. 15 or 18
20. exp Randomized Controlled Trials as Topic/
21. Clinical Trials as Topic/
22. exp Clinical Trial/
23. random*.ab,kw,ti.
24. Intervention*.ab,kw,ti.
25. Cardiac Pacing, Artificial/
26. (pace* or pacing).ab,kw,ti.
27. or/20-26
28. 19 and 27

Embase (09/01/2023)

1. Falling/exp
2. Geriatric assessment/de OR aging/exp OR frail elderly/de OR aged/exp OR middle aged/de
3. #1 and #2
4. ((fall? OR fell OR falling OR fallen OR faller OR stumble? OR stumbling OR stumbles OR slip OR slips OR slipping OR slipped OR trip OR tripped OR 'faintness' OR "non-accidental falls" OR "non accidental falls" OR "unexplained falls") NEAR/3 (old OR older OR senior OR elder OR elderly OR aged OR geriatric* OR middle-age? OR geriatric OR frailty OR Ageing OR elders OR Mci OR ‘postmenopausal wom?n’ OR ‘Geriatric assessment*’ OR aging)):ti,ab,kw
5. #3 or #4 [population]
6. cardiovascular disease/de or hypertension/exp or ecg abnormality/de or 'heart arrhythmia'/exp or ‘heart failure’/exp or ‘ischemic heart disease’/exp or ‘myocardial disease’/exp or ‘valvular heart disease’/exp or ‘coronary artery disease’/exp or 'aortic valve stenosis'/exp OR 'mitral valve stenosis'/exp OR 'pulmonary valve stenosis'/exp OR 'tricuspid valve stenosis'/exp OR 'aortic valve disease'/exp OR 'mitral valve disease'/exp OR 'pulmonary valve disease'/exp OR 'tricuspid valve disease'/exp OR 'aortic regurgitation'/exp OR 'mitral valve regurgitation'/exp OR 'pulmonary valve insufficiency'/exp OR 'tricuspid valve

regurgitation'/exp OR 'heart murmur'/exp OR 'systolic heart murmur'/exp OR 'peripheral vascular disease'/exp OR 'peripheral occlusive artery disease'/exp OR 'hypotension'/exp or 'angina pectoris'/exp OR 'cerebrovascular accident'/exp OR 'transient ischemic attack'/exp OR 'arterial stiffness'/exp OR 'antihypertensive agent'/mj
7. (cardiovascular disease? or hypertension or hypotension or circulatory disease?):ti,ab,kw
8. ‘blood pressure’/de
9. (‘blood pressure’ or ‘systolic pressure’ or ‘diastolic pressure’):ti,ab,kw
10. (((cardiac OR cardiovascular OR heart) NEAR/3 (disorder? or disease? or abnormalit* or failure or dysfunction*)) OR irregular heartbeat OR ‘Sinus node disease’ OR ‘Atrial fibrillation’ OR Bradycardia OR ‘valve disease*’ OR ((valv* OR mitral OR tricuspid OR pulmonary OR aortic) NEAR/3 (insuffic* OR incompet* or stenos* or disease? or regurgitation)) OR cardiomyopath* OR ‘Myocardial ischemia’ OR ‘Myocardial infarction’ OR ‘carotid sinus’ OR orthostasis OR ‘orthostatic hypotension’ OR ‘postural hypotension’ OR ‘postprandial hypotension’ OR ‘vasovagal syncope’ OR ‘Neurocardiogenic syncope’ OR arrhythmia or ‘ventricular dysfunction’ OR ‘heart murmur*’ OR ‘cardiac murmur*’ OR ‘peripheral arterial disease’ OR ‘peripheral vascular disease’ OR angina OR stroke OR TIA OR ‘transient isch*emic attack’ OR ‘arterial stiffness’ OR ‘structural cardiovascular abnormalit*’):ti,ab,kw
11. #6 OR #7 OR #8 OR #9 OR #10 [cardiovascular diseases and -parameters]
12. #5 and #11
13. #11 and (fall? OR fell OR falling OR fallen OR faller OR stumble? OR stumbling OR stumbles OR slip OR slips OR slipping OR slipped OR trip OR tripped):ti,ab,kw
14. (‘older adult?’ or elderly or aged):ti,ab,kw
15. #13 and #14
16. (#12 OR #15)
17. 'randomized controlled trial'/exp
18. 'clinical trial'/exp
19. 'intervention study'/exp
20. random*:ti,ab,kw OR intervention*:ti,ab,kw
21. 'cardiac rhythm management device'/exp
22. #17 OR #18 OR #19 OR #20 OR #21
23. #16 AND #22

Appendix 2. Full text screening table of excluded studies

First author and year	Article title	Reason for exclusion
**Abbs 2019**	Novel risk factors for falls in older adults experiencing homelessness: Results from the hope home cohort study	Brief report, narrative review, editorial, conference abstract, or not a primary research paper
**Abe 2019**	Differences in the prevalence of and factors associated with frailty in five Japanese residential areas	No assessment of associations between CV disorders and falls, or information on prevalence
**Abey-Nesbit 2021**	Risk factors for injuries in New Zealand older adults with complex needs: a national population retrospective study	No assessment of associations between CV disorders and falls, or information on prevalence
**AbuBakar 2021**	Older Adults with Hypertension: Prevalence of Falls and Their Associated Factors	Specific disease/condition defined population
**Aizen 2007**	Risk factors and characteristics of falls during inpatient rehabilitation of elderly patients	No assessment of associations between CV disorders and falls, or information on prevalence
**Aizen 2013**	Prediction of falls in rehabilitation and acute care geriatric setting	No assessment of associations between CV disorders and falls, or information on prevalence
**Akarirmak 2019**	Risk factors associated with falls in inpatients of a physical medicine and rehabilitation clinic	Brief report, narrative review, editorial, conference abstract, or not a primary research paper
**al Tehewy 2015**	A Study of Rate and Predictors of Fall Among Elderly Patients in a University Hospital	No assessment of associations between CV disorders and falls, or information on prevalence
**Alagiakrishnan 2014**	Postural and postprandial hypotension and its association with frailty in hospitalized elderly-a pilot study	Brief report, narrative review, editorial, conference abstract, or not a primary research paper
**Andersen 2015**	Results of specialized geriatric fall clinic intervention for older patients with orthostatic intolerance	Brief report, narrative review, editorial, conference abstract, or not a primary research paper
**Ang 2018**	A Systematic Review and Meta-Analyses of the Association Between Anti-Hypertensive Classes and the Risk of Falls Among Older Adults	Brief report, narrative review, editorial, conference abstract, or not a primary research paper
**Arita 2019**	Impact of Atrial Fibrillation on Falls in Older Patients: Which is a Problem, Existence or Persistence?	Population below minimum age criteria
**Asensio 2015**	Postprandial hypotension in the elderly: Findings in a Mexican population	No assessment of associations between CV disorders and falls, or information on prevalence
**Atkins 2019**	Impact of Low Cardiovascular Risk Profiles on Geriatric Outcomes: Evidence From 421,000 Participants in Two Cohorts	No assessment of associations between CV disorders and falls, or information on prevalence
**Attar 2021**	Common Types of Falls in the Elderly Population, Their Associated Risk Factors and Prevention in a Tertiary Care Center	No assessment of associations between CV disorders and falls, or information on prevalence
**Aubert 2021**	Clinical outcomes of modifying hypertension treatment intensity in older adults treated to low blood pressure	No assessment of associations between CV disorders and falls, or information on prevalence
**Badeshae 2022**	Factors contributing to bed block and emergency department ramping: a prospective audit on proportion of medical inpatients at sir charles gairdner hospital not requiring tertiary care	Brief report, narrative review, editorial, conference abstract, or not a primary research paper

First author and year	Article title	Reason for exclusion
**Banach 2014**	Association of systolic blood pressure levels with cardiovascular events and all-cause mortality among older adults taking antihypertensive medication	Population below minimum age criteria
**Bao 2017**	Comorbidity increased the risk of falls in chinese older adults: A cross-sectional study	Unable to locate full text
**Batko-Szwaczka 2022**	Predictors of Adverse Outcomes in Healthy Aging Adults: Coronary Artery Disease, Lower Educational Status and Higher P-Selectin Levels	No assessment of associations between CV disorders and falls, or information on prevalence
**Bhangu 2014**	Fuse: Falls and unexplained syncope in the elderly, the utility of implantable loop recorders	Brief report, narrative review, editorial, conference abstract, or not a primary research paper
**Bhangu 2016**	Long-term cardiac monitoring in older adults with unexplained falls and syncope	Specific disease/condition defined population
**Blake 1988**	Falls by elderly people at home: Prevalence and associated factors	No assessment of associations between CV disorders and falls, or information on prevalence
**Boockvar 2018**	Antihypertensive drug de-intensification and recurrent falls in long-term care	Brief report, narrative review, editorial, conference abstract, or not a primary research paper
**Borodin 2017**	Morbidity and mortality in elderly presenting to the emergency department with generalized weakness	Brief report, narrative review, editorial, conference abstract, or not a primary research paper
**Botwinick 2016**	Geriatric nursing home falls: A single institution cross-sectional study	Specific disease/condition defined population
**Brandi 2002**	[Retrospective survey on falls among hospitalized patients]	Unable to locate full text
**Brenner 2017**	Reduction of falls and fractures after permanent pacemaker implantation in elderly patients with sinus node dysfunction	Specific disease/condition defined population
**Bress 2021**	Patient Selection for Intensive Blood Pressure Management Based on Benefit and Adverse Events	No assessment of associations between CV disorders and falls, or information on prevalence
**Bromfield 2017**	Blood Pressure, Antihypertensive Polypharmacy, Frailty, and Risk for Serious Fall Injuries among Older Treated Adults with Hypertension	Specific disease/condition defined population
**Bruhn 2014**	Low blood pressure - Increased risk of falling: Study finds correlation in elderly patients	Unable to locate full text
**Butt 2013**	The risk of falls on initiation of antihypertensive drugs in the elderly	No assessment of associations between CV disorders and falls, or information on prevalence
**CaceresSantana 2022**	Incidence of falls in long-stay hospitals: risk factors and strategies for prevention	No assessment of associations between CV disorders and falls, or information on prevalence
**Callisaya 2014**	Greater daily defined dose of antihypertensive medication increases the risk of falls in older people - A population-based study	No assessment of associations between CV disorders and falls, or information on prevalence

First author and year	Article title	Reason for exclusion
**Campos 2010**	Risk of fall from standing height is double in patients who take antihypertensive medicaments: Study in a cohort of hip fracture patients in a central hospital in Portugal	Brief report, narrative review, editorial, conference abstract, or not a primary research paper
**Canaslan 2022**	Predictivity of the comorbidity indices for geriatric syndromes	No assessment of associations between CV disorders and falls, or information on prevalence
**Capone 2013**	Serious fall injuries in hospitalized patients with and without cancer	No assessment of associations between CV disorders and falls, or information on prevalence
**Carpenter 2009**	Assessing three-month fall risk for geriatric emergency department patients	Brief report, narrative review, editorial, conference abstract, or not a primary research paper
**Castaldo 2017**	Fall Prediction in Hypertensive Patients via Short-Term HRV Analysis	Specific disease/condition defined population
**Catikkas 2022**	Prevalence and determinants of falls in community dwelling older adults: A population based cross sectional study	Brief report, narrative review, editorial, conference abstract, or not a primary research paper
**Caton 2010**	Identifying fall risk factors among nursing home patients	Brief report, narrative review, editorial, conference abstract, or not a primary research paper
**Caton 2011**	Examining the relationship between disease burden and falls among nursing home residents	Brief report, narrative review, editorial, conference abstract, or not a primary research paper
**Chang 2016**	Geriatric conditions are associated with potential difficulty of walking a distance of 200-300 meters among older adults in Taiwan	Brief report, narrative review, editorial, conference abstract, or not a primary research paper
**Chang 2019**	Factors associated with increasing geriatric conditions in older adults: Results of a population-based study in Taiwan	Brief report, narrative review, editorial, conference abstract, or not a primary research paper
**Chang 2019**	Recurrent syncope is not an independent risk predictor for future syncopal events or adverse outcomes	No assessment of associations between CV disorders and falls, or information on prevalence
**Chao 2019**	Risk Factors for Poor Functional Recovery, Mortality, Recurrent Fractures, and Falls Among Patients Participating in a Fracture Liaison Service Program	Specific disease/condition defined population
**Charlesworth 2016**	Functional Status and Antihypertensive Therapy in Older Adults: A New Perspective on Old Data	No assessment of associations between CV disorders and falls, or information on prevalence
**Chen 2019**	Identify the alteration of balance control and risk of falling in stroke survivors during obstacle crossing based on kinematic analysis	No assessment of associations between CV disorders and falls, or information on prevalence
**Cheng 1998**	The sit-to-stand movement in stroke patients and its correlation with falling	No assessment of associations between CV disorders and falls, or information on prevalence
**Choi 2010**	Fall risk in low-income elderly people in one urban area	Unable to locate full text
**Cigolle 2007**	Geriatric conditions and disability: The health and retirement study	No assessment of associations between CV disorders and falls, or information on prevalence

First author and year	Article title	Reason for exclusion
**Cimilli Ozturk 2017**	Factors Associated With Multiple Falls Among Elderly Patients Admitted to Emergency Department	Specific disease/condition defined population
**Clark 2016**	Predicting postural hypotension, falls, and cognitive impairment: The InCHIANTI study	Population below minimum age criteria
**Clark 2018**	Detecting Risk Of Postural hypotension (DROP): derivation and validation of a prediction score for primary care	Population below minimum age criteria
**Coveney 2013**	Location, location, location; acute geriatric units in the Irish setting	No assessment of associations between CV disorders and falls, or information on prevalence
**Crilley 1997**	Permanent cardiac pacing in elderly patients with recurrent falls, dizziness and syncope, and a hypersensitive cardioinhibitory reflex	Brief report, narrative review, editorial, conference abstract, or not a primary research paper
**Dahodwala 2014**	Mild parkinsonian signs are a risk factor for falls	Not a randomised controlled trial
**Dai 2018**	Falls and Recurrent Falls among Adults in A Multi-ethnic Asian Population: The Singapore Epidemiology of Eye Diseases Study	Brief report, narrative review, editorial, conference abstract, or not a primary research paper
**Dalgaard 2019**	Rate or Rhythm Control in Older Atrial Fibrillation Patients: Risk of Fall-Related Injuries and Syncope	Population below minimum age criteria
**Dallmeier 2016**	A prospective assessment of cardiac biomarkers for hemodynamic stress and necrosis and the risk of falls among older people: the ActiFE study	No assessment of associations between CV disorders and falls, or information on prevalence
**Damluji 2020**	Physical frailty and five-year outcomes among older adults in the national health and aging trends study (nhats)	Population below minimum age criteria
**Damluji 2021**	Frailty and cardiovascular outcomes in the National Health and Aging Trends Study	No assessment of associations between CV disorders and falls, or information on prevalence
**Dartigues 2022**	Co-Occurrence of Geriatric Syndromes and Diseases in the General Population: Assessment of the Dimensions of Aging	No assessment of associations between CV disorders and falls, or information on prevalence
**de Albuquerque Sousa 2012**	Frailty syndrome and associated factors in community-dwelling elderly in Northeast Brazil	No assessment of associations between CV disorders and falls, or information on prevalence
**De Carvalho 2016**	Prevalence of orthostatic hypotension	No assessment of associations between CV disorders and falls, or information on prevalence
**Del Brutto 2020**	Frailty and Risk of Falls in Community-Dwelling Older Adults Living in a Rural Setting. The Atahualpa Project	No assessment of associations between CV disorders and falls, or information on prevalence
**DePaola 2022**	Orthostatic hypertension and the risk of major adverse events: A systematic review and meta-analysis	Brief report, narrative review, editorial, conference abstract, or not a primary research paper
**Dhar 2022**	The Prevalence and Associated Risk Factors of Fear of Fall in the Elderly: A Hospital-Based, Cross-Sectional Study	No assessment of associations between CV disorders and falls, or information on prevalence
**Dillon 2017**	The effect of gaps in adherence to anti hypertensive medication on falls risk in older adults	Brief report, narrative review, editorial, conference abstract, or not a primary research paper
**Dillon 2017**	Poor medication adherence in the relationship between antihypertensive medication and falls: A prospective cohort study of older adults	Brief report, narrative review, editorial, conference abstract, or not a primary research paper

First author and year	Article title	Reason for exclusion
**Dillon 2019**	Association between gaps in antihypertensive medication adherence and injurious falls in older community-dwelling adults: a prospective cohort study	No assessment of associations between CV disorders and falls, or information on prevalence
**Dokuzlar 2020**	Assessment of factors that increase risk of falling in older women by four different clinical methods	No assessment of associations between CV disorders and falls, or information on prevalence
**Donnell 2022**	The 'Bermuda Triangle' of Orthostatic Hypotension, Cognitive Impairment and Reduced Mobility: Associations with Falls and Fractures in Community-dwelling Older People	Brief report, narrative review, editorial, conference abstract, or not a primary research paper
**Dundar 2020**	Presenting symptoms of older emergency department patients: a single-center experience of 10,692 patients in Turkey	No assessment of associations between CV disorders and falls, or information on prevalence
**Dwyer 2021**	Residential aged care homes: Why do they call â€˜000â€™? A study of the emergency prehospital care of older people living in residential aged care homes	No assessment of associations between CV disorders and falls, or information on prevalence
**Falcao 2019**	Risk of falls in hospitalized elderly people	No assessment of associations between CV disorders and falls, or information on prevalence
**Ferreira 2021**	Analysis of the Nursing Diagnosis Risk for Falls in Older Adults with Hypertension	No assessment of associations between CV disorders and falls, or information on prevalence
**Finucane 2015**	Impaired orthostatic blood pressure stabilization is highly prevalent and a novel risk factor for unexplained falls in older adults: Findings from a prospective national cohort study	Brief report, narrative review, editorial, conference abstract, or not a primary research paper
**Finucane 2016**	Delayed BP recovery on standing is associated with unexplained and injurious falls	Brief report, narrative review, editorial, conference abstract, or not a primary research paper
**Finucane 2017**	Coexisting hypertension and orthostatic hypotension increases the risk of falls in older adults: Findings from The Irish Longitudinal Study on Ageing (TILDA)	Brief report, narrative review, editorial, conference abstract, or not a primary research paper
**Formiga 2018**	Prevalence and clinical significance of interatrial block in very older persons	No assessment of associations between CV disorders and falls, or information on prevalence
**Francois 2016**	Cost-effectiveness of droxidopa in patients with neurogenic orthostatic hypotension: post-hoc economic analysis of Phase 3 clinical trial data	Population below minimum age criteria
**Frisoli 2015**	Frailty predictors and outcomes among older patients with cardiovascular disease: Data from Fragicor	No assessment of associations between CV disorders and falls, or information on prevalence
**Gailland 2017**	Patient's falls during hospitalization: Impact of newly prescribed fall risk-increasing drugs	Unable to locate full text
**Galanopoulou 2021**	The Correlation of the History of Cardiopathy with the Possibility of Fall Injury in Elderly Patients Who Came To The Primary Health Care Center (PHCC)	Brief report, narrative review, editorial, conference abstract, or not a primary research paper
**Gamboa-Esparza 2022**	Heart disease in people older than 95 years-the Cardiogeriatric Clinic experience in Mexico	Brief report, narrative review, editorial, conference abstract, or not a primary research paper
**Glover 2022**	Standardizing the approach to postural hypotension: a quality improvement project	Brief report, narrative review, editorial, conference abstract, or not a primary research paper
**Gnjidic 2015**	Ischemic heart disease, prescription of optimal medical therapy and geriatric syndromes in community-dwelling older men: A population-based study	No assessment of associations between CV disorders and falls, or information on prevalence

First author and year	Article title	Reason for exclusion
**Goh 2015**	The evaluation of new index for blood pressure variability using posture change among older fallers	No assessment of associations between CV disorders and falls, or information on prevalence
**Goldstein 2017**	A risk model for falls in older patients after hospitalization for acute myocardial infarction: The SILVER-AMI study	Specific disease/condition defined population
**Gray 2020**	Asymptomatic carotid stenosis is associated with mobility and cognitive dysfunction and heightens falls in older adults	Population below minimum age criteria
**Gray-Miceli 2014**	Clinical risk factors for orthostatic hypotension among elderly fallers in a continuing care retirement community	Brief report, narrative review, editorial, conference abstract, or not a primary research paper
**Gray-Miceli 2020**	Clinical Risk Factors for Orthostatic Hypotension: Results Among Elderly Fallers in Long-Term Care	Specific disease/condition defined population
**Gribbin 2010**	Risk of falls associated with antihypertensive medication: Population-based case-control study	No assessment of associations between CV disorders and falls, or information on prevalence
**Gribbin 2011**	Risk of falls associated with antihypertensive medication: self-controlled case series	No assessment of associations between CV disorders and falls, or information on prevalence
**Grossman 2016**	Dysrhythmia and occult syncope as an explanation for falls in older patients	Brief report, narrative review, editorial, conference abstract, or not a primary research paper
**Guanlao 2014**	Modifiable risks of fall in geriatric patients with compromised skeletal health	Brief report, narrative review, editorial, conference abstract, or not a primary research paper
**Gul Khattak 2021**	Fall prevalence and associated risk factors in geriatric population	No assessment of associations between CV disorders and falls, or information on prevalence
**Gumprecht 2019**	In older patients with AF, antiarrhythmic drugs were linked to fall-related injuries and syncope vs rate-lowering drugs	Brief report, narrative review, editorial, conference abstract, or not a primary research paper
**Gurumurthy 2014**	The risk of geriatric conditions in patient with diabetes mellitus and hypertension	No assessment of associations between CV disorders and falls, or information on prevalence
**Habtemariam 2015**	Re-examining the effect of antihypertensive medications ons falls in old age	Brief report, narrative review, editorial, conference abstract, or not a primary research paper
**Harfouche 2017**	Syncope workup: Greater yield in select trauma population	Population below minimum age criteria
**Harianto 2017**	In-hospital Falls in Older Patients: The Risk Factors and The Role of Hyponatraemia	Unable to locate full text
**Hartog 2017**	The clinical relevance of orthostatic hypotension in elderly patients	Brief report, narrative review, editorial, conference abstract, or not a primary research paper
**Hauser 2013**	Impact of droxidopa treatment on falls and fall related injuries in patients with Parkinson's disease and symptomatic neurogenic orthostatic hypotension (study 306)	Brief report, narrative review, editorial, conference abstract, or not a primary research paper

First author and year	Article title	Reason for exclusion
**Hauser 2014**	Droxidopa in patients with neurogenic orthostatic hypotension associated with Parkinson's Disease (NOH306A)	Population below minimum age criteria
**Hauser 2016**	Droxidopa and Reduced Falls in a Trial of Parkinson Disease Patients With Neurogenic Orthostatic Hypotension	Population below minimum age criteria
**Hawkins 2011**	The burden of falling on the quality of life of adults with Medicare supplement insurance	Unable to locate full text
**Hill 2007**	Falls in the acute hospital setting--impact on resource utilisation	Population below minimum age criteria
**Hohtari-Kivimaki 2021**	Orthostatic Hypotension is a Risk Factor for Falls Among Older Adults: 3-Year Follow-Up	Specific disease/condition defined population
**Homoud 2002**	Cardiac pacing reduced nonaccidental falls in older adults with cardioinhibitory carotid sinus hypersensitivity	Brief report, narrative review, editorial, conference abstract, or not a primary research paper
**Hyne 2018**	Are we over treating the blood pressure and making our elderly patients fall?	Brief report, narrative review, editorial, conference abstract, or not a primary research paper
**Isacu 2019**	The effects of predisposing and precipitating factors on gravity of falls	Brief report, narrative review, editorial, conference abstract, or not a primary research paper
**Izzati 2017**	Potential risk factors among individuals with recurrent and injurious falls recruited to the Malaysian falls assessment and intervention trial (MyFAIT)	Brief report, narrative review, editorial, conference abstract, or not a primary research paper
**Jansen 2014**	Electrocardiographic abnormalities in patients admitted for hip fracture	No assessment of associations between CV disorders and falls, or information on prevalence
**Jansen 2015**	Effectiveness of a Cardiovascular Evaluation and Intervention in Older Fallers: A Pilot Study	Brief report, narrative review, editorial, conference abstract, or not a primary research paper
**Jödicke 2021**	Risk of falls following the initiation of antihypertensives in the elderly: A self-controlled case series in the UK	Brief report, narrative review, editorial, conference abstract, or not a primary research paper
**Jödicke 2022**	Risk of falls and fractures following the initiation of anti-hypertensives in the elderly: self-controlled case series analyses from the uk	Brief report, narrative review, editorial, conference abstract, or not a primary research paper
**Jonas 2018**	Ambulatory blood-pressure monitoring, antihypertensive therapy and the risk of fall injuries in elderly hypertensive patients	Specific disease/condition defined population
**Jovanovich 2021**	FGF23, Frailty, and Falls in SPRINT	No assessment of associations between CV disorders and falls, or information on prevalence
**Juraschek 2016**	Orthostatic hypotension and risk of falls in the atherosclerosis risk in communities (ARIC) study	Population below minimum age criteria
**Juraschek 2019**	Association between high sensitivity troponin T and N-terminal pro B-type natriuretic peptide and fall risk in older adult participants of the atherosclerosis risk in communities study (ARIC)	Brief report, narrative review, editorial, conference abstract, or not a primary research paper
**Juraschek 2019**	Association of Orthostatic Hypotension Timing With Clinical Events in Adults With Diabetes and Hypertension: Results From the ACCORD Trial	Population below minimum age criteria

First author and year	Article title	Reason for exclusion
**Juraschek 2019**	Effects of Antihypertensive Class on Falls, Syncope, and Orthostatic Hypotension in Older Adults: The ALLHAT Trial	Specific disease/condition defined population
**Juraschek 2021**	Effects of Antihypertensive De-prescribing Strategies on Blood Pressure, Adverse Events, and Orthostatic Symptoms in Older Adults: Results from TONE	No assessment of associations between CV disorders and falls, or information on prevalence
**Kenny 1999**	Brief Report - SAFE PACE 2 Syncope And Falls in the Elderly Pacing And Carotid Sinus Evaluation: A Randomized Control Trial of Cardiac Pacing in Older Patients With Falls and Carotid Sinus Hypersensitivity	Brief report, narrative review, editorial, conference abstract, or not a primary research paper
**Kenny 1999**	SAFE PACE 2: Syncope and Falls in the Elderly--Pacing and Carotid Sinus Evaluation: a randomized controlled trial of cardiac pacing in older patients with falls and carotid sinus hypersensitivity	Brief report, narrative review, editorial, conference abstract, or not a primary research paper
**Keskin 2021**	Orthostatic hypotension and age-related sarcopenia	No assessment of associations between CV disorders and falls, or information on prevalence
**Kiliç 2018**	Incidence of coronary artery disease and associated factors in elderly patients	Unable to locate full text
**Kirshen 1984**	Postural sway and cardiovascular parameters associated with falls in the elderly	Unable to locate full text
**Knight 2022**	Associations between cardiovascular signal entropy and future falls, syncope, and fear of falling	Brief report, narrative review, editorial, conference abstract, or not a primary research paper
**Kochersberger 2014**	Hypertension and falls in the first month of nursing home care	Brief report, narrative review, editorial, conference abstract, or not a primary research paper
**Kondo 2022**	Standing Blood Pressure And Risk Of Falls, Syncope, Cardiovascular Disease, And Mortality	Brief report, narrative review, editorial, conference abstract, or not a primary research paper
**Konova 2019**	The use of fall-risk-increasing drugs in comorbid patients with polypharmacy	Brief report, narrative review, editorial, conference abstract, or not a primary research paper
**Koujiya 2018**	The association of blood pressure level with clinical events in old patients with home medical care	Brief report, narrative review, editorial, conference abstract, or not a primary research paper
**Koujiya 2021**	Associations of blood pressure levels with clinical events in older patients receiving home medical care	No assessment of associations between CV disorders and falls, or information on prevalence
**Krasniqi 2012**	Falls and fractures in the elderly with sinus node disease: the impact of pacemaker implantation	Not a randomised controlled trial
**Krishnaswami 2018**	Individual and Joint Effects of Pulse Pressure and Blood Pressure Treatment Intensity on Serious Adverse Events in the SPRINT Trial	No assessment of associations between CV disorders and falls, or information on prevalence
**Krishnaswami 2019**	Restricted mean survival time analysis to assess efficacy and safety of intensive vs standard blood pressure treatment goals as a function of age: Insights from the SPRINT trial	Brief report, narrative review, editorial, conference abstract, or not a primary research paper

First author and year	Article title	Reason for exclusion
**Kruse 1996**	Falls in community-dwelling older persons	Brief report, narrative review, editorial, conference abstract, or not a primary research paper
**Kuhne 2014**	The effect of pacemaker implantation on falls and fall characteristics in patients with sinus node dysfunction	Brief report, narrative review, editorial, conference abstract, or not a primary research paper
**Kunkel 2017**	A cross-sectional observational study comparing foot and ankle characteristics in people with stroke and healthy controls	No assessment of associations between CV disorders and falls, or information on prevalence
**Kuno 2021**	Falls and associated risk factors among elderly survivors of the Great East Japan Earthquake: RIAS Study	Unable to locate full text
**Kwan 2014**	Assessment and management of falls in older people	Brief report, narrative review, editorial, conference abstract, or not a primary research paper
**Lambert 2016**	Prospective collection of the falls in geriatrics at the Hôpitaux Universitaires of Strasbourg	Brief report, narrative review, editorial, conference abstract, or not a primary research paper
**Larina 2017**	Risk of Cardiovascular Complications and Geriatric Syndromes Among Elderly Women	Unable to locate full text
**Lau 2001**	Risk factors for hip fracture in Asian men and women: The Asian Osteoporosis Study	No assessment of associations between CV disorders and falls, or information on prevalence
**Lazkani 2015**	Predicting falls in elderly patients with chronic pain and other chronic conditions	Specific disease/condition defined population
**Lee 2017**	Risk assessment and falls prevention in the older adult: Asian experience with the Falls Risk for Older People in the Community tool	No assessment of associations between CV disorders and falls, or information on prevalence
**LeJeune 2021**	Prevalence of masked orthostatic hypotension measured by home blood pressure monitoring in elderly hypertensive subjects: Results of the hypauttens study	Unable to locate full text
**Lembeck 2019**	Description of patients with falls or dizziness in a rural and socioeconomic deprived area in Denmark	Brief report, narrative review, editorial, conference abstract, or not a primary research paper
**Li 2021**	The modified Healthy Aging Index is associated with mobility limitations and falls in a community-based sample of oldest old	No assessment of associations between CV disorders and falls, or information on prevalence
**Lin 2020**	Predicting fall risks in older adults with atrial fibrillation	Brief report, narrative review, editorial, conference abstract, or not a primary research paper
**Lipsitz 2015**	Reexamining the Effect of Antihypertensive Medications on Falls in Old Age	Specific disease/condition defined population
**Liu 2021**	Prevalence and the factors associated with malnutrition risk in elderly Chinese inpatients	No assessment of associations between CV disorders and falls, or information on prevalence
**Ma 2021**	Cross-sectional study examining the status of intrinsic capacity decline in community-dwelling older adults in China: Prevalence, associated factors and implications for clinical care	No assessment of associations between CV disorders and falls, or information on prevalence
**Makam 2020**	Rates of falls and risk factors associated with falls in older adults with atrial fibrillation: findings from the prospective sage-af study	Brief report, narrative review, editorial, conference abstract, or not a primary research paper
**Mancini 2005**	Epidemiology of falls among the elderly	Unable to locate full text

First author and year	Article title	Reason for exclusion
**Manemann 2018**	Fall Risk and Outcomes Among Patients Hospitalized With Cardiovascular Disease in the Community	Population below minimum age criteria
**Marcum 2015**	Low blood pressure cut points for fall injuries in community-dwelling elderly: The health, aging and body composition study	Brief report, narrative review, editorial, conference abstract, or not a primary research paper
**Marcum 2015**	Antihypertensive Use and Recurrent Falls in Community-Dwelling Older Adults: Findings From the Health ABC Study	No assessment of associations between CV disorders and falls, or information on prevalence
**Margolis 2014**	Intensive blood pressure control, falls, and fractures in patients with type 2 diabetes: the ACCORD trial	Specific disease/condition defined population
**Marion 2019**	Demographics and characteristics of patients with recurrent falls attending tertiary hospital outpatients department	Brief report, narrative review, editorial, conference abstract, or not a primary research paper
**Martínez 2014**	Experience with the use of an implantable loop recorder in a series of older people with falls and suspected arrhythmic syncopes	Unable to locate full text
**Mayo 1989**	Predicting falls among patients in a rehabilitation hospital	Population below minimum age criteria
**Michael 2016**	Comorbidities and polypharmacy in hip fracture patients	Brief report, narrative review, editorial, conference abstract, or not a primary research paper
**Min 2020**	CV, death, fall injury, & syncope outcomes of high-intensity bp medications	Brief report, narrative review, editorial, conference abstract, or not a primary research paper
**Mizukami 2013**	Falls are associated with stroke, arthritis and multiple medications among community-dwelling elderly persons in Japan	No assessment of associations between CV disorders and falls, or information on prevalence
**Modirian 2014**	Falls and their associated factors among Iranian elderly population	Brief report, narrative review, editorial, conference abstract, or not a primary research paper
**Moe 2018**	Falls' and fallers' profiles in older patients presenting to an Accident and Emergency (A&E) department	Brief report, narrative review, editorial, conference abstract, or not a primary research paper
**Mol 2020**	Blood pressure drop rate after standing up is associated with frailty and number of falls in geriatric outpatients	No assessment of associations between CV disorders and falls, or information on prevalence
**Mol 2021**	Orthostatic blood pressure recovery associates with physical performance, frailty and number of falls in geriatric outpatients	No assessment of associations between CV disorders and falls, or information on prevalence
**Mol 2021**	Orthostatic Blood Pressure Recovery Measured Using a Sphygmomanometer Is Not Associated with Physical Performance or Number of Falls in Geriatric Outpatients	No assessment of associations between CV disorders and falls, or information on prevalence
**Moon 2009**	Comparison of factors of falls between ethnic Korean and Japanese older residents in an urban community in Japan	Unable to locate full text
**Morrison 2011**	Changes in falls risk factors for geriatric diagnostic groups across inpatient, outpatient and domiciliary rehabilitation settings	Population below minimum age criteria

First author and year	Article title	Reason for exclusion
**Morteza Bagi 2017**	Demographics of Fall-Related trauma among the Elderly Presenting to Emergency Department; a Cross-Sectional Study	No assessment of associations between CV disorders and falls, or information on prevalence
**Muangpaisan 2015**	Causes and course of falls resulting in hip fracture among elderly Thai patients	Specific disease/condition defined population
**Muller 2018**	Emergency medicine in the extreme geriatric era: A retrospective analysis of patients aged in their mid 90s and older in the emergency department	No assessment of associations between CV disorders and falls, or information on prevalence
**Mulloy 2019**	Multiple antihypertensive therapy and risk of falls in geriatric patients	Brief report, narrative review, editorial, conference abstract, or not a primary research paper
**Muscari 2021**	The association of proBNPage with manifestations of age-related cardiovascular, physical, and psychological impairment in community-dwelling older adults	No assessment of associations between CV disorders and falls, or information on prevalence
**Najafpour 2019**	Risk Factors for Falls in Hospital In-Patients: A Prospective Nested Case Control Study	Population below minimum age criteria
**Norouzi 2020**	Prevalence of falls in older people and associated factors in Qaemshahr, Iran 2019	Unable to locate full text
**Nugraha 2019**	Multimorbidity increases the risk of falling among Indonesian elderly living in community dwelling and elderly home: A cross sectional study	No assessment of associations between CV disorders and falls, or information on prevalence
**Oliveira Guerra 2003**	Intrinsical risk factors of fall on elderly institutionalized patients	Unable to locate full text
**Omer 2018**	Inpatient falls in older adults: a cohort study of antihypertensive prescribing pre- and post-fall	No assessment of associations between CV disorders and falls, or information on prevalence
**Oztorun 2021**	The relationship between sarcopenia and central hemodynamics in older adults with falls: a cross-sectional study	Unable to locate full text
**Pajewski 2016**	Characterizing Frailty Status in the Systolic Blood Pressure Intervention Trial	No assessment of associations between CV disorders and falls, or information on prevalence
**Pajewski 2020**	Intensive vs Standard Blood Pressure Control in Adults 80 Years or Older: A Secondary Analysis of the Systolic Blood Pressure Intervention Trial	No assessment of associations between CV disorders and falls, or information on prevalence
**Pasqualetti 2014**	The Pisa Emergency Department Elderly assessment (PADEA): Prospective analysis of falls	Brief report, narrative review, editorial, conference abstract, or not a primary research paper
**Pasqualetti 2017**	Clinical differences among the elderly admitted to the emergency department for accidental or unexplained falls and syncope	No assessment of associations between CV disorders and falls, or information on prevalence
**Patel 2018**	Fall risk during opposing stance perturbations among healthy adults and chronic stroke survivors	Population below minimum age criteria
**Peeters 2017**	Associations of Guideline Recommended Medications for Acute Coronary Syndromes With Fall-Related Hospitalizations and Cardiovascular Events in Older Women With Ischemic Heart Disease	Specific disease/condition defined population
**Pengpid 2021**	Geriatric Conditions and Functional Disability among a National Community-Dwelling Sample of Older Adults in India in 2017-2018	No assessment of associations between CV disorders and falls, or information on prevalence
**Pengpid 2022**	National data on the prevalence and correlates of recurrent falls among community-dwelling older adults in India in 2017-2018	Unable to locate full text
**Perego 2021**	The Dilemma of Falls in Older Persons: Never Forget to Investigate the Syncope	Specific disease/condition defined population

First author and year	Article title	Reason for exclusion
**Perracini 2011**	Subclinical bradypedia in a representative population of older persons and its association with clinical and physical function measures	Brief report, narrative review, editorial, conference abstract, or not a primary research paper
**Petriceks 2022**	Timing Of Orthostatic Hypotension And Its Relationship With Falls In Older Adults	Brief report, narrative review, editorial, conference abstract, or not a primary research paper
**Pi 2016**	Risk Factors for In-Hospital Complications of Fall-Related Fractures among Older Chinese: A Retrospective Study	Specific disease/condition defined population
**Polidori 2014**	Evaluation of fall risks in elderly patients	Brief report, narrative review, editorial, conference abstract, or not a primary research paper
**PortoGauterio 2015**	Risk Factors for new accidental falls in elderly patients at traumatology ambulatory center	Specific disease/condition defined population
**Powell 2021**	Self-Report Hearing and Injury or Falls in Older Adults from the National Health and Information Survey	No assessment of associations between CV disorders and falls, or information on prevalence
**Prabhakaran 2020**	Falling Again? Falls in Geriatric Adults—Risk Factors and Outcomes Associated With Recidivism	No assessment of associations between CV disorders and falls, or information on prevalence
**Prada 2017**	Risk factors and complications of arterial hypotension in older people	Unable to locate full text
**Prada 2018**	Patterns and complications of arterial hypotension in older people	Brief report, narrative review, editorial, conference abstract, or not a primary research paper
**Pullen 1999**	Falls in hospital: Prospective study of time, place and circumstances	Unable to locate full text
**Rao 2018**	Clinical Outcomes and History of Fall in Patients with Atrial Fibrillation Treated with Oral Anticoagulation: Insights From the ARISTOTLE Trial	Specific disease/condition defined population
**Rashedi 2019**	Risk factors for fall in elderly with diabetes mellitus type 2	Specific disease/condition defined population
**Rausch 2021**	Geriatric Syndromes and Incident Chronic Health Conditions Among 9094 Older Community-Dwellers: Findings from the Lifelines Cohort Study	No assessment of associations between CV disorders and falls, or information on prevalence
**Rausch 2022**	Geriatric Syndromes and Incident Chronic Health Conditions Among 9094 Older Community-Dwellers: Findings from the Lifelines Cohort Study	No assessment of associations between CV disorders and falls, or information on prevalence
**Ridge 2017**	Frail, falling, alone-30-day readmissions not just about hospital care. a prospective study in a large Irish teaching hospital	Brief report, narrative review, editorial, conference abstract, or not a primary research paper
**Rivasi 2021**	Effects of benzodiazepines on orthostatic blood pressure in older people	Brief report, narrative review, editorial, conference abstract, or not a primary research paper
**Roosendaal 2016**	Postprandial hypotension should always be evaluated in elderly patients with unexplained falls and syncope	Brief report, narrative review, editorial, conference abstract, or not a primary research paper
**Roosendaal 2018**	Different patterns of orthostatic hypotension in older patients with unexplained falls or syncope: orthostatic hypotension patterns in older people	No assessment of associations between CV disorders and falls, or information on prevalence
**Ross 2012**	Falls in the Inpatient Rehabilitation Facility	Brief report, narrative review, editorial, conference abstract, or not a primary research paper
**Ruwald 2013**	Comparison of incidence, predictors, and the impact of co-morbidity and polypharmacy on the risk of recurrent syncope in patients <85 versus >=85 years of age	No assessment of associations between CV disorders and falls, or information on prevalence
**Sabbaghan 2014**	Orthostatic hypotension, frailty and falling risk in elderly care home residents	Brief report, narrative review, editorial, conference abstract, or not a primary research paper

First author and year	Article title	Reason for exclusion
**Safarpour 2018**	Predictors of incidence of fall in elderly women; A six-month cohort study	No assessment of associations between CV disorders and falls, or information on prevalence
**Sagawa 2017**	Metabolic syndrome and risk of incident fall injury in community-dwelling older adults: The health, aging, and body composition study	Unable to locate full text
**Salamon 2012**	Identification of patients at risk for falls in an inpatient rehabilitation program	Population below minimum age criteria
**Salvà 2012**	Falls and risk factors for falls in community-dwelling adults with dementia (NutriAlz Trial)	Specific disease/condition defined population
**Sarigül 2014**	Assessment of balance and falls in geriatric patients and determining risk factors	Unable to locate full text
**Schumacher 2021**	Accelerometer-Measured Daily Steps, Physical Function, and Subsequent Fall Risk in Older Women: The Objective Physical Activity and Cardiovascular Disease in Older Women Study	No assessment of associations between CV disorders and falls, or information on prevalence
**Shah 2021**	Geriatric Syndromes and Atrial Fibrillation: Prevalence and Association with Anticoagulant Use in a National Cohort of Older Americans	Specific disease/condition defined population
**Shahimi 2022**	Psychological status and physical performance are independently associated with autonomic function	No assessment of associations between CV disorders and falls, or information on prevalence
**Shapiro 2018**	Impact of intensive versus standard blood pressure management by tertiles of blood pressure in SPRINT (Systolic Blood Pressure Intervention Trial)	Same sample as in another study
**Shaw 2019**	Relationships between orthostatic hypotension, frailty, falling and mortality in elderly care home residents	No assessment of associations between CV disorders and falls, or information on prevalence
**Sheldon 2022**	Randomized Pragmatic Trial of Pacemaker Versus Implantable Cardiac Monitor in Syncope and Bifascicular Block	No assessment of associations between CV disorders and falls, or information on prevalence
**Sheppard 2020**	Effect of Antihypertensive Medication Reduction vs Usual Care on Short-term Blood Pressure Control in Patients with Hypertension Aged 80 Years and Older: The OPTIMISE Randomized Clinical Trial	Not a randomised controlled trial
**Shimbo 2015**	Risk of serious fall injuries after initiation of antihypertensive medications in older adults	Brief report, narrative review, editorial, conference abstract, or not a primary research paper
**Shimbo 2016**	Short-Term Risk of Serious Fall Injuries in Older Adults Initiating and Intensifying Treatment With Antihypertensive Medication	No assessment of associations between CV disorders and falls, or information on prevalence
**Shimbo 2016**	Short-term risk of serious fall injuries in older adults initiating and intensifying treatment with antihypertensive medication	No assessment of associations between CV disorders and falls, or information on prevalence
**Shuto 2010**	Medication use as a risk factor for inpatient falls in an acute care hospital: A case-crossover study	Population below minimum age criteria
**Sim 2018**	Low Systolic Blood Pressure From Treatment and Association With Serious Falls/Syncope	Population below minimum age criteria
**Sink 2016**	Impact of intensive blood pressure lowering on ER visits and serious adverse events for symptomatic hypotension, syncope, and injurious falls; Results from the SPRINT study	Brief report, narrative review, editorial, conference abstract, or not a primary research paper
**Sink 2018**	Syncope, Hypotension, and Falls in the Treatment of Hypertension: Results from the Randomized Clinical Systolic Blood Pressure Intervention Trial	No assessment of associations between CV disorders and falls, or information on prevalence
**Song 2018**	Antihypertensive Drug Deintensification and Recurrent Falls in Long-Term Care	No assessment of associations between CV disorders and falls, or information on prevalence
**Soultan 2021**	Blood pressure variability is associated with falls in long term care	Brief report, narrative review, editorial, conference abstract, or not a primary research paper
**Spiegel 2019**	Associated factors and outcomes of falls in acute elderly inpatients	Brief report, narrative review, editorial, conference abstract, or not a primary research paper

First author and year	Article title	Reason for exclusion
**Still 2018**	Clinical Outcomes by Race and Ethnicity in the Systolic Blood Pressure Intervention Trial (SPRINT): A Randomized Clinical Trial	Same sample as in another study
**Surmeli 2018**	Is reverse dipping pattern a risk factor for falls in hypertensive older adults?	Brief report, narrative review, editorial, conference abstract, or not a primary research paper
**Szulc 2015**	High risk of fall, poor physical function and low grip strength in men with fracture: The strambo study	Brief report, narrative review, editorial, conference abstract, or not a primary research paper
**Takahashi 2021**	Changes in trends of diseases requiring long-term care in an aging community	Unable to locate full text
**Tan 2019**	Arterial stiffness and falls in older people: Cross-sectional data from the Malaysian elders longitudinal research study (MELOR)	Brief report, narrative review, editorial, conference abstract, or not a primary research paper
**Tas 2007**	Incidence and risk factors of disability in the elderly: the Rotterdam Study	No assessment of associations between CV disorders and falls, or information on prevalence
**Tesi 2017**	Prognostic impact of syncopal-like falls" in elderly patients with dementia: Preliminary results of the follow-up of the "syncope and Dementia" Study"	Brief report, narrative review, editorial, conference abstract, or not a primary research paper
**Testa 2018**	Hypotensive Drugs and Syncope Due to Orthostatic Hypotension in Older Adults with Dementia (Syncope and Dementia Study)	Specific disease/condition defined population
**Thumé 2018**	Health status of the elderly in bagé, state of rio grande do sul, brazil	Brief report, narrative review, editorial, conference abstract, or not a primary research paper
**Tinetti 2014**	Antihypertensive medications and serious fall injuries in a nationally representative sample of older adults	Specific disease/condition defined population
**Tommasini 2008**	Risk factors of falls in elderly population in acute care hospitals and nursing homes in North Italy: A retrospective study	Specific disease/condition defined population
**Trenkwalder 2008**	Combination of amlodipine 10 mg and valsartan 160 mg lowers blood pressure in patients with hypertension not controlled by an ACE inhibitor/CCB combination	Population below minimum age criteria
**Turusheva 2019**	Association between heart rate response during orthostatic test and falls in polymorbid patients aged 60 years and older	Unable to locate full text
**Turusheva 2021**	Silent atrial fibrillation as a risk factor for falls in the elderly.	Unable to locate full text
**Un 2021**	Chronic diseases and sleep duration in association with falls of different severity among the Chinese elderly	Unable to locate full text
**Ungar 2016**	Etiology of Syncope and Unexplained Falls in Elderly Adults with Dementia: Syncope and Dementia (SYD) Study	Specific disease/condition defined population
**Valentina Ostapenko 2017**	Prevalence geriatric syndromes among outpatients with and without chronic heart failure	Brief report, narrative review, editorial, conference abstract, or not a primary research paper
**vandeLoo 2022**	Development of the ADFICE_IT Models for Predicting Falls and Recurrent Falls in Community-Dwelling Older Adults: Pooled Analyses of European Cohorts With Special Attention to Medication	No assessment of associations between CV disorders and falls, or information on prevalence
**Vetrano 2016**	Chronic diseases and geriatric syndromes: The different weight of comorbidity	No assessment of associations between CV disorders and falls, or information on prevalence
**Vlahov 1990**	Epidemiology of falls among patients in a rehabilitation hospital	Unable to locate full text

First author and year	Article title	Reason for exclusion
**Vorilhon 2015**	Optimised management of over-80-year-old heart failure patient improves outcomes: HF80 Pilot randomised study	Brief report, narrative review, editorial, conference abstract, or not a primary research paper
**Vorilhon 2016**	Optimized management of heart failure patients aged 80 years or more improves outcomes versus usual care: The HF80 randomized trial	Specific disease/condition defined population
**Vu 2019**	Effects of chronic comorbidities on the health-related quality of life among older patients after falls in vietnamese hospitals	No assessment of associations between CV disorders and falls, or information on prevalence
**Wang 2014**	Geriatric conditions as predictors of increased number of hospital admissions and hospital bed days over one year: Findings of a nationwide cohort of older adults from Taiwan	No assessment of associations between CV disorders and falls, or information on prevalence
**White 2019**	Orthostatic vital signs do not predict 30day serious outcomes in older emergency department patients with syncope: A multicenter observational study	No assessment of associations between CV disorders and falls, or information on prevalence
**Williamson 2016**	Intensive vs standard blood pressure control and cardiovascular disease outcomes in adults aged â‰¥75 years a randomized clinical trial	Same sample as in another study
**Wilson 2019**	Orthostatic hypotension in the outpatient setting after a fall	Brief report, narrative review, editorial, conference abstract, or not a primary research paper
**Winstead 2021**	Fall-Related Facial Trauma: A Retrospective Review of Fracture Patterns and Medical Comorbidity	Population below minimum age criteria
**Wu 2014**	Study on the relationship between frailty index of elderly patients with coronary heart disease and health related adverse event-a retrospective study	Brief report, narrative review, editorial, conference abstract, or not a primary research paper
**Xu 2022**	A five-year prospective evaluation of anticholinergic cognitive burden and falls in the Malaysian elders longitudinal research (MELoR) study	No assessment of associations between CV disorders and falls, or information on prevalence
**Yaghoubi 2022**	Fall incidence in hospitalized patients and prediction of its risk factors using a weighted Poisson model	Population below minimum age criteria
**Yasumura 1994**	[Risk factors for falls among the elderly living in a Japanese rural community]	Brief report, narrative review, editorial, conference abstract, or not a primary research paper
**Yip 2008**	The Hong Kong diastolic heart failure study: A randomised controlled trial of diuretics, irbesartan and ramipril on quality of life, exercise capacity, left ventricular global and regional function in heart failure with a normal ejection fraction	Population below minimum age criteria
**Yokomoto-Umakoshi 2017**	Association between the risk of falls and osteoporotic fractures in patients with type 2 diabetes mellitus	Specific disease/condition defined population
**Zarudsky 2018**	Falling risk factors in patients with arterial hypertension 55+ years old	Brief report, narrative review, editorial, conference abstract, or not a primary research paper
**Zhang 2016**	Study on incidence and risk factors of fall in the elderly in a rural community in Beijing	Unable to locate full text
**Zhang 2021**	Relationship Between Chronic Conditions and Balance Disorders in Outpatients with Dizziness: A Hospital-Based Cross-Sectional Study	No assessment of associations between CV disorders and falls, or information on prevalence
**Zullo 2019**	Patient-Important Adverse Events of β-blockers in Frail Older Adults after Acute Myocardial Infarction	Specific disease/condition defined population

Appendix 3. Quality assessments

Quality assessment for observational studies (NOS)

Study	Type of study	Quality assessment score (Overall)	Quality assessment score(Selection)	Quality assessment score (Comparability)	Quality assessment score (Exposure/Outcome)
Abbs 2020	Cohort	7 (high quality)	* * * *	* *	*
Aburub 2021	Cross-sectional	7 (high quality)	* * *	* *	* *
Akande-Sholabi 2021	Cross-sectional	6 (intermediate quality)	* * * *	-	* *
Alamgir 2015	Cross-sectional	5 (intermediate quality)	* * *	* *	-
Almegbel 2018	Cross-sectional	7 (high quality)	* * * *	*	* *
Anpalahan 2012	Case-control	6 (intermediate quality)	* * *	*	* *
Aronow, 1997	Cohort	5 (intermediate quality)	* *	*	* *
Arseven 2008	Cohort	10 (high quality)	* * * *	* *	* * * *
Assantachai 2003	Cross-sectional	6 (intermediate quality)	* * *	* *	*
Aydin 2017	Cross-sectional	7 (high quality)	* * * *	* *	*
Banu 2018	Case-control	8 (high quality)	* * * *	* *	* *
Bergland 2003	Cohort	8 (high quality)	* *	* *	* * * *
Bhangu 2017	Cohort	8 (high quality)	* * * *	* *	* *
Brassington 2000	Cross-sectional	6 (intermediate quality)	* * *	* *	*
Bumin 2002	Cross-sectional	5 (intermediate quality)	* * *	*	*
Callisaya 2014	Cohort	10 (high quality)	* * * *	* *	* * * *
Campbell 1981	Cross-sectional	4 (intermediate quality)	* * *	-	
Campbell 1989	Cohort	9 (high quality)	* * * *	* *	* * *
Chan 1997	Cross-sectional	6 (intermediate quality)	* * * *	*	*
Chang 2010	Cross-sectional	7 (high quality)	* * * *	* *	*
Chang 2011	Case-control	7 (high quality)	* * * *	* *	*
Chang 2015	Cross-sectional	6 (intermediate quality)	* * * *	-	* *
Chen 2008	Cross-sectional	8 (high quality)	* * * *	* *	* *
Chen 2010	Case-control	7 (high quality)	* * * *	* *	*
Choi 2014	Cross-sectional	7 (high quality)	* * *	* *	* *
Chu 2007	Cohort	7 (high quality)	* * * *	* *	*
Claffey 2022	Cross-sectional	7 (high quality)	* * * *	* *	*
Coutaz 2012	Cohort	7 (high quality)	* * * *	* *	*
Coutinho 2008	Case-control	5 (intermediate quality)	* *	* *	*
Dahodwala 2017	Cohort	7 (high quality)	* * *	* *	* *
Damian 2013	Cross-sectional	4 (intermediate quality)	* *	* *	-
Davies 2001	Case-control	6 (intermediate quality)	* *	-	* * * *
Davison 2005	Case-control	7 (high quality)	* * * *	*	* *

Study	Type of study	Quality assessment score (Overall)	Quality assessment score(Selection)	Quality assessment score (Comparability)	Quality assessment score (Exposure/Outcome)
Del Brutto 2019	Cross-sectional	8 (high quality)	* * * *	* *	* *
Dokulzar 2020	Cross-sectional	7 (high quality)	* * *	* *	* *
Dolinis 1997	Cross-sectional	8 (high quality)	* * * *	* *	* *
Donoghue 2021	Cohort	8 (high quality)	* * * *	* *	* *
Dos Reis 2015	Cohort	8 (high quality)	* * * *	* *	* *
Downton 1991	Cross-sectional	6 (intermediate quality)	* * * *	*	*
Ek 2019	Cohort	8 (high quality)	* * * *	* *	* *
Ensrud 1992	Cross-sectional	7 (high quality)	* * * *	* *	*
Finucane 2017	Cohort	8 (high quality)	* * * *	* *	* *
Frankenthal 2021	Cohort	7 (high quality)	* * *	* *	* *
Frels 2002	Case-control	7 (high quality)	* * * *	* *	*
Freud 2015	Cross-sectional	8 (high quality)	* * * *	* *	* *
Friedman 2002	Cohort	8 (high quality)	* * *	* *	* * *
Gamage 2019	Cross-sectional	8 (high quality)	* * * *	* *	* *
Gangavati 2011	Cohort	9 (high quality)	* * * *	* *	* * *
Gebre 2021	Cohort	8 (high quality)	* * * *	* *	* *
Gebre 2022	Cohort	8 (high quality)	* * *	* *	* * *
Geng 2017	Cross-sectional	4 (intermediate quality)	* *	*	*
George 2014	Cross-sectional	6 (intermediate quality)	* * *	* *	*
Goh 2017	Cohort	8 (high quality)	* * * *	* *	* *
Graafmans 1996	Cohort	9 (high quality)	* * * *	* *	* * *
Granek 1987	Case-control	4 (intermediate quality)	* * *	-	*
Grundstrom 2012	Cross-sectional	7 (intermediate quality)	* * * *	* *	*
Ha 2021	Cross-sectional	8 (high quality)	* * * *	* *	* *
Ham 2017	Cohort	9 (high quality)	* * * *	* *	* * *
Hanlon 2002	Cohort	7 (high quality)	* * * *	* *	*
Hartog 2015	Cohort	7 (high quality)	* * * *	* *	*
Hartog 2017	Cohort	8 (high quality)	* * * *	* *	* *
Heckenbach 2014	Cross-sectional	6 (intermediate quality)	* * *	* *	*
Heitterachi 2002	Cohort	9 (high quality)	* * * *	* *	* * *
Herndon 1997	Case-control	5 (intermediate quality)	* *	* *	*
Himes 2012	Cohort	7 (high quality)	* * *	* *	* *
Ho 1996	Cross-sectional	8 (high quality)	* * * *	* *	* *
Homer 2017	Cohort	6 (intermediate quality)	* *	* *	* *
Hosseini 2020	Cross-sectional	8 (high quality)	* * * *	* *	* *
Hung 2013	Cross-sectional	5 (intermediate quality)	* *	* *	*
Hung 2017	Cross-sectional	6 (intermediate quality)	* * * *	-	* *
Hussain 2022	Cohort	8 (high quality)	* * * *	* *	* *

Study	Type of study	Quality assessment score (Overall)	Quality assessment score(Selection)	Quality assessment score (Comparability)	Quality assessment score (Exposure/Outcome)
Jacob 2022	Cohort	7 (high quality)	* * * *	* *	*
Jansen 2015	Cross-sectional	6 (intermediate quality)	* * *	* *	*
Jansen 2015	Cross-sectional	6 (intermediate quality)	* * *	* *	*
Jia 2019	Cohort	6 (intermediate quality)	* * *	* *	*
Jitapunkul 1998	Cross-sectional	6 (intermediate quality)	* * *	* *	*
Jodaitis 2015	Case-control	5 (intermediate quality)	*	* *	* *
Jonsson 1990	Cohort	7 (high quality)	* * *	* *	* *
Jorgensen 2015	Case-control	6 (intermediate quality)	* * *	* *	*
Jurascheck 2019	Cohort	8 (high quality)	* * * *	* *	* *
Juraschek 2022	Cohort	9 (high quality)	* * * *	* *	* * *
Just 2021	Cohort	8 (high quality)	* * *	* *	* * *
Kallin 2004	Cohort	6 (intermediate quality)	* * *	*	* *
Kamali 2022	Cross-sectional	7 (high quality)	* * *	* *	* *
Kang 2018	Cohort	7 (high quality)	* * * *	*	* *
Kao 2012	Cross-sectional	6 (intermediate quality)	* * *	* *	*
Kario 2001	Cohort	9 (high quality)	* * * *	* *	* * *
Kelly 2003	Case-control	6 (intermediate quality)	* * *	* *	*
Klein 2013	Cohort	5 (intermediate quality)	* * *	* *	-
Koca 2020	Case-control	8 (high quality)	* * * *	* *	* *
Kocyigit 2020	Case-control	7 (high quality)	* * *	* *	* *
Kocyigit 2021	Cohort	7 (high quality)	* * * *	* *	*
Kojima 2011	Cross-sectional	7 (high quality)	* * *	* *	* *
Ku 2013	Cross-sectional	9 (high quality)	* * * *	* *	* * *
Kumar 2003	Case-control	7 (high quality)	* * * *	*	* *
Lam 2019	Cohort	7 (high quality)	* * * *	* *	*
Lawlor 2003	Cross-sectional	4 (intermediate quality)	* *	*	*
Le Couteur 2003	Cross-sectional	6 (intermediate quality)	* * *	*	* *
Lee 2006	Cross-sectional	5 (intermediate quality)	* *	* *	*
Lee 2009	Cross-sectional	4 (intermediate quality)	* *	*	*
Lee 2020	Cohort	8 (high quality)	* * * *	* *	* *
Lee 2021	Cohort	7 (high quality)	* * * *	* *	*
Liao 2012	Cross-sectional	5 (intermediate quality)	* *	* *	*
Lipsitz 1991	Case-control	8 (high quality)	* * * *	* *	* *
Liu 1995	Cohort	9 (high quality)	* * * *	* *	* * *
Lord 2003	Cohort	7 (high quality)	* * *	* *	* *
Lukaszyk 2018	Cross-sectional	7 (high quality)	* * * *	*	* *
Luukinen 1996	Cohort	9 (high quality)	* * * *	* *	* * *
Mader 1987	Cross-sectional	5 (intermediate quality)	* * *	*	*

Study	Type of study	Quality assessment score (Overall)	Quality assessment score(Selection)	Quality assessment score (Comparability)	Quality assessment score (Exposure/Outcome)
Magnuszewski 2020	Cross-sectional	8 (high quality)	* * * *	* *	* *
Magnuszewski 2022	Cross-sectional	8 (high quality)	* * * *	* *	* *
Margolis 2019	Cohort	8 (high quality)	* * *	* *	* * *
Maurer 2004	Cohort	8 (high quality)	* * * *	* *	* *
Maurer 2005	Cohort	8 (high quality)	* * * *	* *	* *
McDonald 2017	Cohort	9 (high quality)	* * * *	* *	* * *
Menant 2016	Cohort	10 (high quality)	* * * *	* *	* * * *
Mitchell 2013	Cross-sectional	6 (intermediate quality)	* * *	* *	*
Mitchell 2015	Cross-sectional	8 (high quality)	* * * *	* *	* *
Miu 1997	Cross-sectional	7 (high quality)	* * * *	* *	*
Mol 2022	Cross-sectional	7 (high quality)	* * * *	* *	*
Moloney 2021	Cohort	8 (high quality)	* * * *	* *	* *
Murphy 1986	Cohort	7 (high quality)	* * * *	*	* *
O'Neal 2015	Cross-sectional	8 (high quality)	* * * *	* *	* *
Ooi 2000	Cohort	8 (high quality)	* * * *	* *	* *
Ooi 2021	Cohort	7 (high quality)	* * * *	* *	*
Oren 2022	Case-control	7 (high quality)	* * * *	* *	*
Paganini-Hill 2017	Cohort	8 (high quality)	* * * *	* *	*
Paliwal 2017	Cross-sectional	8 (high quality)	* * * *	* *	* *
Pasma 2014	Cross-sectional	6 (intermediate quality)	* * *	* *	*
Press 2016	Cross-sectional	7 (high quality)	* * *	* *	* *
Prudham 1981	Cross-sectional	4 (intermediate quality)	* *	*	*
Puisieux 2000	Case-control	8 (high quality)	* * * *	* *	* *
Rafiq 2014	Cohort	7 (high quality)	* * *	* *	* *
Rivan 2021	Cohort	7 (high quality)	* * * *	* *	*
Rivera-Chavez 2021	Cross-sectional	7 (high quality)	* * * *	* *	*
Roca 2022	Cross-sectional	7 (high quality)	* * * *	* *	*
Romero-Ortuno 2011	Cross-sectional	6 (intermediate quality)	* * *	* *	*
Rosado 1989	Case-control	8 (high quality)	* * * *	* *	* *
Rosendahl 2003	Cohort	5 (intermediate quality)	* * *	*	*
Rutan 1992	Cross-sectional	6 (intermediate quality)	* * *	* *	*
Saedon 2016	Case-control	6 (intermediate quality)	* *	* *	* *
Sagawa 2018	Cohort	8 (high quality)	* * * *	* *	* *
Salgado 1994	Case-control	7 (high quality)	* * *	* *	* *
Salgado 2004	Cohort	6 (intermediate quality)	* * *	* *	*
Sanders 2012	Case-control	5 (intermediate quality)	* *	* *	*
Sasidharan 2022	Cross-sectional	7 (high quality)	* * * *	* *	*
Schell 2021	Cohort	7 (high quality)	* * *	* *	* *

Study	Type of study	Quality assessment score (Overall)	Quality assessment score(Selection)	Quality assessment score (Comparability)	Quality assessment score (Exposure/Outcome)
Schoon 2013	Case-control	8 (high quality)	* * * *	* *	* *
Sharma 2017	Cohort	7 (high quality)	* * *	* *	* *
Shaw 2015	Cross-sectional	5 (intermediate quality)	* * *	*	*
Sibley 2014	Cross-sectional	6 (intermediate quality)	* * *	* *	*
Song 2021	Cohort	8 (high quality)	* * * *	* *	* *
Sorock 1983	Case-control	6 (intermediate quality)	* * *	* *	*
Soysal 2016	Cross-sectional	7 (high quality)	* * * *	* *	*
Stenhagen 2013	Cohort	7 (high quality)	* * * *	* *	*
Subramanian 2020	Cross-sectional	5 (intermediate quality)	* * * *	-	*
Susman 1989	Cross-sectional	7 (high quality)	* * * *	* *	*
Svensson 1992	Cross-sectional	7 (high quality)	* * * *	* *	*
Swanenburg 2010	Cohort	8 (high quality)	* * *	*	* * * *
Teoh 2021	Cohort	8 (high quality)	* * * *	* *	* *
Thapa 2022	Cross-sectional	6 (intermediate quality)	* * *	-	* * *
Tinetti 1986	Cohort	5 (intermediate quality)	* * * *	*	-
Tsai 2021	Cross-sectional	6 (intermediate quality)	* * * *	* *	-
Turusheva 2020	Cross-sectional	8 (high quality)	* * * *	* *	* *
Valderrama-Hinds 2018	Cohort	6 (intermediate quality)	* * *	* *	*
van der Velde 2007 (a)	Cross-sectional	6 (intermediate quality)	* * *	* *	*
van der Velde 2007 (b)	Cohort	9 (high quality)	* * * *	* *	* * *
Vieira 2018	Cross-sectional	8 (high quality)	* * * *	* *	* *
von Heideken Wagert 2009	Cohort	10 (high quality)	* * * *	* *	* * * *
Wallace 2017	Cohort	8 (high quality)	* * * *	* *	* *
Welmer 2020	Cohort	8 (high quality)	* * * *	* *	* *
Wong 2014	Cohort	9 (high quality)	* * * *	* *	* * *
Xu 2015	Cohort	10 (high quality)	* * * *	* *	* * * *
Yasumura 1994	Cross-sectional	7 (high quality)	* * *	* *	* *
Yi 2021	Cross-sectional	8 (high quality)	* * * *	* *	* *
Yoo 2016	Cross-sectional	6 (intermediate quality)	* * *	-	* *
Yu 2009	Cross-sectional	7 (high quality)	* * * *	* *	*
Zhao 2019	Cross-sectional	7 (high quality)	* * * *	* *	*
Zhao 2020	Case-control	7 (high quality)	* * * *	* *	*
Zhu 2016	Cross-sectional	7 (high quality)	* * * *	* *	*
Zia 2015	Case-control	9 (high quality)	* * * *	* *	* * *

Revised Cochrane risk of bias tool for randomized trials (RoB 2)

	Domain					
**Study**	1: Risk of bias arising from the randomization process	2: Risk of bias due to deviations from the intended interventions	3: Missing outcome data	4: Risk of bias in measurement of the outcome	5: Risk of bias in selection of the reported result	Overall risk of bias
Kenny et al., 2001	Some concerns	Some concerns	Low	Low	Some concerns	Some concerns
Parry et al., 2009	Low	Low	Low	Low	Some concerns	Some concerns
Ryan et al., 2010	Some concerns	Low	Low	Low	Some concerns	Some concerns
